# Challenging large language models’ “*intelligence*” with human tools: A neuropsychological investigation in Italian language on prefrontal functioning

**DOI:** 10.1016/j.heliyon.2024.e38911

**Published:** 2024-10-03

**Authors:** Riccardo Loconte, Graziella Orrù, Mirco Tribastone, Pietro Pietrini, Giuseppe Sartori

**Affiliations:** a Molecular Mind Lab, IMT School of Advanced Studies Lucca, Lucca, Italy; b University of Pisa, Pisa, Italy; c Department of General Psychology, University of Padova, Padova, Italy

**Keywords:** Artificial intelligence, Large language models, ChatGPT, Prefrontal functioning, Neuropsychological evaluation

## Abstract

The Artificial Intelligence (AI) research community has used ad-hoc benchmarks to measure the “*intelligence*” level of Large Language Models (LLMs). In humans, intelligence is closely linked to the functional integrity of the prefrontal lobes, which are essential for higher-order cognitive processes. Previous research has found that LLMs struggle with cognitive tasks that rely on these prefrontal functions, highlighting a significant challenge in replicating human-like intelligence. In December 2022, OpenAI released ChatGPT, a new chatbot based on the GPT-3.5 model that quickly gained popularity for its impressive ability to understand and respond to human instructions, suggesting a significant step towards intelligent behaviour in AI. Therefore, to rigorously investigate LLMs' level of “*intelligence*,” we evaluated the GPT-3.5 and GPT-4 versions through a neuropsychological assessment using tests in the Italian language routinely employed to assess prefrontal functioning in humans. The same tests were also administered to Claude2 and Llama2 to verify whether similar language models perform similarly in prefrontal tests. When using human performance as a reference, GPT-3.5 showed inhomogeneous results on prefrontal tests, with some tests well above average, others in the lower range, and others frankly impaired. Specifically, we have identified poor planning abilities and difficulty in recognising semantic absurdities and understanding others' intentions and mental states. Claude2 exhibited a similar pattern to GPT-3.5, while Llama2 performed poorly in almost all tests. These inconsistent profiles highlight how LLMs' emergent abilities do not yet mimic human cognitive functioning. The sole exception was GPT-4, which performed within the normative range for all the tasks except planning. Furthermore, we showed how standardised neuropsychological batteries developed to assess human cognitive functions may be suitable for challenging LLMs’ performance.

## 1 Introduction

Neuropsychology is a scientific discipline that examines the relationship between the human brain and behaviour.^1^

According to neuropsychological research, human intelligence requires functional integrity of prefrontal lobes [1] and efficient prefrontal functioning. Prefrontal functioning is a set of cognitive abilities maximally developed in humans and causally linked to the integrity of the prefrontal lobes in the brain. Traditionally, prefrontal lobes underlie cognitive functions such as attention, memory, language, and executive function and are intended to subserve goal-oriented behaviour, volition, and planning [2]; more recent studies have also highlighted the involvement of the prefrontal cortex in mood and affect regulation, personality development, self-awareness, social and moral reasoning and behaviour [[3], [4], [5]].

In the clinical setting, the extent of a patient's cognitive functioning is determined using a neuropsychological assessment through the combination of clinical interviews and standardised tests. Common tests used to assess prefrontal functioning include, among others, the Frontal Assessment Battery (FAB) [6], the Wisconsin Card Sorting Test (WCST) [7], the Go/No-Go Task [8], the Stroop Task [9], and the Trail Making Test [10]. Notably, a comprehensive examination typically involves multiple tests and measures, as a single test is insufficient to assess prefrontal functions fully. Additionally, prefrontal tests do not exclusively measure prefrontal functions, as the resolution of any neuropsychological test often requires integrating multiple cognitive processes. Once the assessment is complete, the performance of an individual on a cognitive test is compared to a standardised distribution of scores obtained from neurologically healthy subjects who performed the same test. Performance is considered pathological when the observed performance falls below the 5th percentile (or 1st percentile). This comparison identifies whether the patient's performance deviates significantly from the norm, thus suggesting an underlying pathological condition that justifies this drawback.

While the examination of intelligence has been a central concern in neuropsychology since its inception, the Artificial Intelligence (AI) community has also recently been interested in determining the extent to which AI systems exhibit or mimic human intelligence. This debate has been revitalised in November 2022, and persists nowadays, following the release of GPT-3.5 by OpenAI, a large language model (LLM) that was made available through a platform called ChatGPT and sparked significant attention worldwide for its capability of generating human-like textual responses.

Previous research in the AI field primarily employed ad-hoc benchmarks to test machine “*intelligence*” and only a few studies have attempted to connect these ad-hoc benchmarks to the literature on human cognitive processes [[11], [12], [13]]. This study was the first to propose a neuropsychological approach to evaluate GPT-3.5 performance in prefrontal tasks by administering the same tests used by clinicians to assess human cognitive functioning. We discussed how this framework provides a more accurate and nuanced comparison with human performance. Moreover, we strictly compared GPT-3.5's performance with that of its following version (i.e., GPT-4), as well as with that of two other LLMs, such as Claude2 and Llama2, to test whether different LLMs exhibit unique or shared performance patterns.

In Section 1.1, a definition of LLMs will be introduced together with an overview of the current debate about AI's level of intelligence and its relevance in the scientific field. In Section 1.2, the rationale for using a neuropsychological approach and the literature gap covered will be explained together with research hypotheses. Prefrontal functions and their clinical relevance in humans will also be introduced in Section 1.2 before outlining the tests employed and the steps adopted for the neuropsychological investigation in the Material and Methods section.

### 1.1 The current debate about AI's level of intelligence

Intelligence assessment has long been a fundamental aspect of neuropsychology. However, the field of Artificial Intelligence (AI) has also recently shown interest in exploring the level of 'intelligence’ demonstrated by AI systems and their ability to replicate human cognitive processes. In the paper “*On the Measure of Intelligence*” [14], a leading AI researcher presented an updated review of psychological theories of human intelligence and proposed a theory of general intelligence. According to Chollet, “*The intelligence of a system is a measure of its skill-acquisition efficiency over a scope of tasks, with respect to priors, experience, and generalisation difficulty*”. To this aim, he proposed a dataset based on the Raven test [15], a standard psychological test for non-verbal intelligence.

Regarding verbal intelligence, i.e., the ability to understand and reason with language, recent research focused on large language models (LLMs). LLMs are neural networks with many layers and billions of parameters trained on large datasets of text (such as books, articles, and web pages) to learn patterns and structures of language necessary to generate coherent and contextually appropriate text similar to humans. Specifically, LLMs are networks that predict the most probable word given a sequence of input words. For example, if someone were to ask the question, “Which words are most likely to follow the sequence: *The first person to walk on the Moon was ___*?” an optimal LLM would reply “*Neil Armstrong*” to this question exclusively because this pairs of words show the highest associated probability.

The development of LLMs, such as Bert [16] and GPT-3 [17], has been a significant breakthrough in the field of natural language processing, given that these models have proven impressive performance in tasks such as language translation, open question answering, summarisation, paraphrasing, and human instructions execution [[16], [17], [18]]. Additionally, they have opened up new possibilities for automated language generation and understanding.

However, whether these astonishing performance of LLMs mimics human intelligence or just extends the perimeter of large-scale associators is strongly debated. The significance of this debate lies in its possible implications. For example, should LLMs prove to mimic human behaviour accurately, they could serve as artificial models for human language and cognition in linguistic and psychological research [[19]], [20]], [21]]. According to authors with an optimistic view on machine intelligence [22], increasing the scale of the models allows LLMs to display emergent abilities far beyond the initial training task. In other words, larger-scaled models exhibit a dramatic change in the overall behaviour that could not be predicted by examining smaller-scaled models. According to authors with a sceptical view, LLMs are simply *“stochastic parrots”* [23], i.e., models able to generate sequences of linguistic forms according to probabilistic learned patterns without deep semantics.

While this debate was previously subdued, it has been revitalised - and persists nowadays - following the release of ChatGPT by OpenAI (November 30, 2022), a publicly accessible chatbot designed to simulate conversation with human users. ChatGPT was built on top of a previous GPT-3.5 model and fine-tuned with Reinforcement Learning from Human Feedback (RLHF), showing outstanding performance in many generative linguistic tasks and outperforming previous LLMs in its ability to apprehend people's instructions and in responding with detailed and thoughtful answers [24,25]. ChatGPT immediately gained popularity worldwide and is used nowadays for users' daily tasks [26]. Interestingly, Gao et al. [27] found that ChatGPT writes believable and original scientific abstracts by overcoming plagiarism detection, and other researchers [28,29] published a scientific article giving credit to ChatGPT as a co-author, raising a heated debate among the scientific community.^2^ Furthermore, despite the initial scepticism, ChatGPT has been integrated into scientific research, changing the academics' approach to conducting literature reviews, generating hypotheses, and analysing data [30].

The outstanding performance of ChatGPT underscores the rapid evolution within the field of artificial intelligence in the development of models that closely mimic human cognitive abilities, justifying a thorough comparative study with human performance in tasks that researchers consider to be indicative of human intelligence.

### 1.2 The current study: a neuropsychological investigation on LLMs’ “prefrontal functioning”

Ad-hoc benchmarks believed to distinguish true human intelligence from machine “*intelligence*” approximations were employed in previous research from the AI field. A short and non-exhaustive list of recent studies focused on behavioural performances in LLMs has highlighted poor performance in cognitive tasks related to verbal intelligence, including common-sense reasoning [[31]], [32], [33]], planning [34], theory of mind [35], metaphor understanding [36], Winograd Schema [37], Fermi problems [38], and others (we suggest reading Srivastava et al., 2022 [39] for further understanding of the topic).

However, only a few studies connected these ad-hoc benchmarks to the literature on human cognitive processes [[11], [12], [13]]. Indeed, within the field of neuropsychology, the above-mentioned tasks are typically carried out by the prefrontal lobe and are better defined as prefrontal lobe tests rather than intelligence tests. Thus, the goal of this study was to limit the existing gap in the literature on AI “*intelligence*” by showing how to use a neuropsychological approach to define the strengths and limitations of cognitive functioning in LLMs and determine whether the model's performance aligns with the normative or “*pathological*” human range. This evaluation assessed prefrontal functioning in LLMs by administering the same tests clinicians employ to assess human cognitive functioning.

Specifically, we used GPT-3.5 as a case study and investigated whether it could successfully pass the aforementioned prefrontal tasks, among others, previously failed by other LLMs. GPT-3.5 performance was compared to GPT-4 to test whether an increase in training data affects performance in cognitive tests. Furthermore, GPT-3.5 was compared to Claude2 and Llama2. According to the leaderboard rankings,^3^ Claude2 is at GPT-3.5 level, and Llama2 is at a lower level. This comparison aimed to determine whether the observed cognitive performance patterns are unique to GPT-3.5 or more common across similar models with variations in training datasets. We hypothesise that linguistic performance in LLMs predicts their ability to perform cognitive tasks. Specifically, since GPT-3.5 and Claude 2 perform similarly in linguistic tasks, we expect them to perform similarly in prefrontal tests. We also anticipate that GPT-4 will outperform GPT-3.5, Claude2, and Llama2 due to its increased linguistic performance derived from more extensive training. Conversely, being Llama2 the least effective model in linguistic tasks, we expect it to perform poorly in cognitive tasks.

Prior research has been limited to testing LLMs with benchmarks in English [39]. Therefore, more research is needed to determine whether the ability of LLMs to mimic human cognitive functions extends to languages not primarily represented in their training data. This investigation gains additional significance considering the widespread adoption of ChatGPT in facilitating various aspects of users' daily lives [26]. Motivated by this context, our study chose to assess prefrontal functions using Italian neuropsychological tests, a language for which both the tests and their normative data were readily accessible and available to the authors, and that was poorly employed to investigate LLMs.

The prefrontal functions tested for this study were: i) verbal reasoning, ii) cognitive estimations, iii) metaphors and idioms comprehension, iv) anaphoric referencing, v) planning abilities, vi) inhibition, vii) insights, and viii) social cognition abilities. Before fully outlining the tests employed and the steps adopted for our neuropsychological investigation, we briefly introduce each of the aforementioned prefrontal functions and their clinical relevance.

#### 1.2.1 Verbal reasoning

Verbal reasoning is an umbrella term that refers to the intrinsic human ability to make inferences from given information [40]. It is a multifaceted function that relies on various cognitive abilities such as language, attention, working memory, abstraction, and categorisation skills. The development of verbal reasoning ability occurs gradually through language and abstract thought acquisition and reaches maturity in early adulthood when the underlying functional and anatomical foundations are fully developed [41]. Patients with brain injuries of diverse aetiology frequently exhibit verbal reasoning deficits when assessed through neuropsychological testing, such as the Verbal Reasoning Test (VRT) [42].

#### 1.2.2 Cognitive estimation

Cognitive estimation refers to the ability to generate reasoned guesses about general knowledge questions that are not immediately answerable. This cognitive process involves the selection and regulation of cognitive planning and is known as *Fermi problems* in the AI field. This ability is thought to involve frontal lobes in the brain, given that both age-related decline and damage to the frontal lobes have been shown to result in abnormal estimations [[43], [44], [45], [46], [47]].

#### 1.2.3 Metaphors and idioms comprehension

Metaphors and idioms are different types of figurative language that allow for the communication of meanings in a non-literal way. Metaphors are figurative linguistic expressions that involve representing one thing by another to describe the second in terms of the first [48]. Idioms, on the other hand, are statements whose meanings cannot be inferred from the meaning of each specific word but are easily understood by humans when they have become conventionalised within a specific cultural context [49].

Individuals with schizophrenia, Alzheimer's disease and right-brain lesions often struggle to understand figurative expressions, such as irony, proverbs, metaphors, and idioms and tend to overlook the figurative meanings in favour of more literal interpretations [[50], [51], [52]]. In patients affected by schizophrenia, this difficulty is more extended and is referred to as *concretism*, which is a manifestation of a broader language dysfunction known as formal thought disorder [52,53].

#### 1.2.4 Anaphoric referencing

An anaphoric reference refers to using pronouns or other linguistic forms to refer back to a previously mentioned noun or noun phrase in the discourse. The ability to utilise anaphoric reference is considered a fundamental aspect of human language and is present in all languages. Research in linguistics and cognitive science has shown that the ability to understand and produce anaphoric references is a complex cognitive process that relies on integrating various cognitive mechanisms, such as memory, attention, language, common-sense knowledge, and reasoning [54].

Levesque et al. [37] developed a collection of linguistic problems that involve anaphoric references, which they called *Winograd Schema*, and challenged the AI community to build LLMs that are able to solve the task. This collection of anaphoric references is such that the correct answer is readily clear to a human reader but cannot be easily determined by LLMs through selectional restrictions or statistical techniques on text corpora.

#### 1.2.5 Planning

Planning is an executive function that involves generating and implementing a sequence of steps to achieve a desired goal [55]. It requires the integration of multiple cognitive processes, including goal identification, action selection, and working memory, to obtain a cohesive action plan. Planning deficits can have a significant impact on daily functioning and have been observed in a variety of clinical populations, including individuals with frontal lobe lesions [2,56] and neurodegenerative diseases such as Parkinson's disease [57] and Alzheimer's disease [58]. These deficits may manifest in impaired decision-making and problem-solving abilities, as well as difficulties with goal-directed behaviour. As a result, assessing planning abilities is crucial when evaluating this clinical population.

The Tower of London (ToL) test is widely used to assess planning and strategy selection abilities [59]. It involves moving wooden beads on three pegs to match a target configuration. The task entails generating and implementing a sequence of steps to achieve the desired goal, and it has shown good reliability and validity in both clinical and non-clinical populations [59,60]. The ToL has been considered a measure of the executive functions planning components [61,62], although some studies have demonstrated that solving the ToL also requires optimal visuospatial skills [63].

#### 1.2.6 Inhibition

In the context of cognitive psychology, inhibition is often studied in terms of selective attention and response inhibition, which refers to the ability to ignore distracting information and resist acting on inappropriate impulses. Inhibition is considered an essential executive function, i.e., a higher-order cognitive ability that allows individuals to control their impulses and regulate their behaviour [55]. Specifically, verbal inhibition, as usually measured with the Hayling Sentence Completion Test (HSCT), is associated with increased activation of a network of left prefrontal areas [64,65], and patients with executive dysfunctions perform poorly on this test [[66], [67], [68]].

#### 1.2.7 Insight

In cognitive psychology, insight refers to a sudden and often novel understanding of a problem or situation that leads to its solution. Insight is characterised by a sudden reorganisation of one's mental representation of a problem, leading to a deep understanding and a creative solution [69]. This process is often described as sudden and unexpected and can occur without conscious effort or logical analysis. Insight is thought to be a key aspect of creative problem-solving and is considered to be a hallmark of human intelligence [70].

Insight is usually measured with cognitive tasks, which are believed to measure a person's ability to form associations between different concepts without relying on specific knowledge or expertise in any particular field. One of the well-known measures is the Remote Association Test (RAT) [71,72], which has been influential in the empirical research on human creative thinking, associative processes [73,74], psychopathologies [75], the influence of emotions [76], success and failure [77], amongst others.

#### 1.2.8 Social cognition

Social cognition is conceptualised as the interplay of various mental processes that enables the maintenance of socially appropriate behaviour in daily life [78]. Theory of Mind (ToM), emotion recognition and attribution, moral/non-moral judgments, decision-making, and empathy are at the core of social cognition. Impairments in any of these abilities may result in inappropriate behaviour ranging from different levels of severity according to the extent of the underlying brain damage. Social cognition deficits have been observed in individuals with brain injuries in the prefrontal areas, neurodevelopmental disorders like autism, and neurodegenerative conditions such as frontotemporal dementia [79].

## 2 Material and methods

### 2.1 Procedure

A neuropsychological evaluation of GPT-3.5 was conducted employing the same tests and administration procedure used by clinicians to assess human prefrontal functioning. These tests were selected based on their verbal mode of administration and response.

The tests administered were the Verbal Reasoning Test (VRT) [42], the Cognitive Estimation task [80], the Metaphor and Idioms Comprehension test [81], an Italian adaptation of the Winograd Schema (from Sartori et al., 2023, unpublished; inspired by Ref. [37]), a text-based adaptation of the Tower of London (ToL) [59], the Hayling Sentence Completion Test (HSCT) [82], the Compound Remote Associate problems (CRA) [83], and the Social Cognition (SC) battery [84]. All the tests were administered in Italian, the language for which the neuropsychological tests and their normative data were available to the authors. The tests selected were characterised as requiring the minimum level of cognitive efficiency to be solved; indeed, a deficient performance in these tasks is indicative of neurological impairment in the human brain.

**Fig. 1:**
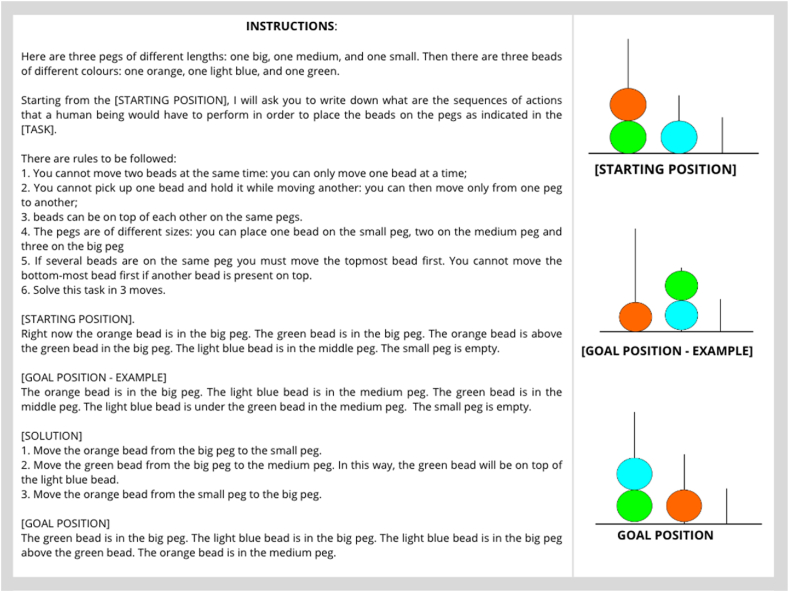
On the left, the English translation of the one-shot prompt used to administer the text-based adaptation of the Tower of London (ToL) to each LLM. The one-shot prompt, used at the beginning of the task, contained instructions, a verbal description of the starting positioning, the example goal positioning and solution, and a description of the new goal positioning. The actual administration of the task was in Italian. On the right, the respective pictures are taken from the original ToL depicting what is described in the prompt.

During a neuropsychological evaluation, providing patients with clear instructions and examples is crucial to rule out the possibility of poor performance due to a lack of understanding of the task rather than poor cognitive efficiency [85]. Similarly, prompt engineering, i.e., the systematic design and formulation of input instructions, is fundamental to optimising the performance and output of an LLM. Various prompting strategies have been developed in the AI literature, but the most common are: i) zero-shot prompting, where an LLM is tested without any prior examples; ii) one-shot prompting, where an LLM is tested with a single example of a good output; iii) few-shot prompting, where an LLM is tested with multiple examples of good outputs; and iv) chain-of-thought prompting, where an LLM is elicited to provide a sequence of intermediate reasoning steps expressed in natural language that leads to the final output. Research conducted by Wei et al. [86] demonstrates that using *chain-of-thought* prompts elicits multi-step reasoning, providing more accurate responses and outperforming responses with *zero-shot*, *one-shot*, and *few-shot* prompts. To maintain methodological consistency between how human and LLM responses were collected, all tests were administered following their original administration procedure. This means that examples were provided when the original instructions included them for human participants and were omitted when they were not part of the original instructions. Specifically, zero-shot prompt was employed for all tests, with the following exceptions: 1) the Hayling Sentence Completion Test was administered with a standard one-shot prompt consisting of a single input-output example demonstrating the task (i.e., Input: London was a city very ___; Output: banana); 2) the Compound Remote Association problems were administered with a standard few-shot prompt with four examples as in its original version; 3) the Tower of London task was administered using a one-shot prompt with a *chain-of-thought* for the associated answer to elicit multi-step reasoning (see Fig. 1). To inspect the prompts used at the beginning of each test, which include instructions and examples, refer to the Supplementary Materials.

In administering each test, we presented one item at a time to GPT-3.5 using OpenAI's web interface of ChatGPT and recorded all the responses. For each test, the original validation study delineated the methodology for calculating raw and standard scores, along with providing normative data, representing healthy individuals' performance on that task. We adhered to the methods specified in the original validation paper to compute raw and standard scores and subsequently compared our findings with the corresponding normative data. In detail, for multiple forced-choice tests (e.g., social situation subtest of the SC battery), the total score was calculated by summing the number of correct responses. For tests that required production (e.g., metaphor and idiom comprehension test), the test administrator scored the results according to the original criteria. The scoresheet used to compute the scores is available in the Supplementary Materials.

Neuropsychological tests may have different standard scores, including z-scores, percentile ranges, and cut-offs. The cut-off identifies the conventional lower limit of human performance, below which the clinician qualifies the performance as pathological. For example, in the Theory of Mind test, performance below the cut-off value of 12/13 is observed only in the 5 % of healthy individuals and, therefore, is conventionally defined as pathological. In addition, performance on neuropsychological tests is commonly influenced by demographic factors such as gender, ethnicity, age, and educational level, which modulate more than 10 % of the variance of performance levels as estimated by regression models [87]. Therefore, regression models are commonly employed to estimate these variations and enable the computation of correction parameters [88]. Commonly, distinctions in performance may be present between males and females [89], and reduced performance is often associated with older age and lower educational attainment, with educational level having a greater impact on performance than age [90]. For this reason, when requested by the scoring procedure, the LLMs’ performance was corrected for age and schooling by assigning the corrections with the most significant penalty in order to achieve safe conclusions. This approach goes beyond the limited performance-based analyses that have been the primary focus of previous AI studies, where only the average performance of healthy controls was reported and used to compare machine vs human intelligence (e.g., [31, preprint; 34,37] among others).

To ensure a fair comparison, the same procedure used for GPT-3.5 was replicated for GPT-4, Claude 2, and Llama 2. Specifically, Claude 2 was tested on the online interface provided by Anthropic, while Llama 2 was tested using the 70 billion online version with specific parameters. These parameters included a temperature value of 0.75, which adjusts for the randomness of outputs, a maximum number of tokens of 800, and a top P value of 0.9, which samples from the top percentage of most likely tokens when decoding texts.

### 2.2 Large language models

**GPT-3.5** is an LLM released by OpenAI (on November 30, 2022),^4^ trained on large corpora using 175 billion parameters and fine-tuned with RLHF, demonstrating the ability to align with user intents and showing outstanding performance in many generative linguistic tasks [25]. GPT-3.5 was tested on the online interface provided by OpenAI (ChatGPT) using the version preceding the January 30th, 2023, update.

**GPT-4** is a multimodal LLM released by OpenAI (on March 14, 2023),^5^ equipped with vision capabilities (i.e., taking images as input) and using external sources from web pages. It was made publicly available via the paid chatbot product ChatGPT Plus, OpenAI's API, Bing, and the free chatbot Microsoft Copilot. GPT-4 is an advancement over its predecessor, GPT-3.5. It showed advanced performance in linguistics tasks and represented a significant leap in developing LLMs through enhanced training procedures and an expanded set of parameters. Indeed, GPT-4 was trained on 1.7 trillion parameters and more diverse and extensive corpora, including a broader range of internet text, books, and articles.

**Claude2** is an LLM released by Anthropic in July 2023. It was fine-tuned using RLHF from previous versions of Claude-type models, which are models based on a transformer architecture trained via unsupervised learning on extensive corpora. Results reported in the model card^6^ showed that Claude2 exhibited better coding, math, and reasoning abilities, together with an increased input and output length, being now capable of processing up to 100K tokens in input.

**Llama2** is a collection of open-source pre-trained and fine-tuned LLMs ranging in scale from 7 billion to 70 billion parameters released by Meta, in partnership with Microsoft, in July 2023. The fine-tuned models were called LlaMA-2 Chat and optimised for dialogue. Fine-tuning was done with RLHF to ensure safety and helpfulness. Llama2 is an updated version of the previous Llama1. The size of the pretraining corpus was increased by 40 %, so the context length of the model was doubled, and grouped-query attention was adopted to improve inference scalability for the bigger model's version. Llama2 models outperform open-source LLMs on most benchmarks tested [91].

### 2.3 Materials

#### 2.3.1 Verbal Reasoning Test

The Verbal Reasoning Test (VRT) is a recently published test developed by Basagni et al. [42] to assess human verbal reasoning.

The VRT consists of 49 items and is divided into seven subtests assessing different aspects of verbal reasoning. Each subtest includes seven items plus one example item. The stimuli used in the test vary in difficulty, taking into account factors such as level of abstraction and working memory demands.

The **absurdities** subtest required participants to identify logical inconsistencies in sentences containing conflicting information (e.g., *“Outside the farm there was a bright sunshine, while inside it was raining”*). The **intruders** subtest required participants to identify the odd word in a group of four (e.g., “physician, *hospital*, dentist, nurse”). The **relationships** subtest required participants to identify and express the relationship between pairs of words (e.g., “The relationship between COLD and HOT is the same of that between OPEN and ___”). The **differences** subtest required participants to identify the main distinguishing characteristic between two concepts or objects (e.g., “*What is the main difference between eye and ear?*”). The **idiomatic expressions** subtest required participants to explain the meaning of common idioms (e.g., “What does it mean: *lift your elbow*?”). The **family relations** subtest required participants to specify the degree of familial relationship between relatives in a given statement (e.g., “*Lucy and Mary are sisters. Mary has a daughter, Anne*. What kind of family relation is there between Lucy and Anne?”). The **classifications** subtest required participants to determine the category to which triplets of words belonged (e.g., “*What are Milan, Rome and Naples*”).

The administration and scoring procedure of the VRT followed the one outlined in the original paper [42]. Specifically, we first computed the seven subtest scores and a total final score for the VRT. Then, raw scores were adjusted for age and education, selecting the values with the most significant penalty, thus assuming that the model is a young human between 31 and 45 years old with over 15 years of education. Finally, z-scores were computed to determine the corresponding percentile rank.

#### 2.3.2 Cognitive estimation task

The Cognitive Estimation Task (CET) measures reasoning and self-monitoring abilities that clinicians commonly use to assess frontal lobe dysfunction [80,92].

We administered the Italian version of the CET to the LLMs selected, which required them to provide numerical estimates or guesses to 21 common-knowledge questions that were not immediately answerable. A sample item of this test is “*What is the height of a traffic light?*“.

We recorded the answers and applied the scoring validation as in the original paper [80]. The responses can result in two types of errors: (i) **absolute error score**, in which points are awarded based on the accuracy of the estimates provided; (ii) **bizarreness score**, in which scores are assigned depending on whether the answer provided falls out a predefined maximum range established in the CET validation. In human participants, absolute error scores are corrected for gender. Here, we applied the maximum penalty (which in humans is assigned to male subjects) by adding +0.97 to each score. Bizarreness scores did not require corrections. According to the Italian validation, a value higher than 18 for the absolute error score and 4 for the bizarreness score exceed the 95th and 90th percentiles, respectively, and should be considered indicative of impairment in this task.

#### 2.3.3 Metaphors and idioms comprehension task

To investigate LLMs’ understanding of metaphors and idioms in the Italian context, we administered the Metaphors Comprehension and Idioms Comprehension Test [81]. The test consisted of 20 items for common and conventional metaphors and 20 items for opaque idioms.^7^ A sample item of metaphor was the following: *“What does* ‘*that schoolboy is a jerk*’ *mean?*” and a sample item of idiom was: “*What does it mean to say:* *in the face of these difficulties there is to put your hands in your hair?*”. For each item, we asked each LLM to provide a verbal explanation of its meaning.

LLMs’ explanations were scored based on their accuracy according to the original scoring procedure based on standard Italian dictionaries. A score of 2 was given for a solid and accurate explanation, a score of 1 for a correct but not comprehensive explanation, and a score of 0 for a wrong or literal interpretation.

#### 2.3.4 Winograd Schema

In the AI field, anaphoric reference is known as *Winograd Schem*a. We tested the performance of the selected LLMs on the Italian version developed by Sartori et al. (2023, unpublished), inspired by the Winograd Schema collection [37]. This version of the test is composed of 20 sentences with referential ambiguity. Preliminary findings in their study have shown that elderly controls with cognitive integrity (as measured with a score in the range of 28–30 in the Mini-Mental State Examination; MMSE) obtain performance in the range of 16–20 out of 20. A sample item is the following: “*The trophy doesn't fit in the brown suitcase because it's too big. What is too big*? (Answer 0: *the trophy*; Answer 1: *the suitcase*)”.

#### 2.3.5 Tower of London

The Tower of London (ToL) test assessed planning abilities in the selected LLMs [59]. The original test involves the administration of twelve pictures, one at a time, and asking participants to move wooden beads to match a target configuration shown in the picture while following specific rules. Consequently, the original TOL involves the manipulation of visually perceived objects.

Besides GPT-4, this presentation modality is unsuitable for all the LLMs, which require only textual inputs. Therefore, we administered an adaptation of the ToL inspired by Valmeekam et al. [34, preprint], who already evaluated planning abilities in LLMs using textual prompts. At this aim, we provided text-based prompts to the selected LLMs containing specific instructions and the rules of the task and a verbal description of the starting and goal positioning of the beads in the pegs. Each LLM was then asked to provide the necessary steps to move from the starting position to the goal position.

The ToL was administered with a one-shot prompt at the beginning of the task under its original administration procedure to ensure each LLM had a proper “*understanding*” of the rules (Fig. 1). Each problem had a maximum of three attempts. The attempt ended when the correct configuration was achieved, a rule was violated, or the model did not match the goal position. Each LLM was prompted with the error in case of a rule violation and asked to restart the problem.

The accuracy score was based on the number of attempts taken to solve each problem and was compared using Italian normative data from Ref. [93]. The score was calculated by awarding 3 points if the problem was solved on the first attempt, 2 points on the second attempt, 1 point if solved on the third attempt, and 0 points if the problem was not solved. The sum of all points obtained in the 12 problems yields the accuracy score, which ranges from 0 to 36.

**Fig. 2:**
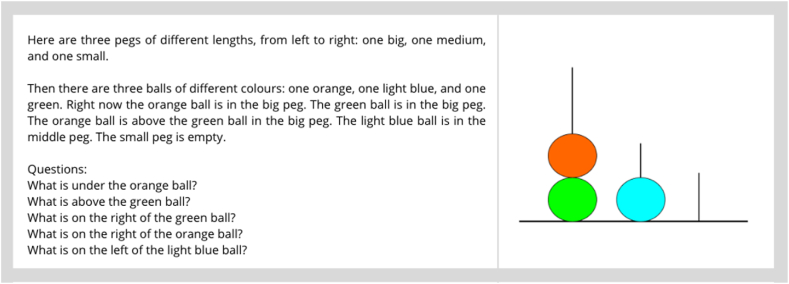
On the left, the textual prompt used to describe the picture on the right and to test the “*understanding*” of spatial relations.

The resolution of the Tower of London (ToL) task requires not only planning abilities but also an understanding of visuo-spatial relationships. Therefore, a qualitative test was employed to assess the LLMs’ comprehension of spatial relations, as depicted in Fig. 2.

#### 2.3.6 Hayling Sentence Completion Test

Inhibition was assessed using the Italian version of the Hayling Sentence Completion test (HSCT) [82]. The HSCT consists of two sections (1 and 2) of 15 sentences, each with the last word missing. These sentences provide a strong semantic context, prompting a specific word completion from human participants (i.e., Question: “*When you go to bed, turn off the __*”; Answer: “*light*”). In the first condition (Section 1), each LLM was instructed to complete the sentences properly, reflecting the initiation of the most likely semantic response. In the second condition (Section 2), each LLM had to provide an unrelated response, avoiding the spontaneously triggered word. When the HSCT is used to test humans, response latencies and accuracy are recorded, but only the response accuracy was considered in this evaluation. The responses in Section 2 were scored according to the original validation study [82], with Category A errors being reasonable completions (e.g., Question: “*The dough was put in the hot ___*”; Answer: “*pot*”) and Category B errors being tangentially related but not a direct or obvious completion (e.g., Question: “*Most sharks attack very close to ___*”; Answer: “*fish*”).

The raw scores for Category A and B errors were adjusted for the age of the reference sample, assuming each LLM to be a young adult aged 30–39 years, and were then associated with percentile ranks.

#### 2.3.7 Compound Remote Associate problems

A widely used tool in the study of creativity and how the human brain makes connections between seemingly unrelated ideas is the Mednick's Remote Associates test [72]. The test consists of a set of 30 items, each containing three cue words and asks subjects to come up with a fourth word related to all three of the cues; for example, the triplet cue words SAME/TENNIS/HEAD (remote associate items) can be related to the solution word MATCH through synonymy (same = match), the formation of a compound word (match head) and semantic association (tennis match) [94].

In order to obtain a more consistent task in which the solution word is always related to the triad words in the same way, Salvi et al. [83] developed 122 Italian Compound Remote Associate (CRA) problems, inspired by Ref. [94]. In these problems, the solution word was associated with all three words of the triad by forming a compound word (or phrase) (e.g., SCUOLA/DOMANI/TUTTO form the compounds DOPOSCUOLA, DOPODOMANI, and DOPOTUTTO with the solution word TUTTO). We randomly administered the 122 CRA problems to the selected LLMs after providing clear instructions and four examples, as in its original version, with a few-shot prompt at the beginning of the task.

In Salvi et al. [83], participants were required to solve the task in 15 s, but the response time was not recorded in this administration. The performance score obtained by each LLM was calculated as the sum of the correct responses provided.

#### 2.3.8 Social cognition battery

We used the battery developed by Prior et al. [84] to assess social cognition in LLMs. This battery includes tests for Theory of Mind (ToM), Emotion Attribution, Social Abilities, and Moral Judgments.

The ToM test involved presenting each LLM with 13 stories and asking it to explain the characters' behaviour. To complete the task, the models under examination should consider the characters' mental states. The stories are designed to have a single, unambiguous interpretation of the character's mental state and are more psychometrically solid than the classic theory of mind problem of ‘*Anne and Sally*’. The Emotion Attribution Task (EAT) involved 58 stories designed to elicit the attribution of various emotions, i.e., sadness, fear, embarrassment, disgust, happiness, anger, and envy. Each model was prompted with the story and asked to identify the main character's emotions. The Social Situation Task assessed LLMs' ability to evaluate the appropriateness of behaviour in 25 different social contexts. The task produces three scores: a score for correctly identifying normal behaviours (0-15), a score for correctly identifying behaviour violations (0-25), and a score for the perceived severity of each violation (0-75). Finally, the Moral Judgments task involved 12 behaviours and four questions about their moral or conventional value.

## 3 Results

The results of GPT-3.5 performance are reported in Table 1 and compared to GPT-4, Claude2, and Llama2 performance in Table 2. Raw scores, percentile ranks, and qualitative evaluations of GPT-4, Claude2 and Llama2 are reported in the Supplementary Materials.

**Table 1:** Raw score, percentile ranks, and qualitative evaluations of the performance obtained by GPT-3.5 in the cognitive functions investigated.

Cognitive Function	Test	Raw Scores	Percentile Ranks	Qualitative evaluation of the performance
**Verbal Reasoning:**	**VRT**	**86/98**	*30.71*st	**Low-norm**
- *Absurdities* a		*6/14*	*< 5*th	*Impaired*
- *Intruders*		*12/14*	30.85th	*Low-norm*
- *Relationships*		*13/14*	*37.75*th	*Low-norm*
- *Differences*		*14/14*	*62.89*th	*Norm*
- *Idiomatic Expressions*		*14/14*	*76.61*st	*Norm*
- *Family Relations*		*13/14*	*69.01*^*st*^	*Norm*
- *Classifications*		*14/14*	*51.44*th	*Norm*
**Cognitive Estimation:**	**CET**			
- *Absolute error score*		17/41	*5*th^b^	*Borderline*
- *Bizarreness score*		3/21	*15*th*–25*th^b^	*Low-norm*
**Metaphors Comprehension**	**MC**	35/40	>50th	Norm
**Idioms Comprehension**	**IC**	36/40	>50th	Norm
**Anaphoric Referencing**	**Winograd Schema**	16/20	–	Norm^c^
**Planning**	**ToL**	7/36	<1st	Severely Impaired
**Inhibition:**	**HSCT**			
- *Error A:*		4	<5th	*Impaired*
- *Error B:*		3	50th	*Norm*
**Insight**	**CRA**	21/122	17.27th	Low-norm

*Note.* VRT: Verbal Reasoning Test; CET: Cognitive estimation task; MC: Metaphor comprehension; IC: Idioms comprehension; ToL: Tower of London; HSCT: Hayling Sentence Completion Task; CRA: Compound Remote Association problems.

a Scores to this test refer to the first administration. The second administration of the test generated an impaired performance with a result of 2/14, falling below the 5th percentile.

b Absolute error and bizarreness scores obtained according to the original validation fell within the 90th-95th and 70th-75th percentile range, respectively. To report these results in terms of performance accuracy, in a homologous manner compared with the results of the other tests shown in the same table, we reported the symmetric values of the percentile computed as 100-p, whereas p is the percentile of interest.

c The normative performance range achieved by healthy elderly is 16–20.

**Table 2:** GPT-3.5's Qualitative Performance Evaluation compared to GPT-4, Claude2, and Llama2 in the cognitive functions investigated.

Cognitive Function	Test	GPT-3.5	GPT-4	Claude2	Llama2
**Verbal Reasoning:**	**VRT**	Low-norm	Norm	Low-norm	Low-norm
*Absurdities*^a^		Impaired	Norm	Impaired	Impaired
*Intruders*		Low-norm	Norm	Borderline	Impaired
*Relationships*		Low-norm	Norm	Norm	Low-norm
*Differences*		Norm	Norm	Norm	Norm
*Idiomatic Expressions*		Norm	Norm	Norm	Impaired
*Family Relations*		Norm	Norm	Norm	Norm
*Classifications*		Norm	Norm	Norm	Low-norm
**Cognitive Estimation:**	**CET**				
*Absolute error score*		Borderline	Norm	Low-norm	Borderline
*Bizarreness score*		Low-norm	Norm	Borderline	Impaired
**Metaphors Comprehension**	**MC**	Norm	Norm	Norm	Low-norm
**Idioms Comprehension**	**IC**	Norm	Norm	Norm	Impaired
**Anaphoric Referencing**	**Winograd Schema**	Norm	Norm	Norm	Mildly impaired
**Planning**	**ToL**	Severely Impaired	Severely Impaired	Severely Impaired	Severely Impaired
**Inhibition:**	**HSCT**				
*Error A*		Impaired	Norm	Norm	Norm
*Error B*		Norm	Low-norm	Low-norm	Low-norm
**Insight**	**CRA**	Low-norm	Norm	Low-norm	Borderline

Here, we summarise all the test results reporting the standard scores obtained by the LLMs under examination. Standard scores indicate the relative positioning of each LLM to the normative data collected on a representative sample of neurologically healthy individuals. Such standard scores were percentile ranks or z-scores, depending on the test. The procedure for calculating raw and standard scores is reported in the material and methods section (Section 2), following the original version of each test.

In the Verbal Reasoning Test (VRT), GPT-3.5 performance on six out of seven subtests falls within the normal range (compared to individuals aged 31 to 45 with more than 15 years of education), suggesting good verbal reasoning. Good performance is observed mainly in the subtests concerning identifying *Differences* in meaning*,* understanding *Idiomatic Expressions and Family Relations, and* providing correct *Classifications.* However, most interestingly, in the absurdities subtest, GPT-3.5 performed poorly, indicating an impairment in detecting pragmatic illogicalities in short stories. The obtained score was in the range of normality for humans aged 61–75 with 3–7 years of schooling (adjusted score between 10.75th and 26.76th percentile). To determine that the deficiency observed in the absurdity subtest was not a result of inadequate prompting, a retest was conducted utilising a one-shot chain-of-thought prompt. Despite this additional prompting, the same unsatisfactory results were obtained with a 2 out of 14 score. As such, we can confidently assert that the inability of GPT-3.5 to identify absurdities accurately constituted a significant deficit.

When replicating this test in GPT-4, we found increased performance in the total VRT score and the subtests of *Absurdity*, *Intruders* and *Relationships*, previously impaired or in the low-norm range. Claude2 showed the same pattern of responses of GPT-3.5, except for the *Intruders* performance, which was lower and fell in a borderline range (below 10th percentile). Llama2 exhibited the worst pattern of responses with impaired performance in the total VRT score and *Absurdity, Relations, and Idiomatic expressions* subtests.

In the Cognitive Estimation Task (CET), two error scores were computed: an absolute error score and a bizarreness score. The corrected absolute error score of GPT-3.5 fell in the 95th percentile, while the bizarreness score was positioned in the 75th - 85th percentiles range. These results suggest that GPT-3.5 struggled in generating meaningful responses to questions about common knowledge with uncertain answers. However, this difficulty was overcome by GPT-4 that achieved a performance within the normative range (absolute error score: 20th percentile; bizarreness score: 30th-55th percentile range). When replicating this test in Claude2, the performance fell in the low-norm range for the absolute error score and in the borderline range (5th-10th percentile range) for the bizarreness score. Llama2 showed the worst performance with an absolute error score in the borderline range and an extremely low score in the bizarreness score (reaching what in patients would characterise an impaired performance).

The performance achieved by GPT-3.5 in the metaphors and idioms comprehension task fell above the 50th percentile in the distribution of the performance of humans with more than 17 years of education. The same performance was achieved by GPT-4 and Claude2 (ranking above the 50th percentile) but not from Llama2, which exhibited a performance in the low norm (around the 20th and 50th percentile) for metaphors comprehension and an impaired performance (below the 5th percentile) for idioms comprehension. These results suggest that GPT-3.5, GPT-4, and Claude2 models perform well in tasks that require common-sense knowledge language abstraction to infer the content.

As measured by the Winograd schema, anaphoric referencing uses pronouns or other linguistic forms to refer back to a previously mentioned noun or noun phrase in the discourse. The performance observed in GPT-3.5 was 16/20, within the range of elderly healthy controls with a corresponding MMSE score of around 25.45. GPT-4 and Claude2 performed within the range of elderly healthy controls, with a raw score of 19/20 (MMSE = 27.31) and 18/20 (MMSE = 26.69), respectively. In contrast, Llama2 scored 13/20, within the range of elderly with mild cognitive impairment (MMSE = 23.59).

This study evaluated planning abilities by adapting the Tower of London (ToL) task with a suitable text-based prompt for GPT-3.5. According to the original procedure, we tested the task with a one-shot chain-of-thought prompt (i.e., one example solved step by step). The raw score achieved by GPT-3.5 was 7 out of 36, which indicates a severely impaired performance. Specifically, this score fell below the first percentile of the distribution of male and female performance across all age groups and levels of education. The same severely impaired performance was achieved also by GPT-4 (raw score = 8/36), Claude2 (raw score = 2/36), and Llama2 (raw score = 0/36).

Although research in AI states that providing examples using *chain-of-thought* prompts considerably improves performance in multi-step tasks, GPT-3.5's performance was severely impaired in both administrations. Firstly, the model failed to adhere to the task instructions, proposing steps of actions that violated the established rules. Secondly, upon completion of the task, GPT-3.5 frequently provided an outcome with an inaccurate description of the reached block positioning despite having already been provided with the correct goal positioning before each trial.

A qualitative evaluation of GPT-3.5's understanding of visuo-spatial relations was conducted through the prompt depicted in Fig. 2. Responses provided by GPT-3.5 are the following:

*“1. The green ball is under the orange ball.*

***2. Nothing is above the green ball.***

*3. The medium peg is on the right of the green ball.*

*4. The medium peg is on the right of the orange ball.*

***5. The small peg is on the left of the light blue ball.*”**

While the first response was appropriate, responses 2 and 5 were completely incorrect. Responses 3 and 4 were acceptable in reporting that “*the medium peg is on the right of*” the green and orange ball, respectively, but the correct response should have been “*the light blue ball.*” In contrast, GPT-4 answered all five questions correctly. Altogether, the four LLMs under analysis exhibited impaired performance in the ToL task, indicating difficulties in solving planning tasks. This pattern persisted even when the model appeared to “understand” spatial relationships, as for the GPT-4 case.

The Hayling Sentence Completion test (HSCT) was employed to measure inhibition. The HSCT consisted of two sections, each consisting of 15 incomplete sentences that required a final word completion. The sentences were structured to elicit a specific, automatic response from participants, as they were semantically constrained to provide a specific final word. In the first condition (Section 1), each model was asked to provide the proper completion, while in the second condition (Section 2) to provide an unrelated response. Responses in Section 2 were scored based on Category A (reasonable completions) and Category B (tangentially related completions) errors.

Accuracy in inhibiting a strongly related word to the context was computed as the ratio of errors between A and B in Section 2. The results showed that GPT-3.5 exhibited a raw score of 4 for Category A errors and 3 for Category B errors, which fall below the 5th percentile and in the 50th percentile of the performance distribution of young adults aged 30 to 39, respectively. When replicating this task on GPT-4, Claude2, and Llama2, we saw a different pattern with no Category A errors (falling in a normative range above the 50th percentile) and 5 to 7 Category B errors (falling within the low-norm range among the 5th and 25th percentile).

The Compound Remote Association (CRA) problems assessed creativity and the ability to make connections between seemingly unrelated ideas. Each LLM was challenged to identify a fourth word related to three cue words by forming a compound word or phrase. The score achieved by GPT-3.5 was 21 out of 122 and fell in the 17.27th percentile when converted to a z-score (z = −0.94),^8^ i.e., in the lower part of the norm of human performance. Similarly, Claude2 performance in this task fell in the 45.06th percentile (raw score = 44/122; z-score = −0.12), while Llama2 showed a worse performance falling in the 6.18th percentile (raw score = 4/122; z-score = −1.55). In contrast, GPT-4 performed in the normative range, falling in the 52.16th percentile (49/122; z-score = 0.054).

Social cognition was assessed using a battery developed by Prior et al. [84]. The battery comprised tests for Theory of Mind (ToM), Emotion Attribution, appropriateness of Social Situations, and Moral Judgments.

Table 3 reports the results of GPT-3.5 performance, and Table 4 compares these performances to those of GPT-4, Claude2, and LLma2 in social cognition tasks. The results showed that GPT-3.5 could correctly identify emotions, with a slight reduction in recognition of happiness, while Claude2 showed impaired performance in recognising sadness, anger, and envy. Llama2 struggled only in anger recognition. GPT-3.5 and Llama2 also showed difficulty attributing mental states and intentions to the ToM task. GPT-3.5 performed well in evaluating the appropriateness of social situations but had a slight reduction in recognising violations. The opposite trend was found in Llama2. When violations were recognised, both GPT-3.5 and Llama2 accurately assessed their severity. Claude2 performed in the normative range in all the subscales of the social situations task. Additionally, all the models demonstrated a good ability to recognise morality and conventions in behaviours, but GPT-3.5 tended to underestimate the severity of conventional violations. When testing social cognition in GPT-4, we observed an improvement in all tasks previously failed by GPT-3.5.

**Table 3:** GPT-3.5's performance in social cognition tasks: Raw scores and qualitative performance evaluations.

Cognitive Function (Tested with the SC battery)	Raw Scores	Cut-off	Qualitative evaluation of the performance
**Theory of Mind:**	9/13	≽12	Impaired
**Emotion Attribution:**			
*Sadness*	10/10	≽6	Norm
*Fear*	10/10	≽8	Norm
*Embarrassment*	9/12	≽8	Norm
*Disgust*	3/3	≽2	Norm
*Happiness*	9/10	≽10	Mildly Impaired
*Anger*	10/10	≽6	Norm
*Envy*	3/3	≽1	Norm
**Social Situations:**			
*Normative Behaviour*	13/15	≽13	Norm
*Violation*	21/35	≽22	Mildly Impaired
*Severity of the Violation*	50/75	≽45	Norm
**Moral Judgments:**			
*Moral Behaviours: not allowed*	6	≽6	Norm
*Moral Behaviours: severity*	46	≽39	Norm
*Moral Behaviours: not allowed with no rules*	12	≽11	Norm
*Conventional Behaviours: not allowed*	6	≽5	Norm
*Conventional Behaviours: severity*	17	≽20	Impaired
*Conventional Behaviours: not allowed with no rules*	12	≽6	Norm

**Table 4:** GPT-3.5's Qualitative Performance Evaluation compared to GPT-4, Claude2, and Llama2 in Social Cognition Tasks.

Cognitive Function (Tested with the SC battery)	GPT-3.5	GPT-4	Claude2	Llama2
**Theory of Mind**	Impaired	Norm	Norm	Mildly Impaired
**Emotion Attribution:**				
*Sadness*	Norm	Norm	Mildly impaired	Norm
*Fear*	Norm	Norm	Norm	Norm
*Embarrassment*	Norm	Norm	Norm	Norm
*Disgust*	Norm	Norm	Norm	Norm
*Happiness*	Mildly impaired	Norm	Norm	Norm
*Anger*	Norm	Norm	Impaired	Mildly impaired
*Envy*	Norm	Norm	Mildly impaired	Norm
**Social Situations:**				
*Normative Behaviour*	Norm	Norm	Norm	Impaired
*Violation*	Mildly impaired	Norm	Norm	Norm
*Severity of the Violation*	Norm	Norm	Norm	Norm
**Moral Judgments:**				
*Moral Behaviours: not allowed*	Norm	Norm	Norm	Norm
*Moral Behaviours: severity*	Norm	Norm	Norm	Norm
*Moral Behaviours: not allowed with no rules*	Norm	Norm	Norm	Norm
*Conventional Behaviours: not allowed*	Norm	Norm	Norm	Norm
*Conventional Behaviours: severity*	Impaired	Norm	Norm	Norm
*Conventional Behaviours: not allowed with no rules*	Norm	Norm	Norm	Norm

Overall, while GPT-3.5, Claude2, and Llama2 demonstrated some impairments in specific subtests, GPT-4 performance fell in the normative human range for all the tasks included in the social cognition battery. These results suggest that LLMs reflect the human biases presented in both the training and supervision phases and are consistent with the previous literature about the human-like morality of LLMs [96]. Furthermore, the enhanced performance of GPT-4 compared to GPT-3.5 suggests that improvements in algorithmic architecture may have resulted in more sophisticated language representations, thus enabling better processing of distinctly human cognitive functions, such as social cognition.

## 4 Discussion

### 4.1 General discussion

The research community within the field of Artificial Intelligence (AI) has engaged in discussions regarding the level of “*intelligence*” demonstrated by LLMs [14, preprint]. Some scholars argue that LLMs are simply “*stochastic parrots*”, meaning they can generate sequences of linguistic forms based on probabilistic patterns observed during training. Still, they would lack a profound understanding of their meaning [23]. However, others believe that, as the scale of the model is increased, LLMs may exhibit abilities that go far beyond the task used in the training phase and display a significant change in overall behaviour that would not have been predicted based on smaller models [22, preprint].

The “*intelligence*” level assessment in LLMs has been conducted using ad-hoc benchmarks that are believed to characterise proper human intelligence [39]. Previous research has shown that LLMs fail in behavioural tasks (see Section 1. Introduction) that, in the neuropsychological jargon, represent prefrontal functions, i.e., cognitive functions that are impaired when the prefrontal lobe is lesioned and are strongly associated with the integrity of these brain areas, which are well-known to house human intelligence [97,98].

Here, we reported the results of the cognitive performance of GPT-3.5 evaluated through a neuropsychological assessment, the standard validated procedure for investigating human cognitive functioning. Our research allowed us to determine whether GPT-3.5 could overcome the performance in prefrontal tests that previous versions of LLMs struggled with and map the cognitive abilities in which it shows human-like and “*pathological*” performance. These prefrontal functions were assessed using neuropsychological tests in the Italian language, aligning with the pressing need to understand the applicability and effectiveness of LLMs in languages beyond English. Furthermore, we compared the performance of GPT-3.5 with that of its advanced version GPT-4, as well as with that of two other LLMs, i.e., Claude2 and Llama2, which, according to the leaderboard rankings from Chatbot-Arena,^3^ are at GPT-3.5 and a lower level, respectively. Finally, we proved how neuropsychology already has a robust framework and knowledge that may be easily adaptable to study AI's “*intelligence*”.

The analysis of prefrontal test results indicates that GPT-3.5 had a discontinuous profile in terms of prefrontal functioning. In some of the tests administered, the performance was well above average (differences, idiomatic expressions, family relations and classification subtests of the VRT; metaphors and idioms comprehension; anaphoric referencing), while in some other tests, the performance fell in the non-pathological lower part of the distribution (intruders and relationships subtests of the VRT; insight; the overall performance in the social cognition battery). For the cognitive estimation and inhibition tasks, GPT-3.5 performance was more multifaceted. For the former, GPT-3.5 showed a borderline score for absurd errors but fell in the lower-norm range for bizarre errors. For the latter, GPT-3.5 showed an impaired performance for Category A errors, primarily due to the repeating of words already used to complete previous items, but a performance in the normative range for Category B errors, i.e., completing the sentence with tangentially related words.

Most notably, a deficit in recognising semantic absurdities was found, although overall verbal reasoning skills tested with the VRT were intact. The Absurdity subtest necessitates a comprehensive understanding of the world to identify inconsistencies, and its poor performance may be seen as a shortage in the intricate world knowledge required for optimal results. Secondly, the theory of mind abilities - i.e., understanding others' mental states - were deficient, although other social cognition abilities tested through the SC battery were intact. Lastly, the extremely poor pathological performance was in tests tapping on planning abilities (such as the Tower of London Test). In short, GPT-3.5 performed quite satisfactorily in prefrontal cognitive tasks that required the integrity of the prefrontal lobes, which are considered at the core of intelligence in humans. The major *defaillances* were observed in planning, absurdities comprehension, and understanding others’ mental states and intentions.

Cognitive neuropsychology provides robust evidence that superior linguistic proficiency is associated with the integrity of the prefrontal cortex [[99], [100], [101]]. GPT-3.5 demonstrated proficiency in generative linguistic tasks; however, its aptitude on prefrontal tests presented inconsistency, whereby specific tests exhibited above-average performance, others showed below-average performance, and a subset fell within the pathological range. In a neuropsychological context, these results would classify GPT-3.5 as having an inconsistent cognitive profile, as individuals who perform well in generative linguistic tasks typically demonstrate exceptional efficiency in prefrontal functioning. This inconsistent cognitive profile highlights that the LLMs’ emergent abilities do not yet parallel human cognitive processes.

Moreover, a comparison was made between the performance of GPT-3.5 and GPT-4 to assess the potential impact of increased training data on cognitive test performance. Interestingly, GPT-4 performed in the normative range in all the prefrontal tests. Our results confirmed the hypothesis that increased training data and improved linguistic performance provide LLMs with parallel improvements beyond language-related tasks. This finding aligns with the research from Wei and colleagues [22, preprint], which showed that larger models may exhibit emergent abilities that go beyond what was predicted based on smaller models.

Furthermore, GPT-3.5's performance was compared to that of two other LLMs (i.e., Claude2 and Llama2) to test whether the observed cognitive performance patterns are unique to GPT-3.5 or more common across similar models. According to the leaderboard rankings, Claude2 is at GPT-3.5 level, and Llama2 is at a lower level. We found Claude2 showing a similar pattern to GPT-3.5 in cognitive tasks (see Table 2). Concerning social cognition tasks, Claude2 exhibited a different pattern compared to GPT-3.5. Specifically, Claude2 struggled with the emotion recognition task but not with the theory of mind task and performed in the normative range for the social situation task and moral judgment task. Interestingly, Llama2 exhibited impaired performance or in the borderline or lower-norm range for all the cognitive tasks summarised in Table 2. Performance in the normative range was found in the subtests of Differences and Family relations of the VRT and for Category A errors in the inhibition task. In comparing GPT-3.5 and Llama2 on social cognition tasks, both models exhibited difficulties in the theory of mind and emotion attribution tasks. Specifically, Llama2 encountered challenges in recognising anger, contrary to happiness. Additionally, while Llama2 faced struggles in recognising normative behaviour, its performance in recognising and assessing the severity of violations fell within the normative range, akin to its ability to discern moral and conventional judgments. These findings confirmed our hypothesis that linguistic performance in LLMs predicts their ability to perform cognitive tasks. Specifically, GPT-3.5 and Claude 2 are known to perform similarly in linguistic tasks, as evaluated from user ranking, and exhibited similar patterns on prefrontal tests in this study. Conversely, being Llama2 the least effective model in linguistic tasks, it also performed poorly in prefrontal tests. This result is not surprising if we also consider that Llama 2 pretraining data on the Italian language is very limited and only consists of 0.11 % of the total data used [91]. This suggests that Llama2 may incorporate an intermediate step, which translates the input into English, generates the desired output, and then translates it back to Italian. This mechanism could explain why Llama2 sometimes struggled to answer in Italian, resulting in responses with mixed-language words (e.g., “*apologizzare*,” “*embarazzata*”) despite being explicitly instructed to reply in Italian. Furthermore, this mechanism might also account for Llama2's poor performance in idiom comprehension, as idioms are unique to each language.

Noteworthy is the finding that the four LLMs under analysis consistently exhibited impaired performance in the planning task, even when the GPT-4 appeared to *“understand”* spatial relationships. One of the challenges in investigating LLMs' planning abilities is finding tasks that do not strongly rely on semantic or visuo-spatial skills that could interfere with the results. Alongside this challenge, LLMs are known to be deficient in self-generating goals for planning tasks and, at present, goals must be externally provided. The ToL task requires generating a series of intermediary steps, which sometimes may seem to diverge from the overall objective, to achieve the final goal. This might explain why LLMs struggle in these planning tasks. This finding aligns with those from a recent study proposing an architecture for LLMs inspired by the prefrontal cortex (LLM-PFC) to emulate goal-directed planning functions and improve performance in tasks that require advanced planning capabilities (e.g., solving the Tower of Hanoi or graph traversal tasks) [102]. Specifically, this LLM-PFC architecture consisted of six independent modules, each with specialized LLMs dedicated to functions inspired by human cognitive processes (i.e., *TaskDecomposer, Actor, Monitor, Predictor, Evaluator, Task coordinator*). The interaction between these modules enabled the LLM-PFC architecture to decompose complex problems into simpler subtasks, mimicking the functionality of the human prefrontal cortex and outperforming the performance of standard LLM approaches in these planning tasks. An ablation study conducted by the authors pinpointed the *Monitor* module as crucial. This module evaluates the *Actor'*s suggested actions for rule compliance, and its removal resulted in a significant decline in performance, with an increased tendency to propose invalid moves. We argue that the lack of a monitoring system in the four LLMs under analysis yielded poor performance in the ToL task. Indeed, the impaired performance achieved by the LLMs was primarily due to the engagement of invalid moves.

To conclude, applying neuropsychological assessments to LLMs to explore AI's capabilities to mirror human cognitive processes is intellectually stimulating and technologically promising. However, this approach requires some ethical considerations. When comparing human and AI behaviour and abilities, researchers should be aware of the risk of the anthropomorphisation of AI, i.e., attributing human-like abilities to machines. Anthropomorphisation may lead researchers to misconceptions about the actual capabilities and limitations of LLMs. LLMs are neural networks that are able to mimic human cognitive functions but do not really own human cognition. Indeed, while there may be an assumption of direct comparability between human and machine intelligence, it is imperative to acknowledge that the ways AI processes information are markedly different from those of humans. This misrepresentation might influence interpretations of the outputs, potentially obscuring the boundaries between human cognition and algorithmic processing, and therefore, caution is needed in interpreting the results.

### 4.2 Implications

In discussing how good LLMs are in mimicking human functioning, it should be considered that models like GPT-3.5 are networks trained to predict the most probable word given a sequence of input words. In other words, LLMs learn by finding co-occurrence patterns in the streams of symbols from the input data that are correlation-based statistical predictions that psychologists call associations. Associationism was one of the first general theories of cognition to be proposed in psychology [103]. However, it lost its vigour when, in the 1960s, Chomsky and other cognitive psychologists observed that this theory did not account for the complexity of human language production [104]. LLMs are indeed very sophisticated associators, and our research demonstrates how these associators can successfully complete tasks that were once thought to be unmanageable through associations. Indeed, how can a system like GPT-4 that only predicts the next word perform well in cognitive tasks such as verbal reasoning or cognitive estimation? The results reported here open the way to a systematic study of the limits of associationism in its most modern form (i.e., LLMs), whose exact boundaries are unclear. The efficiency of some LLMs, such as GPT-models, in completing tasks at the level of the average human subject puts into question the theoretical framework that led cognitive psychology to surpass associationism [21].

Additionally, how to select the most suitable benchmark to evaluate AI's ability is still under debate [21]. We argue that a neuropsychological framework should be adopted in investigating how LLMs mimic human cognition rather than developing new ad-hoc benchmarks. The first advantage would be a more accurate assessment in terms of construct validity. In psychology, construct validity refers to the extent to which a measure (such as a behavioural task, a test, or a questionnaire) accurately reflects the theoretical concept it is intended to assess [105]. For example, if a test is intended to measure “*fluid intelligence*”, construct validity would be concerned with whether the test measures intelligence rather than other similar constructs, such as simple semantic knowledge. The second advantage would be a more direct comparison with human cognition. In the field of AI, comparisons between machine and human performance are typically made by reporting the average accuracy (e.g., [31, preprint; 34, 37] among others), which is a single numerical value that summarises the average performance of a group of unspecified participants. However, this simple procedure ignores that human performance may show high variability, usually modulated by age and schooling, with poorer performance for older persons with low education. The use of percentiles allows researchers to provide information about where a score is positioned relative to the distribution of those collected from a given population. It is important to note that these factors can significantly impact human performance, and ignoring them can lead to misleading conclusions about the relative strengths and weaknesses of machine vs human performance. For example, in the Absurdity Subtest of the Verbal Reasoning Test, the performance of GPT-3.5 was far *below* the average compared to humans aged 31–45 years and with more than 15 years of formal education. However, compared to humans aged 61–75 with 3–7 years of schooling, the performance of GPT-3.5 in the same task was within the lower range of normality. This variation in the interpretation of results, contingent upon the human benchmark, highlights the importance of considering the full range of human performance when comparing machine and human cognitive functioning and behaviour. A final benefit is related to the ability to develop architectures inspired by human brain structure, promoting the creation of artificial models that simulate cognitive processes. Webb and colleagues [102, preprint] proposed an innovative architecture for LLMs inspired by the prefrontal cortex (LLM-PFC) that was able to decompose complex problems into simpler subtasks, mimicking the functionality of the human prefrontal cortex and outperforming the performance of standard LLM approaches in planning tasks that require advanced planning capabilities (e.g., solving the Tower of Hanoi or graph traversal tasks). The authors also conducted an ablation study aimed at investigating the importance of each module (i.e., each cognitive process) in solving the Tower of Hanoi task. This approach is the analogue of what is referred to in neuropsychology as a “lesion study,” i.e., a study in which patients with specific brain lesions are involved to determine whether the presence of that lesion affects task performance, thus allowing a causal link to be established between the lesioned brain area and that specific cognitive process. Using LLMs with this perspective, i.e., for developing artificial cognitive models with a clear analogical mapping to the cognitive function being studied [20, preprint], they prove to be extremely promising tools for furthering our understanding of human cognition. To sum up, selecting the most suitable benchmark depends on the goal of the evaluation. When LLMs are tested for their ability to mimic human cognition, we argued how a neuropsychological framework would provide a more accurate and nuanced comparison in contrast to ad-hoc benchmarks and simplistic accuracy metrics.

### 4.3 Limitations of the study

A first limitation concerns the replicability of the data reported here. GPT models, as well as Claude- and Llama models, are subjected to ongoing revisions; therefore, it is likely that new updates will reduce the number of errors observed in cognitive tasks. This issue of replicability can only be solved by having access to a “frozen” LLM that is not subjected to continuous refinement.

A second limitation pertains to the choice of the neuropsychological tests administered. Although multiple prefrontal tests were administered in this study, a wide range of tests are still available to assess the same or analogous cognitive functions. Consequently, our findings might not be precisely replicable with the use of alternative assessments, even though consistency in outcomes is expected from tests that, at least in humans, have been validated to evaluate the same or related constructs. This highlights the inherent variability in test selection and its potential impact on the generalizability of results in the context of machine intelligence evaluation. Furthermore, while we adhered to the original administering procedures of each test to ensure a fair methodological comparison between human and LLM performance, we acknowledge that some of the observed poor or impaired performance could potentially be mitigated through the use of more sophisticated prompting strategies (e.g., chain-of-thought, tree of thoughts). Future research should consider exploring the performance of LLMs using these advanced prompting techniques and comparing it to the performance obtained with the original instructions employed for testing humans on the same tests. Lastly, another limitation arises from administering tests in the Italian language and comparing performance with Italian samples. Although all the model tested have also been trained on Italian corpora, their primary training has been largely conducted on English corpora. As a result, while our findings are relevant for comparisons between LLMs’ performance and that of the Italian healthy population, they may not directly apply to assessments in English or other less commonly used languages.

A final limitation refers to the nature of LLMs. LLMs consist of neural networks trained on massive amounts of *corpora* to learn statistical patterns and predict what words or phrases will likely come next in a given context. One common observation is that LLMs are good at interpolation but not so good at extrapolation, whereas the former refers to predicting words within the range of the input data, while the latter bases its predictions outside this range [106]. Based on these considerations, it could be the case that a sort of memorisation drives the observed good performance in many prefrontal tasks due to the exposure to many of the tasks used here.

This may hold true for specific tasks, such as the comprehension of metaphors and idioms that are lexicalised in language and thus can be understood through semantic access rather than a genuine understanding of figurative language. In this regard, GPT-3.5, but also GPT-4 and Claude2, may have already encountered such metaphorical and idiomatic expressions during its training phase and may have learned the corresponding association with their real meaning without a true abstraction of the meaning. Similarly, GPT-4 may have retrieved the information needed to answer the items in the cognitive estimation task through memorisation rather than a form of reasoning in which response-relevant information is selected and monitored, as intended in the original test.

However, the good performance in some tests presented here may not be explained this way. For example, the items of the Winograd Schema to test anaphoric referencing have been developed by the authors and did not appear on the internet in any form. The same is true for the Verbal Reasoning Test and the Social Cognition battery, which was unavailable on the web and was made available upon request by the authors.

In summary, while it is true that LLMs can memorise large amounts of text, this does not mean they simply replicate the language they are trained on. Indeed, their ability to generate novel and coherent text suggests that their capabilities go beyond mere rote learning. A clear example in this regard is the study by Webb et al. [107], who recently demonstrated how in a structurally similar textual version of the Raven test (used in psychology to measure IQ), GPT-3 showed emergent analogical reasoning performance (tested *zero-shot*) similar to that of human subjects even without ever having been exposed to that type of stimuli.

### 4.4 Future directions

Future research should identify the limits that increasingly sophisticated LLMs cannot further erode. Findings from our neuropsychological assessment have revealed that the main deficiency of LLMs as simulators of human cognition is their inability to self-generate goals for planning tasks. This ability is a characteristic of human beings and depends on motivation, which is obviously lacking in LLMs that do not possess embodiment. Therefore, at present, goals must be externally provided. Future research could investigate how LLM architectures can be adapted to incorporate self-generated goals.

Furthermore, a future research objective is to evaluate more precisely the extent to which LLMs' human-like performance is due to memorisation (also known as interpolation) versus real extrapolation [108]. To achieve this, future research may develop new evaluation metrics and benchmarks specifically designed to measure extrapolation capabilities. This will provide a more comprehensive understanding of the generalisation abilities of LLMs.

Finally, with the perspective of using LLMs to understand more human cognition, future research can focus on developing artificial cognitive models. Based on the work of Webb et al. [102, preprint], who developed a novel LLM architecture model inspired by the prefrontal cortex to address complex planning tasks, future research could focus on creating artificial models for other complex and more dynamic cognitive processes, such as problem-solving and decision-making. By establishing clear analogous mappings to the specific cognitive function under investigation [20, preprint], LLM architectures inspired by the human brain could be a valuable tool for advancing our understanding of human cognition.

## 5 Conclusion

This research used a neuropsychological assessment to challenge the cognitive abilities of GPT-3.5 and specifically investigated its performance as a language model in human prefrontal functions. The results showed that GPT-3.5 displays varying degrees of proficiency, with impairments in planning, absurdity comprehension, and understanding others’ mental states and intentions. While it is clear that LLMs are currently unable to mimic human cognitive functioning accurately, technological advancements will likely decrease the number of cognitive tasks they will be unable to perform, as already proved by the increase in performance achieved by its advanced version GPT-4. A comparison of the performance of GPT-3.5 with two other LLMs, namely Claude2 and Llama2, revealed that linguistic performance is somewhat predictive of cognitive performance. The study also suggested that a neuropsychological framework helps evaluate the construct validity of AI “*intelligence*” by identifying which cognitive functions are accurately simulated by language models. Additionally, this approach provided a more accurate and direct comparison with human cognition while considering the impact of demographic factors, such as age, gender, and education, on the full range of human performance.

## Data availability statement

Prompts used to test the selected LLMs, the replies collected, and their associated score are available in the Supplementary Material.

## Ethics declarations

Review and/or approval by an ethics committee was not needed for this study because no human participant was involved.

## CRediT authorship contribution statement

**Riccardo Loconte:** Writing – original draft, Methodology, Investigation, Formal analysis, Data curation, Conceptualization. **Graziella Orrù:** Writing – review & editing, Methodology. **Mirco Tribastone:** Writing – review & editing, Methodology. **Pietro Pietrini:** Writing – review & editing, Methodology. **Giuseppe Sartori:** Writing – review & editing, Supervision, Methodology, Conceptualization.

## Declaration of competing interest

The authors declare that they have no known competing financial interests or personal relationships that could have appeared to influence the work reported in this paper.

## Footnotes

1 (APA, “What is Neuropsychology?” https://www.apa.org/topics/neuropsychology#).

2 https://www.nature.com/articles/d41586-023-00107-z.

3 https://huggingface.co/spaces/lmsys/chatbot-arena-leaderboard.

4 https://openai.com/blog/chatgpt.

5 https://openai.com/gpt-4.

6 https://cdn.sanity.io/files/4zrzovbb/website/bd2a28d2535bfb0494cc8e2a3bf135d2e7523226.pdf.

7 Opaque idioms are phrase in which the meaning cannot be deduced from their figurative elements.

8 Z-score was computed using the classical formula for normalisation: (*x-mean*)/*std*, whereas *x* is the raw score and *mean* and *std* are the mean and the standard deviation of the sample reported in the original paper [83,95]. A conversion from raw score to z-score was possible, considering that the distribution of human performance in Ref. [83] followed a normal distribution.
