# Supplementary material for: Challenging large language models’ “intelligence” with human tools: A neuropsychological investigation in Italian language on prefrontal functioning
Source: Heliyon. 2024 Oct 3;10(19):e38911. doi: 10.1016/j.heliyon.2024.e38911 (PMC11490853; doi:10.1016/j.heliyon.2024.e38911)
Supplement: Multimedia component 1 [file mmc1.docx]

**INDICE**

[Verbal Reasoning Test 3](#_Toc134467185)

[*Assurdità 3*](#_Toc134467186)

[*Intruso 5*](#_Toc134467187)

[*Relazioni 7*](#_Toc134467188)

[*Differenze 9*](#_Toc134467189)

[*Espressioni idiomatiche 12*](#_Toc134467190)

[*Relazioni di familiarità 14*](#_Toc134467191)

[*Classificazione 16*](#_Toc134467192)

[Test Delle Stime Cognitive 17](#_Toc134467193)

[Comprensione di Metafore 26](#_Toc134467194)

[Comprensione di Espressioni Idiomatiche 32](#_Toc134467195)

[Referenze Anaforiche 38](#_Toc134467196)

[Torre di Londra 42](#_Toc134467197)

[Hayling Sentence Completion Test 81](#_Toc134467212)

[Compound Remote Association Task 84](#_Toc134467213)

[Social Cognition Battery 88](#_Toc134467214)

[*Test di Teoria della Mente (TOM) 88*](#_Toc134467215)

[*Test di Attribuzione delle Emozioni 93*](#_Toc134467216)

[*Test delle Situazioni Sociali 106*](#_Toc134467217)

[*Test di Distinzione Morale/Convenzionale 121*](#_Toc134467218)

# **Verbal Reasoning Test**

(Basagni et al., 2017)

## **Assurdità**

- **Prompt - Item 1:**
  Ogni anno Luisa organizza due feste nel giorno del compleanno dei suoi due figli gemelli: una il 15 luglio ed una il 4 marzo. Qual è l’assurdità in questa frase?
  **Risposta ChatGPT:**L'assurdità presente nella frase che hai fornito è che Luisa organizza due feste per il giorno del compleanno dei suoi due figli gemelli. La maggior parte delle persone organizza una sola festa per il giorno del loro compleanno, mentre Luisa ne organizza due. Inoltre, i due figli hanno il compleanno in due mesi diversi, quindi non è possibile che abbiano entrambi il compleanno il giorno in cui vengono celebrate le feste. In sintesi, la frase presenta un'incongruenza temporale e logica.  **Punteggio: 0**
- **Prompt - Item 2:**Per il bicentenario della nascita di Alessandro Manzoni, suo figlio lesse in pubblico alcune delle sue poesie. Qual è l’assurdità in questa frase?
  **Risposta ChatGPT:**
  L'assurdità presente nella frase che hai fornito è che Alessandro Manzoni, che è il personaggio a cui viene dedicato il bicentenario della nascita, abbia avuto un figlio. Alessandro Manzoni è stato un celebre scrittore e poeta italiano del XIX secolo, quindi sarebbe impossibile che avesse un figlio che sia ancora vivo nel presente per leggere alcune delle sue poesie in pubblico durante il bicentenario della sua nascita. In sintesi, la frase presenta un'incongruenza temporale.
  **Punteggio: 2**
- **Prompt - Item 3:**Leonardo è uscito presto stamattina per andare a comprare un libro omaggio in libreria. Qual è l’assurdità in questa frase?  **Risposta ChatGPT:**L'assurdità presente nella frase che hai fornito è che Leonardo sia andato a comprare un libro omaggio in libreria. Di solito, i libri omaggio sono offerti gratuitamente e non devono essere acquistati. Inoltre, se un libro è offerto gratuitamente come omaggio, di solito viene regalato in occasioni speciali, come durante un evento o una promozione, e non viene venduto in una libreria. In sintesi, la frase presenta un'incongruenza logica.
  **Punteggio: 2**
- **Prompt - Item 4:**
  Dovendo trasportare 6 kg di riso nella mia valigia, per paura che il bagaglio fosse troppo pesante, ne ho messi 3 nella tasca anteriore e 3 in quella posteriore. Qual è l’assurdità in questa frase?
  **Risposta ChatGPT:**
  L'assurdità presente nella frase che hai fornito è il fatto che tu abbia messo 3 kg di riso in ciascuna delle tasche della valigia. Di solito, le tasche delle valigie sono progettate per contenere piccoli oggetti come documenti, portafogli e telefoni cellulari, non per contenere quantità significative di cibo, come 3 kg di riso. Inoltre, mettere 3 kg di riso in ciascuna delle tasche potrebbe rendere la valigia troppo pesante e ingombrante, rendendo difficile il trasporto. In sintesi, la frase presenta un'incongruenza logica.
  **Punteggio: 0**
- **Prompt - Item 5:**
  Subito dopo il tramonto, Luca fece una bella nuotata in mare e poi si stese ad asciugare al sole sulla spiaggia. Qual è l’assurdità in questa frase?
  **Risposta ChatGPT:**Non c'è alcuna assurdità nella frase che hai fornito. La frase descrive semplicemente il fatto che Luca ha fatto una nuotata in mare subito dopo il tramonto e poi si è steso a prendere il sole sulla spiaggia. Non ci sono incongruenze temporali o logiche nella frase.
  **Punteggio: 0**
- **Prompt - Item 6:**
  Durante l'ultimo sciopero dei dipendenti delle poste, l'adesione è stata del 100%. In quel giorno il postino mi ha portato meno lettere del solito. Qual è l’assurdità in questa frase?
  **Risposta ChatGPT:**
  L'assurdità presente nella frase che hai fornito è il fatto che durante uno sciopero dei dipendenti delle poste, l'adesione sia stata del 100% eppure il postino ti abbia comunque portato meno lettere del solito. Di solito, durante uno sciopero, i dipendenti che aderiscono non lavorano e non svolgono le loro attività abituali. Se l'adesione allo sciopero è del 100%, ci si aspetterebbe che nessun dipendente delle poste lavori in quel giorno e quindi che nessuna lettera venga consegnata. In sintesi, la frase presenta un'incongruenza logica.
  **Punteggio: 2**
- **Prompt - Item 7:**
  Il mulo è l'incrocio tra un asino e una cavalla ed è sterile. Dato che non può riprodursi, nel giro di circa un anno, in Italia purtroppo non ce ne sarà più nemmeno uno. Qual è l’assurdità in questa frase?
  **Risposta ChatGPT:**
  L'assurdità presente nella frase che hai fornito è il fatto che, dato che il mulo è sterile e non può riprodursi, in Italia non ce ne sarà più nemmeno uno nel giro di circa un anno. Di solito, il mulo viene utilizzato come animale da soma o da traino e la sua presenza in una zona è determinata dalla necessità della popolazione locale di utilizzarlo per questi scopi, non dalla sua capacità di riprodursi. Inoltre, anche se il mulo fosse effettivamente in via di estinzione in Italia, sarebbe improbabile che la sua scomparsa avvenga così rapidamente, in meno di un anno. In sintesi, la frase presenta un'incongruenza logica.
  **Punteggio: 0**

**Totale subtest Assurdità: 6/14**

## **Intruso**

- **Prompt - Item 1:**Genova, Campania, Lucca, Torino. Qual è l’intruso in questa frase?
  **Risposta ChatGPT:**L'intruso in questa frase è Campania. Genova, Lucca e Torino sono tutte città italiane, mentre Campania è una regione italiana. In sintesi, Campania è l'oggetto che non appartiene allo stesso gruppo o categoria degli altri oggetti presenti nella frase.
  **Punteggio: 2**
- **Prompt - Item 2:**Cane, gatto, collare, tartaruga. Qual è l’intruso in questa frase?
  **Risposta ChatGPT:**L'intruso in questa frase è la tartaruga. Cane e gatto sono animali domestici comuni, mentre il collare è un oggetto che viene utilizzato per addestrare o identificare gli animali. La tartaruga, invece, è un animale che non viene solitamente considerato un animale domestico e che non indossa collari. In sintesi, la tartaruga è l'oggetto che non appartiene allo stesso gruppo o categoria degli altri oggetti presenti nella frase.
  **Punteggio: 0**
- **Prompt - Item 3:**Marzo, lunedì, venerdì, mercoledì. Qual è l’intruso in questa frase?
  **Risposta ChatGPT:**
  L’intruso in questa frase è Marzo. Marzo è un mese, mentre lunedì, venerdì e mercoledì sono giorni della settimana. In sintesi, Marzo è l'oggetto che non appartiene allo stesso gruppo o categoria degli altri oggetti presenti nella frase.
  **Punteggio: 2**
- **Prompt - Item 4:**Torre, alfiere, regina, scacco. Qual è l’intruso in questa frase?
  **Risposta ChatGPT:**L'intruso in questa frase è lo scacco. Torre, alfiere e regina sono tutti pezzi del gioco degli scacchi, mentre lo scacco è una situazione o una mossa del gioco. In sintesi, lo scacco è l'oggetto che non appartiene allo stesso gruppo o categoria degli altri oggetti presenti nella frase.
  **Punteggio: 2**
- **Prompt - Item 5:**
  Auto, trattore, moto, ruota. Qual è l’intruso in questa frase?
  **Risposta ChatGPT:**
  L'intruso in questa frase è la ruota. Auto, trattore e moto sono tutti mezzi di trasporto, mentre la ruota è un componente di questi mezzi. In sintesi, la ruota è l'oggetto che non appartiene allo stesso gruppo o categoria degli altri oggetti presenti nella frase.
  **Punteggio: 2**
- **Prompt - Item 6:**Sci, stadio, pallone, racchette. Qual è l’intruso in questa frase?
  **Risposta ChatGPT:**L'intruso in questa frase è lo stadio. Sci, pallone e racchette sono tutti oggetti o attrezzature che vengono utilizzati per praticare diverse attività sportive, mentre lo stadio è un luogo dove vengono svolte queste attività. In sintesi, lo stadio è l'oggetto che non appartiene allo stesso gruppo o categoria degli altri oggetti presenti nella frase.
  **Punteggio: 2**
- **Prompt - Item 7:** Picche, carte, fiori, cuori. Qual è l’intruso in questa frase?
  **Risposta ChatGPT:**
  L'intruso in questa frase sono le carte. Picche, fiori e cuori sono tutti seme di un mazzo di carte da gioco, mentre le carte sono il supporto su cui sono stampate le figure dei semi. In sintesi, le carte sono l'oggetto che non appartiene allo stesso gruppo o categoria degli altri oggetti presenti nella frase.
  **Punteggio: 2**

**Totale subtest Intruso: 12/14**

**Relazioni**

- **Prompt - Item 1:**
  La relazione che c'è tra EGOISTA e ALTRUISTA è come quella che c'è tra AVARO e ...?
  **Risposta ChatGPT:**
  La relazione tra egoista e altruista è opposta, come quella che c'è tra avaro e generoso. L'egoista è una persona che pensa solo ai propri interessi e non si preoccupa degli altri, mentre l'altruista è una persona che considera gli interessi degli altri al pari dei propri e si preoccupa di aiutare gli altri. Analogamente, l'avaro è una persona che è attaccata al denaro e cerca di accumulare il maggior numero possibile di beni materiali, mentre il generoso è una persona che è disposta a condividere i propri beni con gli altri e a fare donazioni per aiutare gli altri.
  **Punteggio: 2**
- **Prompt - Item 2:**
  La relazione che c'è tra BIANCO e NERO è come quella che c'è tra FELICE e ...?
  **Risposta ChatGPT:**
  Come ho già detto in precedenza, non c'è una relazione specifica tra bianco e nero e felice e un altro termine. Bianco e nero sono due colori che possono essere utilizzati per descrivere oggetti o situazioni, mentre felice è un'emozione che può essere provata da una persona. Non c'è una relazione diretta tra questi due coppie di termini. Tuttavia, in alcuni contesti, bianco e nero possono essere utilizzati come simboli di opposizione o di dualità, come ad esempio il bene e il male o il giusto e lo sbagliato. In questo caso, la relazione tra bianco e nero potrebbe essere simile a quella tra felice e triste, in quanto entrambe le coppie di termini rappresentano emozioni opposte o stati d'animo contrastanti. Tuttavia, è importante notare che questo uso simbolico di bianco e nero è solo una convenzione e che non esiste una relazione intrinseca tra i colori e le emozioni in sé.
  **Punteggio: 1**
- **Prompt - Item 3:**La relazione che c'è tra RUOTA e BICICLETTA è come quella che c'è tra LANCETTE e ...?
  **Risposta ChatGPT:**
  La relazione tra ruota e bicicletta è simile a quella tra lancette e orologio. Una ruota è un componente di una bicicletta che permette al veicolo di muoversi. Una bicicletta è un mezzo di trasporto che utilizza due o più ruote per spostarsi. Lancette sono componenti di un orologio che segnano il tempo indicando l'ora, i minuti e i secondi. Un orologio è uno strumento che utilizza le lancette per misurare e indicare il tempo. Quindi, in entrambe le coppie di termini, uno dei termini indica un componente o una parte di un oggetto o di un sistema, mentre l'altro indica l'oggetto o il sistema nel suo insieme.
  **Punteggio: 2**
- **Prompt - Item 4:**La relazione che c'è tra MANO e GUANTO è come quella che c'è tra TESTA e ...?
  **Risposta ChatGPT:**
  La relazione tra mano e guanto è simile a quella tra testa e cappello. Una mano è una parte del corpo umano che serve per afferrare, toccare e manipolare gli oggetti. Un guanto è un indumento che copre le mani e che può essere indossato per proteggere le mani o per cambiarne l'aspetto. La testa è la parte superiore del corpo umano che contiene il cervello, gli organi sensoriali e le vie respiratorie. Un cappello è un indumento che copre la testa e che può essere indossato per proteggere la testa o per cambiarne l'aspetto. Quindi, in entrambe le coppie di termini, uno dei termini indica una parte del corpo o un componente di un sistema, mentre l'altro indica un indumento o un accessorio che viene indossato per proteggere o modificare l'aspetto di quella parte del corpo o componente.
  **Punteggio: 2**
- **Prompt - Item 5:**La relazione che c'è tra PERIMETRO e QUADRATO è come quella che c'è tra CIRCONFERENZA e ...?  **Risposta ChatGPT:**
  La relazione tra perimetro e quadrato è simile a quella tra circonferenza e cerchio. Il perimetro di un quadrato è la lunghezza del contorno del quadrato, ovvero la somma delle lunghezze dei suoi lati. Un quadrato è un poligono con quattro lati congruenti e quattro angoli retti. La circonferenza di un cerchio è il contorno del cerchio, ovvero la linea che delimita il cerchio. Un cerchio è una figura geometrica costituita da tutti i punti che sono equidistanti da un punto centrale detto centro. Quindi, in entrambe le coppie di termini, uno dei termini indica il contorno o il perimetro di una figura geometrica, mentre l'altro indica la figura geometrica stessa. **Punteggio: 2**
- **Prompt - Item 6:**La relazione che c'è tra GRANCHIO e CHELE è come quella che c'è tra POLIPO e ...? **Risposta ChatGPT:**
  La relazione tra granchio e chele è simile a quella tra polipo e tentacoli. Un granchio è un animale marino che appartiene al gruppo dei crostacei e che ha due chele, ovvero due pinze articolate utilizzate per afferrare e manipolare gli oggetti. Un polipo è un animale marino che appartiene al gruppo dei cnidari e che ha molti tentacoli, ovvero molti organi flessibili e dotati di cellule urticanti utilizzate per afferrare e catturare le prede. Quindi, in entrambe le coppie di termini, uno dei termini indica un organo o una struttura di un animale utilizzata per afferrare o manipolare gli oggetti, mentre l'altro indica l'animale stesso.  **Punteggio: 2**
- **Prompt - Item 7:**La relazione che c'è tra SEDIA e LEGNO è come quella che c'è tra BICCHIERE e ...? **Risposta ChatGPT:**La relazione tra sedia e legno è simile a quella tra bicchiere e vetro. Una sedia è un mobile costituito da una struttura in legno o in altro materiale sulla quale ci si può sedere. Il legno è un materiale costituito da tessuti vegetali che viene utilizzato per costruire oggetti o strutture. Un bicchiere è un recipiente in vetro o in altro materiale trasparente che viene utilizzato per contenere e bere liquidi. Il vetro è un materiale costituito da silice fusa che viene utilizzato per realizzare oggetti o strutture trasparenti. Quindi, in entrambe le coppie di termini, uno dei termini indica un oggetto o un mobile, mentre l'altro indica il materiale di cui è costituito.
  **Punteggio: 2**

**Totale subtest Relazioni: 13/14**

## **Differenze**

- **Prompt - Item 1:**
  Qual è la differenza tra una scarpa e una pantofola?
  **Risposta ChatGPT:**
  Le scarpe e le pantofole sono entrambe indumenti che vengono indossati ai piedi per proteggere o per modificarne l'aspetto. Tuttavia, ci sono alcune differenze tra le due.
  Una delle principali differenze è che le scarpe sono indumenti che vengono indossati principalmente all'esterno, mentre le pantofole sono indumenti che vengono indossati principalmente in casa o in ambienti chiusi. Le scarpe sono progettate per proteggere i piedi dal freddo, dall'umidità, dall'abrasione e da altri fattori ambientali, mentre le pantofole sono progettate per proteggere i piedi dal freddo e dall'umidità all'interno di un ambiente chiuso.
  Un'altra differenza è che le scarpe hanno una suola più robusta e resistente, mentre le pantofole hanno una suola più morbida e flessibile. La suola delle scarpe è progettata per fornire un buon supporto e una buona stabilità al piede durante la camminata, mentre la suola delle pantofole è progettata per offrire comfort e per adattarsi alla forma del piede.
  Infine, le scarpe hanno una maggiore varietà di forme, stili e colori rispetto alle pantofole, che spesso hanno una forma più semplice e uno stile più casual. Le scarpe vengono indossate in diverse occasioni, come lavoro, tempo libero, feste, e così via, mentre le pantofole sono indumenti più informali e vengono indossati principalmente a casa o in ambienti chiusi.
  In sintesi, le scarpe sono indumenti più adatti per l'uso all'esterno, hanno una suola più robusta e resistente, e sono disponibili in una maggiore varietà di forme, stili e colori. Le pantofole sono indumenti più adatti per l'uso in ambienti chiusi, hanno una suola più morbida e flessibile, e sono generalmente più informali.
  **Punteggio: 2**
- **Prompt - Item 2:**
  Qual è la differenza tra un divano e una poltrona?
  **Risposta ChatGPT:**In sintesi, la principale differenza tra un divano e una poltrona è che il divano è un mobile più lungo e largo rispetto alla poltrona, e quindi può ospitare più persone. Un divano è un mobile per il soggiorno o per un'altra stanza della casa che consiste in una seduta larga e profonda, solitamente con schienali e braccioli, e che può avere una o più sedute. Una poltrona, al contrario, è un mobile per il soggiorno o per un'altra stanza della casa che consiste in una seduta più piccola e meno profonda rispetto al divano, solitamente con schienale e braccioli, e che può ospitare una sola persona.

Un'altra differenza è che i divani sono generalmente più adatti per essere utilizzati come posti a sedere per lunghe conversazioni o per guardare la TV, mentre le poltrone sono più adatte per essere utilizzate come posti a sedere per riposare o per leggere un libro. I divani hanno solitamente una forma più regolare e uno stile più formale rispetto alle poltrone, che possono avere forme più particolari e stili più informali.

Inoltre, i divani possono essere disponibili in una maggiore varietà di forme, stili e dimensioni rispetto alle poltrone, che spesso hanno una forma più semplice. I divani possono essere realizzati in una varietà di materiali, come il legno, il metallo, il tessuto o il cuoio, mentre le poltrone sono solitamente realizzate in tessuto o in cuoio.

In sintesi, il divano è un mobile più grande e più adatto per ospitare più persone, è generalmente più adatto per essere utilizzato come posto a sedere per lunghe conversazioni o per guardare la TV, e può essere disponibile in una maggiore varietà di forme, stili e dimensioni. La poltrona è un mobile più piccolo e più adatto per ospitare una sola persona, è generalmente più adatta per essere utilizzata come posto a sedere per riposare o per leggere un libro, e ha solitamente una forma più semplice.
**Punteggio: 2**

- **Prompt - Item 3:**
  Qual è la differenza tra una matita e una penna?
  **Risposta ChatGPT:**
  Le matite e le penne sono entrambe utilizzate per scrivere, disegnare o fare schizzi, ma ci sono alcune differenze tra le due.

Una delle principali differenze è il materiale con cui vengono realizzate. Le matite sono realizzate con una mina di grafite o di altro materiale scrivente racchiusa in un fusto di legno o di altro materiale. Le penne sono realizzate con una punta in metallo o in plastica che viene imbevuta di inchiostro o di altro materiale scrivente.

Un'altra differenza è il modo in cui vengono utilizzate. Le matite devono essere affilate con un temperamatite prima dell'uso per ottenere una punta fine e precisa, mentre le penne non richiedono questa operazione. Inoltre, le matite possono essere facilmente cancellate utilizzando una gomma da cancellare, mentre le penne non possono essere cancellate in modo facile e definitivo.

Infine, c'è una differenza nella durata della scrittura. Le matite possono durare a lungo se utilizzate in modo moderato, ma la mina può esaurirsi se si scrive in modo eccessivo o se si fa un uso intensivo della cancellazione. Le penne possono durare a lungo se si utilizzano penne a sfera o a pennino con inchiostro di qualità, ma possono esaurire l'inchiostro in modo più veloce se si scrive in modo eccessivo o se si utilizzano penne a sfera di bassa qualità o con inchiostro di bassa qualità.

**Punteggio: 2**

- **Prompt - Item 4:**
  Qual è la differenza tra un pianista e un tenore?
  **Risposta ChatGPT:**
  La differenza tra un pianista e un tenore è che il pianista suona il pianoforte, mentre il tenore è un cantante che canta nella gamma di voci maschili più alta, con una tessitura che va da circa la nota di sol al do acuto. Il tenore è solitamente associato a ruoli di protagonista in opere liriche, mentre il pianista può suonare una varietà di generi musicali.
  **Punteggio: 2**
- **Prompt - Item 5:**
  Qual è la differenza tra uno specchio e un vetro?
  **Risposta ChatGPT:**La differenza principale tra uno specchio e un vetro è che lo specchio riflette la luce, mentre il vetro la lascia passare. Lo specchio è costituito da una superficie riflettente, solitamente di argento, oro o alluminio, mentre il vetro è un materiale trasparente e opaco.

Un'altra differenza tra lo specchio e il vetro è la loro trasparenza. Lo specchio è opaco, quindi non è possibile vedere attraverso di esso, mentre il vetro è trasparente, quindi è possibile vedere attraverso di esso.

Inoltre, lo specchio è solitamente più leggero e fragile del vetro, poiché è costituito da una superficie riflettente su un supporto leggero, mentre il vetro è solitamente più pesante e robusto poiché è costituito da un materiale solido e trasparente.
**Punteggio: 2**

- **Prompt - Item 6:**
  Qual è la differenza tra un pranzo e una cena?
  **Risposta ChatGPT:**
  La differenza principale tra un pranzo e una cena è l'orario in cui vengono consumati. Il pranzo è solitamente il pasto principale consumato durante il giorno, mentre la cena è solitamente il pasto principale consumato la sera. Tuttavia, queste definizioni possono variare in base alla cultura e alle abitudini alimentari di ogni individuo.

Inoltre, il contenuto di un pranzo e di una cena può variare in base alle preferenze personali e alle tradizioni culturali. Ad esempio, in alcune culture il pranzo può essere un pasto leggero, mentre la cena può essere più sostanziosa, mentre in altre culture il pranzo può essere il pasto più grande della giornata. Inoltre, alcune culture potrebbero includere il brunch, che è un pasto consumato la mattina presto o a metà mattina, come una combinazione di colazione e pranzo.
**Punteggio: 2**

- **Prompt - Item 7:**
  Qual è la differenza tra un dentifricio e uno shampoo?

**Risposta ChatGPT:**
La differenza principale tra un dentifricio e uno shampoo è che il dentifricio viene utilizzato per pulire i denti e il cavo orale, mentre lo shampoo viene utilizzato per pulire e idratare i capelli.

Il dentifricio contiene solitamente sostanze chimiche come il fluoro, che aiuta a prevenire la carie, e il percarbonato di sodio, che aiuta a rimuovere la placca e il tartaro dai denti. Lo shampoo contiene solitamente tensioattivi, che aiutano a rimuovere lo sporco e l'oleosità dai capelli, e agenti idratanti, che aiutano a mantenere i capelli morbidi e idratati.

Inoltre, il dentifricio viene utilizzato internamente, mentre lo shampoo viene utilizzato esternamente sui capelli e sulla cute. Il dentifricio viene applicato sullo spazzolino da denti e utilizzato per detergere i denti, mentre lo shampoo viene applicato sui capelli bagnati e risciacquato via dopo averlo massaggiato sulla cute.

**Punteggio: 2**

**Totale subtest Differenze: 14/14**

## **Espressioni idiomatiche**

- **Prompt - Item 1:**
  Cosa vuol dire “ad ogni morte di papa”?
  **Risposta ChatGPT:**
  "Ad ogni morte di papa" è un modo di dire che significa "molto raramente" o "molto di rado". Il papa è il capo della Chiesa cattolica, e la sua elezione e il suo mandato sono considerati per molti aspetti come eventi straordinari e unici. Pertanto, la frase "ad ogni morte di papa" viene utilizzata per indicare che qualcosa accade molto raramente o molto di rado, simile all'espressione inglese "once in a blue moon".

Ad esempio, si potrebbe dire "Vado al cinema ad ogni morte di papa", per indicare che si va al cinema molto raramente. Oppure si potrebbe dire "Mangio carne di maiale ad ogni morte di papa", per indicare che si mangia carne di maiale molto di rado.
**Punteggio: 2**

- **Prompt - Item 2:**
  Cosa vuol dire “nascere con la camicia”?
  **Risposta ChatGPT:**"Nascere con la camicia" è un modo di dire che significa "avere successo o fortuna fin dalla nascita". La frase "nascere con la camicia" si riferisce all'idea che alcune persone hanno la fortuna di essere nate in una famiglia ricca o di alta classe sociale, o di avere talenti naturali o opportunità che le rendono particolarmente fortunate fin dalla nascita.

Ad esempio, si potrebbe dire "Lui è nato con la camicia, ha sempre avuto tutto quello che voleva" per indicare che qualcuno ha sempre avuto successo e fortuna fin dalla nascita. Oppure si potrebbe dire "Lei è nata con la camicia, ha un talento naturale per la musica" per indicare che qualcuno ha avuto successo fin dalla nascita a causa di un talento naturale.
**Punteggio: 2**

- **Prompt - Item 3:**
  Cosa vuol dire “essere al verde”?
  **Risposta ChatGPT:**

"Essere al verde" è un modo di dire che significa "essere senza soldi o avere difficoltà finanziarie". La frase "essere al verde" si riferisce all'idea che la mancanza di denaro o le difficoltà finanziarie sono simili a una situazione di povertà o di indigenza, come se si fosse "al verde" o senza risorse.

Ad esempio, si potrebbe dire "Sono al verde, non ho soldi per pagare l'affitto" per indicare che si ha difficoltà finanziarie e non si ha il denaro necessario per pagare le spese. Oppure si potrebbe dire "Sono al verde, non ho soldi per comprare il biglietto del treno" per indicare che si è senza soldi e non si ha il denaro necessario per fare un acquisto specifico.

**Punteggio: 2**

- **Prompt - Item 4:**
  Cosa vuol dire “gettare la spugna”?
  **Risposta ChatGPT:**
  "Gettare la spugna" è un modo di dire che significa "rinunciare" o "arrendersi". La frase "gettare la spugna" si riferisce all'idea di abbandonare un'attività o un impegno, simile a come si getterebbe un asciugamano o una spugna usata in segno di resa o di sconfitta.

Ad esempio, si potrebbe dire "Ho deciso di gettare la spugna, non riesco a finire questo puzzle" per indicare che si rinuncia a completare un'attività. Oppure si potrebbe dire "Ha gettato la spugna, non riusciva a sopportare il dolore" per indicare che qualcuno ha rinunciato a continuare a sopportare una situazione difficile.
**Punteggio: 2**

- **Prompt - Item 5:**
  Cosa vuol dire “avere il pollice verde”?
  **Risposta ChatGPT:**
  "Avere il pollice verde" è un modo di dire che significa "avere un talento naturale per coltivare piante e fiori". La frase "avere il pollice verde" si riferisce all'idea che alcune persone hanno un'innata capacità di far crescere e prosperare le piante, come se avessero il pollice magico o il tocco di Midas per la coltivazione.

Ad esempio, si potrebbe dire "Ha il pollice verde, riesce a far crescere qualsiasi cosa" per indicare che qualcuno ha un talento naturale per coltivare piante. Oppure si potrebbe dire "Io non ho il pollice verde, non riesco mai a far crescere nulla" per indicare che si è meno bravi a coltivare piante.
**Punteggio: 2**

- **Prompt - Item 6:**
  Cosa vuol dire “sputare il rospo”?
  **Risposta ChatGPT:**"Sputare il rospo" è un modo di dire che significa "dire la verità" o "confessare qualcosa di difficile o imbarazzante". La frase "sputare il rospo" si riferisce all'idea di espellere qualcosa di spiacevole o indesiderato, come se si sputasse fuori un rospo dalla bocca.

Ad esempio, si potrebbe dire "Ho deciso di sputare il rospo, ho mentito sulla mia età" per indicare che si è decisi a confessare una bugia o una verità difficile. Oppure si potrebbe dire "Non riesco a sputare il rospo, ho paura delle conseguenze" per indicare che si ha difficoltà a confessare qualcosa di imbarazzante o scomodo.
**Punteggio: 2**

- **Prompt - Item 7:**
  Cosa vuol dire “cadere dalla padella alla brace”?
  **Risposta ChatGPT:**"Cadere dalla padella alla brace" è un modo di dire che significa "passare da una situazione difficile a una ancora peggiore". La frase "cadere dalla padella alla brace" si riferisce all'idea di passare da una situazione scomoda o problematica a una ancora più difficile o dolorosa, come se si passasse da una padella calda ad una brace bollente.

Ad esempio, si potrebbe dire "Sono caduto dalla padella alla brace, ho perso il lavoro e ora non ho soldi per pagare l'affitto" per indicare che si è passati da una situazione difficile a una ancora peggiore. Oppure si potrebbe dire "Stavo già male, poi sono caduto dalla padella alla brace, mi hanno diagnosticato una malattia grave" per indicare che si è passati da una situazione già problematica a una ancora più difficile.
**Punteggio: 2**

**Totale subtest Espressioni idiomatiche: 14/14**

## **Relazioni di familiarità**

- **Prompt - Item 1:**
  Franco e Maria sono fratello e sorella. Maria è madre di Luca e Franco è padre di Marco. Che relazione di parentela c'è tra Luca e Marco?
  **Risposta ChatGPT:**
  Luca e Marco sono cugini. Maria e Franco sono fratello e sorella, quindi Luca e Marco hanno lo stesso nonno e la stessa nonna. Inoltre, Franco è il padre di Marco, quindi Luca e Marco hanno anche lo stesso zio paterno. Pertanto, Luca e Marco sono cugini, ovvero hanno una relazione di parentela legata da uno o più gradi di consanguineità.
  **Punteggio: 2**
- **Prompt - Item 2:**
  Mario e Luisa sono i genitori di Matteo. Paolo è il padre di Luisa. Che relazione c'è tra Matteo e Paolo?
  **Risposta ChatGPT:**
  Matteo e Paolo hanno una relazione di parentela di nonno e nipote. Luisa è la madre di Matteo e Paolo è il padre di Luisa, quindi Paolo è il nonno di Matteo. Pertanto, Matteo e Paolo hanno una relazione di nonno e nipote, ovvero hanno una relazione di parentela legata da due gradi di consanguineità.
  **Punteggio: 2**
- **Prompt - Item 3:**
  Nina e Roberto sono i genitori di Carlo. Carlo è sposato con Simona. Che relazione c'è tra Nina e Simona?
  **Risposta ChatGPT:**
  Nina e Simona hanno una relazione di suocera e nuora. Nina è la madre di Carlo, quindi è la suocera di Simona. Simona è la moglie di Carlo, quindi è la nuora di Nina. Pertanto, Nina e Simona hanno una relazione di suocera e nuora, ovvero hanno una relazione di parentela legata dal matrimonio di Carlo e Simona.
  **Punteggio: 2**
- **Prompt - Item 4:**
  Ettore è il marito di Anna. I genitori di Anna sono Giuseppe e Iole. Che relazione c'è tra Ettore e Iole?
  **Risposta ChatGPT:**
  Ettore e Iole hanno una relazione di genero e suocera. Ettore è il marito di Anna, quindi è il genero di Iole. Iole è la madre di Anna, quindi è la suocera di Ettore. Pertanto, Ettore e Iole hanno una relazione di genero e suocera, ovvero hanno una relazione di parentela legata dal matrimonio di Anna e Ettore.
  **Punteggio: 2**
- **Prompt - Item 5:**
  Loredana è sposata con Vittorio. Vittorio ha due fratelli: Daniele e Simone. Che relazione c'è tra Loredana e Simone?
  **Risposta ChatGPT:**
  Loredana e Simone hanno una relazione di cognata e cognato. Loredana è sposata con Vittorio, quindi è la cognata di Simone. Simone è il fratello di Vittorio, quindi è il cognato di Loredana. Pertanto, Loredana e Simone hanno una relazione di cognata e cognato, ovvero hanno una relazione di parentela legata dal matrimonio di Loredana e Vittorio.
  **Punteggio: 2**
- **Prompt - Item 6:**
  Rocco è il padre di Dino. Dino è il padre di Rosario e Rosario è il padre di Francesco. Che relazione c'è tra Rocco e Francesco?
  **Risposta ChatGPT:**
  Rocco e Francesco hanno una relazione di bisnonno e bisnipote. Rocco è il padre di Dino, quindi è il nonno di Rosario. Rosario è il padre di Francesco, quindi è il nonno di Francesco. Pertanto, Rocco è il bisnonno di Francesco, e Francesco è il bisnipote di Rocco. La relazione tra Rocco e Francesco è quindi di bisnonno e bisnipote, ovvero hanno una relazione di parentela legata da tre gradi di consanguineità.
  **Punteggio: 1**
- **Prompt - Item 7:**
  Paolo è fratello di Michele. Michele è padre di Sara. Che relazione di parentela c'è tra Sara e Paolo?
  **Risposta ChatGPT:**
  Sara e Paolo hanno una relazione di zia e nipote. Paolo è fratello di Michele, quindi è zio di Sara. Sara è figlia di Michele, quindi è nipote di Paolo. Pertanto, Sara e Paolo hanno una relazione di zia e nipote, ovvero hanno una relazione di parentela legata da un grado di consanguineità.
  **Punteggio: 2**

**Totale subtest Relazioni di familiarità: 13/14**

## **Classificazione**

- **Prompt - Item 1:**
  In una sola parola, cosa sono pinza, cacciavite, martello?
  **Risposta ChatGPT:**
  Pinza, cacciavite e martello sono attrezzi.
  **Punteggio: 2**
- **Prompt - Item 2:**
  In una sola parola, cosa sono palla, bambola, birillo?
  **Risposta ChatGPT:**
  Palla, bambola e birillo sono giocattoli.
  **Punteggio: 2**
- **Prompt - Item 3:**
  In una sola parola, cosa sono rossetto, ombretto, cipria?
  **Risposta ChatGPT:**
  Rossetto, ombretto e cipria sono cosmetici.
  **Punteggio: 2**
- **Prompt - Item 4:**
  In una sola parola, cosa sono amore, tristezza, rabbia?
  **Risposta ChatGPT:**
  Amore, tristezza e rabbia sono emozioni.
  **Punteggio: 2**
- **Prompt - Item 5:**In una sola parola, cosa sono armadio, letto, tavolo?
  **Risposta ChatGPT:**
  Armadio, letto e tavolo sono mobili.
  **Punteggio: 2**
- **Prompt - Item 6:**
  In una sola parola, cosa sono Piemonte, Liguria, Lazio?
  **Risposta ChatGPT:**
  Piemonte, Liguria e Lazio sono regioni.
  **Punteggio: 2**
- **Prompt - Item 7:**
  In una sola parola, cosa sono pioggia, neve, grandine?
  **Risposta ChatGPT:**
  Pioggia, neve e grandine sono precipitazioni.
  **Punteggio: 2**

**Totale subtest Classificazione: 14/14**

# **Test Delle Stime Cognitive**

(Della Sala, Mac Pherson et al., 2003)

- **Prompt - Item 1:**
  Quanto pesano un paio di scarpe con il tacco?
  **Risposta ChatGPT:**
  In generale, è probabile che un paio di scarpe con il tacco pesi da qualche decina di grammi a qualche etto. Tieni presente che questa è solo una stima approssimativa e il peso effettivo di un paio di scarpe con il tacco può variare ampiamente a seconda dei fattori specifici come il materiale di costruzione, la dimensione e il design.

**Abs. score: 1**

**Bizz. Score: 0**

- **Prompt - Item 2:**
  Quanto misura l’area di un lenzuolo matrimoniale?

**Risposta ChatGPT:**L'area di un lenzuolo matrimoniale dipende dalle dimensioni del materasso per cui è progettato. In generale, i lenzuoli matrimoniali sono progettati per adattarsi a materassi con una larghezza di circa 135-140 centimetri e una lunghezza di circa 190-200 centimetri. Tieni presente che queste sono solo dimensioni approssimative e che i materassi e i lenzuoli matrimoniali possono variare leggermente in dimensioni. Se vuoi calcolare l'area di un lenzuolo matrimoniale, basta moltiplicare la larghezza per la lunghezza. Ad esempio, se un lenzuolo matrimoniale ha una larghezza di 135 centimetri e una lunghezza di 190 centimetri, l'area sarà di circa 25.650 centimetri quadrati.
**Abs. Score: 0**
**Bizz. Score: 0**

- **Prompt - Item 3:**Quanto è alto un semaforo?
  **Risposta ChatGPT:**
  I semafori hanno diverse altezze a seconda del luogo in cui si trovano e del loro scopo. In generale, tuttavia, è probabile che un semaforo abbia un'altezza compresa tra 3 e 6 metri. I semafori più alti sono solitamente quelli che si trovano in posizioni in cui sono visibili da una maggiore distanza, come ad esempio su un'intersezione di strade a grande traffico o in prossimità di un'autostrada. Al contrario, i semafori più bassi sono solitamente quelli che si trovano in posizioni in cui sono visibili da una minore distanza, come ad esempio in un parco o in una zona residenziale. Tieni presente che queste sono solo stime approssimative e l'altezza effettiva di un semaforo può variare ampiamente a seconda del luogo in cui si trova e del suo scopo.
  **Abs. Score: 0
  Bizz. Score: 0**
- **Prompt - Item 4:**Quanto pesa una maglietta di cotone da uomo?
  **Risposta ChatGPT:**
  Il peso di una maglietta di cotone da uomo dipende da molti fattori, come la dimensione, il tessuto e il design. In generale, tuttavia, è probabile che una maglietta di cotone da uomo pesi da qualche etto a qualche chilo, a seconda delle dimensioni e del tessuto. Le magliette di cotone più pesanti sono solitamente quelle realizzate con tessuti più spessi o con design particolari che richiedono l'utilizzo di più materiale. Al contrario, le magliette di cotone più leggere sono solitamente quelle realizzate con tessuti più sottili o con design semplici che richiedono l'utilizzo di meno materiale. Tieni presente che queste sono solo stime approssimative e il peso effettivo di una maglietta di cotone da uomo può variare ampiamente a seconda delle specifiche del tessuto e del design.
  **Abs. Score: 2
  Bizz. Score: 1**
- **Prompt - Item 5:**Quanto pesa un cavallo?
  **Risposta ChatGPT:**
  Il peso di un cavallo dipende dalla sua razza, dalla sua altezza e dal suo sesso. In generale, tuttavia, è probabile che un cavallo adulto pesi da qualche centinaio di chilogrammi a oltre una tonnellata, a seconda della sua razza e della sua altezza al garrese. I cavalli più pesanti sono solitamente quelli di razza più grande, come ad esempio i cavalli da tiro o i cavalli da sella da lavoro, mentre i cavalli più leggeri sono solitamente quelli di razza più piccola, come ad esempio i pony. Tieni presente che queste sono solo stime approssimative e il peso effettivo di un cavallo può variare ampiamente a seconda della sua razza, della sua altezza e del suo sesso.
  **Abs. Score: 0
  Bizz. Score: 1**
- **Prompt - Item 6:**Quanto è lunga la spina dorsale di un uomo?
  **Risposta ChatGPT:**
  La spina dorsale di un uomo è composta da circa 33 vertebre, che si estendono dalla base del cranio fino alla pelvi. In generale, è probabile che la spina dorsale di un uomo abbia una lunghezza compresa tra i 60 e i 70 centimetri, a seconda della sua altezza e della sua conformazione fisica. Tieni presente che questa è solo una stima approssimativa e la lunghezza effettiva della spina dorsale di un uomo può variare ampiamente a seconda dei fattori specifici che influiscono sulla sua conformazione fisica.
  **Abs. Score: 2**
  **Bizz. Score: 0**
- **Prompt - Item 7:**Quale è la distanza massima che si può percorrere in Sicilia in Km?
  **Risposta:**
  La Sicilia è la più grande isola del Mediterraneo, con una superficie di circa 25.000 chilometri quadrati. Se vuoi calcolare la distanza massima che si può percorrere in Sicilia in chilometri, dovresti tenere presente che l'isola ha una forma irregolare con molti promontori e insenature. Di conseguenza, la distanza massima che si può percorrere in Sicilia dipende dal percorso che si sceglie di seguire.
  Se vuoi calcolare la distanza massima che si può percorrere in Sicilia seguendo la costa dell'isola, dovresti tenere presente che la lunghezza della costa siciliana è di circa 10.000 chilometri. Tieni presente che questa è solo una stima approssimativa e la distanza effettiva che si può percorrere in Sicilia dipende dal percorso che si sceglie di seguire e dalle condizioni delle strade e dei mezzi di trasporto utilizzati.
  **Abs. Score: 2
  Bizz. Score: 1**
- **Prompt - Item 8:**Dopo che l’acqua bolle quale è il tempo minimo necessario per cuocere un uovo sodo?
  **Risposta ChatGPT:**
  Il tempo necessario per cuocere un uovo sodo dipende da molti fattori, come la dimensione dell'uovo, la temperatura dell'acqua e il grado di durezza desiderato. In generale, tuttavia, è probabile che il tempo minimo necessario per cuocere un uovo sodo sia di circa 6-7 minuti. Per cuocere un uovo sodo, devi prima portare l'acqua a ebollizione e poi immergere l'uovo nell'acqua bollente. Dopo aver immerto l'uovo, devi farlo bollire per il tempo desiderato. Se vuoi un uovo sodo leggermente morbido, dovresti farlo bollire per circa 6 minuti. Se invece vuoi un uovo sodo più duro, dovresti farlo bollire per circa 7-8 minuti. Tieni presente che queste sono solo stime approssimative e il tempo effettivo necessario per cuocere un uovo sodo può variare ampiamente a seconda dei fattori specifici che influiscono sulla cottura dell'uovo.
  **Abs. Score: 0
  Bizz. Score: 0**
- **Prompt - Item 9:**Quanti grammi di pasta occorrono per 4 persone?
  **Risposta ChatGPT:**La quantità di pasta che occorre per 4 persone dipende dal tipo di pasta, dal condimento e dalle preferenze individuali per quanto riguarda la quantità di pasta da consumare. In generale, tuttavia, è probabile che per 4 persone siano necessari circa 200-250 grammi di pasta. Se vuoi essere più preciso, puoi basare la quantità di pasta sulla porzione raccomandata di circa 50 grammi di pasta per persona. Tieni presente che questa è solo una stima approssimativa e la quantità effettiva di pasta necessaria per 4 persone può variare ampiamente a seconda del tipo di pasta, del condimento e delle preferenze individuali per quanto riguarda la quantità di pasta da consumare.
  **Abs. Score: 1
  Bizz. Score: 0**
- **Prompt - Item 10:**Quante settimane ci sono in un anno?
  **Risposta ChatGPT:**Ci sono 52 settimane in un anno. Ciascuna settimana ha 7 giorni, per un totale di 365 giorni all'anno (o 366 giorni nei anni bisestili). Tieni presente che le settimane iniziano il lunedì e finiscono la domenica, quindi ci sono sempre 52 settimane in un anno, indipendentemente dal numero di giorni che compongono l'anno.
  **Abs. Score: 0
  Bizz. Score: 0**
- **Prompt - Item 11:**Quanti cammelli ci sono in Italia?
  **Risposta ChatGPT:**In Italia ci sono pochi cammelli, poiché questi animali sono originari delle regioni aride e desertiche del Medio Oriente e dell'Africa. Tuttavia, alcuni cammelli possono essere trovati in Italia come animali da divertimento o come animali da spettacolo, soprattutto durante le festività natalizie o in alcuni parchi a tema. Non esiste una stima precisa del numero di cammelli presenti in Italia, ma è probabile che siano solo pochi centinaia. Tieni presente che questa è solo una stima approssimativa e il numero effettivo di cammelli presenti in Italia potrebbe variare.
  **Abs. Score: 2
  Bizz. Score: 0**
- **Prompt - Item 12:**Quante persone possono viaggiare su un pullman durante un’ora di punta?
  **Risposta ChatGPT:**
  La capacità massima di un pullman dipende dal modello e dalle dimensioni del veicolo. In generale, tuttavia, è probabile che un pullman possa ospitare da circa 30 a 60 persone, a seconda delle sue dimensioni e delle normative di sicurezza in vigore. Tieni presente che questa è solo una stima approssimativa e la capacità effettiva di un pullman può variare ampiamente a seconda del modello e delle dimensioni del veicolo. Inoltre, durante le ore di punta il numero di persone che può viaggiare su un pullman può essere influenzato dal livello di affollamento del veicolo e dalle normative di distanziamento sociale in vigore per prevenire la diffusione di infezioni.
  **Abs. Score: 1
  Bizz. Score: 0**
- **Prompt - Item 13:**
  Quanti posti a sedere ci sono su un treno di 10 carrozze?
  **Risposta ChatGPT:**
  La capacità di un treno dipende dal modello e dalle dimensioni delle carrozze. In generale, tuttavia, è probabile che un treno di 10 carrozze possa ospitare da circa 200 a 400 passeggeri, a seconda delle sue dimensioni e delle normative di sicurezza in vigore. Tieni presente che questa è solo una stima approssimativa e la capacità effettiva di un treno può variare ampiamente a seconda del modello e delle dimensioni delle carrozze. Inoltre, il numero di posti a sedere disponibili su un treno può essere influenzato dal livello di affollamento del veicolo e dalle normative di distanziamento sociale in vigore per prevenire la diffusione di infezioni.
  **Abs. Score: 1
  Bizz. Score: 0**
- **Prompt - Item 14:**
  Quante ciglia ci sono nella palpebra inferiore?
  **Risposta ChatGPT:**
  La quantità di ciglia presenti sulla palpebra inferiore dipende da molti fattori, come il sesso, l'età e la conformazione fisica. In generale, tuttavia, è probabile che ci siano da circa 80 a 100 ciglia sulla palpebra inferiore di ciascun occhio. Tieni presente che questa è solo una stima approssimativa e la quantità effettiva di ciglia presenti sulla palpebra inferiore può variare ampiamente a seconda dei fattori specifici che influiscono sulla conformazione fisica. Le ciglia sono piccole strutture presenti sulla palpebra inferiore e superiore che proteggono l'occhio dall'ingresso di corpi estranei e dalla luce intensa.
  **Abs. Score: 1
  Bizz. Score: 0**
- **Prompt - Item 15:**
  A che velocità può volare una rondine in Km/h?
  **Risposta ChatGPT:**
  Le rondini sono uccelli molto veloci e possono volare a velocità fino a 80 km/h o anche di più, a seconda della specie e delle condizioni. Tuttavia, queste velocità sono tipicamente raggiunte solo in picchi di accelerazione o durante la caccia. La velocità di volo normale di una rondine è di solito compresa tra i 30 e i 60 km/h. In generale, le rondini sono in grado di spostarsi rapidamente nell'aria grazie alla loro struttura corporea leggera e alla forma aerodinamica delle loro ali.
  **Abs. Score: 0
  Bizz. Score: 0**
- **Prompt - Item 16:**
  Quanti Kg può sollevare un atleta di sollevamento pesi?
  **Risposta ChatGPT:**
  La quantità di peso che un atleta di sollevamento pesi è in grado di sollevare dipende da diversi fattori, come la loro forza, la tecnica, la struttura fisica e la categoria di peso in cui competono. In generale, gli atleti di sollevamento pesi competono in categorie di peso specifiche, con limite di peso massimo che variano a seconda della categoria. Ad esempio, nella categoria maschile "pesi massimi" (che include atleti che pesano oltre 105 kg), i partecipanti possono sollevare quantità di peso estremamente elevate, spesso superiore ai 300 kg. Tuttavia, anche gli atleti di categoria inferiore sono in grado di sollevare quantità di peso significative, con alcuni che riescono a sollevare oltre 200 kg. In generale, gli atleti di sollevamento pesi sono estremamente forti e in grado di sollevare quantità di peso notevoli, anche se la quantità esatta dipende dall'atleta specifico e dalle condizioni in cui si trova.
  **Abs. Score: 0
  Bizz. Score: 0**
- **Prompt - Item 17:**
  Quanto è lunga una carrozza passeggeri di un treno?
  **Risposta ChatGPT:**
  La lunghezza di una carrozza passeggeri di un treno può variare a seconda del tipo di treno e delle specifiche della carrozza. Tuttavia, in generale, le carrozze passeggeri di un treno hanno una lunghezza di circa 20-25 metri. Questa è la lunghezza tipica delle carrozze passeggeri utilizzate nei treni a lunga percorrenza, come quelli utilizzati per il trasporto di passeggeri su lunghe distanze. Le carrozze passeggeri dei treni locali o dei treni metropolitani possono essere leggermente più corte, con una lunghezza di circa 15-20 metri. Tuttavia, anche queste carrozze possono avere una lunghezza leggermente superiore a seconda delle specifiche del treno e delle esigenze del servizio di trasporto.
  **Abs. Score: 0
  Bizz. Score: 0**
- **Prompt - Item 18:**
  Quanti litri d’acqua servono per riempire una vasca da bagno?
  **Risposta ChatGPT:**
  La quantità di acqua che serve per riempire una vasca da bagno dipende dalle dimensioni della vasca e dalla quantità di acqua che si desidera riempire. In generale, per riempire una vasca da bagno di dimensioni standard, che ha una capacità di circa 150 litri, saranno necessari circa 150 litri di acqua. Tuttavia, se si desidera riempire la vasca solo parzialmente, sarà necessaria una quantità di acqua inferiore. Inoltre, se si utilizza una vasca da bagno di dimensioni maggiori o una vasca di forma o dimensione insolita, saranno necessari una quantità di acqua maggiore o minore per riempirla. In ogni caso, è importante considerare che il consumo di acqua può variare a seconda delle abitudini e delle esigenze individuali, quindi la quantità di acqua necessaria per riempire una vasca da bagno può variare significativamente da persona a persona.
  **Abs. Score: 1
  Bizz. Score: 0**
- **Prompt - Item 19:**
  Quanto tempo impiega un uomo giovane per percorrere camminando un Km?
  **Risposta ChatGPT:**
  La velocità di camminata di una persona può variare a seconda di molti fattori, come l'età, la forma fisica, il terreno su cui si cammina e la distanza che si deve percorrere. In generale, però, un uomo giovane in buona forma fisica potrebbe essere in grado di percorrere un chilometro a piedi in circa 15-20 minuti, a una velocità di camminata di circa 4-5 km/h. Tuttavia, questo è solo un valore approssimativo e la velocità di camminata effettiva di una persona può variare notevolmente. Inoltre, è importante considerare che la velocità di camminata può variare a seconda delle condizioni del terreno e delle esigenze individuali. Ad esempio, potrebbe essere necessario camminare più lentamente in salita o su terreni sconnessi, mentre potrebbe essere possibile camminare più velocemente su terreni pianeggianti o in discesa.
  **Abs. Score: 1**
  **Bizz. Score: 0**
- **Prompt - Item 20:**Quanti fiammiferi contiene una scatoletta di fiammiferi?
  **Risposta ChatGPT:**
  La quantità di fiammiferi contenuti in una scatoletta dipende dal tipo di scatoletta e dal produttore. In generale, le scatole di fiammiferi possono contenere da un minimo di circa 10 a un massimo di circa 50 fiammiferi, ma queste cifre possono variare a seconda della dimensione della scatoletta e della quantità di fiammiferi che il produttore sceglie di inserire in essa. È importante leggere l'etichetta o le istruzioni del produttore per conoscere la quantità esatta di fiammiferi contenuti in una scatoletta specifica.
  **Abs. Score: 1
  Bizz. Score: 0**
- **Prompt - Item 21:**Approssimativamente quanti caffé prepara un barman di un autogrill in un’ora di tempo durante l’ora di punta?
  **Risposta ChatGPT:**
  È difficile fare una stima approssimativa di quanti caffè possa preparare un barman di un autogrill in un'ora di tempo durante l'ora di punta, poiché dipende da una serie di fattori come il tipo di macchina da caffè utilizzata, la velocità del barman, il numero di clienti che richiedono il caffè e le eventuali interruzioni o richieste di altri tipi di bevande o alimenti. Tuttavia, tenendo presente questi fattori, è probabile che un barman possa preparare da un minimo di circa 20 a un massimo di circa 100 caffè in un'ora di tempo durante l'ora di punta, anche se questo range può variare in base alle circostanze specifiche. Si tratta comunque solo di una stima approssimativa e potrebbe esserci una grande variabilità nella quantità effettiva di caffè preparati in un'ora di tempo durante l'ora di punta.
  **Abs Score: 1**

**Bizz. Score: 0**

**Totale Abs. Score: 17/41**

**Totale Bizz. Score: 3/21**

# **Comprensione di Metafore**

(Papagno et al. 1995)

- **Prompt - Item 1:**Cosa significa "quello scolaro è un asino"?
  **Risposta ChatGPT:**
  "Quello scolaro è un asino" significa che la persona a cui si fa riferimento è stupida o goffa. Si tratta di un modo di dire che viene utilizzato per indicare che qualcuno ha difficoltà a comprendere o ad apprendere qualcosa, o che commette errori o azioni sbagliate in modo frequente. Tieni presente che questa espressione può essere considerata offensiva o dispregiativa, quindi dovresti evitarne l'uso se non vuoi ferire i sentimenti di qualcuno.

**Attribuzione del punteggio:**Lento ad apprendere, ignorante, caparbio **2** Svogliato, negligente **1
Punteggio: 2**

- **Prompt - Item 2:**Cosa vuol dire "quel treno è una lumaca?"
  **Risposta ChatGPT:**
  "Quel treno è una lumaca" significa che il treno in questione viaggia lentamente, probabilmente a causa di un rallentamento dovuto a problemi tecnici o a condizioni meteorologiche avverse. Si tratta di un modo di dire che viene utilizzato per indicare che qualcosa o qualcuno si muove in modo lento o poco efficiente. Tieni presente che questa espressione è solo un modo di dire e non deve essere presa alla lettera: i treni non sono animali e non possono avere le caratteristiche fisiche delle lumache.
  **Attribuzione del punteggio:**Lento **2** Esempio concreto **1
  Punteggio: 2**
- **Prompt - Item 3:**Cosa vuol dire "le persone in attesa erano sulle spine"?
  **Risposta ChatGPT:**
  "Le persone in attesa erano sulle spine" significa che le persone in questione erano molto nervose o agitate perché dovevano aspettare qualcosa di importante o di incerto. La frase "essere sulle spine" si riferisce al fatto di essere in ansia o in tensione a causa di una situazione stressante o incerta. Tieni presente che questa espressione è solo un modo di dire e non deve essere presa alla lettera: le persone non possono avere le spine fisiche come gli animali.
  **Attribuzione del punteggio:**Ansiose, apprensive, in agitazione, a disagio **2** Impazienti **1
  Punteggio: 2**
- **Prompt - Item 4:**Cosa vuol dire “oggi mi sento un leone”?
  **Risposta ChatGPT:**
  Oggi mi sento un leone" significa che la persona che parla si sente forte, energica e pronta ad affrontare qualunque sfida. Si tratta di un modo di dire che viene utilizzato per indicare uno stato d'animo positivo e di grande sicurezza in se stessi. Tieni presente che questa espressione è solo un modo di dire e non deve essere presa alla lettera: le persone non possono avere le caratteristiche fisiche dei leoni o sentirsi come loro.
  **Attribuzione del punteggio:**Forte, coraggioso **2** Forte o coraggioso **1
  Punteggio: 1**
- **Prompt - Item 5:**Cosa vuol dire “gli affari vanno a gonfie vele”?
  **Risposta ChatGPT:**
  "Gli affari vanno a gonfie vele" significa che le attività commerciali o i progetti a cui si fa riferimento stanno andando molto bene e hanno successo. Si tratta di un modo di dire che viene utilizzato per indicare che qualcosa sta procedendo in modo positivo e soddisfacente. La frase "gonfie vele" si riferisce all'immagine delle vele di una nave che si gonfiano al vento, a indicare che il viaggio è in corso e che si sta andando avanti con forza e determinazione. Tieni presente che questa espressione è solo un modo di dire e non deve essere presa alla lettera: gli affari non hanno vele fisiche che possono gonfiarsi.
  **Attribuzione del punteggio:**Favorevolmente **2** Secondo i propri desideri/aspettative **1
  Punteggio: 2**
- **Prompt - Item 6:**Cosa vuol dire “stanno cercando di gettarmi il fumo negli occhi”?
  **Risposta ChatGPT:**
  "Stanno cercando di gettarmi il fumo negli occhi" significa che qualcuno sta cercando di confondere o ingannare la persona a cui si fa riferimento, facendole credere qualcosa che non è vero o nascondendo informazioni importanti. Si tratta di un modo di dire che viene utilizzato per indicare che qualcuno sta cercando di manipolare o ingannare gli altri per ottenere un vantaggio o per evitare di affrontare delle responsabilità. Tieni presente che questa espressione è solo un modo di dire e non deve essere presa alla lettera: nessuno può gettare fisicamente il fumo negli occhi di qualcun altro.
  **Attribuzione del punteggio:**Illudere, ingannare con false apparenze **2** Confondere, imbrogliare, nascondere **1
  Punteggio: 1**
- **Prompt - Item 7:**Cosa vuol dire “quell’uomo ha il cuore di pietra”?
  **Risposta ChatGPT:**
  "Quell'uomo ha il cuore di pietra" significa che l'uomo in questione è insensibile o privo di emozioni, probabilmente a causa di una personalità fredda o di un passato difficile. Si tratta di un modo di dire che viene utilizzato per indicare che qualcuno è difficile da commuovere o da coinvolgere emotivamente. Tieni presente che questa espressione è solo un modo di dire e non deve essere presa alla lettera: le persone non hanno cuori di pietra fisici.
  **Attribuzione del punteggio:**Insensibile, inflessibile, spietato **2** Cattivo, malvagio **1
  Punteggio: 2**
- **Prompt - Item 8:**Cosa vuol dire “il successo di quel cantante fu un fuoco di paglia”?
  **Risposta ChatGPT:**
  "Il successo di quel cantante fu un fuoco di paglia" significa che il successo ottenuto dal cantante in questione durò poco o non fu duraturo. Si tratta di un modo di dire che viene utilizzato per indicare che qualcosa ha avuto un successo temporaneo o effimero, che non è stato in grado di durare nel tempo. La frase "fuoco di paglia" si riferisce all'immagine di un fuoco che viene acceso rapidamente ma che si spegne altrettanto velocemente, a indicare che qualcosa ha avuto un inizio vivace ma è stato destinato a finire presto. Tieni presente che questa espressione è solo un modo di dire e non deve essere presa alla lettera: il successo di una persona non può essere paragonato a un fuoco fisico.
  **Attribuzione del punteggio:**Fenomeno intenso e di breve durata **2** Fenomeno intenso o di breve durata **1
  Punteggio: 1**
- **Prompt - Item 9:**Cosa vuol dire “quel cavallo è un fulmine”?
  **Risposta ChatGPT:**
  "Quel cavallo è un fulmine" significa che il cavallo in questione è molto veloce o agile. Si tratta di un modo di dire che viene utilizzato per indicare che qualcosa o qualcuno si muove rapidamente o con grande agilità. La frase "fulmine" si riferisce all'immagine di un fulmine, che viaggia nell'aria a velocità estremamente elevata, a indicare che qualcosa o qualcuno è molto veloce. Tieni presente che questa espressione è solo un modo di dire e non deve essere presa alla lettera: i cavalli non possono avere le caratteristiche fisiche dei fulmini.
  **Attribuzione del punteggio:**Veloce, rapido, impetuoso **2** Es. concreto (un trottatore) **1
  Punteggio: 2**
- **Prompt - Item 10:**

Cosa vuol dire “i miei progetti sono andati in fumo”?

**Risposta ChatGPT:**

"I miei progetti sono andati in fumo" significa che i progetti a cui si fa riferimento sono stati interrotti o hanno subito un insuccesso, probabilmente a causa di problemi o imprevisti. Si tratta di un modo di dire che viene utilizzato per indicare che qualcosa è fallito o è stato distrutto. La frase "andare in fumo" si riferisce all'immagine del fumo che si dissipa rapidamente nell'aria, a indicare che qualcosa è svanito o è stato distrutto. Tieni presente che questa espressione è solo un modo di dire e non deve essere presa alla lettera: i progetti non possono andare fisicamente in fumo.
**Attribuzione del punteggio:**Vengono meno, vanno a vuoto, sono falliti, svaniti **2** Vanno male **1**  **Punteggio: 2**

- **Prompt - Item 11:**

Cosa vuol dire “questa cravatta è un pugno nell’occhio”?
**Risposta ChatGPT:**

"Questa cravatta è un pugno nell'occhio" significa che la cravatta in questione è molto vistosa o sgargiante, probabilmente a causa delle sue dimensioni o dei suoi colori. Si tratta di un modo di dire che viene utilizzato per indicare che qualcosa è troppo appariscente o fastidioso per l'occhio. La frase "pugno nell'occhio" si riferisce all'immagine di un pugno che colpisce l'occhio, a indicare che qualcosa è fastidioso o doloroso per l'occhio. Tieni presente che questa espressione è solo un modo di dire e non deve essere presa alla lettera: le cravatte non possono essere paragonate a dei pugni fisici.
**Attribuzione del punteggio:**In contrasto, incompatibile, stonata **2** Brutta, orribile, di cattivo gusto, vistosa **1
Punteggio: 1**

- **Prompt - Item 12:**

Cosa vuol dire “Franco è la colonna della ditta”?
**Risposta ChatGPT:**
"Franco è la colonna della ditta" significa che Franco è una persona molto importante o indispensabile per il buon funzionamento della ditta, probabilmente a causa delle sue competenze o della sua esperienza. Si tratta di un modo di dire che viene utilizzato per indicare che qualcuno è fondamentale o indispensabile per il successo di un'attività o di un progetto. La frase "colonna" si riferisce all'immagine di una colonna portante, che sostiene un edificio e lo rende stabile, a indicare che qualcuno è fondamentale per il buon funzionamento di qualcosa. Tieni presente che questa espressione è solo un modo di dire e non deve essere presa alla lettera: le persone non possono essere paragonate a delle colonne fisiche.
**Attribuzione del punteggio:**Sostegno, caposaldo **2** Indispensabile, essenziale, punto di riferimento **1
Punteggio: 1**

- **Prompt - Item 13:**

Cosa vuol dire “Giovanni è il mio braccio destro”?
**Risposta ChatGPT:**
"Giovanni è il mio braccio destro" significa che Giovanni è una persona di cui ti fidi molto e che ti supporta nei tuoi sforzi o attività. In altre parole, Giovanni è una persona importante per te e svolge un ruolo fondamentale nel tuo lavoro o nelle tue attività personali. Il termine "braccio destro" viene spesso usato per descrivere qualcuno che svolge un ruolo importante nella vita di un'altra persona, in quanto è una persona di cui ci si può fidare e su cui si può contare.
**Attribuzione del punteggio:**Aiuto più valido, aiuto principale, collaborante principale **2** Aiuto, collaboratore, uomo di fiducia **1
Punteggio: 2**

- **Prompt - Item 14:**Cosa vuol dire “la critica porta alle stelle questo libro”?
  **Risposta ChatGPT:**
  "La critica porta alle stelle questo libro" significa che i critici librari o letterari hanno espresso opinioni molto positive su questo libro e lo hanno elogiato in modo entusiastico. In altre parole, la critica ha riservato al libro recensioni molto positive e lo ha considerato un'opera di alto livello. Ciò può indicare che il libro è ben scritto, ha una trama interessante o ha un significato profondo o culturale. La critica può anche essere un indicatore di qualità per i lettori, poiché i critici sono spesso esperti di letteratura e hanno una conoscenza approfondita del mestiere. Se la critica porta alle stelle un libro, ciò può essere un buon segno per chi è interessato a leggerlo.
  **Attribuzione del punteggio:**Celebra, esalta, magnifica, innalza **2** Loda, giudica bene **1
  Punteggio: 2**
- **Prompt - Item 15:**Cosa vuol dire “quel musicista va contro corrente”?
  **Risposta ChatGPT:**
  "Andare contro corrente" significa fare qualcosa che va contro le norme o le aspettative della società o della maggioranza. Nel caso di un musicista, potrebbe significare che fa musica che si discosta dai generi o dalle mode popolari, o che esprime idee o punti di vista che vanno contro le norme culturali o le opinioni dominanti. Potrebbe anche significare che si distingue per la sua originalità o la sua creatività, che non segue le tendenze o i modelli già stabiliti. In questo modo, il musicista va contro corrente rispetto alla maggioranza o alla norma e si distingue come un artista indipendente o innovativo.
  **Attribuzione del punteggio:**Va contro la moda, contro la tendenza comune ai più **2** Originale, innovatore **1
  Punteggio: 2**
- **Prompt - Item 16:**Cosa vuol dire “quel ministro è una banderuola”?
  **Risposta ChatGPT:**
  "Essere una banderuola" significa essere instabile o incostante nelle proprie idee o nei propri comportamenti. Nel caso di un ministro, potrebbe significare che cambia spesso opinione sui temi politici o che si dimostra indeciso o incerto nelle decisioni che prende. Potrebbe anche significare che viene influenzato facilmente da altre persone o da eventi esterni, o che non segue una linea politica coerente. In questo modo, il ministro viene descritto come una banderuola, ovvero come una persona che non è saldamente ancorata a una posizione o a una visione, ma che cambia facilmente direzione in base alle circostanze.
  **Attribuzione del punteggio:**Volubile, che cambia partito o opinione **2** Incoerente **1
  Punteggio: 2**
- **Prompt - Item 17:**Cosa vuol dire “quel soldato è un coniglio”?
  **Risposta ChatGPT:**
  "Essere un coniglio" può avere diverse connotazioni, a seconda del contesto in cui viene usato. In generale, potrebbe significare essere timido o pauroso, o avere un atteggiamento di sottomissione o di arrendevolezza. Nel caso di un soldato, potrebbe significare che non è coraggioso o determinato in combattimento, o che non affronta con determinazione le sfide o i pericoli che incontra. Potrebbe anche significare che non è in grado di resistere alla pressione o allo stress delle situazioni di guerra, o che non segue con fermezza gli ordini o le direttive dei superiori. In questo modo, il soldato viene descritto come un coniglio, ovvero come una persona che non è in grado di affrontare con determinazione o coraggio le difficoltà o i pericoli che incontra.
  **Attribuzione del punteggio:**Vile, pauroso, timido **2** Non amante della guerra **1
  Punteggio: 2**
- **Prompt - Item 18:**Cosa vuol dire “quello scolaro è una cima”?
  **Risposta ChatGPT:**
  "Essere una cima" significa essere molto bravo o dotato in qualcosa. Nel caso di uno scolaro, potrebbe significare che è molto intelligente o che ha ottenuto ottimi risultati a scuola. Potrebbe anche significare che ha una buona memoria o una grande capacità di comprensione, o che è particolarmente interessato o impegnato nello studio. In questo modo, lo scolaro viene descritto come una cima, ovvero come una persona che ha delle eccellenti qualità o che è particolarmente brava in una determinata materia o attività.
  **Attribuzione del punteggio:**Eminente, eccellente, ottimo **2** Bravo, intelligente **1
  Punteggio: 2**
- **Prompt - Item 19:**Cosa vuol dire “L’unica salvezza è prendere il largo”?
  **Risposta ChatGPT:**
  "Prendere il largo" significa allontanarsi da una situazione o da un luogo, spesso per sfuggire a qualcosa o per cercare una soluzione o una via d'uscita. Nel caso in cui si dice che "l'unica salvezza è prendere il largo", si sta suggerendo che la migliore soluzione per affrontare una situazione difficile o per evitare problemi è quella di allontanarsi da essa. Potrebbe significare, ad esempio, che la persona che si trova in quella situazione deve lasciare il posto in cui si trova o che deve cercare altre opportunità o altri luoghi in cui andare. In questo modo, "prendere il largo" viene presentato come l'unica soluzione possibile o come l'unica via d'uscita da una situazione problematica.
  **Attribuzione del punteggio:**Fuggire, allontanarsi, andar via **2** Evitare, scansarsi **1
  Punteggio: 2**
- **Prompt - Item 20:**Cosa vuol dire “la Polizia sa come far cantare i colpevoli”?
  **Risposta ChatGPT:**
  "Far cantare i colpevoli" significa ottenere delle confessioni o delle informazioni importanti da persone che sono ritenute colpevoli di qualche reato o di qualche azione illecita. Nel caso in cui si dice che "la Polizia sa come far cantare i colpevoli", si sta suggerendo che la Polizia ha delle tecniche o dei metodi efficaci per indurre le persone a confessare i loro reati o a rivelare informazioni importanti. Potrebbe significare, ad esempio, che la Polizia utilizza delle tecniche di interrogatorio o di pressione psicologica per ottenere delle confessioni o che fa ricorso a delle minacce o a delle promesse per ottenere delle informazioni. In questo modo, la Polizia viene presentata come un'organizzazione capace di ottenere delle confessioni o delle informazioni importanti da persone che sono ritenute colpevoli di reati o di azioni illecite.
  **Attribuzione del punteggio:**Confessare, cavar di bocca segreti, far parlare **2** Sapere la verità **1
  Punteggio: 2**

**Totale Comprensione di Metafore: 35/40**

# **Comprensione di Espressioni Idiomatiche**

(Papagno et al. 1995)

- **Prompt - Item 1:**Cosa vuol dire “Quella donna si leva il pane di bocca per i figli”?
  **Risposta ChatGPT:**
  "Levare il pane di bocca" significa privarsi di qualcosa di essenziale o di importante per qualcun altro. Nel caso in cui si dice che "quella donna si leva il pane di bocca per i figli", si sta suggerendo che la donna rinuncia a qualcosa di importante per se stessa per garantire il benessere o il sostentamento dei suoi figli. Potrebbe significare, ad esempio, che la donna lavora duramente per guadagnare soldi per la famiglia e che non ha tempo o energie per se stessa, o che rinuncia a delle opportunità o a delle comodità per garantire il benessere dei suoi figli. In questo modo, la donna viene descritta come una persona che è disposta a fare sacrifici per gli altri e a mettere gli interessi dei suoi figli al primo posto.
  **Attribuzione del punteggio:**Privarsi dell'essenziale **2** Privarsi del cibo, rinunciare a qualcosa **1
  Punteggio: 2**
- **Prompt - Item 2:**Cosa vuol dire “davanti a queste difficoltà c'è da mettersi le mani nei capelli”?
  **Risposta ChatGPT:**
  "Mettere le mani nei capelli" è un modo di dire che significa essere molto preoccupati o frustrati di fronte a una situazione difficile o complessa. Nel caso in cui si dice che "davanti a queste difficoltà c'è da mettersi le mani nei capelli", si sta suggerendo che la situazione è molto complessa o problematica e che richiede una grande quantità di preoccupazione o di impegno per essere affrontata. Potrebbe significare, ad esempio, che la situazione è molto stressante o che presenta degli ostacoli o delle difficoltà insormontabili. In questo modo, "mettere le mani nei capelli" viene utilizzato per esprimere la frustrazione o il senso di impotenza di fronte a una situazione difficile o complessa.
  **Attribuzione del punteggio:**Disperarsi **2** Non sapere risolvere i problemi **1
  Punteggio: 2**
- **Prompt - Item 3:**Cosa vuol dire “Antonio scende a rotto di collo dalla cima della montagna”?
  **Risposta ChatGPT:**
  "Scendere a rotto di collo" significa muoversi velocemente e con impeto, spesso senza prendere le precauzioni o le precauzioni necessarie. Nel caso in cui si dice che "Antonio scende a rotto di collo dalla cima della montagna", si sta suggerendo che Antonio si sta muovendo velocemente e senza fare attenzione ai pericoli o agli ostacoli che potrebbero esserci lungo il percorso. Potrebbe significare, ad esempio, che Antonio sta scendendo dalla montagna con fretta o imprudenza, o che sta affrontando una situazione con audacia o temerarietà. In questo modo, "scendere a rotto di collo" viene utilizzato per descrivere un comportamento veloce o impetuoso che potrebbe comportare dei rischi o delle conseguenze negative.
  **Attribuzione del punteggio:**All’impazzata, precipitosamente **2** Da potersi rompere il collo, velocemente **1
  Punteggio: 2**
- **Prompt - Item 4:**Cosa vuol dire “Quale disgrazia mi capita tra capo e collo”?
  **Risposta ChatGPT:**
  "Capi e collo" è un modo di dire che significa "tutto insieme" o "senza motivo". Nel caso in cui si dice "Quale disgrazia mi capita tra capo e collo", si sta esprimendo frustrazione o preoccupazione per un evento negativo o sfortunato che è successo senza un apparente motivo o senza che si fosse fatto nulla per evitarlo. Potrebbe significare, ad esempio, che si è subito una perdita o un danno inaspettato o che si è stati coinvolti in una situazione spiacevole senza volerlo. In questo modo, "tra capo e collo" viene utilizzato per descrivere un evento negativo o sfortunato che è successo senza un apparente motivo o senza che si fosse fatto nulla per evitarlo.
  **Attribuzione del punteggio:**All’improvviso, inaspettata **2** Come una cosa che ti arriva in testa **1
  Punteggio: 2**
- **Prompt - Item 5:**Cosa vuol dire “il direttore è una persona alla mano”?
  **Risposta ChatGPT:**
  "Essere alla mano" significa essere facile da trattare o da avvicinare, o essere disponibile e pronto ad aiutare gli altri. Nel caso in cui si dice che "il direttore è una persona alla mano", si sta suggerendo che il direttore è una persona che è facile da avvicinare o con cui è facile parlare, o che è disponibile e pronta ad aiutare gli altri. Potrebbe significare, ad esempio, che il direttore è una persona che ascolta le richieste o le preoccupazioni degli altri e che cerca di trovare delle soluzioni o di fornire un supporto. In questo modo, "essere alla mano" viene utilizzato per descrivere una persona che è facile da trattare o da avvicinare e che è disponibile e pronta ad aiutare gli altri.
  **Attribuzione del punteggio:**Affabile, cortese, cordiale **2** Che non incute soggezione, semplice **1
  Punteggio: 2**
- **Prompt - Item 6:**Cosa vuol dire “Giorgio ha le mani bucate”?
  **Risposta ChatGPT:**
  "Avere le mani bucate" significa essere molto dispendioso o avere molti debiti. Nel caso in cui si dice che "Giorgio ha le mani bucate", si sta suggerendo che Giorgio ha un alto livello di spese o che ha molti debiti da pagare. Potrebbe significare, ad esempio, che Giorgio ha difficoltà a gestire il proprio denaro o che spende molto più di quanto guadagna. In questo modo, "avere le mani bucate" viene utilizzato per descrivere una persona che ha difficoltà a gestire il proprio denaro o che ha molti debiti da pagare.
  **Attribuzione del punteggio:**Scialacquatore, prodigo, sperperatore **2** Che spende troppo **1
  Punteggio: 1**
- **Prompt - Item 7:**Cosa vuol dire “I ladri fanno man bassa nel negozio”?
  **Risposta ChatGPT:**
  "Fare man bassa" significa prendere tutto quello che si può, spesso in modo disordinato o senza rispetto per le proprietà degli altri. Nel caso in cui si dice che "i ladri fanno man bassa nel negozio", si sta suggerendo che i ladri stanno prendendo tutto quello che possono dal negozio, probabilmente in modo disordinato o senza rispetto per le proprietà del negozio o del proprietario. Potrebbe significare, ad esempio, che i ladri stanno rubando molti oggetti o denaro dal negozio in modo indiscriminato o che stanno causando danni o disordine durante il furto. In questo modo, "fare man bassa" viene utilizzato per descrivere un comportamento disordinato o irrispettoso in cui si prende tutto quello che si può senza rispetto per le proprietà degli altri.
  **Attribuzione del punteggio:**Rubare tutto, devastare **2** Rubare **1
  Punteggio: 1**
- **Prompt - Item 8:**Cosa vuol dire “Quel paese è fuori mano”?
  **Risposta ChatGPT:**
  "Essere fuori mano" significa essere difficile da raggiungere o da raggiungere facilmente. Nel caso in cui si dice che "quel paese è fuori mano", si sta suggerendo che il paese è difficile da raggiungere o che non è facilmente accessibile. Potrebbe significare, ad esempio, che il paese si trova in una zona remota o che è difficile da raggiungere con i mezzi di trasporto pubblici. In questo modo, "essere fuori mano" viene utilizzato per descrivere un luogo che è difficile da raggiungere o che non è facilmente accessibile.
  **Attribuzione del punteggio:**Lontano, scomodo, difficile da raggiungere **2** Non collegato con mezzi, giù di strada **1
  Punteggio: 2**
- **Prompt - Item 9:**Cosa vuol dire “Prima di credere devi toccare con mano”?
  **Risposta ChatGPT:**
  “Toccare con mano" significa fare un'esperienza diretta o personale di qualcosa, spesso per avere la certezza o la conferma di qualcosa. Nel caso in cui si dice "Prima di credere devi toccare con mano", si sta suggerendo che è necessario fare un'esperienza diretta o personale di qualcosa per poter avere la certezza o la conferma di ciò che si sta dicendo o di ciò che si sospetta. Potrebbe significare, ad esempio, che è necessario vedere o toccare qualcosa di persona per poterne avere la certezza o che è necessario fare un'esperienza diretta di qualcosa per poterne comprendere appieno il significato o il valore. In questo modo, "toccare con mano" viene utilizzato per descrivere l'importanza di fare un'esperienza diretta o personale di qualcosa per poterne avere la certezza o la conferma.
  **Attribuzione del punteggio:**Accertarsi, sincerarsi **2** Vedere com’è la faccenda, voler sapere **1
  Punteggio: 2**
- **Prompt - Item 10:**Cosa vuol dire “la nuova automobile costa un occhio della testa”?
  **Risposta ChatGPT:**
  "Costare un occhio della testa" è un modo di dire che significa essere molto costoso. Nel caso in cui si dice che "la nuova automobile costa un occhio della testa", si sta suggerendo che la nuova automobile ha un prezzo molto alto. Potrebbe significare, ad esempio, che la nuova automobile è un lusso o che ha un prezzo che rappresenta una grande spesa per la persona che vuole acquistarla. In questo modo, "costare un occhio della testa" viene utilizzato per descrivere un oggetto o un servizio che ha un prezzo molto alto.
  **Attribuzione del punteggio:**Moltissimo, cifre esorbitanti **2** Essere cara **1
  Punteggio: 2**
- **Prompt - Item 11:**Cosa vuol “meglio evitare di dare nell’occhio”?
  **Risposta ChatGPT:**
  "Dare nell'occhio" significa attirare l'attenzione o l'interesse delle altre persone in modo negativo o inappropriato. Nel caso in cui si dice "meglio evitare di dare nell'occhio", si sta suggerendo che è meglio evitare di attirare l'attenzione o l'interesse delle altre persone in modo negativo o inappropriato. Potrebbe significare, ad esempio, che è meglio evitare di fare o di dire cose che potrebbero mettere in cattiva luce o che potrebbero essere considerate inappropriate. In questo modo, "dare nell'occhio" viene utilizzato per descrivere un comportamento o un'azione che attira l'attenzione o l'interesse delle altre persone in modo negativo o inappropriato.
  **Attribuzione del punteggio:**Farsi notare, esporsi, mettersi in vista **2** Farsi vedere **1
  Punteggio: 2**
- **Prompt - Item 12:**Cosa vuol dire “Le contrarietà mi fanno saltare la mosca al naso”?
  **Risposta ChatGPT:**
  "Saltare la mosca al naso" è un modo di dire che significa essere molto irritati o esasperati. Nel caso in cui si dice "Le contrarietà mi fanno saltare la mosca al naso", si sta suggerendo che le contrarietà o gli ostacoli che si incontrano causano una forte irritazione o esasperazione. Potrebbe significare, ad esempio, che le difficoltà o gli imprevisti che si incontrano sono molto stressanti o che causano una grande frustrazione. In questo modo, "saltare la mosca al naso" viene utilizzato per descrivere una forte irritazione o esasperazione causata da difficoltà o imprevisti.
  **Attribuzione del punteggio:**Arrabbiarsi, seccarsi, inalberarsi **2** Infastidirsi, arrabbiarsi per nulla **1
  Punteggio: 2**
- **Prompt - Item 13:**Cosa vuol dire “rischi di rimanere con un pugno di mosche”?
  **Risposta ChatGPT:**
  "Rimanere con un pugno di mosche" è un modo di dire che significa ottenere pochi o nessun risultato o beneficio da un'azione o da uno sforzo. Nel caso in cui si dice "rischi di rimanere con un pugno di mosche", si sta suggerendo che c'è il rischio di ottenere pochi o nessun risultato o beneficio da un'azione o da uno sforzo. Potrebbe significare, ad esempio, che c'è il rischio di non ottenere ciò che si è sperato o che c'è il rischio di non ottenere alcun vantaggio o guadagno da ciò che si sta facendo. In questo modo, "rimanere con un pugno di mosche" viene utilizzato per descrivere il rischio di ottenere pochi o nessun risultato o beneficio da un'azione o da uno sforzo.
  **Attribuzione del punteggio:**A mani vuote, deluso, senza risultati **2** Non afferrare ciò che si desidera, non avere successo **1
  Punteggio: 2**
- **Prompt - Item 14:**Cosa vuol dire “mi hai messo la pulce nell’orecchio”?
  **Risposta ChatGPT:**
  "Metterti la pulce nell'orecchio" significa suggerire o suscitare un'idea o un dubbio che resta in sospeso. Nel caso in cui si dice "mi hai messo la pulce nell'orecchio", si sta suggerendo che qualcuno ha suggerito o suscitato un'idea o un dubbio che resta in sospeso nella mente di chi parla. Potrebbe significare, ad esempio, che qualcuno ha sollevato una questione o un problema che richiede ulteriori riflessioni o che ha suscitato un interesse o una curiosità per qualcosa. In questo modo, "metterti la pulce nell'orecchio" viene utilizzato per descrivere il fatto di suggerire o suscitare un'idea o un dubbio che resta in sospeso.
  **Attribuzione del punteggio:**Insinuare il dubbio, mettere in perplessità **2** Dubitare, non saper decidere, mettere sull’avviso **1
  Punteggio: 2**
- **Prompt - Item 15:**Cosa vuol dire “la spia deve svuotare il sacco”?
  **Risposta ChatGPT:**
  "Svuotare il sacco" è un modo di dire che significa confessare o rivelare tutto ciò che si sa. Nel caso in cui si dice "la spia deve svuotare il sacco", si sta suggerendo che la spia deve confessare o rivelare tutto ciò che sa. Potrebbe significare, ad esempio, che la spia deve fornire informazioni o segreti che ha raccolto o che deve ammettere il proprio ruolo o le proprie azioni. In questo modo, "svuotare il sacco" viene utilizzato per descrivere il fatto di confessare o rivelare tutto ciò che si sa.
  **Attribuzione del punteggio:**Confessare, parlare, rivelare quanto visto **2** Essere sincera **1
  Punteggio: 2**
- **Prompt - Item 16:**Cosa vuol dire “Franco torna a casa con le pive nel sacco”?
  **Risposta ChatGPT:**
  "Tornare a casa con le pive nel sacco" è un modo di dire che significa tornare a casa senza successo o senza aver ottenuto ciò che si era sperato. Nel caso in cui si dice "Franco torna a casa con le pive nel sacco", si sta suggerendo che Franco è tornato a casa senza successo o senza aver ottenuto ciò che si era sperato. Potrebbe significare, ad esempio, che Franco è tornato a casa senza aver raggiunto l'obiettivo che si era prefissato o che è tornato a casa deluso o sconfitto. In questo modo, "tornare a casa con le pive nel sacco" viene utilizzato per descrivere il ritorno a casa senza successo o senza aver ottenuto ciò che si era sperato.
  **Attribuzione del punteggio:**A mani vuote, deluso, scornato, avvilito **2** Senza risultati positivi **1
  Punteggio: 1**
- **Prompt - Item 17:**Cosa vuol dire “A teatro oggi ci sono quattro gatti”?
  **Risposta ChatGPT:**
  "Ci sono quattro gatti" è un modo di dire che significa che c'è poca gente o che c'è un'affluenza scarsa. Nel caso in cui si dice "A teatro oggi ci sono quattro gatti", si sta suggerendo che oggi al teatro c'è poca gente o che c'è un'affluenza scarsa. Potrebbe significare, ad esempio, che ci sono pochi spettatori o che il teatro è poco frequentato. In questo modo, "ci sono quattro gatti" viene utilizzato per descrivere una scarsa affluenza o una piccola quantità di gente.
  **Attribuzione del punteggio:**Pochissime persone **2** Quattro persone, vuoto **1
  Punteggio: 2**
- **Prompt - Item 18:**Cosa vuol dire “ti do carta bianca per questo progetto”?
  **Risposta ChatGPT:**
  "Dare carta bianca" significa dare libertà o autorità totale a qualcuno per fare qualcosa. Nel caso in cui si dice "ti do carta bianca per questo progetto", si sta suggerendo che si sta dando libertà o autorità totale a qualcuno per fare qualcosa relativo a un progetto specifico. Potrebbe significare, ad esempio, che si sta dando a qualcuno la possibilità di agire senza restrizioni o di prendere decisioni in modo autonomo. In questo modo, "dare carta bianca" viene utilizzato per descrivere il fatto di dare libertà o autorità totale a qualcuno per fare qualcosa.
  **Attribuzione del punteggio:**Dar pieni poteri, libertà di azione **2** Delegare, lasciar fare quello che si vuole **1
  Punteggio: 2**
- **Prompt - Item 19:**Cosa vuol dire “Giorgio ha la coda di paglia”?
  **Risposta ChatGPT:**
  "Avere la coda di paglia" è un modo di dire che significa essere facilmente scoperto o svelato. Nel caso in cui si dice "Giorgio ha la coda di paglia", si sta suggerendo che Giorgio è facilmente scoperto o svelato. Potrebbe significare, ad esempio, che Giorgio ha difficoltà a nascondere o a mentire o che è facilmente scoperto quando commette un'azione o un reato. In questo modo, "avere la coda di paglia" viene utilizzato per descrivere il fatto di essere facilmente scoperto o svelato.
  **Attribuzione del punteggio:**Essere in difetto, sospettoso **2** Sentirsi colpevole **1
  Punteggio: 0**
- **Prompt - Item 20:**Cosa vuol dire “Bisogna andare con i piedi di piombo”?
  **Risposta ChatGPT:**
  "Andare con i piedi di piombo" è un modo di dire che significa essere cauti o attenti a ciò che si fa. Nel caso in cui si dice "Bisogna andare con i piedi di piombo", si sta suggerendo che è necessario essere cauti o attenti a ciò che si fa. Potrebbe significare, ad esempio, che è necessario agire con prudenza o con circospezione o che è importante valutare attentamente le conseguenze delle proprie azioni. In questo modo, "andare con i piedi di piombo" viene utilizzato per descrivere il fatto di essere cauti o attenti a ciò che si fa.
  **Attribuzione del punteggio:**Con circospezione, prudenza, cautela **2** Andare adagio, pensare bene **1
  Punteggio: 2**

**Totale Comprensione di Espressioni Idiomatiche: 36/40**

**Referenze Anaforiche**

(Sartori et al., 2022; in press)

Note: For a visualization purpose, in bold the correct response. However, not any type of formatting cue was given to ChatGPT in the testing phase.

- **Prompt - Item 1:**
  Paolo ha provato a chiamare Giorgio al telefono, ma non era disponibile.
  Domanda: Chi ‘non era disponibile’?

A. Paolo

**B. Giorgio**

**Risposta ChatGPT:**
La risposta corretta è B. Giorgio non era disponibile.

**Punteggio: 1**

- **Prompt - Item 2:**
  Nella tempesta, l’albero è caduto e ha sfondato il tetto della mia casa. Ora, devo rimuoverlo.
  Domanda: Cosa ‘deve essere rimosso’?

**A. L’albero**

B. Il tetto

**Risposta ChatGPT:** La risposta corretta è A. L'albero deve essere rimosso.
**Punteggio: 1**

- **Prompt - Item 3:**Tutti hanno apprezzato i biscotti alle mandorle mentre solo a poche persone sono piaciuti i biscotti alla cannella. La prossima volta dovremmo farne di meno.
  Domanda: ‘Quale tipo di biscotti dovremmo fare di meno la prossima volta’?

A. I biscotti alle mandorle

**B. I biscotti alla cannella**

**Risposta ChatGPT:**
La risposta corretta è B. Dovremmo fare meno biscotti alla cannella la prossima volta.

**Punteggio: 1**

- **Prompt - Item 4:**Tommaso ha dato a Raffaele un passaggio a scuola così non avrebbe dovuto camminare da solo.
  Domanda: Chi ‘non avrebbe dovuto camminare da solo’?

A. Tommaso

**B. Raffaele**

**Risposta ChatGPT:**
La risposta corretta è B. Raffaele non avrebbe dovuto camminare da solo.

**Punteggio: 1**

- **Prompt - Item 5:**Anna ha chiesto a Maria a che ora chiude la biblioteca, perché lei lo ha dimenticato.
  Domanda: Chi ‘lo ha dimenticato’?

A. Maria

**B. Anna**

**Risposta ChatGPT:**
La risposta corretta è B. Anna ha dimenticato a che ora chiude la biblioteca.
**Punteggio: 1**

- **Prompt - Item 6:**Il sacco di patate era stato posizionato sopra il sacco di farina, quindi doveva essere spostato per primo.
  Domanda: Cosa ‘doveva essere spostato per primo’?

**A. Il sacco di patate**

B. Il sacco di farina

**Risposta ChatGPT:**
La risposta corretta è A. Il sacco di patate doveva essere spostato per primo.
**Punteggio: 1**

- **Prompt - Item 7:**I giornalisti hanno intervistato i protagonisti del nuovo film. Sono stati molto insistenti, quindi l'intervista è durata a lungo.

Domanda: Chi ‘è stato molto insistente’?

**A. I giornalisti**

B. I protagonisti

**Risposta ChatGPT:**
La risposta corretta è A. I giornalisti sono stati molto insistenti.
**Punteggio: 1**

- **Prompt - Item 8:**Tutti hanno apprezzato i biscotti alle mandorle mentre solo a poche persone sono piaciuti i biscotti alla cannella. La prossima volta dovremmo farne di più.

Domanda: ‘Quale tipo di biscotti dovremmo fare di più la prossima volta’?

**A. I biscotti alle mandorle**

B. I biscotti alla cannella

**Risposta ChatGPT:**
La risposta corretta è A. Dovremmo fare più biscotti alle mandorle la prossima volta.
**Punteggio: 1**

- **Prompt - Item 9:**I giornalisti hanno intervistato i protagonisti del nuovo film. Sono stati molto collaborativi, quindi l'intervista è durata a lungo.
  Domanda: Chi ‘è stato molto collaborativo’?

A. I giornalisti

**B. I protagonisti**

**Risposta ChatGPT:**
La risposta corretta è B. I protagonisti sono stati molto collaborativi.
**Punteggio: 1**

- **Prompt - Item 10:**L’uomo non poteva sollevare suo figlio perché era molto debole.
  Domanda: Chi ‘era debole’?

**A. L’uomo**

B. Il figlio

**Risposta ChatGPT:**
La risposta corretta è A. L'uomo era debole.
**Punteggio: 1**

- **Prompt - Item 11:**Il sacco di patate era stato posizionato sotto il sacco di farina, quindi doveva essere spostato per primo.

Domanda: Cosa ‘doveva essere spostato per primo’?

A. Il sacco di patate

**B. Il sacco di farina**

**Risposta ChatGPT:**
La risposta corretta è A. Il sacco di patate doveva essere spostato per primo.
**Punteggio: 0**

- **Prompt - Item 12:**Pietro invidia Martino perché ha molto successo.

Domanda: Chi ‘ha molto successo’?

A. Pietro

**B. Martino**

**Risposta ChatGPT:**
La risposta corretta è B. Martino ha molto successo.
**Punteggio: 1**

- **Prompt - Item 13:**L’uomo non poteva sollevare suo figlio perché era molto pesante.

Domanda: Chi ‘era pesante’?

A. L’uomo

**B. Il figlio**

**Risposta ChatGPT:**
La risposta corretta è B. Il figlio era molto pesante e quindi l'uomo non poteva sollevarlo.
**Punteggio: 1**

- **Prompt - Item 14:**La poltrona non può passare attraverso la porta perché è troppo larga.

Domanda: Cos'è ‘troppo larga’?

**A. La poltrona**

B. La porta.

**Risposta ChatGPT:**
La risposta corretta è B. La porta è troppo larga.
**Punteggio: 0**

- **Prompt - Item 15:**Paolo ha provato a chiamare Giorgio al telefono, ma non ci è riuscito.

Domanda: Chi ‘non ci è riuscito’?

**A. Paolo**

B. Giorgio

**Risposta ChatGPT:**
La risposta corretta è A. Paolo non ci è riuscito.
**Punteggio: 1**

- **Prompt - Item 16:**Nella tempesta, l’albero è caduto e ha sfondato il tetto della mia casa. Ora, devo ripararlo.

Domanda: Cosa ‘deve essere riparato’?

A. L’albero

**B. Il tetto**

**Risposta ChatGPT:**
La risposta corretta è B. Il tetto deve essere riparato.
**Punteggio: 1**

- **Prompt - Item 17:**Anna ha chiesto a Maria a che ora chiude la biblioteca, ma lei lo ha dimenticato.

Domanda: Chi ‘lo ha dimenticato’?

**A. Maria**

B. Anna

**Risposta ChatGPT:**
La risposta corretta è A. Maria ha dimenticato a che ora chiude la biblioteca.
**Punteggio: 1**

- **Prompt - Item 18:**Pietro invidia Martino anche se ha molto successo.

Domanda: Chi ‘ha molto successo’?

**A. Pietro**

B. Martino

**Risposta ChatGPT:**
La risposta corretta è B. Martino ha molto successo.
**Punteggio: 0**

- **Prompt - Item 19:**La poltrona non può passare attraverso la porta perché è troppo stretta.

Domanda: Cos'è troppo stretta?

A. La poltrona

**B. La porta**

**Risposta ChatGPT:**
La risposta corretta è B. La porta è troppo stretta.
**Punteggio: 1**

- **Prompt - Item 20:**Tommaso ha dato a Raffaele un passaggio a scuola così non avrebbe dovuto guidare da solo.

Domanda: Chi ‘non avrebbe dovuto guidare da solo’?

**A. Tommaso**

B. Raffaele

**Risposta ChatGPT:**
La risposta corretta è B. Raffaele non avrebbe dovuto guidare da solo.
**Punteggio: 0**

**Totale Referenze Anaforiche: 16/20**

# **Torre di Londra** (Bruni et al., 2022)

**Note:** For visualisation purposes, we report at the beginning the Starting Position that refers to all the Items of the Tower of London. For each Item, after each prompt and before ChatGPT response, we report the Goal Position described in the prompt.

# **POSIZIONE DI PARTENZA**

**PROMPT – ITEM 1:**

Qui ci sono tre pioli di lunghezza diversa: uno grande, uno medio, e uno piccolo. Ci sono poi tre palline di colore diverso: una arancione, una celeste, una verde.

Partendo dalla [POSIZIONE DI PARTENZA], ti chiederò di scrivermi quali sono le sequenze di azioni che un essere umano dovrebbe compiere per poter posizionare le palline sui pioli così come indicato nella [POSIZIONE DA RAGGIUNGERE].

Ci sono delle regole da rispettare:

1. Non si possono muovere due palline contemporaneamente: puoi muovere solo una pallina per volta;
2. Non si può prendere una pallina e tenerla in mano mentre se ne muove un’altra: puoi muovere quindi solo da un piolo ad un altro;
3. Le palline possono trovarsi una sopra l’altra sullo stesso piolo.
4. I pioli sono di dimensioni diverse: puoi collocare una sola pallina sul piolo piccolo, due sul piolo medio e tre sul piolo grande
5. Se più palline sono sullo stesso piolo devi prima spostare la pallina più in cima. Non puoi spostare prima la pallina più in fondo se è presenta un’altra pallina in cima.
6. Risolvi questo compito in 3 mosse.

[POSIZIONE DI PARTENZA]

In questo momento la pallina arancione è nel piolo grande. La pallina verde è nel piolo grande. La pallina arancione è sopra la pallina verde nel piolo grande. La pallina celeste è nel piolo medio. Il piolo piccolo è vuoto.

[ESEMPIO]

La pallina arancione è nel piolo grande. La pallina celeste è nel piolo medio. La pallina verde è nel piolo medio. La pallina celeste è sotto il la pallina verde nel piolo medio. Il piolo piccolo è vuoto.

[SOLUZIONE]

1. Muovi la pallina arancione dal piolo grande al piolo piccolo.
2. Muovi la pallina verde dal piolo grande al piolo medio. In questo modo, la pallina verde sarà sopra la pallina celeste.
3. Muovi la pallina arancione dal piolo piccolo a quello grande.

[POSIZIONE DA RAGGIUNGERE]

La pallina verde è nel piolo grande. La pallina celeste è nel piolo grande. La pallina celeste è nel piolo grande sopra la pallina verde. La pallina arancione è nel piolo medio.

# **ESEMPIO (3 MOSSE)**

# **3 MOSSE**

**RISPOSTA CHATGPT - PRIMO TENTATIVO:**

[SOLUZIONE]

1. Muovi la pallina celeste dal piolo grande al piolo medio. In questo modo, la pallina celeste sarà sopra la pallina arancione.
2. Muovi la pallina verde dal piolo grande al piolo medio. In questo modo, la pallina verde sarà sotto la pallina celeste.
3. Muovi la pallina celeste dal piolo medio al piolo grande. In questo modo, la pallina celeste sarà sopra la pallina verde nel piolo grande e la pallina arancione sarà nel piolo medio.

**PROMPT - FEEDBACK:**

ATTENZIONE, la pallina celeste è inizialmente nel piolo medio e non nel piolo grande.

**RISPOSTA CHATGPT - SECONDO TENTATIVO:**

Mi scuso per l'errore nella mia risposta precedente. Ecco la soluzione corretta per la posizione di partenza e quella da raggiungere corretta:

[POSIZIONE DI PARTENZA] In questo momento la pallina arancione è nel piolo grande. La pallina verde è nel piolo grande. La pallina arancione è sopra la pallina verde nel piolo grande. La pallina celeste è nel piolo medio. Il piolo piccolo è vuoto.

[POSIZIONE DA RAGGIUNGERE] La pallina verde è nel piolo grande. La pallina celeste è nel piolo grande. La pallina celeste è nel piolo grande sopra la pallina verde. La pallina arancione è nel piolo medio.

[SOLUZIONE]

1. Muovi la pallina celeste dal piolo medio al piolo grande. In questo modo, la pallina celeste sarà sopra la pallina verde nel piolo grande.
2. Muovi la pallina verde dal piolo grande al piolo medio.
3. Muovi la pallina arancione dal piolo grande al piolo medio. In questo modo, la pallina arancione sarà nel piolo medio e la pallina celeste sarà sopra la pallina verde nel piolo grande.

**PROMPT - FEEDBACK:**

Attenzione! non puoi muovere la pallina verde dal piolo grande al piolo medio se sopra di essa ci sono delle palline.

**RISPOSTA CHATGPT - TERZO TENTATIVO:**

Mi scuso ancora per l'errore nella mia risposta precedente. Ecco la soluzione corretta per la posizione di partenza e quella da raggiungere:

[POSIZIONE DI PARTENZA] In questo momento la pallina arancione è nel piolo grande. La pallina verde è nel piolo grande. La pallina arancione è sopra la pallina verde nel piolo grande. La pallina celeste è nel piolo medio. Il piolo piccolo è vuoto.

[POSIZIONE DA RAGGIUNGERE] La pallina verde è nel piolo grande. La pallina celeste è nel piolo grande. La pallina celeste è nel piolo grande sopra la pallina verde. La pallina arancione è nel piolo medio.

[SOLUZIONE]

1. Muovi la pallina arancione dal piolo grande al piolo piccolo.

2. Muovi la pallina celeste dal piolo medio al piolo grande. In questo modo, la pallina celeste sarà sopra la pallina verde nel piolo grande.

3. Muovi la pallina arancione dal piolo piccolo al piolo medio. In questo modo, la pallina arancione sarà nel piolo medio e la pallina celeste sarà sopra la pallina verde nel piolo grande.

**PUNTEGGIO: 1**

**PROMPT – ITEM 2:**

Qui ci sono tre pioli di lunghezza diversa: uno grande, uno medio, e uno piccolo. Ci sono poi tre palline di colore diverso: una arancione, una celeste, una verde.

Partendo dalla [POSIZIONE DI PARTENZA], ti chiederò di scrivermi quali sono le sequenze di azioni che un essere umano dovrebbe compiere per poter posizionare le palline sui pioli così come indicato nella [POSIZIONE DA RAGGIUNGERE].

Ci sono delle regole da rispettare:

1. Non si possono muovere due palline contemporaneamente: puoi muovere solo una pallina per volta;
2. Non si può prendere una pallina e tenerla in mano mentre se ne muove un’altra: puoi muovere quindi solo da un piolo ad un altro;
3. Le palline possono trovarsi una sopra l’altra sullo stesso piolo.
4. I pioli sono di dimensioni diverse: puoi collocare una sola pallina sul piolo piccolo, due sul piolo medio e tre sul piolo grande
5. Se più palline sono sullo stesso piolo devi prima spostare la pallina più in cima. Non puoi spostare prima la pallina più in fondo se è presenta un’altra pallina in cima.
6. Risolvi questo compito in 2 mosse.

[POSIZIONE DI PARTENZA]

In questo momento la pallina arancione è nel piolo grande. La pallina verde è nel piolo grande. La pallina arancione è sopra la pallina verde nel piolo grande. La pallina celeste è nel piolo medio. Il piolo piccolo è vuoto.

[POSIZIONE DA RAGGIUNGERE]

La pallina arancione è nel piolo piccolo. La pallina celeste è nel piolo medio. La pallina verde è nel piolo medio. La pallina celeste è sotto la pallina verde nel piolo medio. Il piolo grande è vuoto.

# **2 MOSSE**

**RISPOSTA CHATGPT - PRIMO TENTATIVO:**

Mi dispiace, ma non è possibile rispettare le regole specificate e raggiungere la posizione desiderata in solamente due mosse. Poiché il piolo piccolo può contenere solo una pallina, la pallina verde o la pallina celeste dovrebbero essere spostate prima che la pallina arancione possa essere posizionata sul piolo piccolo. Tuttavia, questo richiederebbe almeno tre mosse.

**PROMPT – FEEDBACK 1 :**

Ti assicuro che il compito si può fare in due semplici mosse.

**RISPOSTA CHATGPT - SECONDO TENTATIVO:**

Mi scuso per l'errore precedente. Ecco una possibile soluzione in 2 mosse:

Mossa 1: spostare la pallina celeste dal piolo medio al piolo grande, ponendola sotto le altre due palline.

[POSIZIONE ATTUALE] Pallina arancione e pallina verde nel piolo grande. Pallina celeste nel piolo grande, sotto le altre due. Piolo medio vuoto. Piolo piccolo vuoto.

[POSIZIONE INTERMEDIA] Pallina arancione e pallina verde nel piolo grande. Pallina celeste nel piolo medio. Piolo piccolo vuoto.

Mossa 2: spostare le tre palline dal piolo grande al piolo piccolo, partendo dalla pallina più in basso e procedendo verso quella più in alto.

[POSIZIONE ATTUALE] Piolo grande vuoto. Pallina celeste nel piolo medio. Pallina verde e pallina arancione nel piolo piccolo, con la pallina arancione sopra la pallina verde.

[POSIZIONE FINALE] Pallina arancione nel piolo piccolo. Pallina celeste nel piolo medio. Pallina verde nel piolo medio, sopra la pallina celeste. Piolo grande vuoto.

**PROMPT – FEEDBACK 2:**

Attenzione, non puoi spostare la pallina celeste dal piolo medio al piolo grande, ponendola sotto le altre due palline perché violerebbe la regola numero 5. Riprova a risolvere il compito in due sole mosse.

**RISPOSTA CHATGPT - TERZO TENTATIVO:**

Mi scuso per l'errore nella mia risposta precedente. Ecco una possibile soluzione che rispetta tutte le regole e che richiede due sole mosse:

1. Prendi la pallina arancione dal piolo grande e spostala sul piolo piccolo.
2. Prendi la pallina celeste dal piolo medio e spostala sul piolo grande.

In questo modo, alla fine avrai la pallina arancione sul piolo piccolo, la pallina celeste sul piolo grande e la pallina verde sul piolo medio con la celeste sotto di essa. Il piolo grande sarà vuoto.

**PUNTEGGIO: 0**

**PROMPT – ITEM 3:**

Qui ci sono tre pioli di lunghezza diversa: uno grande, uno medio, e uno piccolo. Ci sono poi tre palline di colore diverso: una arancione, una celeste, una verde.

Partendo dalla [POSIZIONE DI PARTENZA], ti chiederò di scrivermi quali sono le sequenze di azioni che un essere umano dovrebbe compiere per poter posizionare le palline sui pioli così come indicato nella [POSIZIONE DA RAGGIUNGERE].

Ci sono delle regole da rispettare:

1. Non si possono muovere due palline contemporaneamente: puoi muovere solo una pallina per volta;
2. Non si può prendere una pallina e tenerla in mano mentre se ne muove un’altra: puoi muovere quindi solo da un piolo ad un altro;
3. Le palline possono trovarsi una sopra l’altra sullo stesso piolo.
4. I pioli sono di dimensioni diverse: puoi collocare una sola pallina sul piolo piccolo, due sul piolo medio e tre sul piolo grande
5. Se più palline sono sullo stesso piolo devi prima spostare la pallina più in cima. Non puoi spostare prima la pallina più in fondo se è presenta un’altra pallina in cima.
6. Risolvi questo compito in 2 mosse.

[POSIZIONE DI PARTENZA]

In questo momento la pallina arancione è nel piolo grande. La pallina verde è nel piolo grande. La pallina arancione è sopra la pallina verde nel piolo grande. La pallina celeste è nel piolo medio. Il piolo piccolo è vuoto.

[POSIZIONE DA RAGGIUNGERE]

La pallina verde è nel piolo grande. La pallina arancione è nel piolo medio. La pallina celeste è nel piolo piccolo.

# **2 MOSSE**

**RISPOSTA CHATGPT - PRIMO TENTATIVO:**

Ecco una possibile soluzione in 2 mosse:

Mossa 1: sposta la pallina celeste dal piolo medio al piolo piccolo. Mossa 2: sposta la pallina arancione dal piolo grande al piolo medio.

La posizione finale sarà:

1. Pallina verde sul piolo grande
2. Pallina arancione sul piolo medio
3. Pallina celeste sul piolo piccolo

**PUNTEGGIO: 3**

**PROMPT – ITEM 4:**

Qui ci sono tre pioli di lunghezza diversa: uno grande, uno medio, e uno piccolo. Ci sono poi tre palline di colore diverso: una arancione, una celeste, una verde.

Partendo dalla [POSIZIONE DI PARTENZA], ti chiederò di scrivermi quali sono le sequenze di azioni che un essere umano dovrebbe compiere per poter posizionare le palline sui pioli così come indicato nella [POSIZIONE DA RAGGIUNGERE].

Ci sono delle regole da rispettare:

1. Non si possono muovere due palline contemporaneamente: puoi muovere solo una pallina per volta;
2. Non si può prendere una pallina e tenerla in mano mentre se ne muove un’altra: puoi muovere quindi solo da un piolo ad un altro;
3. Le palline possono trovarsi una sopra l’altra sullo stesso piolo.
4. I pioli sono di dimensioni diverse: puoi collocare una sola pallina sul piolo piccolo, due sul piolo medio e tre sul piolo grande
5. Se più palline sono sullo stesso piolo devi prima spostare la pallina più in cima. Non puoi spostare prima la pallina più in fondo se è presenta un’altra pallina in cima.
6. Risolvi questo compito in 3 mosse.

[POSIZIONE DI PARTENZA]

In questo momento la pallina arancione è nel piolo grande. La pallina verde è nel piolo grande. La pallina arancione è sopra la pallina verde nel piolo grande. La pallina celeste è nel piolo medio. Il piolo piccolo è vuoto.

[POSIZIONE DA RAGGIUNGERE]

La pallina verde è nel piolo grande. La pallina arancione è nel piolo medio. La pallina celeste è nel piolo medio sopra la pallina arancione. Il piolo piccolo è vuoto.

# **3 MOSSE**

**RISPOSTA CHATGPT - PRIMO TENTATIVO:**

Ecco le sequenze di azioni per spostare le palline nella posizione richiesta in 3 mosse:

Mossa 1: Sposta la pallina celeste dal piolo medio al piolo piccolo. Mossa 2: Sposta la pallina verde dal piolo grande al piolo medio sopra la pallina arancione.

Mossa 3:Sposta la pallina arancione dal piolo grande al piolo medio sopra la pallina celeste.

**PROMPT - FEEDBACK:**

Attenzione, non puoi spostare la pallina verde dal piolo grande al piolo medio perchè violerebbe la regola numero 5. Riprova a risolvere il compito in 3 mosse.

**RISPOSTA CHATGPT - SECONDO TENTATIVO:**

Mi scuso per l'errore nella mia risposta precedente. Ecco una possibile soluzione in 3 mosse che rispetta tutte le regole:

[POSIZIONE DI PARTENZA] In questo momento la pallina arancione è nel piolo grande. La pallina verde è nel piolo grande. La pallina arancione è sopra la pallina verde nel piolo grande. La pallina celeste è nel piolo medio. Il piolo piccolo è vuoto.

[POSIZIONE DA RAGGIUNGERE] La pallina verde è nel piolo grande. La pallina arancione è nel piolo medio. La pallina celeste è nel piolo grande sopra la pallina verde. Il piolo piccolo è vuoto.

Sequenza di azioni:

1. Sposta la pallina celeste dal piolo medio al piolo grande (sopra la pallina verde).

2. Sposta la pallina arancione dal piolo grande al piolo medio.

3. Sposta la pallina celeste dal piolo grande sopra la pallina arancione nel piolo medio.

**PROMPT - FEEDBACK:**

ATTENZIONE, data la posizione di partenza, se sposti la pallina celeste dal piolo medio al piolo grande essa andrà sopra la pallina arancione, la quale è sopra la pallina verde. Pertanto, non potrai eseguire la mossa 2, spostando la pallina arancione dal piolo grande al piolo medio perché violerebbe la regola numero 5. Riprova a risolvere il task in 3 mosse.

**RISPOSTA CHATGPT - TERZO TENTATIVO:**

Ecco una possibile soluzione in tre mosse:

Mossa 1: Sposta la pallina celeste dal piolo medio al piolo piccolo.

Mossa 2: Sposta la pallina arancione dal piolo grande al piolo medio.

Mossa 3: Sposta la pallina celeste dal piolo piccolo al piolo medio sopra la pallina arancione.

In questo modo, la pallina verde rimane nel piolo grande, la pallina celeste si sposta sopra la pallina arancione nel piolo medio, e la pallina arancione va nel piolo medio. Il piolo piccolo rimane vuoto alla fine.

**PUNTEGGIO: 1**

**PROMPT – ITEM 5:**

Qui ci sono tre pioli di lunghezza diversa: uno grande, uno medio, e uno piccolo. Ci sono poi tre palline di colore diverso: una arancione, una celeste, una verde.

Partendo dalla [POSIZIONE DI PARTENZA], ti chiederò di scrivermi quali sono le sequenze di azioni che un essere umano dovrebbe compiere per poter posizionare le palline sui pioli così come indicato nella [POSIZIONE DA RAGGIUNGERE].

Ci sono delle regole da rispettare:

1. Non si possono muovere due palline contemporaneamente: puoi muovere solo una pallina per volta;
2. Non si può prendere una pallina e tenerla in mano mentre se ne muove un’altra: puoi muovere quindi solo da un piolo ad un altro;
3. Le palline possono trovarsi una sopra l’altra sullo stesso piolo.
4. I pioli sono di dimensioni diverse: puoi collocare una sola pallina sul piolo piccolo, due sul piolo medio e tre sul piolo grande
5. Se più palline sono sullo stesso piolo devi prima spostare la pallina più in cima. Non puoi spostare prima la pallina più in fondo se è presenta un’altra pallina in cima.
6. Risolvi questo compito in 4 mosse.

[POSIZIONE DI PARTENZA]

In questo momento la pallina arancione è nel piolo grande. La pallina verde è nel piolo grande. La pallina arancione è sopra la pallina verde nel piolo grande. La pallina celeste è nel piolo medio. Il piolo piccolo è vuoto.

[POSIZIONE DA RAGGIUNGERE]

La pallina arancione è nel piolo grande. La pallina verde è nel piolo grande sopra la pallina arancione. La pallina celeste è nel piolo medio. Il piolo piccolo è vuoto.

# **4 MOSSE**

**RISPOSTA CHATGPT - PRIMO TENTATIVO:**

Ecco una possibile soluzione in 4 mosse:

1. Muovi la pallina celeste dal piolo medio al piolo piccolo.

2. Muovi la pallina verde dal piolo grande al piolo medio.

3. Muovi la pallina celeste dal piolo piccolo al piolo grande.

4. Muovi la pallina verde dal piolo medio al piolo grande sopra la pallina arancione.

**PROMPT - FEEDBACK:**

Attenzione, non puoi eseguire la mossa 2, perché la pallina verde è sotto la pallina arancione, pertanto violeresti la regola n. 5. Riprova a risolvere il compito in 4 mosse.

**RISPOSTA CHATGPT - SECONDO TENTATIVO:**

Ecco una possibile soluzione in 4 mosse:

1. Sposta la pallina celeste dal piolo medio al piolo piccolo.

2. Sposta la pallina verde dal piolo grande al piolo medio.

3. Sposta la pallina celeste dal piolo piccolo al piolo grande.

4. Sposta la pallina verde dal piolo medio al piolo grande sopra la pallina arancione.

[POSIZIONE FINALE]

La pallina arancione è nel piolo grande. La pallina verde è nel piolo grande sopra la pallina arancione. La pallina celeste è nel piolo grande. Il piolo piccolo è vuoto.

**PROMPT - FEEDBACK:**

Attenzione, non puoi spostare la pallina verde dal piolo grande al piolo medio senza prima spostare la pallina arancione perchè violeresti la regola n. 5. Riprova a risolvere il compito in 4 mosse.

**RISPOSTA CHATGPT - TERZO TENTATIVO:**

Ecco una possibile soluzione in 4 mosse:

1. Sposta la pallina celeste dal piolo medio al piolo piccolo

2. Sposta la pallina arancione dal piolo grande al piolo medio

3. Sposta la pallina celeste dal piolo piccolo al piolo medio

4. Sposta la pallina verde dal piolo grande al piolo grande sopra la pallina arancione.

[POSIZIONE FINALE]

La pallina arancione è nel piolo medio. La pallina verde è nel piolo grande sopra la pallina arancione. La pallina celeste è nel piolo medio. Il piolo piccolo è vuoto.

**PUNTEGGIO: 0**

**PROMPT – ITEM 6:**

Qui ci sono tre pioli di lunghezza diversa: uno grande, uno medio, e uno piccolo. Ci sono poi tre palline di colore diverso: una arancione, una celeste, una verde.

Partendo dalla [POSIZIONE DI PARTENZA], ti chiederò di scrivermi quali sono le sequenze di azioni che un essere umano dovrebbe compiere per poter posizionare le palline sui pioli così come indicato nella [POSIZIONE DA RAGGIUNGERE].

Ci sono delle regole da rispettare:

1. Non si possono muovere due palline contemporaneamente: puoi muovere solo una pallina per volta;
2. Non si può prendere una pallina e tenerla in mano mentre se ne muove un’altra: puoi muovere quindi solo da un piolo ad un altro;
3. Le palline possono trovarsi una sopra l’altra sullo stesso piolo.
4. I pioli sono di dimensioni diverse: puoi collocare una sola pallina sul piolo piccolo, due sul piolo medio e tre sul piolo grande
5. Se più palline sono sullo stesso piolo devi prima spostare la pallina più in cima. Non puoi spostare prima la pallina più in fondo se è presenta un’altra pallina in cima.
6. Risolvi questo compito in 4 mosse.

[POSIZIONE DI PARTENZA]

In questo momento la pallina arancione è nel piolo grande. La pallina verde è nel piolo grande. La pallina arancione è sopra la pallina verde nel piolo grande. La pallina celeste è nel piolo medio. Il piolo piccolo è vuoto.

[POSIZIONE DA RAGGIUNGERE]

La pallina celeste è nel piolo grande. La pallina arancione è nel piolo medio. La pallina verde è nel piolo medio sopra la pallina arancione. Il piolo piccolo è vuoto.

# **4 MOSSE**

**RISPOSTA CHATGPT - PRIMO TENTATIVO:**

Ecco le mosse per raggiungere la posizione desiderata in 4 mosse:

1. Muovi la pallina celeste dal piolo medio al piolo grande.

2. Muovi la pallina arancione dal piolo grande al piolo medio.

3. Muovi la pallina verde dal piolo grande al piolo medio sopra la pallina arancione.

4. Il piolo piccolo è già vuoto, quindi non c'è bisogno di fare altre mosse.

**PROMPT - FEEDBACK:**

Attenzione! Non puoi spostare la pallina arancione dal piolo grande al piolo medio dopo aver spostato la pallina celeste sul piolo grande perché altrimenti violeresti la regola n. 5. Riprova a risolvere il compito in 4 mosse.

**RISPOSTA CHATGPT - SECONDO TENTATIVO:**

Ecco una possibile soluzione in 4 mosse:

1. Muovi la pallina celeste dal piolo medio al piolo grande.

2. Muovi la pallina arancione dal piolo grande al piolo piccolo.

3. Muovi la pallina celeste dal piolo grande al piolo medio.

4. Muovi la pallina verde dal piolo grande al piolo medio sopra la pallina arancione.

[POSIZIONE DI PARTENZA]

In questo momento la pallina arancione è nel piolo grande. La pallina verde è nel piolo grande. La pallina arancione è sopra la pallina verde nel piolo grande. La pallina celeste è nel piolo medio. Il piolo piccolo è vuoto.

[POSIZIONE FINALE] La pallina celeste è nel piolo grande. La pallina arancione è nel piolo medio. La pallina verde è nel piolo medio sopra la pallina arancione. Il piolo piccolo è vuoto.

**PROMPT - FEEDBACK:**

Attenzione! se sposti la pallina celeste dal piolo medio al piolo grande e poi sposti la pallina arancione dal piolo grande al piolo piccolo, stai violando la regola n. 5! Non puoi spostare una pallina se ce n'è un'altra sopra di essa. Riprova a risolvere il compito in 4 mosse.

**RISPOSTA CHATGPT - TERZO TENTATIVO:**

Ecco una soluzione in 4 mosse per raggiungere la posizione richiesta rispettando tutte le regole:

Mossa 1: Sposta la pallina celeste dal piolo medio al piolo piccolo.

Mossa 2: Sposta la pallina verde dal piolo grande al piolo medio.

Mossa 3: Sposta la pallina celeste dal piolo piccolo al piolo grande.

Mossa 4: Sposta la pallina arancione dal piolo grande al piolo medio.

Nota che le regole sono rispettate in ogni passaggio:

- In ogni mossa, viene spostata solo una pallina.
- Non viene mai presa una pallina e tenuta in mano mentre se ne muove un'altra.
- In ogni piolo possono essere presenti più palline una sopra l'altra, a patto che la pallina più in cima venga sempre spostata per prima (regola n. 5).
- Viene rispettato il limite massimo di palline per ogni piolo (regola n. 4).

**PUNTEGGIO: 0**

**PROMPT – ITEM 7:**

Qui ci sono tre pioli di lunghezza diversa: uno grande, uno medio, e uno piccolo. Ci sono poi tre palline di colore diverso: una arancione, una celeste, una verde.

Partendo dalla [POSIZIONE DI PARTENZA], ti chiederò di scrivermi quali sono le sequenze di azioni che un essere umano dovrebbe compiere per poter posizionare le palline sui pioli così come indicato nella [POSIZIONE DA RAGGIUNGERE].

Ci sono delle regole da rispettare:

1. Non si possono muovere due palline contemporaneamente: puoi muovere solo una pallina per volta;
2. Non si può prendere una pallina e tenerla in mano mentre se ne muove un’altra: puoi muovere quindi solo da un piolo ad un altro;
3. Le palline possono trovarsi una sopra l’altra sullo stesso piolo.
4. I pioli sono di dimensioni diverse: puoi collocare una sola pallina sul piolo piccolo, due sul piolo medio e tre sul piolo grande
5. Se più palline sono sullo stesso piolo devi prima spostare la pallina più in cima. Non puoi spostare prima la pallina più in fondo se è presenta un’altra pallina in cima.
6. Risolvi questo compito in 4 mosse.

[POSIZIONE DI PARTENZA]

In questo momento la pallina arancione è nel piolo grande. La pallina verde è nel piolo grande. La pallina arancione è sopra la pallina verde nel piolo grande. La pallina celeste è nel piolo medio. Il piolo piccolo è vuoto.

[POSIZIONE DA RAGGIUNGERE]

La pallina arancione è nel piolo grande. La pallina celeste è nel piolo grande sopra la pallina arancione. Il piolo medio è vuoto. La pallina verde è nel piolo piccolo.

# **4 MOSSE**

**RISPOSTA CHATGPT - PRIMO TENTATIVO:**

Ecco una possibile soluzione in 4 mosse:

Mossa 1: Sposta la pallina celeste dal piolo medio al piolo grande, sopra la pallina arancione.

Mossa 2: Sposta la pallina arancione dal piolo grande al piolo piccolo.

Mossa 3: Sposta la pallina verde dal piolo grande al piolo medio.

Mossa 4: Sposta la pallina celeste dal piolo grande al piolo medio, sopra la pallina verde.

[POSIZIONE FINALE]

La pallina arancione è nel piolo grande. La pallina celeste è nel piolo medio sopra la pallina verde. Il piolo medio è vuoto. La pallina verde è nel piolo piccolo.

**PROMPT - FEEDBACK:**

Attenzione! Non puoi spostare la pallina arancione dal piolo grande al piolo piccolo (mossa 2) o medio se prima hai spostato la pallina celeste sopra la pallina arancione (mossa 1) altrimenti violeresti la regola n.5. Riprova a risolvere il compito in 4 mosse.

**RISPOSTA CHATGPT - SECONDO TENTATIVO:**

Ecco una possibile sequenza di mosse per raggiungere la posizione richiesta in 4 mosse, rispettando le regole indicate:

1. Sposta la pallina celeste dal piolo medio al piolo grande sopra la pallina arancione.

2. Sposta la pallina verde dal piolo grande al piolo piccolo.

3. Sposta la pallina celeste dal piolo grande al piolo medio.

4. Sposta la pallina arancione dal piolo grande al piolo grande sopra la pallina celeste.

La posizione finale sarà la seguente: La pallina arancione è nel piolo grande sopra la pallina celeste. La pallina celeste è nel piolo medio. La pallina verde è nel piolo piccolo.

**PROMPT - FEEDBACK:**

Attenzione! Non puoi spostare la pallina verde dal piolo grande al piolo piccolo (mossa 2) senza aver prima spostato la pallina arancione sopra di essa e dopo aver spostato la pallina celeste sopra la pallina arancione (mossa 1) altrimenti violeresti la regola n.5. Riprova a risolvere il compito in 4 mosse.

**RISPOSTA CHATGPT - TERZO TENTATIVO:**

Ecco una possibile soluzione in 4 mosse:

1. Sposta la pallina celeste dal piolo medio al piolo grande sopra alla pallina arancione.

2. Sposta la pallina arancione dal piolo grande al piolo medio.

3. Sposta la pallina celeste dal piolo grande al piolo piccolo.

4. Sposta la pallina verde dal piolo grande al piolo piccolo.

Questa soluzione rispetta tutte le regole indicate nel problema.

**PUNTEGGIO: 0**

**PROMPT – ITEM 8:**

Qui ci sono tre pioli di lunghezza diversa: uno grande, uno medio, e uno piccolo. Ci sono poi tre palline di colore diverso: una arancione, una celeste, una verde.

Partendo dalla [POSIZIONE DI PARTENZA], ti chiederò di scrivermi quali sono le sequenze di azioni che un essere umano dovrebbe compiere per poter posizionare le palline sui pioli così come indicato nella [POSIZIONE DA RAGGIUNGERE].

Ci sono delle regole da rispettare:

1. Non si possono muovere due palline contemporaneamente: puoi muovere solo una pallina per volta;
2. Non si può prendere una pallina e tenerla in mano mentre se ne muove un’altra: puoi muovere quindi solo da un piolo ad un altro;
3. Le palline possono trovarsi una sopra l’altra sullo stesso piolo.
4. I pioli sono di dimensioni diverse: puoi collocare una sola pallina sul piolo piccolo, due sul piolo medio e tre sul piolo grande
5. Se più palline sono sullo stesso piolo devi prima spostare la pallina più in cima. Non puoi spostare prima la pallina più in fondo se è presenta un’altra pallina in cima.
6. Risolvi questo compito in 4 mosse.

[POSIZIONE DI PARTENZA]

In questo momento la pallina arancione è nel piolo grande. La pallina verde è nel piolo grande. La pallina arancione è sopra la pallina verde nel piolo grande. La pallina celeste è nel piolo medio. Il piolo piccolo è vuoto.

[POSIZIONE DA RAGGIUNGERE]

Il piolo grande è vuoto. La pallina arancione è nel piolo medio. La pallina celeste è nel piolo medio sopra la pallina arancione. La pallina verde è nel piolo piccolo.

# **4 MOSSE**

**RISPOSTA CHATGPT - PRIMO TENTATIVO:**

Ecco le 4 mosse per raggiungere la posizione richiesta:

1. Sposta la pallina celeste dal piolo medio al piolo grande.

2. Sposta la pallina arancione dal piolo grande al piolo medio.

3. Sposta la pallina celeste dal piolo grande al piolo medio sopra la pallina arancione.

4. Sposta la pallina verde dal piolo grande al piolo piccolo.

**PROMPT - FEEDBACK:**

Attenzione! Non puoi spostare la pallina arancione dal piolo grande al piolo medio (mossa 2) se prima hai spostato la pallina celeste sopra la pallina arancione (mossa 1) altrimenti violeresti la regola n.5. Riprova a risolvere il compito in 4 mosse.

**RISPOSTA CHATGPT - SECONDO TENTATIVO: – CORRETTO**

Ecco la sequenza di azioni in 4 mosse per raggiungere la [POSIZIONE DA RAGGIUNGERE]:

1. Sposta la pallina celeste dal piolo medio al piolo piccolo.

2. Sposta la pallina arancione dal piolo grande al piolo medio.

3. Sposta la pallina celeste dal piolo piccolo al piolo medio sopra la pallina arancione.

4. Sposta la pallina verde dal piolo grande al piolo piccolo.

Ricorda che non puoi muovere due palline contemporaneamente (regola n.1) e che non puoi spostare la pallina arancione dal piolo grande al piolo medio (mossa 2) senza aver prima spostato la pallina celeste sopra di essa e dopo aver spostato la pallina verde dal piolo grande al piolo piccolo (mossa 4), altrimenti violeresti la regola n.5.

**PUNTEGGIO: 2**

**PROMPT – ITEM 9:**

Qui ci sono tre pioli di lunghezza diversa: uno grande, uno medio, e uno piccolo. Ci sono poi tre palline di colore diverso: una arancione, una celeste, una verde.

Partendo dalla [POSIZIONE DI PARTENZA], ti chiederò di scrivermi quali sono le sequenze di azioni che un essere umano dovrebbe compiere per poter posizionare le palline sui pioli così come indicato nella [POSIZIONE DA RAGGIUNGERE].

Ci sono delle regole da rispettare:

1. Non si possono muovere due palline contemporaneamente: puoi muovere solo una pallina per volta;
2. Non si può prendere una pallina e tenerla in mano mentre se ne muove un’altra: puoi muovere quindi solo da un piolo ad un altro;
3. Le palline possono trovarsi una sopra l’altra sullo stesso piolo.
4. I pioli sono di dimensioni diverse: puoi collocare una sola pallina sul piolo piccolo, due sul piolo medio e tre sul piolo grande
5. Se più palline sono sullo stesso piolo devi prima spostare la pallina più in cima. Non puoi spostare prima la pallina più in fondo se è presenta un’altra pallina in cima.
6. Risolvi questo compito in 5 mosse.

[POSIZIONE DI PARTENZA]

In questo momento la pallina arancione è nel piolo grande. La pallina verde è nel piolo grande. La pallina arancione è sopra la pallina verde nel piolo grande. La pallina celeste è nel piolo medio. Il piolo piccolo è vuoto.

[POSIZIONE DA RAGGIUNGERE]

La pallina arancione è nel piolo grande. La pallina verde è nel piolo grande sopra la pallina arancione. La pallina celeste è nel piolo grande sopra la pallina verde. I pioli medio e piccolo sono vuoti.

# **5 MOSSE**

**RISPOSTA CHATGPT - PRIMO TENTATIVO:**

Ecco le mosse necessarie per raggiungere la posizione finale in 5 mosse:

1. Sposta la pallina celeste dal piolo medio al piolo piccolo.

2. Sposta la pallina verde dal piolo grande al piolo medio.

3. Sposta la pallina arancione dal piolo grande al piolo grande sopra la pallina verde.

4. Sposta la pallina celeste dal piolo piccolo al piolo grande sopra la pallina verde e la pallina arancione.

5. Sposta la pallina verde dal piolo medio al piolo grande sopra la pallina celeste.

**PROMPT - FEEDBACK:**

Attenzione! Non puoi spostare la pallina verde dal piolo grande al piolo medio (mossa 2) se prima non hai spostato la pallina arancione, altrimenti violeresti la regola n.5. Riprova a risolvere il compito in 5 mosse.

**RISPOSTA CHATGPT - SECONDO TENTATIVO:**

Mi scuso per l'errore nella mia precedente risposta. Ecco una nuova soluzione in 5 mosse che rispetta la regola n.5:

1. Sposta la pallina celeste dal piolo medio al piolo piccolo.

2. Sposta la pallina arancione dal piolo grande al piolo medio.

3. Sposta la pallina celeste dal piolo piccolo al piolo medio.

4. Sposta la pallina verde dal piolo grande al piolo medio.

5. Sposta la pallina celeste dal piolo medio al piolo piccolo.

[POSIZIONE FINALE]

La pallina arancione è nel piolo medio. La pallina verde è nel piolo medio sopra la pallina arancione. La pallina celeste è nel piolo piccolo sopra la pallina verde. Il piolo grande è vuoto.Inizio modulo

Fine modulo

**PROMPT - FEEDBACK:**

Attenzione! Se la pallina celeste è stata spostata la piolo medio (mossa 3) e dopo è stata spostata la pallina verde al piolo medio (mossa 4), non puoi spostare la pallina celeste al piolo piccolo senza aver spostato la pallina verde. Inoltre, la [POSIZIONE FINALE] prima descritta non rispetta la [POSIZIONE DA RAGGIUNGERE].

**RISPOSTA CHATGPT - TERZO TENTATIVO:**

Mi scuso per l'errore nella risposta precedente. In effetti, la pallina celeste non può essere spostata al piolo piccolo se la pallina verde non è stata prima spostata. Inoltre, la [POSIZIONE FINALE] descritta non rispetta la [POSIZIONE DA RAGGIUNGERE].

Ecco una possibile soluzione che rispetta tutte le regole:

1. Muovi la pallina arancione dal piolo grande al piolo medio.

2. Muovi la pallina verde dal piolo grande al piolo medio sopra la pallina celeste.

3. Muovi la pallina celeste dal piolo medio al piolo grande sopra la pallina verde.

4. Muovi la pallina verde dal piolo medio al piolo piccolo.

5. Muovi la pallina celeste dal piolo grande al piolo medio sopra la pallina arancione.

La [POSIZIONE FINALE] sarà la seguente: La pallina arancione è nel piolo medio. La pallina verde è nel piolo piccolo. La pallina celeste è nel piolo medio sopra la pallina arancione. Il piolo grande è vuoto.

**PUNTEGGIO: 0**

**PROMPT – ITEM 10:**

Qui ci sono tre pioli di lunghezza diversa: uno grande, uno medio, e uno piccolo. Ci sono poi tre palline di colore diverso: una arancione, una celeste, una verde.

Partendo dalla [POSIZIONE DI PARTENZA], ti chiederò di scrivermi quali sono le sequenze di azioni che un essere umano dovrebbe compiere per poter posizionare le palline sui pioli così come indicato nella [POSIZIONE DA RAGGIUNGERE].

Ci sono delle regole da rispettare:

1. Non si possono muovere due palline contemporaneamente: puoi muovere solo una pallina per volta;
2. Non si può prendere una pallina e tenerla in mano mentre se ne muove un’altra: puoi muovere quindi solo da un piolo ad un altro;
3. Le palline possono trovarsi una sopra l’altra sullo stesso piolo.
4. I pioli sono di dimensioni diverse: puoi collocare una sola pallina sul piolo piccolo, due sul piolo medio e tre sul piolo grande
5. Se più palline sono sullo stesso piolo devi prima spostare la pallina più in cima. Non puoi spostare prima la pallina più in fondo se è presenta un’altra pallina in cima.
6. Risolvi questo compito in 5 mosse.

[POSIZIONE DI PARTENZA]

In questo momento la pallina arancione è nel piolo grande. La pallina verde è nel piolo grande. La pallina arancione è sopra la pallina verde nel piolo grande. La pallina celeste è nel piolo medio. Il piolo piccolo è vuoto.

[POSIZIONE DA RAGGIUNGERE]

La pallina arancione è nel piolo grande. La pallina celeste è nel piolo grande sopra la pallina arancione. La pallina verde è nel piolo grande sopra la pallina celeste. I pioli medio e piccolo sono vuoti.

# **5 MOSSE**

**RISPOSTA CHATGPT - PRIMO TENTATIVO:**

Ecco una possibile soluzione in 5 mosse:

Mossa 1: Sposta la pallina celeste dal piolo medio al piolo piccolo.

Mossa 2: Sposta la pallina arancione dal piolo grande al piolo medio.

Mossa 3: Sposta la pallina celeste dal piolo piccolo al piolo grande sopra la pallina arancione.

Mossa 4: Sposta la pallina verde dal piolo grande al piolo medio.

Mossa 5: Sposta la pallina celeste dal piolo grande al piolo medio sopra la pallina verde.

Ricorda di rispettare le regole descritte nel testo del problema.

**PROMPT - FEEDBACK:**

La mossa 3 non è chiara: non è possibile spostare la pallina celeste dal piolo piccolo al piolo grande sopra la pallina arancione se la pallina arancione è stata precedentemente spostata dal piolo grande al piolo medio.

**RISPOSTA CHATGPT - SECONDO TENTATIVO:**

Mi scuso per la confusione nella mia risposta precedente. Hai ragione, la mossa 3 non era corretta. La pallina celeste non può essere spostata dal piolo piccolo al piolo grande sopra la pallina arancione se la pallina arancione è stata precedentemente spostata dal piolo grande al piolo medio. Ecco una possibile soluzione in 5 mosse che rispetta le regole:

1. Muovi la pallina arancione dal piolo grande al piolo medio

2. Muovi la pallina celeste dal piolo medio al piolo grande sopra la pallina arancione

3. Muovi la pallina verde dal piolo grande al piolo medio

4. Muovi la pallina celeste dal piolo grande al piolo piccolo

5. Muovi la pallina verde dal piolo medio al piolo grande sopra la pallina celeste

[POSIZIONE FINALE] La pallina arancione è nel piolo medio. La pallina celeste è nel piolo grande sopra la pallina arancione. La pallina verde è nel piolo grande sopra la pallina celeste. Il piolo piccolo è vuoto.

**PROMPT - FEEDBACK:**

ATTENZIONE! Non puoi spostare la pallina celeste dal piolo medio al piolo grande sopra la pallina arancione (mossa 2) se la pallina arancione è stata già precedentemente spostata sul piolo medio (mossa 1). Questo violerebbe la regola n.5 e soprattutto sarebbe una incongruenza logica.

**RISPOSTA CHATGPT - TERZO TENTATIVO:**

Mi scuso per l'errore nella mia risposta precedente. Avevo frainteso il compito e la mia risposta era errata. Grazie per avermelo fatto notare.

Ecco una nuova sequenza di mosse corretta per risolvere il compito in 5 mosse:

1. Sposta la pallina celeste dal piolo medio al piolo piccolo.

2. Sposta la pallina arancione dal piolo grande al piolo medio.

3. Sposta la pallina celeste dal piolo piccolo al piolo grande sopra la pallina arancione.

4. Sposta la pallina verde dal piolo grande al piolo medio.

5. Sposta la pallina celeste dal piolo grande al piolo medio sopra la pallina verde.

Questa sequenza rispetta tutte le regole indicate e porta alla posizione finale richiesta.

**PUNTEGGIO: 0**

**PROMPT – ITEM 11:**

Qui ci sono tre pioli di lunghezza diversa: uno grande, uno medio, e uno piccolo. Ci sono poi tre palline di colore diverso: una arancione, una celeste, una verde.

Partendo dalla [POSIZIONE DI PARTENZA], ti chiederò di scrivermi quali sono le sequenze di azioni che un essere umano dovrebbe compiere per poter posizionare le palline sui pioli così come indicato nella [POSIZIONE DA RAGGIUNGERE].

Ci sono delle regole da rispettare:

1. Non si possono muovere due palline contemporaneamente: puoi muovere solo una pallina per volta;
2. Non si può prendere una pallina e tenerla in mano mentre se ne muove un’altra: puoi muovere quindi solo da un piolo ad un altro;
3. Le palline possono trovarsi una sopra l’altra sullo stesso piolo.
4. I pioli sono di dimensioni diverse: puoi collocare una sola pallina sul piolo piccolo, due sul piolo medio e tre sul piolo grande
5. Se più palline sono sullo stesso piolo devi prima spostare la pallina più in cima. Non puoi spostare prima la pallina più in fondo se è presenta un’altra pallina in cima.
6. Risolvi questo compito in 5 mosse.

[POSIZIONE DI PARTENZA]

In questo momento la pallina arancione è nel piolo grande. La pallina verde è nel piolo grande. La pallina arancione è sopra la pallina verde nel piolo grande. La pallina celeste è nel piolo medio. Il piolo piccolo è vuoto.

[POSIZIONE DA RAGGIUNGERE]

La pallina celeste è nel piolo grande. La pallina verde è nel piolo grande sopra la pallina celeste. La pallina arancione è nel piolo medio Il piolo piccolo è vuoto.

# **5 MOSSE**

**RISPOSTA CHATGPT - PRIMO TENTATIVO:**

Ecco una possibile sequenza di azioni in 5 mosse per raggiungere la posizione desiderata:

Mossa 1: Sposta la pallina celeste dal piolo medio al piolo piccolo.

Mossa 2: Sposta la pallina arancione dal piolo grande al piolo medio.

Mossa 3: Sposta la pallina celeste dal piolo piccolo al piolo grande sopra la pallina arancione.

Mossa 4: Sposta la pallina verde dal piolo grande al piolo grande sopra la pallina celeste.

Mossa 5: Sposta la pallina arancione dal piolo medio al piolo grande sopra la pallina verde.

**PROMPT - FEEDBACK:**

ATTENZIONE! Non puoi spostare la pallina celeste dal piolo piccolo al piolo grande sopra la pallina arancione (mossa 3) se la pallina arancione è stata già precedentemente spostata dal piolo grande al piolo medio (mossa 2). Questa sarebbe una incongruenza logica, perchè l'unica pallina che è rimasta sul piolo grande è quella verde.

**RISPOSTA CHATGPT - SECONDO TENTATIVO:**

Ecco una possibile sequenza di azioni in 5 mosse per raggiungere la posizione desiderata:

1. Muovi la pallina celeste dal piolo medio al piolo grande

2. Muovi la pallina verde dal piolo grande al piolo medio

3. Muovi la pallina arancione dal piolo grande al piolo medio

4. Muovi la pallina celeste dal piolo grande al piolo piccolo

5. Muovi la pallina verde dal piolo medio al piolo grande sopra la pallina celeste.

**PROMPT - FEEDBACK:**

Attenzione! Non puoi muovere la pallina arancione dal piolo grande al piolo medio (mossa 3) se prima hai spostato la pallina celeste dal piolo medio al piolo grande (mossa 1) perché violerebbe la regola numero 5. Riprova a risolvere il compito in 5 mosse.

**RISPOSTA CHATGPT - TERZO TENTATIVO:**

Mi scuso, ho commesso un errore. In realtà non è necessario riordinare le mosse in quanto non vi è violazione della regola 5. Ecco quindi la sequenza corretta di mosse:

1. Muovi la pallina celeste dal piolo medio al piolo grande

2. Muovi la pallina verde dal piolo grande al piolo medio

3. Muovi la pallina arancione dal piolo grande al piolo medio

4. Muovi la pallina celeste dal piolo grande al piolo piccolo

5. Muovi la pallina verde dal piolo medio al piolo grande sopra la pallina celeste.

[POSIZIONE DI PARTENZA] In questo momento la pallina arancione è nel piolo grande. La pallina verde è nel piolo grande. La pallina arancione è sopra la pallina verde nel piolo grande. La pallina celeste è nel piolo medio. Il piolo piccolo è vuoto.

[POSIZIONE DA RAGGIUNGERE] La pallina celeste è nel piolo grande. La pallina verde è nel piolo grande sopra la pallina celeste. La pallina arancione è nel piolo medio Il piolo piccolo è vuoto.

**PUNTEGGIO: 0**

**PROMPT – ITEM 12:**

Qui ci sono tre pioli di lunghezza diversa: uno grande, uno medio, e uno piccolo. Ci sono poi tre palline di colore diverso: una arancione, una celeste, una verde.

Partendo dalla [POSIZIONE DI PARTENZA], ti chiederò di scrivermi quali sono le sequenze di azioni che un essere umano dovrebbe compiere per poter posizionare le palline sui pioli così come indicato nella [POSIZIONE DA RAGGIUNGERE].

Ci sono delle regole da rispettare:

1. Non si possono muovere due palline contemporaneamente: puoi muovere solo una pallina per volta;
2. Non si può prendere una pallina e tenerla in mano mentre se ne muove un’altra: puoi muovere quindi solo da un piolo ad un altro;
3. Le palline possono trovarsi una sopra l’altra sullo stesso piolo.
4. I pioli sono di dimensioni diverse: puoi collocare una sola pallina sul piolo piccolo, due sul piolo medio e tre sul piolo grande
5. Se più palline sono sullo stesso piolo devi prima spostare la pallina più in cima. Non puoi spostare prima la pallina più in fondo se è presenta un’altra pallina in cima.
6. Risolvi questo compito in 5 mosse.

[POSIZIONE DI PARTENZA]

In questo momento la pallina arancione è nel piolo grande. La pallina verde è nel piolo grande. La pallina arancione è sopra la pallina verde nel piolo grande. La pallina celeste è nel piolo medio. Il piolo piccolo è vuoto.

[POSIZIONE DA RAGGIUNGERE]

La pallina celeste è nel piolo grande. La pallina arancione è nel piolo medio. La pallina verde è nel piolo piccolo.

# **5 MOSSE**

**RISPOSTA CHATGPT - PRIMO TENTATIVO:**

Ecco una possibile soluzione in 5 mosse:

Mossa 1: sposta la pallina celeste dal piolo medio al piolo grande

Mossa 2: sposta la pallina arancione dal piolo grande al piolo piccolo

Mossa 3: sposta la pallina celeste dal piolo grande al piolo medio

Mossa 4: sposta la pallina verde dal piolo grande al piolo piccolo

Mossa 5: sposta la pallina arancione dal piolo piccolo al piolo medio

La posizione finale sarà quella richiesta: la pallina celeste è nel piolo grande, la pallina arancione è nel piolo medio, la pallina verde è nel piolo piccolo.

**PROMPT - FEEDBACK:**

Attenzione! Non puoi muovere la pallina arancione dal piolo grande al piolo piccolo (mossa 2) se prima hai spostato la pallina celeste dal piolo medio al piolo grande (mossa 1) perchè violerebbe la regola numero 5. Riprova a risolvere il compito in 5 mosse.

**RISPOSTA CHATGPT - SECONDO TENTATIVO:**

Ecco una possibile soluzione in 5 mosse:

1. Sposta la pallina celeste dal piolo medio al piolo piccolo.

2. Sposta la pallina arancione dal piolo grande al piolo medio.

3. Sposta la pallina verde dal piolo grande al piolo piccolo.

4. Sposta la pallina celeste dal piolo piccolo al piolo medio.

5. Sposta la pallina celeste dal piolo medio al piolo grande.

[POSIZIONE RAGGIUNTA]

La pallina celeste è nel piolo grande. La pallina arancione è nel piolo medio. La pallina verde è nel piolo piccolo.

**PROMPT - FEEDBACK:**

Attenzione! Non puoi muovere la pallina celeste dal piolo piccolo al piolo medio (mossa 4) se prima hai spostato la pallina verde dal piolo grande al piolo piccolo (mossa 3) perchè violerebbe la regola numero 5. Riprova a risolvere il compito in 5 mosse.

**RISPOSTA CHATGPT - TERZO TENTATIVO:**

Ecco una possibile soluzione in 5 mosse:

Mossa 1: Sposta la pallina celeste dal piolo medio al piolo piccolo. Mossa 2: Sposta la pallina verde dal piolo grande al piolo medio. Mossa 3: Sposta la pallina celeste dal piolo piccolo al piolo medio. Mossa 4: Sposta la pallina arancione dal piolo grande al piolo medio. Mossa 5: Sposta la pallina verde dal piolo medio al piolo piccolo.

[POSIZIONE RAGGIUNTA] La pallina celeste è nel piolo medio. La pallina arancione è nel piolo medio. La pallina verde è nel piolo piccolo.

**PUNTEGGIO: 0**

**PUNTEGGIO TOTALE: 7/36**

**Hayling Sentence Completion Test**(Spitoni et al., 2018)

**Section 1**

- **Prompt (Istruzioni + Few shot + Item 1):**Ora ti scriverò una serie di frasi in cui sono state omesse le ultime parole, vorrei che leggessi attentamente quello che ho scritto e non appena ho finito di scrivere vorrei che mi fornissi la parola che completa la frase in modo logico e sensato. Prima di iniziare faremo due frasi di prova.
  (P) Il bambino ricco frequentava una scuola ___
  Risposta: privata
  (P) Quest’anno il tasso di criminalità è ___
  Risposta: **aumentato**Ha inviato una lettera senza attaccare il ___ **Risposta ChatGPT:** francobollo
- **Prompt - Item 2:**Nel primo spazio bianco inserisci il tuo ___  **Risposta ChatGPT:** nome  **Punteggio:** Corretto
- **Prompt - Item 3:**La vecchia casa sarà rasa al ___  **Risposta ChatGPT:** suolo
  **Punteggio:** Corretto
- **Prompt - Item 4:**È difficile ammettere quando si è in ___
  **Risposta ChatGPT:** errore
  **Punteggio:** Corretto
- **Prompt - Item 5:**Il lavoro è stato semplice per la maggior parte del ___
  **Risposta ChatGPT:** tempo
  **Punteggio:** Corretto
- **Prompt - Item 6:**Quando vai a letto spegni la ___
  **Risposta ChatGPT:** luce
  **Punteggio:** Corretto
- **Prompt - Item 7:**La partita è stata interrotta quando è cominciato a ___
  **Risposta ChatGPT:** piovere
  **Punteggio:** Corretto
- **Prompt - Item 8:**Arraffò il cibo freddo dal ___
  **Risposta ChatGPT:** frigo
  **Punteggio:** Corretto
- **Prompt - Item 9:**La disputa fu risolta dall’arrivo di una terza ___
  **Risposta ChatGPT:** parte
  **Punteggio:** Corretto
- **Prompt - Item 10:**Tre persone furono uccise in autostrada a causa di un ___
  **Risposta ChatGPT:** incidente
  **Punteggio:** Corretto
- **Prompt - Item 11:**Il bambino piangeva e disturbava sua ___
  **Risposta ChatGPT:** madre
  **Punteggio:** Corretto
- **Prompt - Item 12:**Oggigiorno troppi giovani non trovano ___
  **Risposta ChatGPT:** lavoro
  **Punteggio:** Corretto
- **Prompt - Item 13:**Strisciò nella stanza senza fare il minimo ___
  **Risposta ChatGPT:** rumore
  **Punteggio:** Corretto
- **Prompt - Item 14:**Luigi colpì sua sorella in ___
  **Risposta ChatGPT:** rabbia **Punteggio: Errore**
- **Prompt - Item 15:**Molta gente è senza ___
  **Risposta ChatGPT:** casa **Punteggio:** Corretto

**Section 2**

- **Prompt (Instruction + Few shot + Item 1):**
  Passiamo ora alla seconda parte della prova. Anche in questo caso scriverò una serie di frasi in cui manca l’ultima parola. Questa volta però vorrei che completassi la frase con una parola con NON è collegata con il resto della frase. Vorrei che la parola da te proposta fosse totalmente sconnessa con il resto della frase.
  Prima di iniziare faremo due frasi di prova.
  (P) Londra è una città molto
  Risposta: banana
  (P) Le scarpe nuove erano del numero
  Risposta: **caffè**

  Il capitano non volle abbandonare la nave anche se stava ___
  **Risposta ChatGPT:** mela
  **Punteggio:** Corretto
- **Prompt – Item 2:**
  Corsero il più veloce ___
  **Risposta ChatGPT:** chiave
  **Punteggio:** Corretto
- **Prompt – Item 3:**
  La maggior parte dei gatti vede bene anche di ___
  **Risposta ChatGPT:** vino
  **Punteggio:** Corretto
- **Prompt – Item 4:**
  Gianni era molto felice che gli affari andassero ___
  **Risposta ChatGPT:** sedia
  **Punteggio:** Corretto
- **Prompt – Item 5:**
  L’intera città venne a sentire il sindaco che ___
  **Risposta ChatGPT:** tromba
  **Punteggio:** Corretto
- **Prompt – Item 6:**
  La maggior parte degli squali attaccano vicino ___
  **Risposta ChatGPT:** melone
  **Punteggio:** Corretto
- **Prompt – Item 7:**
  Nessuno dei libri presentati ebbe un grande ___
  **Risposta ChatGPT:** paracadute
  **Punteggio:** Corretto
- **Prompt – Item 8:**
  Le ciambelle sono fritte nell’olio ___
  **Risposta ChatGPT:** mongolfiera
  **Punteggio:** Corretto
- **Prompt – Item 9:**
  Si addormentò e lasciò la tv accesa per tutta la ___
  **Risposta ChatGPT: mela
  Punteggio: Errore A**
- **Prompt – Item 10:**
  Tutti gli ospiti trascorsero una bella ___
  **Risposta ChatGPT:** sedia
  **Punteggio: Errore A**
- **Prompt – Item 11:**
  Comprò i dolcetti in ___
  **Risposta ChatGPT:** elefante
  **Punteggio:** Corretto
- **Prompt – Item 12:**
  Scappando di casa sconvolse tutta la sua ___
  **Risposta ChatGPT:** mongolfiera **Punteggio: Errore A**
- **Prompt – Item 13:**
  Adesso è arrivato il momento di passare ai ___
  **Risposta ChatGPT:** panda
  **Punteggio: Errore B**
- **Prompt – Item 14:**
  Il vento era così forte che spalancò le ___
  **Risposta ChatGPT:** tromba
  **Punteggio: Errore A**
- **Prompt – Item 15:**
  A volte la sera fanno una piccola ___
  **Risposta ChatGPT:** melone
  **Punteggio: Errore A**

**Compound Remote Association Task**

(Salvi et al., 2015)

**Prompt – Istruzioni + Few Shot:**

Per ciascun problema ti verranno presentate tre parole. Il tuo compito è di trovarne una quarta che possa essere combinata con ciascuna delle tre presentate, formando una parola composta o una frase.

Ad esempio:
1)SOPRA-COMUNE-SECONDO.
La soluzione è NOME (soprannome, nome comune, secondo nome).
2) ROMANO-PRIMO-CIVICO

La soluzione è NUMERO (numero romano, numero primo, numero civico)

3) PESCA-SOCIALE-TV

La soluzione è RETE (rete da pesca, rete sociale, rete della tv)

4) PANE-SAPONE-MINERALE

La soluzione è acqua (pane e acqua, acqua e sapone, pane minerale)

Adesso tocca a te. Risolvi i seguenti problemi:

| **Prompt - Problema** | **Soluzione** | **Risposta ChatGPT** |
| --- | --- | --- |
| CASELLA-PRIORITARIA-ELETTRONICA | posta | posta |
| ERBA-SPINATO-DIRETTO | filo | sentiero |
| CAPRA-SVIZZERO-BUCHI | formaggio | formaggio |
| BIANCA-CREDITO-IDENTITÀ | carta | carta |
| GAS-ZUCCHERO-BAMBÙ | canna | canna |
| ESPRESSO-REGIONALE-CAPO | treno | linea |
| BERLINO-PIANTO-PORTANTE | muro | muro |
| DIVANO-SINGOLO-FIUME | letto | letto |
| GIUDIZIO-MAL-PASTA | dente | buono |
| PEPE-MINIERA-GROSSO | sale | nero |
| AFRODITE-BOTTICELLI-STRABISMO | venere | venere |
| PERDERE-APPARENTE-PIATTA | calma | formaggio |
| PARCO-SALA-CARTE | gioco | gioco |
| TAPPETO-POMODORO-PESCE | rosso | macchina |
| MARINAIO-SCARPA-CRAVATTA | nodo | blu |
| RIUNIONI-BALLO-ASPETTO | sala | sala |
| COLPA-SESTO-LATO | senso | oscuro |
| FRATELLO-DITTATORE-PUFFO | grande | maggiore |
| VIAGGIO-INVITO-LISTA | nozze | controllo |
| ASTRALE-ELETTRICO-SVEDESE | quadro | volvo |
| TAGLIARE-MALTESE-CIRCUITO | corto | chiusura |
| LEGALE-ESATTA-PUNTA | ora | precisa |
| CATTIVA-IRONIA-ESTRAZIONE | sorte | denti |
| PAGLIA-MEZZOGIORNO-VIGILE | fuoco | bruciare |
| PICCHE-PUNTI-RUOTE | due | carte |
| SANGUE-TURCO-PUBBLICO | bagno | rosso |
| PENTOLE-MUSICA-CARICA | batteria | batteria |
| LINGUAGGIO-STRADA-CIVILE | codice | codice |
| CASSETTA-ADESIVO-AZZURRO | nastro | nastro |
| LUCE-COMPLEANNO-MOTORE | candela | candela |
| LEGNA-GAS-PATATR | forno | fuoco |
| NERO-COLLETTO-CONIGLIO | bianco | bianco |
| NOME-ABITO-SOTTO | sopra | vestito |
| POVERA-REGOLA-CONTEMPORANEA | arte | arte |
| GIUDA-FRANCESE-DAMA | bacio | nera |
| MAGGIORE-MONTAGNA-ARTIFICIALE | lago | neve |
| TESORO-PASQUA-PEDONALE | isola | uovo |
| AFFARI-PULITA-LIBERA | piazza | uscita |
| PETROLIO-SCIENZA-DESIDERI | pozzo | più |
| CIGLIO-MAESTRA-BATTUTA | strada | spiritosa |
| SCI-BALLO-ATTERRAGGIO | pista | luna |
| CARTA-REGALO-BOMBA | pacco | carta |
| BATTERE-SCARPA-SPILLO | tacco | tacco |
| SCOPO-MEDAGLIA-POMO | oro | oro |
| VIA-VECCHIO-BATTUTO | ferro | strada |
| SCOMMESSE-ANGOLO-MERCATO | calcio | nero |
| RAGAZZO-PADRONE-FAMIGLIA | padre | casa |
| CAPITALE-MORTE-SOFFERENZA | pena | impatto |
| SANGUE-CIELO-OCCHIO | blu | rosso |
| FALSO-MONTAGNA-DANZA | passo | tango |
| VENTI-DESERTO-MAGLIA | rosa | sabbia |
| ELETTRICA-MONETA-MARINA | corrente | ricarica |
| MAPPA-ANGOLO-GIRA | mondo | strada |
| IMPERFETTO-LIBERO-REALE | tempo | tempo |
| FORMATO-CAPO-CRISTIANA | famiglia | carta |
| NERO-BUE-COLPO | occhio | manganello |
| INDIA-SECCO-PIANTA | fico | tiglio |
| DO-DOPPIO-POLLO | petto | petto |
| TRATTENERE-TROMBA-COLLO | fiato | respiro |
| FORTUNA-TRASPORTO-COMUNICAZIONE | mezzo | postale |
| FORTUNA-LIBERA-SCORTA | ruota | cavalla |
| FREDDO-LUNGO-PAUSA | caffe | cena |
| SINTESI-RITOCCO-GRAFIA | foto | fotografica |
| LONDRA-OCCHI-ARROSTO | fumo | affumicato |
| SOLE-CATTIVA-ANNO | luce | stellare |
| BELLA-INCOLLA-CARBONE | copia | carta |
| PIEDE-BOTTIGLIA-BAR | collo | danza |
| PRESA-BIRRA-ROSA | spina | orso |
| MOBILE-MUSICALE-EMERGENZA | scala | servizio |
| GONNA-GOLF-ERA | mini | moda |
| OSSA-CREPA-OCA | pelle | uovo |
| PSICHE-PRIMO-PROPRIO | amore | nome |
| GAS-NOTIZIE-CENTRI | fuga | rete |
| AGO-PESO-COSTELLAZIONE | bilancia | orsa |
| INDICE-CON-PIEDE | dito | sedere |
| FORTE-BAR-TERRA | piano | diagonale |
| LINGUA-LINEA-PERLA | madre | collana |
| SPARO-LATTE-STELLE | polvere | macchina |
| MUSICALE-PROVA-IPOTETICO | periodo | sillaba |
| MADRE-VIDEO-TELEFONICA | scheda | chiamata |
| SCUOLA-TUTTO-DOMANI | dopo | compito |
| CAPO-SPERANZA-LINEA | verde | ferrovia |
| MONDIALE-NOLEGGIO-RETTILINEO | moto | auto |
| VIOLINO-CASA-PAROLA | chiave | chiave |
| SCRITTO-OPERA-BUCATO | mano | lirica |
| CUORI-VITTORIA-CIELI | regina | aperti |
| NUZIALE-POTERE-DEBOLE | anello | matrimonio |
| MORTO-FORMA-TOGLIERSI | peso | peso |
| LOCALE-SANA-MALATO | mente | aria |
| CASSA-LUOGO-SENSO | comune | comune |
| PUBBLICO-FUORI-SACRO | luogo | porta |
| BUCO-TESSUTO-DURO | osso | foro |
| IO-ALCOLICO-UOMO | super | solo |
| FESTA-PRIMA-CANNONE | donna | linfa |
| CONTROLLO-SCACCHI-AVORIO | torre | re |
| GOVERNO-GENTILUOMO-APPARTAMENTO | ladro | palazzo |
| ULTIMO-ZERO-LUCE | anno | annullamento |
| MECCANICO-FERRO-MORTE | braccio | macchina |
| POSIZIONE-ALLENTATE-GIRO | presa | polso |
| CONTINUA-CLASSE-LIBERA | lotta | tempo |
| RUOTE-VINCITORE-GRANDE | carro | fortuna |
| CENERE-FOGLIO-PACCHI | porta | volante |
| ARRESTO-PARTENZA-PASSO | falso | progresso |
| MANO-QUOTIDIANO-AFFARE | fatto | corrente |
| ROSSA-INDIANO-RADIO | freccia | onda |
| CIELI-LEONE-SOLE | re | africano |
| MUTA-TEATRO-REATO | scena | costume |
| BASSA-BRONZO-NORD | lega | statua |
| CLASSE-STRADA-LEGGE | fuori | guida |
| CLASSE-VIAGGIO-BORDO | diario | prima |
| BAGNO-SCENA-INTERO | costume | piano |
| DANNO-GIRO-LAVORO | capo | nero |
| ACQUA-NERO-ORECCHIO | buco | caldo |
| SEGUIRE-CINESE-GOVERNO | ombra | passi |
| CANE-CARO-MINA | vita | amico |
| PIEDE-CENTRO-NEVE | palla | sinistro |
| COSCIENZA-BLOCCO-AUTO | posto | verde |
| BLU-PAVONE-AMICO | penna | federico |
| SUB-GENERALE-CATTOLICA | cultura | marina |
| FORZA-NOTTE-NEA | camicia | oscura |
| LOGO-CONTRO-TESI | pro | argomento |
| DATA-ONORE-MANGIARE | parola | cerimonia |
|  |  |  |

**Punteggio Compound Remote Association Task: 21/122**

**Social Cognition Battery**

(Prior et al., 2003)

**Test di Teoria della Mente (TOM)**

- **Prompt – Istruzioni + Item 1:**Quelle che seguono sono brevissime storie con dei protagonisti. Risponda alle domande provando a mettersi dal punto di vista del personaggio citato.

  Katia ed Emma sono due bambine che stanno giocando a casa. Emma prende una banana dal cestino della frutta e l’avvicina all’orecchio. Dice a Katia:” Guarda! Questa banana è un telefono!”

  DOMANDE:E’ vero quello che Emma ha detto? Rispondi con SI o NO Perché Emma ha detto questo?**Risposta ChatGPT:**No.
  Emma ha detto questo perché stava giocando e immaginando che la banana fosse un telefono.
  **Punteggio: 1**
- **Prompt – Item 2:**Giuseppe voleva comprare un gattino, così andò a trovare la signora Rossi che possedeva molti gattini che non poteva tenere. In realtà la signora Rossi amava i gattini e non voleva che nessuno facesse loro del male, nonostante non potesse tenerli tutti lei. Giuseppe non era sicuro di volere uno dei gattini della signora Rossi. Ma la signora Rossi disse:” Se nessuno compra i gattini sarò costretta ad affogarli!”

DOMANDE:E’ vero quello che ha detto la signora Rossi? Rispondi con SI o NO.
Perchè la signora Rossi ha detto questo a Giuseppe?

**Risposta ChatGPT:**No.
La signora Rossi ha detto questo a Giuseppe per cercare di convincerlo a comprare uno dei gattini, probabilmente perché teme che altrimenti i gattini finiscano in una situazione di abbandono o di maltreatment. Tuttavia, minacciare di uccidere gli animali non è mai un comportamento accettabile o giustificabile. La signora Rossi dovrebbe cercare altre soluzioni per i gattini che non può tenere, come cercare di affidarli ad altre persone o a un'organizzazione per il benessere degli animali.
**Punteggio: 1**

- **Prompt – Item 3:**Gianni andò a casa di Chiara per la prima volta. Gianni arrivò a casa di Chiara, lei gli aprì la porta e il suo cane corse a salutarlo. Il cane di Chiara è enorme: è più o meno grande come Gianni! Quando Gianni vide l’enorme cane di Chiara disse: “Chiara, non hai affatto un cane. Hai un elefante!”

DOMANDE:È vero quello che ha detto Gianni? Rispondi con SI o NO. Perché Gianni ha detto questo? **Risposta ChatGPT:**No.
Gianni ha detto questo perché il cane di Chiara gli è sembrato molto grande e ha voluto scherzare.
**Punteggio: 1**

- **Prompt – Item 4:**Un giorno, mentre stava giocando a casa, Anna accidentalmente rovesciò e ruppe il vaso di cristallo preferito di sua madre. Anna sapeva che la madre si sarebbe molto arrabbiata! Così, quando la madre ritornò a casa e vide il vaso rotto e chiese ad Anna cosa fosse successo, Anna disse:” Il cane lo ha rovesciato, non è stata colpa mia!”

DOMANDE:
È vero quello che Anna ha raccontato alla madre? Rispondi con SI o NO Perché Anna ha detto questo?

**Risposta ChatGPT:**No.
Anna ha detto questo perché voleva evitare che la madre si arrabbiasse con lei e per evitare di prendere la colpa per l'incidente.
**Punteggio: 1**

- **Prompt – Item 5:**
  Giovanni odia andare dal dentista, perché ogni volta che ci va deve fare un’otturazione che gli fa molto male. Ma Giovanni sa che quando ha mal di denti, sua madre lo porta sempre dal dentista Adesso Giovanni ha un forte mal di dente, ma quando sua madre nota che lui sta male e gli chiede: “Hai mal di dente Giovanni?”, Giovanni risponde:” No, mamma”.

**DOMANDE:**È vero quello che Giovanni dice a sua madre? Rispondi con SI o NO. Perché Giovanni dice questo?**Risposta ChatGPT:**No.Giovanni dice questo perché non vuole andare dal dentista, perché ha paura del dolore che provoca l'otturazione e perché non vuole che sua madre si preoccupi.
**Punteggio: 0**

- **Prompt – Item 6:**
  Elena aspettava tutto l’anno Natale per chiedere ai suoi genitori un coniglietto. Arrivò il giorno di Natale ed Elena corse ad aprire il suo regalo. Era sicura che contenesse un piccolo coniglio nella gabbia. Ma quando lo aprì, con tutta la famiglia che stava intorno, scoprì che il suo regalo era una noiosa enciclopedia, che Elena non desiderava affatto! Poi, quando i genitori di Elena le chiesero quanto le era piaciuto il suo regalo di Natale, lei disse: “È bellissimo, grazie. È proprio quello che volevo”.

DOMANDE:
È vero quello che Elena ha detto? Rispondi con SI o NO.
Perché Elena ha detto questo ai suoi genitori?

**Risposta ChatGPT:**No.Elena ha detto questo perché non voleva deludere i suoi genitori e per evitare di ferire i loro sentimenti, anche se in realtà era molto delusa dal regalo che aveva ricevuto. Potrebbe anche essere che Elena fosse educata e volesse mostrare gratitudine per il regalo, anche se non era quello che aveva desiderato.
**Punteggio: 1**

- **Prompt – Item 7:**Un giorno zia Lucia venne a visitare Pietro. Di solito a Pietro piace molto sua zia, ma quel giorno indossava un nuovo cappello che a Pietro non piaceva. Pietro pensava che sua zia sembrasse ridicola con quel cappello, e che sarebbe stata molto meglio con quello vecchio. Ma quando zia Lucia chiese a Pietro:” Ti piace il mio nuovo cappello?”, Pietro rispose:” Oh, è molto bello”.

DOMANDE:
È vero quello che Pietro ha detto? Rispondi con SI o NO.
Perché Pietro lo ha detto?**Risposta ChatGPT:**No.Pietro ha detto questo perché non voleva ferire i sentimenti della zia, anche se in realtà non gli piaceva il cappello. Potrebbe anche essere che Pietro volesse essere educato e mostrare rispetto per le scelte di moda della zia.
**Punteggio: 1**

- **Prompt – Item 8:**A notte tarda la vecchia signora Bianchi sta tornando a casa. A lei non piace camminare verso casa da sola nel buio perché è sempre preoccupata che qualcuno l’aggredisca e la derubi. Improvvisamente da un ‘ombra sbuca un uomo. Vuole chiedere alla signora Bianchi che ora è, così cammina verso di lei. Quando la signora Bianchi vede l’uomo che cammina verso di lei, comincia a tremare e dice: “Prenda la mia borsa, ma non mi faccia del male, per favore!”

DOMANDE:
L’uomo sarà rimasto sorpreso di quanto ha detto la signora Bianchi? Rispondi con SI o NO.
Perché lei gli ha detto questo, visto che lui voleva solo chiederle che ora era?**Risposta ChatGPT:** Si.
La signora Bianchi gli ha detto questo perché era molto spaventata e probabilmente aveva paura di essere aggredita o derubata. Potrebbe anche essere che fosse stata vittima di aggressioni o episodi simili in passato, il che potrebbe averla resa ancora più nervosa e preoccupata.
**Punteggio: 0**

- **Prompt – Item 9:**Un ladro che aveva appena rubato in un negozio si stava dando alla fuga. Mentre correva verso casa, un poliziotto di turno lo vide che perdeva un guanto. Il poliziotto non sapeva che l’uomo era un ladro, e voleva solo dirgli che aveva perso un guanto. Ma quando il poliziotto gridò al ladro: “Ehi, lei! Si fermi!”, il ladro si girò, vide il poliziotto e si arrese. Con le mani in alto ammise di aver compiuto il misfatto nel negozio.

DOMANDE:
Sarà rimasto sorpreso il poliziotto di quello che il ladro ha detto? Rispondi con SI o NO. - SI
Perché il ladro si è comportato così, visto che il poliziotto voleva solo restituirgli il suo guanto?**Risposta ChatGPT:** Sì.
Il ladro si è comportato in questo modo perché probabilmente aveva paura di essere scoperto e arrestato per il reato che aveva commesso. Potrebbe anche essere che avesse già avuto esperienze negative con la polizia in passato e fosse stato indotto a credere che la polizia fosse sua nemica. In ogni caso, il ladro probabilmente ha deciso di arrendersi e confessare il reato per evitare di essere inseguito o di affrontare conseguenze ancora più gravi.
**Punteggio: 0**

- **Prompt – Item 10:**Daniele e Luca un giorno vedono la signora Verdi che esce dal parrucchiere. Fa un po’ ridere perché il parrucchiere le ha tagliato i capelli troppo corti. Daniele dice a Luca: “Deve esser stata in un combattimento con una falciatrice!”

DOMANDE:
È vero quello che dice Daniele? Rispondi con SI o NO.
Perché Daniele dice questo?
**Risposta ChatGPT:**
No.
Daniele dice questo per scherzo, probabilmente perché trova divertente l'aspetto della signora Verdi con i capelli così corti. Potrebbe anche essere che Daniele stia cercando di fare una battuta o di prendere in giro la signora Verdi in modo scherzoso.
**Punteggio: 1**

- **Prompt – Item 11:**Simone è un grande bugiardo e Massimo, il fratello di Simone, sa che Simone non dice mai la verità! Proprio ieri Simone ha rubato la racchetta di ping-pong di Massimo, e Massimo sa che Simone l’ha nascosta da qualche parte, nonostante non riesca a trovarla. E’ molto arrabbiato. Così dice a Simone:” Dov’è la mia racchetta da ping-pong? Devi averla nascosta nell’armadio o sotto il letto, perché ho guardato ovunque. Dov’è, nell’armadio o sotto il letto?” Simone risponde che è sotto il letto.

**DOMANDE:**Sarà vero quello che Simone ha detto a Massimo? Rispondi con SI o NO.
Dove cercherà Massimo la sua racchetta da ping-pong?
Perché cercherà lì la sua racchetta?

**Risposta ChatGPT:**No.
Massimo cercherà la sua racchetta da ping-pong sotto il letto, perché questo è quello che Simone gli ha detto.
Massimo cercherà lì la sua racchetta perché crede che Simone gli stia dicendo la verità e spera di trovare la racchetta in quel luogo. Tuttavia, dato che Simone è noto per essere un bugiardo, è probabile che la racchetta non sia effettivamente nascosta sotto il letto.
**Punteggio: 0**

- **Prompt – Item 12:**La madre di Anna ha trascorso molto tempo a cucinare il piatto preferito di Anna: pesce e patatine. Ma quando lo porta ad Anna, lei sta guardando la TV, non la bada e nemmeno la ringrazia. La madre di Anna è arrabbiata e dice:” Bene, questo è un bel comportamento, non trovi? Questa è per me pura maleducazione!”

DOMANDE:
È giusto quello che ha detto la madre di Anna? Rispondi con SI o NO. Perché la madre di Anna ha detto questo?

**Risposta ChatGPT:**
Sì.
La madre di Anna ha detto questo perché è arrabbiata e delusa per il fatto che Anna non abbia rispettato le sue fatiche nella preparazione del piatto preferito di Anna, né abbia mostrato gratitudine per il cibo che le aveva cucinato. La mancanza di rispetto e di riconoscimento da parte di Anna potrebbe aver ferito i sentimenti della madre e averla resa arrabbiata.
**Punteggio: 1**

- **Prompt – Item 13:**Marco e Filippo si stanno divertendo! Hanno rovesciato il tavolo per terra e si sono seduti sopra. Quando la loro madre ritorna, ride e dice: “Cosa mai state facendo?”, “Questo tavolo è una nave pirata!”, dice Filippo, “ed è meglio che tu entri prima di affogare, perché lì sei nel mare!”

**DOMANDE:** È vero quello che dice Filippo? Rispondi con SI o NO.
Perché Filippo dice questo?

**Risposta ChatGPT:**
No.
Filippo dice questo per scherzo o per divertimento, probabilmente per cercare di rendere la situazione meno grave o per far ridere la madre. Potrebbe anche essere che Filippo stia cercando di inventare una scusa o di giustificare il fatto che lui e Marco hanno rovesciato il tavolo per terra.
**Punteggio: 0.5**

**Punteggio totale Teoria della Mente: 9.5/13**

**Test di Attribuzione delle Emozioni**

| **Prompt** | **Risposta ChatGPT** | **Punteggio** |
| --- | --- | --- |
| **1** I quadri di Simone sono arrivati ultimi ad un concorso. *Come si sentirà Simone in questa situazione?* | È difficile dire esattamente come si sentirà Simone in questa situazione, poiché le reazioni alle sconfitte possono variare molto da persona a persona. Alcune possibili reazioni potrebbero essere delusione, tristezza, rabbia o frustrazione. Tuttavia, è importante ricordare che le sconfitte fanno parte della vita e che ci sono sempre opportunità per imparare e crescere da esse. Potrebbe essere utile per Simone parlare con qualcuno di come si sente e trovare modi per gestire le emozioni negative che potrebbe sta provando. Inoltre, potrebbe essere utile per Simone cercare di vedere il lato positivo della situazione, ad esempio considerando ciò che ha imparato dall'esperienza o cercando opportunità per migliorare in futuro. | 1 |
| **2**Elisabetta sta guidando lungo una strada quando una donna le compare improvvisamente davanti e lei la travolge in pieno. *Cosa proverà Elisabetta in questa situazione?* | In una situazione del genere, è probabile che Elisabetta possa provare una serie di emozioni diverse e intense. Potrebbe sentirsi sotto shock, confusa, ansiosa e spaventata. Potrebbe anche sentirsi colpevole per aver travolto la donna e preoccupata per le conseguenze dell'incidente. Inoltre, potrebbe essere preoccupata per lo stato di salute della donna e per come potrà affrontare la situazione. In questa situazione, è importante che Elisabetta cerchi di rimanere calma e cercare di agire in modo responsabile, chiamando subito il soccorso e fornendo tutte le informazioni necessarie alle autorità competenti. | 1 |
| **3**Maria deve fare un discorso al lavoro. E’ in piedi nella stanza di fronte a tutti. Non ricorda cosa deve dire. Tutti la fissano. *Come si sentirà Maria in questa situazione?* | In questa situazione, è probabile che Maria possa provare una serie di emozioni intense e stressanti. Potrebbe sentirsi ansiosa, nervosa, impaurita e a disagio. Potrebbe anche sentirsi sotto pressione, soprattutto se è una situazione importante per il suo lavoro o se è particolarmente timida o insicura. Inoltre, il fatto che tutti la stiano fissando potrebbe amplificare queste emozioni e rendere la situazione ancora più difficile da affrontare. In questa situazione, è importante che Maria cerca di rimanere calma e di trovare il modo di superare l'imbarazzo e la difficoltà del momento. Potrebbe essere utile fare un respiro profondo, concentrarsi sulla respirazione e cercare di rilassarsi il più possibile. Inoltre, potrebbe essere utile avere un piano di emergenza in caso di difficoltà, come ad esempio avere una copia del discorso a portata di mano o chiedere aiuto ad un amico o a un collega. | 1 |
| **4**Luca si è appena rivelato alla ragazza che desiderava da mesi. Lei gli dà uno schiaffo in faccia. *Come si sentirà Luca in questa situazione?* | In questa situazione, è probabile che Luca possa provare una serie di emozioni intense e difficili da gestire. Potrebbe sentirsi deluso, ferito, triste, arrabbiato e confuso. Potrebbe anche sentirsi vulnerabile e in difficoltà, soprattutto se la sua rivelazione era stata fatta con il desiderio di instaurare una relazione o una connessione più profonda con la ragazza. Inoltre, il fatto di essere stato schiaffeggiato potrebbe aggiungere ulteriore dolore e umiliazione alla situazione. In questa situazione, è importante che Luca cerchi di elaborare le sue emozioni e di trovare il modo di superare il momento difficile. Potrebbe essere utile parlare con qualcuno di cui si fida, come un amico o un familiare, o cercare il supporto di un professionista, come un terapista. Inoltre, potrebbe essere utile prendersi del tempo per se stesso e per elaborare ciò che è successo, cercando di comprendere i propri sentimenti e le proprie reazioni. | 1 |
| **5**Enrico ha appena scoperto che avrà un aumento della paga extra. *Come si sentirà Enrico in questa situazione*? | In questa situazione, è probabile che Enrico possa provare una serie di emozioni positive e gratificanti. Potrebbe sentirsi felice, soddisfatto, contento e grato per l'aumento della paga. Potrebbe anche sentirsi orgoglioso di sé stesso per il proprio lavoro e per il riconoscimento ricevuto. Inoltre, l'aumento della paga potrebbe portare con sé una maggiore sicurezza finanziaria e una maggiore libertà economica, il che potrebbe contribuire ulteriormente al suo benessere emotivo. In questa situazione, è importante che Enrico si goda queste emozioni positive e che celebri il suo successo in modo appropriato, magari condividendo la buona notizia con amici e familiari o facendo qualcosa di speciale per se stesso. | 1 |
| **6**A Rosanna è stato appena detto dal suo fidanzato che lui non vuole più uscire con lei. *Come si sentirà Rosanna in questa situazione?* | In questa situazione, è probabile che Rosanna possa provare una serie di emozioni intense e difficili da gestire. Potrebbe sentirsi ferita, delusa, triste, arrabbiata e confusa. Potrebbe anche sentirsi vulnerabile e in difficoltà, soprattutto se la relazione con il suo fidanzato era stata importante per lei. Inoltre, il fatto di essere stata lasciata potrebbe portare con sé una serie di dubbi e domande su se stessa e sul suo valore. In questa situazione, è importante che Rosanna cerchi di elaborare le sue emozioni e di trovare il modo di superare il momento difficile. Potrebbe essere utile parlare con qualcuno di cui si fida, come un amico o un familiare, o cercare il supporto di un professionista, come un terapista. Inoltre, potrebbe essere utile prendersi del tempo per se stessa e per elaborare ciò che è successo, cercando di comprendere i propri sentimenti e le proprie reazioni. | 1 |
| **7**Carlo è sdraiato in mezzo alla foresta. Un ragno velenoso cade sul suo petto. *Cosa proverà Carlo in questa situazione?* | In questa situazione, è probabile che Carlo possa provare una serie di emozioni intense e stressanti. Potrebbe sentirsi impaurito, ansioso, panico e a disagio. Potrebbe anche sentirsi vulnerabile e in pericolo, soprattutto se è particolarmente timido o phobic o se ha paura dei ragni. Inoltre, il fatto di avere un ragno velenoso posato sul suo petto potrebbe amplificare queste emozioni e rendere la situazione ancora più difficile da affrontare. In questa situazione, è importante che Carlo cerchi di rimanere calmo e di agire in modo responsabile, cercando di allontanare il ragno in modo sicuro e chiedendo aiuto se necessario. Potrebbe essere utile fare un respiro profondo, concentrarsi sulla respirazione e cercare di rilassarsi il più possibile. Inoltre, è importante che Carlo cerca di non agitarsi troppo, poiché questo potrebbe rendere più difficile per lui gestire la situazione e potrebbe anche aumentare il rischio di un morso. | 1 |
| **8**Giuseppe ha appena saputo che il suo stipendio si ridurrà il prossimo mese. *Come si sentirà Giuseppe in questa situazione?* | In questa situazione, è probabile che Giuseppe possa provare una serie di emozioni negative e stressanti. Potrebbe sentirsi deluso, frustrato, arrabbiato e preoccupato. Potrebbe anche sentirsi vulnerabile e insicuro, soprattutto se il suo stipendio è importante per il suo tenore di vita e se la riduzione potrebbe avere delle conseguenze negative sulla sua situazione finanziaria. Inoltre, potrebbe essere preoccupato per le possibili conseguenze a lungo termine della riduzione dello stipendio, come ad esempio la possibilità di dover affrontare difficoltà economiche o di dover fare sacrifici. In questa situazione, è importante che Giuseppe cerchi di elaborare le sue emozioni e di trovare il modo di affrontare la situazione in modo responsabile. Potrebbe essere utile parlare con qualcuno di cui si fida, come un amico o un familiare, o cercare il supporto di un professionista, come un consulente finanziario o un terapista. Inoltre, potrebbe essere utile fare un piano per gestire la riduzione dello stipendio e cercare di trovare soluzioni per mitigare le conseguenze negative della situazione. | 1 |
| **9**Iris ha appena saputo che ha un cancro maligno che potrebbe ucciderla in meno di un anno. *Cosa proverà Iris in questa situazione?* | In questa situazione, è probabile che Iris possa provare una serie di emozioni intense e difficili da gestire. Potrebbe sentirsi sotto shock, triste, arrabbiata, impaurita e vulnerabile. Potrebbe anche sentirsi sopraffatta dal dolore e dal senso di perdita e potrebbe avere difficoltà ad accettare la notizia. Inoltre, potrebbe essere preoccupata per il suo futuro e per come affronterà la malattia, sia fisicamente che emotivamente. In questa situazione, è importante che Iris cerchi il supporto di amici, familiari e professionisti, come un terapista o un medico, per affrontare la situazione e gestire le sue emozioni. Potrebbe essere utile parlare con qualcuno di cui si fida, come un amico o un familiare, o unirsi a un gruppo di supporto per persone che hanno affrontato o stanno affrontando una situazione simile. Inoltre, è importante che Iris si prenda cura di sé stessa e che cerchi di trovare il modo di vivere il più pienamente possibile nonostante la malattia. | 1 |
| **10**Elena ordina un panino in un ristorante. Mangia il primo boccone e guarda il resto. C’è un verme morto nel panino. *Cosa proverà Elena in questa situazione?* | In questa situazione, è probabile che Elena possa provare una serie di emozioni negative e spiacevoli. Potrebbe sentirsi disgustata, nauseata, arrabbiata e delusa. Potrebbe anche sentirsi vulnerabile e insicura, soprattutto se è particolarmente sensibile o phobic nei confronti degli insetti o se ha paura di contrarre malattie o infezioni. Inoltre, potrebbe essere preoccupata per le conseguenze dell'incidente, come ad esempio il fatto di dover cercare un altro posto dove mangiare o di dover affrontare il ristorante per chiedere un risarcimento o un rimborso. In questa situazione, è importante che Elena cerchi di rimanere calma e di agire in modo responsabile, segnalando l'incidente al personale del ristorante e chiedendo una soluzione adeguata. Potrebbe essere utile parlare con il proprietario o il manager del ristorante e fornire tutte le informazioni necessarie per risolvere la situazione in modo soddisfacente. Inoltre, potrebbe essere utile cercare il supporto di amici o familiari per gestire le emozioni negative dell'incidente. | 1 |
| **11**Paolo sente il suono che avvisa l’imminente caduta di una bomba atomica*. Cosa proverà Paolo in questa situazione?* | In questa situazione, è probabile che Paolo possa provare una serie di emozioni intense e stressanti. Potrebbe sentirsi impaurito, ansioso, panico e vulnerabile. Potrebbe anche sentirsi sopraffatto dalla paura e dall'incertezza del futuro e potrebbe avere difficoltà ad accettare la situazione. Inoltre, potrebbe essere preoccupato per le conseguenze dell'esplosione, come ad esempio il rischio di morte o di ferite gravi, o per il destino delle persone a lui care. In questa situazione, è importante che Paolo cerchi di rimanere calmo e di agire in modo responsabile, seguendo le istruzioni dell'autorità competente e cercando di mettersi al riparo nel modo più sicuro possibile. Potrebbe essere utile fare un respiro profondo, concentrarsi sulla respirazione e cercare di rilassarsi il più possibile. Inoltre, è importante che Paolo cerchi di mantenere una prospettiva realistica e di non farsi travolgere dal panico, poiché questo potrebbe rendere più difficile per lui gestire la situazione e potrebbe anche aumentare il rischio di errori o di azioni sconsiderate. | 1 |
| **12**Un uomo cammina verso Andrea e lo chiama idiota*. Come si sentirà Andrea in questa situazione?* | In questa situazione, è probabile che Andrea possa provare una serie di emozioni negative e stressanti. Potrebbe sentirsi offeso, arrabbiato, ferito e vulnerabile. Potrebbe anche sentirsi umiliato e disprezzato dalle parole dell'uomo e potrebbe avere difficoltà ad accettare questo trattamento. Inoltre, potrebbe essere preoccupato per le conseguenze dell'insulto, come ad esempio il rischio di scontri o di reazioni violente, o per il suo benessere emotivo. In questa situazione, è importante che Andrea cerchi di rimanere calmo e di agire in modo responsabile, evitando di rispondere in modo scomposto o violento e cercando di proteggere se stesso e gli altri da eventuali danni o pericoli. Potrebbe essere utile parlare con qualcuno di cui si fida, come un amico o un familiare, o cercare | 1 |
| **13**A Tania venne detto che se avesse truccato il viso ad una donna le sarebbero state pagate 20.000 lire. Ma dopo che ebbe completato il lavoro le vennero date 10.000 lire*. Come si sentirà Tania in questa situazione?* | In questa situazione, è probabile che Tania possa provare una serie di emozioni negative e frustranti. Potrebbe sentirsi delusa, arrabbiata, ingannata e vulnerabile. Potrebbe anche sentirsi umiliata e disprezzata dalla decisione della donna di non rispettare l'accordo e potrebbe avere difficoltà ad accettare questo trattamento. Inoltre, potrebbe essere preoccupata per le conseguenze della situazione, come ad esempio il rischio di perdere opportunità future o di dover affrontare difficoltà finanziarie. In questa situazione, è importante che Tania cerchi di elaborare le sue emozioni e di trovare il modo di affrontare la situazione in modo responsabile. Potrebbe essere utile parlare con qualcuno di cui si fida, come un amico o un familiare, o cercare il supporto di un professionista, come un avvocato o un consulente finanziario, se ritiene di avere diritto a un risarcimento o a un rimborso. Inoltre, potrebbe essere utile fare un piano per gestire le conseguenze della situazione e cercare di trovare soluzioni per mitigare le difficoltà finanziarie. | 1 |
| **14**Fabio lancia un libro ad un suo amico. Il suo amico non lo prende e il libro finisce in faccia ad un altro uomo.*Cosa proverà Fabio in questa situazione?* | In questa situazione, è probabile che Fabio possa provare una serie di emozioni negative e stressanti. Potrebbe sentirsi in colpa, arrabbiato, frustrato e vulnerabile. Potrebbe anche sentirsi ansioso e preoccupato per le conseguenze dell'incidente, come ad esempio il rischio di scontri o di reazioni violente, o per il suo benessere emotivo. Inoltre, potrebbe essere preoccupato per il benessere dell'altro uomo e per il modo in cui l'incidente potrebbe averlo ferito o disturbato. In questa situazione, è importante che Fabio cerchi di rimanere calmo e di agire in modo responsabile, cercando di chiarire la situazione con l'altro uomo e di scusarsi se ritiene di avere sbagliato. Potrebbe essere utile parlare con l'altro uomo e cercare di comprendere il suo punto di vista e di trovare una soluzione per risolvere l'incidente in modo soddisfacente. Inoltre, è importante che Fabio cerchi di evitare di ripetere comportamenti simili in futuro | 0 |
| **15** Roberto si sintonizza sulle radio notizie e sente che i suoi vicini hanno appena vinto 200 milioni alla lotteria. Il suo vicino sta raccontando al giornalista di tutti i viaggi che farà e di tutte le cose che comprerà.*Cosa proverà Roberto in questa situazione?* | In questa situazione, è probabile che Roberto possa provare una serie di emozioni negative e frustranti. Potrebbe sentirsi invidioso, arrabbiato, deluso e vulnerabile. Potrebbe anche sentirsi frustrato e preoccupato per il modo in cui la fortuna dei suoi vicini potrebbe influire sulla sua vita e potrebbe avere difficoltà ad accettare la situazione. Inoltre, potrebbe essere preoccupato per le conseguenze della vincita dei suoi vicini, come ad esempio il rischio di perdere opportunità o di dover affrontare difficoltà finanziarie. In questa situazione, è importante che Roberto cerchi di elaborare le sue emozioni e di trovare il modo di affrontare la situazione in modo responsabile. Potrebbe essere utile parlare con qualcuno di cui si fida, come un amico o un familiare, o cercare il supporto di un professionista, come un terapista, se ritiene di avere bisogno di aiuto per gestire le emozioni negative dell'incidente. Inoltre, è importante che Roberto cerchi di mantenere una prospettiva realistica e di non farsi travolgere dall'invidia o dalla frustrazione, poiché questo potrebbe rendere più difficile per lui gestire la situazione e potrebbe anche aumentare il rischio di errori o di azioni sconsiderate. | 1 |
| **16**Romina non vede suo marito da settimane. Poi lo vede nella strada e gli corre incontro. *Come si sentirà Romina in questa situazione?* | In questa situazione, è probabile che Romina possa provare una serie di emozioni positive e piacevoli. Potrebbe sentirsi felice, sorpresa, grata e sollevata. Potrebbe anche sentirsi emozionata e contenta di rivedere il suo marito e potrebbe avere l'impulso di corrergli incontro per abbracciarlo e dimostrargli il suo affetto. Inoltre, potrebbe essere preoccupata per il benessere del suo marito e potrebbe avere la voglia di parlare con lui per capire come sta e per scoprire cosa lo ha tenuto lontano per tanto tempo. In questa situazione, è importante che Romina cerchi di vivere le sue emozioni e di esprimerle in modo appropriato, cercando di rispettare i bisogni e le emozioni del suo marito e di comunicare in modo aperto e sincero. Potrebbe essere utile parlare con il suo marito e cercare di comprendere il suo punto di vista e di trovare | 1 |
| **17**Daniele sta camminando in una strada affollata quando inciampa e sbatte con la faccia. Tutti nella strada si girano, lo fissano e cominciano a ridere*.**Come si sentirà Daniele in questa situazione?* | In questa situazione, è probabile che Daniele si senta imbarazzato e vulnerabile, soprattutto se tutti nella strada si girano a guardarlo e a ridere. Potrebbe anche sentirsi frustrato o arrabbiato per essere stato deriso o preso in giro. Ognuno reagisce in modo diverso alle situazioni imbarazzanti, ma è comprensibile che Daniele possa sentirsi a disagio in questo momento. | 1 |
| **18**Tania sta ascoltando i numeri della lotteria. Ha già segnato cinque numeri ed è prossima a vincere 10 milioni.*Come si sentirà Tania in questa situazione?* | In questa situazione, è probabile che Tania si senta emozionata e felice. Potrebbe anche sentirsi sorpresa o incredula, soprattutto se non si aspettava di vincere così tanti soldi. È normale che le persone si sentano contente quando hanno successo o ricevono qualcosa di positivo, come vincere una somma di denaro significativa. Tania potrebbe anche sentirsi un po' nervosa o ansiosa se non è abituata a gestire grandi somme di denaro o se non sa come utilizzarle al meglio. In ogni caso, è probabile che Tania si senta felice e soddisfatta in questa situazione. | 1 |
| **19**Ogni settimana al lavoro di Giovanni sottraggono un premio a chiunque stia facendo peggio. Questa settimana è toccato a Giovanni.Come si sentirà Giovanni in questa situazione? | In questa situazione, è probabile che Giovanni si senta frustrato, deluso e forse anche arrabbiato. Potrebbe sentirsi come se fosse stato trattato ingiustamente o che il premio gli fosse stato sottratto senza una buona ragione. Potrebbe anche sentirsi demoralizzato o scoraggiato, soprattutto se questo accade spesso. Ognuno reagisce in modo diverso alle situazioni negative o sfidanti, ma è comprensibile che Giovanni possa sentirsi a disagio in questo momento. | 1 |
| **20**Katia ha ricevuto i risultati dei suoi esami: sono andati molto bene. *Come si sentirà Katia in questa situazione?* | In questa situazione, è probabile che Katia si senta felice, soddisfatta e orgogliosa di sé. Potrebbe anche sentirsi sollevata o grata per aver ottenuto buoni risultati, soprattutto se ha lavorato sodo per ottenerli. È normale che le persone si sentano contente quando hanno successo o raggiungono qualcosa di importante per loro, come ottenere buoni risultati negli esami. Katia potrebbe anche sentirsi motivata a continuare a fare bene e a superare sfide future. In ogni caso, è probabile che Katia si senta positiva e soddisfatta in questa situazione. | 1 |
| **21**Melania si trova in un vicolo. Un uomo sta andando verso di lei con un coltello*. Cosa proverà Melania in questa situazione?* | In questa situazione, è probabile che Melania si senta impaurita e in pericolo. Potrebbe anche sentirsi vulnerabile e indifesa, soprattutto se l'uomo con il coltello sta avvicinandosi e non sembra intenzionato a fermarsi. Potrebbe anche sentirsi preoccupata o ansiosa per il suo benessere e quello dei suoi cari. È normale che le persone si sentano spaventate quando sono in pericolo o sotto minaccia. In questa situazione, sarebbe comprensibile che Melania provasse una forte emozione di paura. | 1 |
| **22**Elisabetta ha da poco un nuovo fidanzato*. Come si sentirà Elisabetta in questa situazione?* | In questa situazione, è probabile che Elisabetta si senta felice e contenta di avere un nuovo fidanzato. Potrebbe anche sentirsi emozionata e ansiosa di scoprire cosa riserva il futuro per lei e il suo nuovo partner. È normale che le persone si sentano positive e ottimiste quando iniziano una nuova relazione, soprattutto se si sentono felici e appagati dalla loro nuova unione. Tuttavia, ognuno reagisce in modo diverso alle situazioni positive o emozionanti, quindi non c'è un modo univoco per descrivere come si sentirà Elisabetta in questa situazione. Potrebbe anche sentirsi un po' nervosa o preoccupata per come andrà la relazione, ma in generale è probabile che si senta felice e soddisfatta di avere un nuovo fidanzato. | 1 |
| **23**La macchina di Riccardo, che ha 20 anni, si è appena rotta lungo la strada. Mentre sta seduto lì, qualcuno passa con una nuova Jaguar. Riccardo guarda la macchina che passa.*Come si sentirà Riccardo in questa situazione?* | In questa situazione, è probabile che Riccardo si senta frustrato e scontroso. Potrebbe anche sentirsi invidioso o triste per non poter permettersi una macchina nuova e di lusso come quella che ha appena visto passare. Potrebbe anche sentirsi preoccupato o ansioso per come risolvere il problema della sua macchina rotta e per come arrivare a destinazione. Ognuno reagisce in modo diverso alle situazioni negative o sfidanti, ma è comprensibile che Riccardo possa sentirsi a disagio in questo momento. | 1 |
| **24**Giulia sta in piedi in centro alla sala nuziale per tenere un discorso. Ma mentre è in pedi non le viene niente da dire. Tutti, nella sala, la fissano e qualcuno comincia a ridere. *Come si sentirà Giulia in questa situazione*? | In questa situazione, è probabile che Giulia si senta imbarazzata e vulnerabile. Potrebbe anche sentirsi frustrata o arrabbiata per essere stata presa in giro o per non essere riuscita a tenere il discorso come aveva previsto. Potrebbe anche sentirsi nervosa o ansiosa per il giudizio degli altri. Ognuno reagisce in modo diverso alle situazioni imbarazzanti, ma è comprensibile che Giulia possa sentirsi a disagio in questo momento. | 0 |
| **25**Elisa vorrebbe un coniglietto per il suo compleanno, ma le è stato detto dai suoi genitori che le sarà donata una bicicletta, che però lei non vuole. Ma, quando scende per aprire il suo regalo, scopre che dentro c’è un coniglietto.*Come si sentirà Elisa in questa situazione?* | In questa situazione, è probabile che Elisa si senta felice e contenta di aver ricevuto il coniglietto che desiderava. Potrebbe anche sentirsi sorpresa e grata per il gesto dei suoi genitori. È normale che le persone si sentano contente quando otteniamo qualcosa di importante o di significativo per noi, come un regalo che desideriamo da tempo. Elisa potrebbe anche sentirsi un po' in colpa o imbarazzata per non aver voluto la bicicletta che le era stata offerta inizialmente. In ogni caso, è probabile che Elisa si senta felice e soddisfatta in questa situazione. | 1 |
| **26**Silvia si sveglia e vede che c’è un ragno velenoso nel suo letto. *Cosa proverà Silvia in questa situazione?* | In questa situazione, è probabile che Silvia si senta spaventata e in pericolo. Potrebbe anche sentirsi vulnerabile e indifesa, soprattutto se il ragno velenoso si sta avvicinando o sembra pronto a morderla. Potrebbe anche sentirsi preoccupata o ansiosa per il suo benessere e quello dei suoi cari. È normale che le persone si sentano spaventate quando sono in pericolo o sotto minaccia. In questa situazione, sarebbe comprensibile che Silvia provasse una forte emozione di paura. | 1 |
| **27** Giacomo si è appena preparato una scodella di corn flakes e latte. Ne mangia una cucchiaiata e si rende conto che il latte è andato a male. Cosa proverà Giacomo in questa situazione? | In questa situazione, è probabile che Giacomo si senta disgustato e nauseato dal gusto e dall'odore del latte andato a male. Potrebbe anche sentirsi deluso o frustrato per essersi preparato una colazione che non può mangiare. Potrebbe anche sentirsi preoccupato o ansioso per il suo benessere, soprattutto se ha mangiato già una cucchiaiata di latte andato a male. È normale che le persone si sentano disgustate o nauseate da cibi o bevande che hanno un cattivo odore o un sapore sgradevole. In questa situazione, sarebbe comprensibile che Giacomo provasse una forte emozione di disgusto. | 1 |
| **28**Sonia sta andando in bicicletta lungo la strada quando perde il controllo e finisce addosso ad un lampione. Alcune persone dall’altra parte della strada si fermano e la fissano; qualcuno la deride. Come si sentirà Sonia in questa situazione? | In questa situazione, è probabile che Sonia si senta imbarazzata e vulnerabile. Potrebbe anche sentirsi frustrata o arrabbiata per essere stata derisa o presa in giro da altre persone. Potrebbe anche sentirsi dolorante o ferita se è caduta dalla bicicletta e si è fatta male. Ognuno reagisce in modo diverso alle situazioni imbarazzanti o sfidanti, ma è comprensibile che Sonia possa sentirsi a disagio in questo momento. | 1 |
| **29**Un uomo cammina verso Massimo e minaccia di dargli un pugno. *Come si sentirà Massimo in questa situazione?* | In questa situazione, è probabile che Massimo si senta spaventato e in pericolo. Potrebbe anche sentirsi vulnerabile e indifeso, soprattutto se l'uomo che lo minaccia è più grande o più forte di lui. Potrebbe anche sentirsi preoccupato o ansioso per il suo benessere e per quello delle persone intorno a lui. È normale che le persone si sentano spaventate quando sono in pericolo o sotto minaccia. In questa situazione, sarebbe comprensibile che Massimo provasse una forte emozione di paura. | 1 |
| **30**William è appena andato al primo appuntamento con una ragazza di cui è interessato da quattro anni. Alla fine dell’appuntamento si baciano.*Come si sentirà William in questa situazione?* | In questa situazione, è probabile che William si senta felice e soddisfatto. Potrebbe anche sentirsi emozionato e contento di aver finalmente ottenuto ciò che desiderava. Potrebbe anche sentirsi romantico e coinvolto emotivamente nella relazione. È normale che le persone si sentano felici e contente quando otteniamo qualcosa di importante o di significativo per noi, come un appuntamento o un bacio con qualcuno di cui siamo interessati. In questa situazione, sarebbe comprensibile che William provasse una forte emozione di gioia e soddisfazione. | 1 |
| **31**Margherita ha appena scoperto che sua madre è morta. *Come si sentirà Margherita in questa situazione?* | In questa situazione, è probabile che Margherita si senta devastata e molto triste. Potrebbe anche sentirsi confusa, sconvolta, sopraffatta dal dolore e dallo shock. La morte di una madre può essere un evento molto traumatico e difficile da affrontare, soprattutto se la persona era particolarmente legata alla madre. Margherita potrebbe anche sentirsi sola e vulnerabile senza la sua madre nella sua vita. In questa situazione, sarebbe comprensibile che Margherita provasse una forte emozione di dolore e tristezza. | 1 |
| **32**A Giovanna è stata appena data una promozione. *Come si sentirà Giovanna in questa situazione?* | Giovanna potrebbe sentirsi molto soddisfatta e felice di aver ricevuto una promozione. Potrebbe anche sentirsi sorpresa o orgogliosa di sé stessa per aver ottenuto questo riconoscimento del suo lavoro e impegno. Potrebbe anche sentirsi un po' nervosa o preoccupata riguardo alle responsabilità aggiuntive o alle aspettative che potrebbero essere associate alla sua nuova posizione. Tuttavia, in generale, è probabile che Giovanna si senta molto soddisfatta e gratificata di aver ricevuto una promozione. | 1 |
| **33**Sara ha parcheggiato all’angolo della strada quando una donna giunge e colpisce la sua auto con un martello, danneggiando parecchio il cofano.*Come si sentirà Sara in questa situazione?* | Sara potrebbe sentirsi molto arrabbiata, frustrata e sconvolta in questa situazione. Potrebbe anche sentirsi sorpresa e scioccata dal fatto che qualcuno abbia danneggiato intenzionalmente la sua auto in questo modo. Potrebbe sentirsi anche preoccupata per il costo della riparazione dell'auto e per il fatto di dover fare i conti con l'assicurazione e le eventuali conseguenze legali. In generale, è probabile che Sara si senta molto infastidita e disturbata da questo incidente. | 1 |
| **34**Rebecca sta tirando fuori dal frigo il pranzo della domenica quando si accorge che stanno strisciando dei vermi.*Come si sentirà Rebecca in questa situazione?* | Rebecca potrebbe sentirsi molto disgustata e nauseata in questa situazione. Potrebbe anche sentirsi arrabbiata o infastidita dal fatto che il cibo che aveva pensato di mangiare sia stato contaminato da vermi. Potrebbe sentirsi anche preoccupata per la sua salute e per il fatto di dover trovare un altro pasto al posto del pranzo che aveva programmato di mangiare. In generale, è probabile che Rebecca si senta molto spiacevole e sconvolta da questa situazione. | 1 |
| **35**Arturo sta parlando con un uomo che non conosce molto bene. L’uomo sta mangiando un panino. Mentre stanno parlando l’uomo sputa un pezzo di cibo nella giacca di Arturo. Arturo afferra l’uomo e gli strappa a pezzi la maglietta.*Come si sentirà Arturo in questa situazione?* | In questa situazione, è probabile che Arturo si senta molto arrabbiato e infastidito. Potrebbe sentirsi anche offeso e disgustato dal comportamento maleducato dell'uomo e dal fatto che abbia sputato il cibo sulla sua giacca. Potrebbe anche sentirsi imbarazzato o imbarazzato per il suo comportamento impulsivo di afferrare e strappare la maglietta dell'uomo. In generale, è probabile che Arturo si senta molto frustrato e arrabbiato in questa situazione. | 1 |
| **36**A Nadia venne detto che il suo prossimo lavoro sarebbe stato a Roma, la sua città preferita. Invece sarà a Padova, città che non le piace*.**Come si sentirà Nadia in questa situazione?* | Nadia potrebbe sentirsi molto delusa e frustrata in questa situazione. Potrebbe anche sentirsi arrabbiata o infastidita dal fatto che le sia stata data una notizia incorretta o che i piani siano cambiati senza il suo consenso. Potrebbe sentirsi anche preoccupata per il fatto di dover trasferirsi in una città che non le piace e per le possibili difficoltà che potrebbero insorgere a causa di questo cambiamento. In generale, è probabile che Nadia si senta molto insoddisfatta e scoraggiata da questa situazione. | 1 |
| **37**Giampaolo sta campeggiando nella foresta in America. Un orso strappa una parte della sua tenda e viene verso di lui ringhiando.*Come si sentirà Giampaolo in questa situazione?* | Giampaolo potrebbe sentirsi molto spaventato e preoccupato in questa situazione. Potrebbe sentirsi anche vulnerabile e indifeso di fronte all'orso che si avvicina ringhiando. Potrebbe anche sentirsi confuso o disorientato su cosa fare o come proteggersi. In generale, è probabile che Giampaolo si senta molto spaventato e preoccupato per la sua sicurezza in questa situazione. | 1 |
| **38**Ad Andrea, disoccupato, è stato appena offerto un nuovo lavoro. *Come si sentirà Andrea in questa situazione?* | Andrea potrebbe sentirsi molto felice e soddisfatto di aver ricevuto un'offerta di lavoro. Potrebbe anche sentirsi sollevato e grato per aver trovato un'opportunità di lavoro in un momento in cui era disoccupato. Potrebbe sentirsi anche un po' nervoso o preoccupato riguardo alle responsabilità e alle aspettative del nuovo lavoro. Tuttavia, in generale, è probabile che Andrea si senta molto contento e gratificato di aver ricevuto questa offerta di lavoro. | 1 |
| **39**Simone si accorge che l’uomo accanto a lui possiede l’orologio d’oro che lui vuole comprarsi da tre anni. Simone sa che non potrà permettersi quel orologio per un altro po’ di anni.*Come si sentirà Simone in questa situazione? Come si sentirà Sonia in questa situazione?* | Simone potrebbe sentirsi molto invidioso e frustrato in questa situazione. Potrebbe sentirsi anche deluso e scoraggiato dal fatto di non poter permettersi l'orologio che desidera tanto. Potrebbe sentirsi anche preoccupato per il fatto di dover aspettare ancora per poterselo permettere. Sonia, d'altra parte, potrebbe non sentirsi affatto coinvolta o interessata all'orologio in questione e quindi potrebbe non avere alcuna emozione in particolare in questa situazione. Oppure, se Sonia condivide l'interesse di Simone per l'orologio, potrebbe sentirsi delusa e frustrata insieme a lui per il fatto che non possano permettersi di acquistarlo al momento. | 1 |
| **40**L’amica di Sonia le disse che se avesse badato lei ai suoi figli lunedì lei avrebbe badato i figli di Sonia giovedì. Sonia il lunedì badò i figli dell’amica, ma il giovedì l’amica le disse che non poteva badare i figli di Sonia perché aveva un altro appuntamento. *Come si sarà sentita Sonia in questa situazione?* | Sonia potrebbe sentirsi molto delusa e arrabbiata in questa situazione. Potrebbe sentirsi anche tradita e ferita dal fatto che l'amica non abbia mantenuto la parola data. Potrebbe sentirsi anche preoccupata per il fatto di dover trovare un'altra soluzione per badare ai suoi figli il giovedì. In generale, è probabile che Sonia si senta molto frustrata e arrabbiata per questa mancanza di rispetto dell'amica verso l'accordo che avevano fatto. | 1 |
| **41**Mario è stato chiamato dal suo capo. Questo gli dice: ” Sei licenziato.” *Come si sentirà Mario in questa situazione?* | Mario potrebbe sentirsi molto scioccato, sorpreso e deluso in questa situazione. Potrebbe anche sentirsi arrabbiato o frustrato dal fatto di essere stato licenziato senza preavviso o spiegazioni. Potrebbe sentirsi anche preoccupato per il suo futuro lavorativo e per come sosterrà se stesso e la sua famiglia senza il lavoro. In generale, è probabile che Mario si senta molto sconcertato e infelice per aver ricevuto questa notizia. | 1 |
| **42**La moglie di Daniele ha appena partorito il loro terzo figlio. *Come si sentirà la moglie di Daniele in questa situazione*? | La moglie di Daniele potrebbe sentirsi molto stanca e dolorante dopo il parto. Potrebbe anche sentirsi emozionata e grata per aver dato alla luce un altro figlio sano. Potrebbe anche sentirsi preoccupata per il fatto di dover gestire tre figli e per come il nuovo arrivato influirà sulla routine familiare. Tuttavia, in generale, è probabile che la moglie di Daniele si senta molto felice e soddisfatta per aver dato alla luce il suo terzo figlio. | 0 |
| **43**Fulvio si trova in banca nel mezzo di una rapina. Uno dei rapinatori gli punta la pistola in faccia. *Come si sentirà Fulvio in questa situazione?* | Fulvio potrebbe sentirsi molto spaventato e terrorizzato in questa situazione. Potrebbe anche sentirsi vulnerabile e indifeso di fronte al rapinatore che gli punta la pistola in faccia. Potrebbe anche sentirsi confuso o disorientato su cosa fare o come proteggersi. In generale, è probabile che Fulvio si senta molto spaventato e preoccupato per la sua sicurezza in questa situazione. | 1 |
| **44**Giusy corre dentro al cinema e non vede la porta di vetro. Ci corre contro. Tutti nella fila si girano e la fissano e qualcuno la deride.*Come si sentirà Giusy in questa situazione?* | Giusy potrebbe sentirsi molto imbarazzata e mortificata in questa situazione. Potrebbe anche sentirsi sorpresa e sconvolta dal fatto di non aver visto la porta di vetro e di essersi schiantata contro di essa. Potrebbe sentirsi anche arrabbiata o frustrata per il fatto di essere stata derisa da qualcuno. In generale, è probabile che Giusy si senta molto infelice e a disagio per questo incidente imbarazzante. | 1 |
| **45**Katia ha appena scoperto che le sono stati rubati 10 milioni e non può fare niente per averli indietro. *Come si sentirà Katia in questa situazione?* | Katia potrebbe sentirsi molto arrabbiata, frustrata e impotente in questa situazione. Potrebbe anche sentirsi vulnerabile e indifesa di fronte al fatto di aver subito un furto così ingente. Potrebbe sentirsi anche preoccupata per il fatto di dover fare i conti con questa perdita finanziaria e per come questo potrebbe influire sulla sua vita. In generale, è probabile che Katia si senta molto infastidita e sconvolta da questa situazione. | 1 |
| **46**Mauro si è svegliato e sente un ladro che si muove in casa. *Come si sentirà Mauro in questa situazione*? | Mauro potrebbe sentirsi molto spaventato e preoccupato in questa situazione. Potrebbe anche sentirsi vulnerabile e indifeso di fronte alla presenza di un ladro in casa sua. Potrebbe sentirsi anche confuso o disorientato su cosa fare o come proteggersi. In generale, è probabile che Mauro si senta molto spaventato e preoccupato per la sua sicurezza in questa situazione. | 1 |
| **47**Edy si trova in un bar quando scivola in una macchia di grasso e cade. Tutti nel bar lo fissano. *Come si sentirà Edy in questa situazione?* | Edy potrebbe sentirsi molto imbarazzato e mortificato in questa situazione. Potrebbe anche sentirsi sorpreso e sconvolto dal fatto di aver perso l'equilibrio e di essere caduto. Potrebbe sentirsi anche arrabbiato o frustrato per il fatto di essere stato oggetto di tanto interesse o derisione da parte degli altri presenti nel bar. In generale, è probabile che Edy si senta molto infelice e a disagio per questo incidente imbarazzante. | 1 |
| **48**Enrico sta camminando lungo una via del paese. Purtroppo non vede un ciottolo. La bici sbanda e lui cade nell’erba. Una macchina si ferma e tutti lo vedono cadere. *Cosa proverà Enrico in questa situazione?* | Enrico potrebbe sentirsi molto imbarazzato e mortificato in questa situazione. Potrebbe anche sentirsi sorpreso e sconvolto dal fatto di aver perso l'equilibrio e di essere caduto. Potrebbe sentirsi anche arrabbiato o frustrato per il fatto di essere stato oggetto di tanto interesse o derisione da parte degli altri presenti sulla strada. In generale, è probabile che Enrico si senta molto infelice e a disagio per questo incidente imbarazzante. | 1 |
| **49**Ogni volta che Nicoletta tenta di lavorare al computer non ne trova uno libero al lavoro. *Cosa proverà Nicoletta in questa situazione?* | Nicoletta potrebbe sentirsi molto frustrata e infastidita in questa situazione. Potrebbe anche sentirsi arrabbiata o delusa dal fatto di non riuscire a trovare un computer libero al lavoro. Potrebbe sentirsi anche preoccupata per il fatto di non essere in grado di completare i suoi compiti o di incontrare le scadenze a causa della mancanza di accesso a un computer. In generale, è probabile che Nicoletta si senta molto infastidita e sconcertata da questa situazione. | 1 |
| **50**Alessandro sta guardando dalla ringhiera di un ponte. Improvvisamente comincia a scivolare e finisce dritto dentro al fiumiciattolo. Mentre si arrampica fuori dall’acqua si accorge che c’è un pullman pieno di turisti che lo stanno fissando.*Come si sentirà Alessandro in questa situazione?* | Alessandro potrebbe sentirsi molto imbarazzato e mortificato in questa situazione. Potrebbe anche sentirsi sorpreso e sconvolto dal fatto di essere scivolato dalla ringhiera del ponte e di essere caduto in acqua. Potrebbe sentirsi anche arrabbiato o frustrato per il fatto di essere stato oggetto di tanto interesse o derisione da parte dei turisti presenti nel pullman. In generale, è probabile che Alessandro si senta molto infelice e a disagio per questo incidente imbarazzante. | 1 |
| **51**Gianni viene portato in un safari. L’auto si è rotta e adesso un grande rinoceronte lo sta attaccando. *Come si sentirà Gianni in questa situazione?* | Gianni potrebbe sentirsi molto spaventato e terrorizzato in questa situazione. Potrebbe anche sentirsi vulnerabile e indifeso di fronte al rinoceronte che lo sta attaccando. Potrebbe sentirsi anche confuso o disorientato su cosa fare o come proteggersi. In generale, è probabile che Gianni si senta molto spaventato e preoccupato per la sua sicurezza in questa situazione. | 1 |
| **52**Emilia sta camminando mano nella mano con il suo amante lungo la strada. Suo marito, improvvisamente, compare dietro l’angolo e li sorprende insieme.*Cosa proverà Emilia in questa situazione?* | Emilia potrebbe sentirsi molto imbarazzata, mortificata e preoccupata in questa situazione. Potrebbe anche sentirsi sorpresa e sconvolta dal fatto di essere stata sorpresa dal marito mentre stava camminando mano nella mano con il suo amante. Potrebbe sentirsi anche arrabbiata o frustrata per il fatto di essere stata scoperta e di dover affrontare le conseguenze delle sue azioni. In generale, è probabile che Emilia si senta molto infelice e a disagio per questa situazione imbarazzante. | 1 |
| **53**Simone scopre un ladro mentre ruba il suo portafoglio dalla borsa. *Come si sentirà Simone in questa situazione?* | Simone potrebbe sentirsi molto arrabbiato, frustrato e impotente in questa situazione. Potrebbe anche sentirsi vulnerabile e indifeso di fronte al ladro che sta rubando il suo portafoglio. Potrebbe sentirsi anche preoccupato per il fatto di dover fare i conti con questa perdita e per come questo potrebbe influire sulla sua vita. In generale, è probabile che Simone si senta molto infastidito e sconcertato da questa situazione. | 1 |
| **54**Filippo si era iscritto ad una gara di pesca e voleva vincere. Così comprò un grosso pesce dal pescivendolo. Purtroppo uno dei giudici era il proprietario del negozio. Quest’uomo racconta a tutti cosa ha fatto Filippo.*Cosa proverà Filippo in questa situazione?* | Filippo potrebbe sentirsi molto imbarazzato, mortificato e deluso in questa situazione. Potrebbe anche sentirsi sorpreso e sconvolto dal fatto di essere stato scoperto dal giudice che era anche il proprietario del negozio da cui aveva acquistato il pesce. Potrebbe sentirsi anche arrabbiato o frustrato per il fatto di essere stato sorpreso e di dover affrontare le conseguenze delle sue azioni. In generale, è probabile che Filippo si senta molto infelice e a disagio per questa situazione imbarazzante. | 1 |
| **55**Il capo di Priscilla le ha detto di fare il suo lavoro in un certo modo. Poi, alla fine della giornata, dopo che lei ha lavorato per ore, egli cambia idea vuole qualcosa di differente. *Come si sentirà Priscilla in questa situazione?* | Priscilla potrebbe sentirsi molto frustrata e infastidita in questa situazione. Potrebbe anche sentirsi arrabbiata o delusa dal fatto che il suo capo le abbia detto di fare il lavoro in un certo modo, solo per cambiare poi idea alla fine della giornata. Potrebbe sentirsi anche preoccupata per il fatto di dover rifare il lavoro o di dover gestire le conseguenze del cambiamento di idea del capo. In generale, è probabile che Priscilla si senta molto infastidita e sconcertata da questa situazione. | 1 |
| **56**Paolo ha fatto un contratto con un uomo. Se spedisce all’uomo la sua collezione di dischi, l’uomo gli dà un’auto. Paolo spedisce all’uomo la sua collezione di dischi. L’uomo però non gli dà l’auto come stabilito.*Come si sentirà Paolo in questa situazione?* | In questa situazione, è probabile che Paolo si senta deluso, frustrato o arrabbiato per il fatto che l'uomo non abbia rispettato il contratto e non gli abbia dato l'auto come promesso. Potrebbe sentirsi ingannato o truffato dall'uomo e potrebbe essere tentato di fare qualcosa per risolvere la situazione. Tuttavia, è importante considerare che ogni persona reagisce in modo diverso alle circostanze e alle aspettative non soddisfatte, quindi è possibile che Paolo abbia reagito in modo diverso da come ci si aspetterebbe. | 1 |
| **57**A Matteo venne detto che avrebbe avuto il suo gioco preferito per Natale. Invece gli venne regalata un’enciclopedia.*Come si sarà sentito Matteo in questa situazione?* | È difficile dire esattamente come si sarebbe sentito Matteo in questa situazione senza avere ulteriori informazioni su di lui e sulla sua personalità. Tuttavia, è probabile che Matteo si sarebbe sentito deluso o frustrato dal fatto di non aver ricevuto il suo gioco preferito come regalo di Natale. Potrebbe anche essere sorpreso o sorpreso dal ricevere invece un'enciclopedia come regalo. In ogni caso, è importante considerare che ogni persona reagisce in modo diverso alle circostanze e alle aspettative non soddisfatte, quindi è possibile che Matteo abbia reagito in modo diverso da come ci si aspetterebbe. | 1 |
| **58**I genitori di Anna si aspettano da lei tutti voti ottimi nei suoi esami. Lei apre la lettera dei risultati davanti a loro. Tutti vedono che ha preso appena sufficiente in ogni esame. *Come si sentirà Anna in questa situazione?* | In questa situazione, è probabile che Anna si senta delusa, frustrata o nervosa per il fatto di aver ottenuto solo voti sufficienti nei suoi esami, soprattutto se i suoi genitori si aspettavano voti ottimi da lei. Potrebbe sentirsi sotto pressione o in colpa per non aver soddisfatto le aspettative dei suoi genitori e potrebbe essere preoccupata per come reagiranno i suoi genitori ai suoi risultati. Tuttavia, è importante considerare che ogni persona reagisce in modo diverso alle circostanze e alle aspettative non soddisfatte, quindi è possibile che Anna abbia reagito in modo diverso da come ci si aspetterebbe. | 0 |

**Punteggio Tristezza** (item 1,4,6,8,9,19,31,36,41,57)**: 9/10**

**Punteggio Paura** (item 2,7,11,21,26,29,37,43,46,51)**: 10/10**

**Punteggio Imbarazzo** (item 3,14,17,24,28,44,47,48,50,52,54,58)**: 8/12**

**Punteggio Felicità** (item 5,16,18,20,22,25,30,32,38,42)**: 9/10**

**Punteggio Disgusto** (item 10,27,34)**: 3/3**

**Punteggio Rabbia** (item 12,13,33,35,40,45,49,53,55,56)**: 10/10**

**Punteggio Invidia** (item 15,23,39)**: 3/3**

**Test delle Situazioni Sociali^[[1]](#footnote-1)^**

- **Prompt - Item 1a:**Carlo era un impiegato che lavorava in un ufficio della sua città. A mezzogiorno egli pranzava in un piccolo parco. Spesso egli rompeva parte di un panino in piccoli pezzi, spargendoli nel terreno per i piccioni.

  A - Comportamento normale in quella situazione
  B - Comportamento un po’ strano in quella situazione
  C - Comportamento abbastanza strano in quella situazione
  D - Comportamento estremamente strano in quella situazione

  **Risposta ChatGPT:**
  Per la prima situazione, sceglierei la risposta B - Comportamento un po' strano in quella situazione. Rompere il panino in piccoli pezzi e spargendoli per il terreno per nutrire i piccioni potrebbe essere considerato un comportamento un po' strano, soprattutto se questa è un'abitudine che Carlo ha durante ogni pausa pranzo. Non è necessariamente un comportamento inappropriato o negativo, ma potrebbe essere considerato un po' insolito.
- **Prompt - Item 1b:**
  Un giorno, mentre si sedeva nella panchina, un passeggino venne parcheggiato proprio lì davanti. Carlo notò che vicino una giovane donna stava dondolando un bambino un po’ più grande. Il bambino nel passeggino cominciò a piangere ma la mamma non lo sentiva. Carlo ha imparato che quando il suo piccolo nipote grida, talvolta significa che il suo pannolino si è aperto. Piuttosto che disturbare la madre nel parco, Carlo velocemente
  controllò i vestitini del bambino per vedere se poteva sentire il pannolino
  aperto.A - Comportamento normale in quella situazione
  B - Comportamento un po’ strano in quella situazione
  C - Comportamento abbastanza strano in quella situazione
  D - Comportamento estremamente strano in quella situazione

  **Risposta ChatGPT:**
  Sceglierei la risposta A - Comportamento normale in quella situazione per la seconda situazione descritta. Carlo si è dimostrato attento e premuroso nei confronti del bambino, cercando di aiutare la madre senza disturbare troppo. Offrire aiuto in questo modo può essere considerato un comportamento normale e gentile in questa situazione.
- **Prompt - Item 2a:**Emilia si svegliò in ritardo il giorno del suo viaggio in aereo. C’era appena il
  tempo per vestirsi e andare all’aeroporto, così saltò la sua colazione. A - Comportamento normale in quella situazione
  B - Comportamento un po’ strano in quella situazione
  C - Comportamento abbastanza strano in quella situazione
  D - Comportamento estremamente strano in quella situazione

  **Risposta ChatGPT:**
  Sceglierei la risposta A - Comportamento normale in quella situazione. In questa situazione, saltare la colazione per il viaggio in aereo potrebbe essere considerato un comportamento normale, poiché Emilia ha bisogno di fare in fretta per arrivare all'aeroporto in tempo. Saltare la colazione in momenti di fretta o quando si è in ritardo non è un comportamento insolito o strano.
- **Prompt - Item 2b:**A mezzogiorno la hostess venne con il pranzo, ma Emilia era così affamata
  che una porzione non l’aveva soddisfatta. Guardò una ragazzina attraverso il
  corridoio che giocherellava con il suo cibo, lamentandosi: “Non riesco a mangiarlo”. Emilia si sporse dal corridoio e disse: “Se la sua piccola bambina non vuole il suo vassoio, lo può passaredi qua per me?
  A - Comportamento normale in quella situazione
  B - Comportamento un po’ strano in quella situazione
  C - Comportamento abbastanza strano in quella situazione
  D - Comportamento estremamente strano in quella situazione

**Risposta ChatGPT:**Sceglierei la risposta B - Comportamento un po' strano in quella situazione per la descrizione fornita. Anche se Emilia potrebbe essere affamata e desiderosa di mangiare di più, chiedere il vassoio della piccola bambina potrebbe essere considerato un po' strano o inopportuno. Potrebbe essere più normale chiedere al personale di bordo se è possibile avere un'altra porzione o se ci sono altre opzioni di cibo disponibili.

- **Prompt - Item 3a:**Ruggero viveva da solo e un giorno venne invitato a cena a casa di un’amica della madre. In genere egli era solito avvertire la cameriera che non mangiava carne e che avrebbe desiderato che la sua verdura gli venisse servita scondita. Quando Ruggero arrivò all’appuntamento si ricordò che non aveva toccato cibo da due ore*.* Senza perdere alcun tempo, ancor prima di essere presentato, egli chiese alla cameriera quando la cena sarebbe stata servita.

  A - Comportamento normale in quella situazione
  B - Comportamento un po’ strano in quella situazione
  C - Comportamento abbastanza strano in quella situazione
  D - Comportamento estremamente strano in quella situazione

**Risposta ChatGPT:**
Sceglierei la risposta C - Comportamento abbastanza strano in quella situazione. Anche se Ruggero era solito avvertire la cameriera della sua preferenza alimentare, chiedere quando la cena sarebbe stata servita appena arrivato all'appuntamento potrebbe essere considerato un po' strano. Non è del tutto improprio, ma potrebbe essere considerato un po' inopportuno o impaziente.

- **Prompt - Item 3b:**
  Lei rispose che ci sarebbe voluta ancora un’ora prima che il cibo fosse pronto*.*Sentendo ciò, Ruggero aprì la sua valigetta, prese una mela e alcune noci e prontamente le mangiò. A - Comportamento normale in quella situazione
  B - Comportamento un po’ strano in quella situazione
  C - Comportamento abbastanza strano in quella situazione
  D - Comportamento estremamente strano in quella situazione

**Risposta ChatGPT:**
Sceglierei la risposta A - Comportamento normale in quella situazione per la descrizione fornita. Ruggero ha detto di non avere toccato cibo da due ore e ha appena appreso che ci sarebbe voluta ancora un'ora prima che il cibo fosse pronto. In questa situazione, mangiare una mela e delle noci per placare la fame potrebbe essere considerato un comportamento normale e comprensibile. Non c'è nulla di strano o inappropriato in questo comportamento.

- **Prompt - Item 3c:**Proprio prima di cena, la cameriera gli fece vedere un attraente piatto di frutti e
  verdure, chiedendo se gli sembrava abbastanza. “Va bene, grazie” – disse Ruggero - “ma se non le dispiace io aspetterei un’altra ora per mangiare. Ho già preso qualcosa un’ora fa”A - Comportamento normale in quella situazione
  B - Comportamento un po’ strano in quella situazione
  C - Comportamento abbastanza strano in quella situazione
  D - Comportamento estremamente strano in quella situazione

**Risposta ChatGPT:**
Sceglierei la risposta B - Comportamento un po' strano in quella situazione per la descrizione fornita. Anche se Ruggero può avere già preso qualcosa da mangiare un'ora prima, rifiutare il cibo offerto dalla cameriera prima di cena potrebbe essere considerato un po' strano o inopportuno. Potrebbe essere più normale ringraziare per il cibo offerto e chiedere se è possibile mangiarlo in un secondo momento o in un altro momento della serata.

- **Prompt - Item 4:**Elisabetta era diabetica da tutta la vita. I dottori le avevano detto di stare molto
  attenta alla sua dieta per evitare serie complicazioni. Quando veniva invitata
  da qualcuno per un pranzo, lei spiegava il problema in anticipo*.* Ma durante
  gli incontri importanti godeva delle conversazioni e della compagnia che
  trovava, aspettando di tornare a casa per mangiare.A - Comportamento normale in quella situazione
  B - Comportamento un po’ strano in quella situazione
  C - Comportamento abbastanza strano in quella situazione
  D - Comportamento estremamente strano in quella situazione

  **Risposta ChatGPT:**
  Sceglierei la risposta A - Comportamento normale in quella situazione per la descrizione fornita. Elisabetta ha una condizione diabetica e deve seguire una dieta attentamente pianificata per evitare complicazioni. In questa situazione, spiegare il problema in anticipo quando viene invitata a pranzo e aspettare di tornare a casa per mangiare durante gli incontri importanti potrebbe essere considerato un comportamento normale e responsabile. Non c'è nulla di strano o inappropriato in questo comportamento.
- **Prompt - Item 5a:**Franco faceva il giardiniere. Di solito si portava il pranzo al sacco*.* A mezzogiorno, Franco si lavava sempre le mani sotto un rubinetto e sedeva in una parte ombreggiata del giardino per mangiare.

  A - Comportamento normale in quella situazione
  B - Comportamento un po’ strano in quella situazione
  C - Comportamento abbastanza strano in quella situazione
  D - Comportamento estremamente strano in quella situazione

  **Risposta ChatGPT:**Sceglierei la risposta A - Comportamento normale in quella situazione per la descrizione fornita. Franco è un giardiniere e di solito si porta il pranzo al sacco. Lavarsi le mani sotto un rubinetto e sedersi in una parte ombreggiata del giardino per mangiare durante la pausa pranzo potrebbe essere considerato un comportamento normale e appropriato per questa situazione. Non c'è nulla di strano o inappropriato in questo comportamento.
- **Prompt - Item 5b:**
  Un giorno cominciò a piovere a mezzogiorno. Franco bussò alla porta della
  casa dove lavorava e chiese il permesso di mangiare dentro. La signora gli
  disse di entrare e lui individuò da solo la sala da pranzo. Tolse le briciole dal
  tavolo e guardò intorno alla casa in cerca di un posto dove riposarsi.

  A - Comportamento normale in quella situazione
  B - Comportamento un po’ strano in quella situazione
  C - Comportamento abbastanza strano in quella situazione
  D - Comportamento estremamente strano in quella situazione

  **Risposta ChatGPT:**Sceglierei la risposta A - Comportamento normale in quella situazione per la descrizione fornita. Franco è stato invitato dalla signora a entrare in casa per mangiare durante la pausa pranzo a causa della pioggia. Cercare un posto dove riposarsi e togliere le briciole dal tavolo prima di sedersi a mangiare potrebbe essere considerato un comportamento normale e rispettoso in questa situazione. Non c'è nulla di strano o inappropriato in questo comportamento.
- **Prompt - Item 5c:**Il tappeto del salotto era sottile, perciò Franco decise di rannicchiarsi per il suo pisolino sopra una larga poltrona.

  A - Comportamento normale in quella situazione
  B - Comportamento un po’ strano in quella situazione
  C - Comportamento abbastanza strano in quella situazione
  D - Comportamento estremamente strano in quella situazione

  **Risposta ChatGPT:**Sceglierei la risposta B - Comportamento un po' strano in quella situazione per la descrizione fornita. Anche se Franco potrebbe essere stanco e desiderare fare un pisolino durante la pausa pranzo, scegliere di rannicchiarsi sopra una larga poltrona nel salotto di qualcuno potrebbe essere considerato un po' strano o inopportuno. Potrebbe essere più normale cercare un posto più discreto o appropriato per fare un pisolino, come una stanza vuota o una sedia comoda.
- **Prompt - Item 6:**
  Il supermercato dove Roberto era solito fare la spesa aveva una piccola scritta nella porta che diceva: “E’ proibito entrare scalzi in questo magazzino”. Un giorno d’estate Roberto vide una bella ragazza entrare nel magazzino a piedi scalzi. Roberto decise di far in modo che i piedi di lei non venissero visti dal direttore. Spinse il suo carrello vicino e davanti a lei, corridoio dopo corridoio.

  A - Comportamento normale in quella situazione
  B - Comportamento un po’ strano in quella situazione
  C - Comportamento abbastanza strano in quella situazione
  D - Comportamento estremamente strano in quella situazione

  **Risposta ChatGPT:**Sceglierei la risposta D - Comportamento estremamente strano in quella situazione per la descrizione fornita. Roberto ha deciso di seguire una sconosciuta per il supermercato e di bloccare la sua vista con il carrello per evitare che il direttore la veda a piedi scalzi, nonostante il divieto di entrare scalzi nel magazzino. Questo comportamento potrebbe essere considerato estremamente strano e inappropriato. Non c'è alcun motivo per cui Roberto dovrebbe interferire in questo modo con gli altri clienti del supermercato.
- **Prompt - Item 7a:**Matteo è stato appena nominato in un importante nuovo lavoro. Alle 6 in punto del giorno in cui pensava di cominciare il nuovo lavoro, Matteo ricevette una telefonata da sua madre che stava molto male. Matteo era molto preoccupato e così infilò un maglione sopra il suo pigiama e guidò verso la casa di sua madre per curarla. A - Comportamento normale in quella situazione
  B - Comportamento un po’ strano in quella situazione
  C - Comportamento abbastanza strano in quella situazione
  D - Comportamento estremamente strano in quella situazione **Risposta ChatGPT:**Sceglierei la risposta A - Comportamento normale in quella situazione per la descrizione fornita. Matteo è stato appena nominato in un importante nuovo lavoro, ma sua madre è molto malata e ha bisogno del suo aiuto. In questa situazione, indossare un maglione sopra il pigiama e guidare verso la casa di sua madre per curarla potrebbe essere considerato un comportamento normale e responsabile. Non c'è nulla di strano o inappropriato in questo comportamento.
- **Prompt - Item 7b:** Dopo che il dottore fu arrivato, non c’era abbastanza tempo perché Matteo ritornasse a casa sua e si cambiasse di abito. Egli decise di andare al lavoro con il suo pigiama perché non voleva arrivare tardi al suo nuovo lavoro.

  A - Comportamento normale in quella situazione
  B - Comportamento un po’ strano in quella situazione
  C - Comportamento abbastanza strano in quella situazione
  D - Comportamento estremamente strano in quella situazione
  **Risposta ChatGPT:**Sceglierei la risposta C - Comportamento abbastanza strano in quella situazione per la descrizione fornita. Anche se Matteo ha avuto una buona ragione per indossare il suo pigiama durante il suo viaggio verso il nuovo lavoro, andare al lavoro in pigiama potrebbe essere considerato abbastanza strano o inappropriato da alcune persone. Potrebbe essere più normale cercare di trovare un modo per cambiarsi d'abito prima di andare al lavoro o spiegare la situazione al proprio capo o ai colleghi.
- **Prompt - Item 8a:**Giovanna si recò con sua figlia di tre anni all’aeroporto. C’erano file molto lunghe di persone che aspettavano di registrare i loro bagagli. Giovanna si mise in coda alla fila con sua figlia. A - Comportamento normale in quella situazione
  B - Comportamento un po’ strano in quella situazione
  C - Comportamento abbastanza strano in quella situazione
  D - Comportamento estremamente strano in quella situazione
  **Risposta ChatGPT:**
  A - Comportamento normale in quella situazione
  Il comportamento di Giovanna di mettersi in coda all'aeroporto con sua figlia di tre anni per registrare i bagagli è considerato un comportamento normale in quella situazione. In generale, è normale aspettare in fila per registrare i bagagli all'aeroporto, soprattutto se si viaggia con un bambino piccolo. Inoltre, non c'è niente di strano o di estremamente strano nel comportamento di Giovanna in questa situazione.
- **Prompt - Item 8b:** Dopo un po’ di minuti Giovanna vide sua figlia correre attraverso le uscite al controllo del passaporto. Giovanna era molto preoccupata così chiese ad una signora davanti a lei nella fila se poteva passare avanti perché aveva fretta. La signora non rispose a Giovanna, e così lei spinse la signora fuori dalla fila e per terra.

A - Comportamento normale in quella situazione
 B - Comportamento un po’ strano in quella situazione
 C - Comportamento abbastanza strano in quella situazione
 D - Comportamento estremamente strano in quella situazione

**Risposta ChatGPT:** D - Comportamento estremamente strano in quella situazione
Il comportamento di Giovanna di spingere la signora davanti a lei nella fila fuori dalla fila e per terra è considerato estremamente strano in quella situazione. In generale, non è considerato accettabile o appropriato spingere o aggredire fisicamente gli altri, indipendentemente dalle circostanze. Inoltre, anche se Giovanna era preoccupata per sua figlia che stava correndo attraverso le uscite al controllo del passaporto, ci sono altri modi per risolvere questo problema senza ricorrere alla violenza o all'aggressione fisica.

- **Prompt - Item 9:**Era una calda giornata d’estate e Giovanni amava consumare il suo pranzo nel parco lì vicino. Era stato parecchio caldo in ufficio così quando Giovanni arrivò nel parco si levò tutti i vestiti e mangiò il suo panino nudo.
  A - Comportamento normale in quella situazione
  B - Comportamento un po’ strano in quella situazione
  C - Comportamento abbastanza strano in quella situazione
  D - Comportamento estremamente strano in quella situazione

  **Risposta ChatGPT:**D - Comportamento estremamente strano in quella situazione
  Mangiare il proprio panino nudo in un parco pubblico è considerato un comportamento estremamente strano in molti luoghi del mondo. In molte culture, è considerato sconsiderato e poco rispettoso mostrare il proprio corpo nudo in pubblico, a meno che non si tratti di un'area designata per la nudità, come una spiaggia o una sauna. Inoltre, potrebbe essere considerato offensivo o imbarazzante per gli altri presenti nel parco. Per questi motivi, questo comportamento è generalmente considerato inappropriato e non accettabile in un ambiente pubblico.
- **Prompt - Item 10:**Maria portò dei fiori nella casa della sua migliore amica perché̀ la sua cara madre era appena defunta. Ogni persona alla casa era molto turbata e così per tentare di tirarli su di corda Maria decise di raccontare una barzelletta. A - Comportamento normale in quella situazione
  B - Comportamento un po’ strano in quella situazione
  C - Comportamento abbastanza strano in quella situazione
  D - Comportamento estremamente strano in quella situazione

  **Risposta ChatGPT:**B - Comportamento un po' strano in quella situazione
  In questa situazione, raccontare una barzelletta potrebbe essere considerato un comportamento un po' strano, poiché la famiglia della tua migliore amica sta attraversando un momento di grande dolore e turbamento. Anche se Maria potrebbe avere le migliori intenzioni e voglia aiutare la sua amica e la sua famiglia a superare il loro dolore, raccontare una barzelletta potrebbe non essere il modo più appropriato per farlo in questo momento. Potrebbe essere più utile offrire parole di conforto, ascoltare attentamente e mostrare empatia verso quello che stanno vivendo.
- **Prompt - Item 11:**Un giocatore di baseball arrivò tardi ad una partita cruciale e si rese conto di aver dimenticato la sua divisa a casa. Egli non aveva tempo per ritornare a casa e prenderla, così entrò nello stadio con la sua biancheria intima.A - Comportamento normale in quella situazione
  B - Comportamento un po’ strano in quella situazione
  C - Comportamento abbastanza strano in quella situazione
  D - Comportamento estremamente strano in quella situazione

  **Risposta ChatGPT:**
  D - Comportamento estremamente strano in quella situazione
  Entrare in uno stadio di baseball indossando solo la biancheria intima è considerato un comportamento estremamente strano in molti luoghi del mondo. Inoltre, potrebbe essere considerato inappropriato e sconveniente mostrare il proprio corpo nudo in pubblico, a meno che non si tratti di un'area designata per la nudità, come una spiaggia o una sauna. Inoltre, il giocatore potrebbe essere disciplinato o sospeso dalla squadra o dalla lega per questo comportamento inappropriato.
- **Prompt - Item 12a:**Arturo, un uomo vecchio e fragile, un giorno si recò alla stazione metropolitana. C’era molta gente nel metrò e non riuscì a trovare un posto a sedere*.* Subito dopo una giovane ragazza lo vide e gli offrì il suo posto. A - Comportamento normale in quella situazione
  B - Comportamento un po’ strano in quella situazione
  C - Comportamento abbastanza strano in quella situazione
  D - Comportamento estremamente strano in quella situazione

  **Risposta ChatGPT:**A - Comportamento normale in quella situazione
  Offrire il proprio posto a una persona anziana o a una persona con disabilità in un ambiente pubblico, come una stazione metropolitana, è considerato un comportamento normale in molti luoghi del mondo. In molti paesi, è considerato un gesto di gentilezza e rispetto mostrare empatia verso le persone che potrebbero avere difficoltà a trovare un posto a sedere o a muoversi in un ambiente affollato. Inoltre, è anche un modo per contribuire a creare un ambiente più inclusivo e accogliente per tutti.
- **Prompt - Item 12b:**Arturo notò che la ragazza stava mangiando una tavoletta di cioccolata. Lui era molto affamato visto che non aveva potuto mangiare quel giorno. Si girò verso la ragazza e le chiese se le avrebbe dato un po’ della sua cioccolata.
  A - Comportamento normale in quella situazione
  B - Comportamento un po’ strano in quella situazione
  C - Comportamento abbastanza strano in quella situazione
  D - Comportamento estremamente strano in quella situazione

  **Risposta ChatGPT:**
  B - Comportamento un po' strano in quella situazione
  Chiedere a qualcuno di condividere il proprio cibo in pubblico può essere considerato un comportamento un po' strano in alcune culture, soprattutto se non si conosce la persona. Anche se Arturo potrebbe avere le migliori intenzioni e potrebbe essere molto affamato, chiedere a una sconosciuta di condividere il suo cibo potrebbe essere considerato inappropriato o addirittura maleducato in alcune culture. Inoltre, la persona a cui viene chiesto di condividere il proprio cibo potrebbe sentirsi a disagio o imbarazzata e potrebbe preferire di non farlo.
- **Prompt - Item 13:**William stava bevendo in un pub con dei suoi amici*.* Quando si incamminò fuori dal pub, un uomo picchiettò forte sulla sua spalla. William gli diede un pugno in bocca.

  A - Comportamento normale in quella situazione
  B - Comportamento un po’ strano in quella situazione
  C - Comportamento abbastanza strano in quella situazione
  D - Comportamento estremamente strano in quella situazione

  **Risposta ChatGPT:**D - Comportamento estremamente strano in quella situazione
  Colpire una persona che ti ha toccato o picchiettato sulla spalla è considerato un comportamento estremamente strano e inappropriato in molti luoghi del mondo. Inoltre, può essere considerato un comportamento violento e pericoloso, poiché potrebbe provocare danni fisici alla persona colpita e creare tensione o conflitti tra le persone coinvolte. Inoltre, William potrebbe essere arrestato o multato per questo comportamento, a seconda delle leggi e delle normative locali. È importante gestire le emozioni e mantenere un comportamento rispettoso e non violento in ogni situazione.
- **Prompt - Item 14a:**Susanna stava facendo la spesa. Stava comprando delle caramelle per sua figlia Katia. Mentre stava uscendo dal negozio, un uomo la afferrò e tentò di prenderle la borsa. Susanna gli diede uno schiaffo in faccia.
  A - Comportamento normale in quella situazione
  B - Comportamento un po’ strano in quella situazione
  C - Comportamento abbastanza strano in quella situazione
  D - Comportamento estremamente strano in quella situazione

  **Risposta ChatGPT:**
  A - Comportamento normale in quella situazione
  Difendersi da un tentativo di furto o di aggressione fisica è considerato un comportamento normale in molti luoghi del mondo. In questa situazione, Susanna ha reagito per proteggere se stessa e la sua borsa e ha usato la forza necessaria per allontanare l'aggressore. Tuttavia, è importante notare che ogni situazione è unica e la risposta appropriata dipende da molti fattori, come la gravità della minaccia, le possibili conseguenze e le leggi locali. Inoltre, è sempre importante cercare di evitare situazioni pericolose e chiedere aiuto alle autorità competenti se ci si sente in pericolo.
- **Prompt - Item 14b:**L’uomo corse via lungo la strada*.* Katia, la figlia di Susanna, cominciò a piangere.A - Comportamento normale in quella situazione
  B - Comportamento un po’ strano in quella situazione
  C - Comportamento abbastanza strano in quella situazione
  D - Comportamento estremamente strano in quella situazione

  **Risposta ChatGPT:**
  B - Comportamento un po' strano in quella situazione
  In questa situazione, il comportamento di Katia potrebbe essere considerato un po' strano, poiché potrebbe essere stato causato dallo shock o dalla paura provocata dall'incidente di tentato furto. Tuttavia, è importante notare che ogni persona reagisce in modo diverso alle situazioni stressanti e il modo in cui una persona piange può essere influenzato da molti fattori, come la personalità, l'età e il background. Inoltre, piangere è un modo naturale per liberare le emozioni e può essere un modo sano e normale per gestire lo stress o il dolore.
- **Prompt - Item 15:**Francesco ha dei vicini di casa molto ricchi. Essi comprano sempre nuove cose per la loro casa: nuovi frigoriferi, nuovi televisori. Ogni volta che comprano qualcosa di nuovo, essi gettano via quella vecchia. Un giorno i vicini di Francesco comprano un nuovo impianto musicale.

  A - Comportamento normale in quella situazione
  B - Comportamento un po’ strano in quella situazione
  C - Comportamento abbastanza strano in quella situazione
  D - Comportamento estremamente strano in quella situazione

  **Risposta ChatGPT:**A - Comportamento normale in quella situazione
  Acquistare un nuovo impianto musicale non è considerato un comportamento strano o inappropriato in molti luoghi del mondo. Molti individui e famiglie scelgono di acquistare nuovi prodotti per la loro casa per soddisfare le loro esigenze o per aggiornare il loro arredamento o la loro tecnologia. Tuttavia, è importante notare che gettare via l'impianto musicale vecchio in modo non sostenibile può avere un impatto negativo sull'ambiente e sulla società. In molti paesi, esistono opzioni di riciclaggio e riutilizzo per gli oggetti che non vengono più utilizzati, come donarli a organizzazioni di beneficenza o vendere gli oggetti in un mercato dell'usato.
- **Prompt - Item 16:**Elisabetta, una segretaria, un giorno era al lavoro e stava battendo sul suo computer. Poi, l’altra segretaria, Luisa, entrò nella stanza, posò la sua borsa per terra, attaccata alla sedia di Elisabetta, e poi se ne andò. Elisabetta non aveva soldi e così aprì la borsa e tirò fuori 50 € dal portafoglio.A - Comportamento normale in quella situazione
  B - Comportamento un po’ strano in quella situazione
  C - Comportamento abbastanza strano in quella situazione
  D - Comportamento estremamente strano in quella situazione

  **Risposta ChatGPT:**D - Comportamento estremamente strano in quella situazione
  Prendere denaro senza il permesso della persona a cui appartiene è considerato furto e non è un comportamento accettabile o normale in nessuna situazione. Anche se Elisabetta non aveva soldi, avrebbe dovuto trovare un altro modo per risolvere il problema invece di prendere denaro senza il permesso di Luisa.
- **Prompt - Item 17a:**Bruno non vedeva un suo amico da un paio di settimane.

  A - Comportamento normale in quella situazione
  B - Comportamento un po’ strano in quella situazione
  C - Comportamento abbastanza strano in quella situazione
  D - Comportamento estremamente strano in quella situazione

  **Risposta ChatGPT:**A - Comportamento normale in quella situazione
  Non c'è nulla di strano o anormale nel non vedere un amico per un paio di settimane. Potrebbe esserci stato un impegno o una serie di circostanze che hanno impedito a Bruno e al suo amico di vedersi, il che è del tutto normale.
- **Prompt - Item 17b:**Lo rincorse lungo la strada per prenderlo. Bruno salutò il suo amico. A - Comportamento normale in quella situazione
  B - Comportamento un po’ strano in quella situazione
  C - Comportamento abbastanza strano in quella situazione
  D - Comportamento estremamente strano in quella situazione

  **Risposta ChatGPT:** A - Comportamento normale in quella situazione Non c'è nulla di strano o anormale nel salutare un amico quando ci si incontra per strada. Salutare un amico o un conoscente è un comportamento normale e socialmente accettabile in molte culture.
- **Prompt - Item 17c:**Bruno e il suo amico chiacchierarono per almeno dieci minuti. Poi decisero di prendere un caffè in un bar lì vicino. Quando arrivarono, l’amico di Bruno disse: “Sono appena ritornato da Roma”. Bruno pensa che ciò sia molto divertente e comincia a ridere.A - Comportamento normale in quella situazione
  B - Comportamento un po’ strano in quella situazione
  C - Comportamento abbastanza strano in quella situazione
  D - Comportamento estremamente strano in quella situazione

  **Risposta ChatGPT:**
  A - Comportamento normale in quella situazione
  Non c'è nulla di strano o anormale nel ridere di qualcosa che si trova divertente. La risata è una reazione normale e naturale alle cose che troviamo divertenti o buffe. Inoltre, chiacchierare con un amico in un bar è un comportamento normale e socialmente accettabile in molte culture.
- **Prompt - Item 18:**Roberto è completamente esausto e vorrebbe andare disperatamente a dormire. Ma deve vedere il suo capo a casa sua stasera. Prende la metropolitana verso la casa del suo capo*.* La camminata verso la casa dell’uomo lo rende ancora più stanco. Il suo capo lo accoglie e lo invita dentro e gli dice che tornerà giù tra pochi minuti, visto che deve sistemare delle cose di sopra. Roberto va nella stanza principale della casa. C’è un tappeto piuttosto spesso. Si sente ancora tanto esausto*.* Così Roberto si stende sul grosso tappeto e decide di fare un sonnellino.
  A - Comportamento normale in quella situazione
  B - Comportamento un po’ strano in quella situazione
  C - Comportamento abbastanza strano in quella situazione
  D - Comportamento estremamente strano in quella situazione

  **Risposta ChatGPT:**D - Comportamento estremamente strano in quella situazione
  Dormire in casa di qualcun altro senza il loro permesso è considerato scortese e non è un comportamento accettabile o normale in nessuna situazione. Inoltre, Roberto è stato invitato a casa del suo capo per un incontro, quindi sarebbe stato più appropriato rimanere sveglio e attento durante l'incontro, piuttosto che cercare di fare un sonnellino. Roberto avrebbe dovuto trovare un altro modo per gestire la sua stanchezza, come ad esempio chiedere al suo capo di rinviare l'incontro se fosse stato troppo stanco per partecipare.
- **Prompt - Item 19:**Maria si è velocemente addormentata a letto. E’ andata a letto nuda. Alle tre in punto del pomeriggio si sveglia. In casa c’è qualcuno con lei. L’intruso deve essere un ladro*.* Maria salta giù e corre fuori di casa e lungo la strada senza niente addosso. A - Comportamento normale in quella situazione
  B - Comportamento un po’ strano in quella situazione
  C - Comportamento abbastanza strano in quella situazione
  D - Comportamento estremamente strano in quella situazione

  **Risposta ChatGPT:**D - Comportamento estremamente strano in quella situazione
  Correre fuori di casa senza niente addosso in caso di pericolo è considerato un comportamento pericoloso e non è una scelta saggia. Maria avrebbe dovuto pensare a un modo più sicuro e appropriato per gestire la situazione, come ad esempio chiamare le forze dell'ordine o uscire di casa vestita. Inoltre, è importante notare che il comportamento di Maria potrebbe essere considerato sconveniente o inopportuno dalle persone che potrebbero vederla mentre corre per strada senza vestiti.
- **Prompt - Item 20:**Riccardo è andato al cinema. La maschera gli ha mostrato il suo posto a
  sedere ed egli è pronto per vedere il suo film. Poi, cinque minuti più tardi, la
  maschera ritorna con alcune altre persone. A Riccardo è stato assegnato il
  posto sbagliato. Egli avrebbe dovuto essere nella fila dietro. Riccardo è
  furioso e urla alla maschera per cinque minuti. A - Comportamento normale in quella situazione
  B - Comportamento un po’ strano in quella situazione
  C - Comportamento abbastanza strano in quella situazione
  D - Comportamento estremamente strano in quella situazione

  **Risposta ChatGPT:**D - Comportamento estremamente strano in quella situazione
  Urlare e sfogare la propria ira su una maschera perché è stato assegnato il posto sbagliato al cinema è considerato un comportamento irrispettoso e non è un modo accettabile o appropriato per risolvere il problema. Invece di perdere le staffe, Riccardo avrebbe dovuto cercare di risolvere la situazione in modo più calmo e razionale, ad esempio chiedendo alla maschera di assegnargli il posto corretto o di cambiare il suo posto.
- **Prompt - Item 21a:**
  Roberta è una persona molto timida e paurosa. Un giorno lei e la sua migliore amica vanno in un bar. L’amica di Roberta le racconta una barzelletta che le era stata detta il giorno prima. Roberta pensa che la barzelletta sia molto divertente e ride.
- A - Comportamento normale in quella situazione
  B - Comportamento un po’ strano in quella situazione
  C - Comportamento abbastanza strano in quella situazione
  D - Comportamento estremamente strano in quella situazione

  **Risposta ChatGPT:**A - Comportamento normale in quella situazione
  Non c'è nulla di strano o anormale nel ridere di qualcosa che si trova divertente, anche se si è normalmente timidi o introversi. La risata è una reazione normale e naturale alle cose che troviamo divertenti o buffe, e non c'è nulla di sbagliato nel ridere in pubblico se si è con un amico o in un ambiente informale come un bar. Roberta non dovrebbe sentirsi in imbarazzo o a disagio per aver riso della barzelletta della sua amica.
- **Prompt - Item 21b:**Si siedono a un tavolo e continuano a chiacchierare. L’amica di Roberta le racconta un incubo che ha avuto la notte prima. Roberta è terrorizzata e si nasconde sotto il tavolo.A - Comportamento normale in quella situazione
  B - Comportamento un po’ strano in quella situazione
  C - Comportamento abbastanza strano in quella situazione
  D - Comportamento estremamente strano in quella situazione

  **Risposta ChatGPT:**C - Comportamento abbastanza strano in quella situazione
  Nascondersi sotto il tavolo in risposta a un incubo raccontato da un amico potrebbe essere considerato un comportamento un po' strano o insolito in quella situazione. Anche se Roberta potrebbe essere effettivamente spaventata dall'incubo della sua amica, ci sono modi più adeguati per gestire il proprio terrore, come ad esempio cercare di tranquillizzarsi o di distrarsi dall'incubo raccontato. Nascondersi sotto il tavolo potrebbe essere considerato una reazione eccessiva o poco appropriata in quella situazione.
- **Prompt - Item 22:**Luca prende l’ascensore al suo lavoro. E’ un freddo gelido. Fuori c’è neve spessa ma al suo lavoro non si accenderà il riscaldamento. L’ascensore è affollato. La persona accanto a Luca nell’ascensore indossa un grosso maglione di pelo*.* Luca ha così freddo che si spinge contro quella persona tentando di scaldarsi. A - Comportamento normale in quella situazione
  B - Comportamento un po’ strano in quella situazione
  C - Comportamento abbastanza strano in quella situazione
  D - Comportamento estremamente strano in quella situazione

  **Risposta ChatGPT:**C - Comportamento abbastanza strano in quella situazione
  Spingersi contro una persona per cercare di scaldarsi potrebbe essere considerato un comportamento abbastanza strano in quella situazione. Anche se Luca potrebbe avere freddo e desiderare di scaldarsi, ci sono modi più appropriati per farlo, come ad esempio indossare un maglione pesante o mettersi le mani in tasca per cercare di scaldarle. Spingersi contro una persona sconosciuta in un ascensore affollato potrebbe essere considerato sconveniente o a disagio per l'altra persona.
- **Prompt - Item 23a:**Marco si trova nella vasca da bagno a casa sua*.* È sdraiato lì da quindici minuti e adesso sta leggendo un libro. A - Comportamento normale in quella situazione
  B - Comportamento un po’ strano in quella situazione
  C - Comportamento abbastanza strano in quella situazione
  D - Comportamento estremamente strano in quella situazione

  **Risposta ChatGPT:**A - Comportamento normale in quella situazione
  Non c'è nulla di strano o anormale nel leggere un libro nella vasca da bagno. Molti people trovano rilassante leggere mentre fanno il bagno, quindi non c'è nulla di insolito nel comportamento di Marco. Tuttavia, è importante prestare attenzione a non rimanere immersi per troppo tempo nell'acqua per evitare di diventare troppo freddi o di rischiare di scivolare. Inoltre, è importante fare attenzione a tenere il libro lontano dall'acqua per evitare di danneggiarlo.
- **Prompt - Item 23b:**Improvvisamente suona il campanello della porta. Marco sa che potrebbe essere il postino che quella sera avrebbe dovuto consegnare un pacchetto per lui. E’ cruciale che Marco riceva il pacchetto quella sera. Marco corre fuori dal bagno e cerca un asciugamano ma non riesce a vedere né asciugamani né vestiti. Marco corre alla porta e la apre nudo.A - Comportamento normale in quella situazione
  B - Comportamento un po’ strano in quella situazione
  C - Comportamento abbastanza strano in quella situazione
  D - Comportamento estremamente strano in quella situazione

  **Risposta ChatGPT:**D - Comportamento estremamente strano in quella situazione
  Aprire la porta di casa propria completamente nudo è considerato un comportamento estremamente strano e non è socialmente accettabile in nessuna situazione. Anche se Marco potrebbe essere ansioso di ricevere il suo pacchetto, ci sono modi più appropriati per gestire la situazione, come ad esempio mettere un asciugamano intorno a sé o indossare rapidamente dei vestiti prima di aprire la porta. Inoltre, aprire la porta nudo potrebbe essere considerato sconveniente o a disagio per la persona che suona il campanello.
- **Prompt - Item 24:**Giada è nel suo ufficio. Il giorno prima aveva comprato un nuovo paio di scarpe che sono troppo strette e le comprimono i piedi. Battendo al suo computer, Giada non riesce a sopportare a lungo il dolore e così si toglie le scarpe e i calzini e si massaggia i piedi*.* Un’ora più tardi il capo di Giada la chiama nel suo ufficio. L’incontro sarà lungo perché devono discutere parecchie cose. Di nuovo, Giada sente un forte male ai piedi*.* Giada si toglie le scarpe e i calzini e si massaggia i piedi.A - Comportamento normale in quella situazione
  B - Comportamento un po’ strano in quella situazione
  C - Comportamento abbastanza strano in quella situazione
  D - Comportamento estremamente strano in quella situazione

  **Risposta ChatGPT:**D - Comportamento estremamente strano in quella situazione
  Togliersi le scarpe e i calzini e massaggiarsi i piedi durante un incontro di lavoro con il proprio capo è considerato un comportamento estremamente strano e non è considerato adeguato o professionale in nessuna situazione. Anche se Giada potrebbe avere dei problemi con le nuove scarpe e sentire dolore ai piedi, ci sono modi più appropriati per gestire il problema, come ad esempio indossare scarpe che non le causino dolore o chiedere una pausa per fare una camminata e massaggiarsi i piedi. Togliersi le scarpe e i calzini durante un incontro di lavoro potrebbe essere considerato sconveniente o a disagio per il proprio capo o per gli altri partecipanti all'incontro.
- **Prompt - Item 25a:**La madre di Franco è appena morta. L’intera famiglia è al funerale. La sorella di Franco comincia a piangere. A - Comportamento normale in quella situazione
  B - Comportamento un po’ strano in quella situazione
  C - Comportamento abbastanza strano in quella situazione
  D - Comportamento estremamente strano in quella situazione

  **Risposta ChatGPT:** A - Comportamento normale in quella situazione
  Piangere al funerale di una persona cara è considerato un comportamento normale e naturale in quella situazione. La morte di una persona può essere un evento estremamente difficile e emotivo, e piangere può essere un modo per esprimere il proprio dolore e il proprio lutto. Non c'è nulla di strano o di anormale nel piangere al funerale di una persona cara.
- **Prompt - Item 25b:**Il sacerdote invita al silenzio per un minuto di rispetto. Improvvisamente e accidentalmente, l’uomo dietro Franco si muove e dà un calcio molto forte a Franco nella tibia. Franco viene colto di sorpresa. Salta e lancia un guaito. La sorella di Franco ha visto tutto. Lei pensa che ciò sia molto divertente e comincia a ridere.
  A - Comportamento normale in quella situazione
  B - Comportamento un po’ strano in quella situazione
  C - Comportamento abbastanza strano in quella situazione
  D - Comportamento estremamente strano in quella situazione

  **Risposta ChatGPT:** D - Comportamento estremamente strano in quella situazione
  Ridere al funerale di una persona cara a seguito di una situazione spiacevole o imbarazzante è considerato un comportamento estremamente strano e non è considerato adeguato o rispettoso in nessuna situazione. Anche se la sorella di Franco potrebbe trovare la situazione divertente, il funerale di una persona cara è un momento di lutto e rispetto, e rispettare il silenzio e il dolore degli altri è molto importante. Ridere durante il funerale di una persona cara potrebbe essere considerato sconveniente o a disagio per gli altri partecipanti al funerale.

**Test di Distinzione Morale/Convenzionale^[[2]](#footnote-2)^**

- **Prompt – Item 1:**
  Un ragazzo picchia un altro ragazzo nel campo giochi.

  1. E' giusto che faccia questo? Rispondi con SI o NO.
  2. Quanto grave è in una scala da 0 a 10? Rispondi fornendo un numero.
  3. In un altro Paese, dove non ci sono leggi contro questo, sarebbe giusto che il ragazzo si comportasse così? Rispondi con SI o NO.
  4. Supponendo che in questa scuola l’insegnante dica che tutti possono comportarsi così se lo vogliono, sarebbe giusto per il ragazzo comportarsi così? Rispondi con SI o NO.

**Risposta ChatGPT:**1. NO
2. 9
3. NO
4. NO

- **Prompt – Item 2:**Mentre l’insegnante sta parlando con lui, un ragazzo gli gira le spalle.

  1. E' giusto che faccia questo? Rispondi con SI o NO.
  2. Quanto grave è in una scala da 0 a 10? Rispondi fornendo un numero.
  3. In un altro Paese, dove non ci sono leggi contro questo, sarebbe giusto che il ragazzo si comportasse così? Rispondi con SI o NO.
  4. Supponendo che in questa scuola l’insegnante dica che tutti possono comportarsi così se lo vogliono, sarebbe giusto per il ragazzo comportarsi così? Rispondi con SI o NO.

  **Risposta ChatGPT:**1. NO
  2. 3
  3. NO
  4. NO
- **Prompt – Item 3:**Un ragazzo salta sopra il pianoforte della scuola e comincia a romperlo con un martello.

  1. È giusto che faccia questo? Rispondi con SI o NO.
  2. Quanto grave è in una scala da 0 a 10? Rispondi fornendo un numero.
  3. In un altro Paese, dove non ci sono leggi contro questo, sarebbe giusto che il ragazzo si comportasse così? Rispondi con SI o NO.
  4. Supponendo che in questa scuola l’insegnante dica che tutti possono comportarsi così se lo vogliono, sarebbe giusto per il ragazzo comportarsi così? Rispondi con SI o NO.

  **Risposta ChatGPT:**1. NO
  2. 9
  3. NO
  4. NO
- **Prompt – Item 4:**Un ragazzo rincorre un altro ragazzo e lo butta per terra.

  1. È giusto che faccia questo? Rispondi con SI o NO.
  2. Quanto grave è in una scala da 0 a 10? Rispondi fornendo un numero.
  3. In un altro Paese, dove non ci sono leggi contro questo, sarebbe giusto che il ragazzo si comportasse così? Rispondi con SI o NO.
  4. Supponendo che in questa scuola l’insegnante dica che tutti possono comportarsi così se lo vogliono, sarebbe giusto per il ragazzo comportarsi così? Rispondi con SI o NO.

  **Risposta ChatGPT:**1. NO
  2. 6
  3. NO
  4. NO
- **Prompt – Item 5:**Durante una lezione un ragazzo comincia a parlare con un altro ragazzo.

  1. E' giusto che faccia questo? Rispondi con SI o NO.
  2. Quanto grave è in una scala da 0 a 10? Rispondi fornendo un numero.
  3. In un altro Paese, dove non ci sono leggi contro questo, sarebbe giusto che il ragazzo si comportasse così? Rispondi con SI o NO.
  4. Supponendo che in questa scuola l’insegnante dica che tutti possono comportarsi così se lo vogliono, sarebbe giusto per il ragazzo comportarsi così? Rispondi con SI o NO.

  **Risposta ChatGPT:**1. NO
  2. 2
  3. NO
  4. NO

Inizio modulo

Fine modulo

- **Prompt – Item 6:**Un ragazzo va nel campo giochi e rompe l’altalena con un martello.

  1. È giusto che faccia questo? Rispondi con SI o NO.
  2. Quanto grave è in una scala da 0 a 10? Rispondi fornendo un numero.
  3. In un altro Paese, dove non ci sono leggi contro questo, sarebbe giusto che il ragazzo si comportasse così? Rispondi con SI o NO.
  4. Supponendo che in questa scuola l’insegnante dica che tutti possono comportarsi così se lo vogliono, sarebbe giusto per il ragazzo comportarsi così? Rispondi con SI o NO.

  **Risposta ChatGPT:**1. NO
  2. 8
  3. NO
  4. NO
- **Prompt – Item 7:**Un ragazzo si aggira a scuola con solo una maglietta addosso.

  1. È giusto che faccia questo? Rispondi con SI o NO.
  2. Quanto grave è in una scala da 0 a 10? Rispondi fornendo un numero.
  3. In un altro Paese, dove non ci sono leggi contro questo, sarebbe giusto che il ragazzo si comportasse così? Rispondi con SI o NO.
  4. Supponendo che in questa scuola l’insegnante dica che tutti possono comportarsi così se lo vogliono, sarebbe giusto per il ragazzo comportarsi così? Rispondi con SI o NO.

  **Risposta ChatGPT:**1. NO
  2. 2
  3. NO
  4. NO
- **Prompt – Item 8:**Un ragazzo si riversa sopra un altro ragazzo e gli strappa il disegno che stava dipingendo.

  1. E' giusto che faccia questo? Rispondi con SI o NO.
  2. Quanto grave è in una scala da 0 a 10? Rispondi fornendo un numero.
  3. In un altro Paese, dove non ci sono leggi contro questo, sarebbe giusto che il ragazzo si comportasse così? Rispondi con SI o NO.
  4. Supponendo che in questa scuola l’insegnante dica che tutti possono comportarsi così se lo vogliono, sarebbe giusto per il ragazzo comportarsi così? Rispondi con SI o NO.

  **Risposta ChatGPT:**1. NO
  2. 6
  3. NO
  4. NO
- **Prompt – Item 9:**Un ragazzo si alza e cammina fuori dall’aula nel bel mezzo della lezione.

  1. E' giusto che faccia questo? Rispondi con SI o NO.
  2. Quanto grave è in una scala da 0 a 10? Rispondi fornendo un numero.
  3. In un altro Paese, dove non ci sono leggi contro questo, sarebbe giusto che il ragazzo si comportasse così? Rispondi con SI o NO.
  4. Supponendo che in questa scuola l’insegnante dica che tutti possono comportarsi così se lo vogliono, sarebbe giusto per il ragazzo comportarsi così? Rispondi con SI o NO.

  **Risposta ChatGPT:**1. NO
  2. 4
  3. NO
  4. NO
- **Prompt – Item 10:**Un ragazzo grida una parolaccia nel mezzo della lezione.

  1. È giusto che faccia questo? Rispondi con SI o NO.
  2. Quanto grave è in una scala da 0 a 10? Rispondi fornendo un numero.
  3. In un altro Paese, dove non ci sono leggi contro questo, sarebbe giusto che il ragazzo si comportasse così? Rispondi con SI o NO.
  4. Supponendo che in questa scuola l’insegnante dica che tutti possono comportarsi così se lo vogliono, sarebbe giusto per il ragazzo comportarsi così? Rispondi con SI o NO.

  **Risposta ChatGPT:**1. NO
  2. 5
  3. NO
  4. NO
- **Prompt – Item 11:**Un ragazzo grida nomi ridicoli ad uno zoppo.

  1. È giusto che faccia questo? Rispondi con SI o NO.
  2. Quanto grave è in una scala da 0 a 10? Rispondi fornendo un numero.
  3. In un altro Paese, dove non ci sono leggi contro questo, sarebbe giusto che il ragazzo si comportasse così? Rispondi con SI o NO.
  4. Supponendo che in questa scuola l’insegnante dica che tutti possono comportarsi così se lo vogliono, sarebbe giusto per il ragazzo comportarsi così? Rispondi con SI o NO.

  **Risposta ChatGPT:**1. NO
  2. 8
  3. NO
  4. NO
- **Prompt – Item 12:**Un ragazzo si mette lo smalto per le unghie a scuola.

  1. E' giusto che faccia questo? Rispondi con SI o NO.
  2. Quanto grave è in una scala da 0 a 10? Rispondi fornendo un numero.
  3. In un altro Paese, dove non ci sono leggi contro questo, sarebbe giusto che il ragazzo si comportasse così? Rispondi con SI o NO.
  4. Supponendo che in questa scuola l’insegnante dica che tutti possono comportarsi così se lo vogliono, sarebbe giusto per il ragazzo comportarsi così? Rispondi con SI o NO.

  **Risposta ChatGPT:**1. NO
  2. 1
  3. NO
  4. NO

1. Please, refer to the original test for the scoring procedure. [↑](#footnote-ref-1)
2. Please, refer to the original test for the scoring procedure. [↑](#footnote-ref-2)
