# Supplementary material for: Challenging large language models’ “intelligence” with human tools: A neuropsychological investigation in Italian language on prefrontal functioning
Source: Heliyon. 2024 Oct 3;10(19):e38911. doi: 10.1016/j.heliyon.2024.e38911 (PMC11490853; doi:10.1016/j.heliyon.2024.e38911)
Supplement: Multimedia component 2 [file mmc2.docx]

**Note:** This is an English Translation of the Italian prompts and ChatGPT’s responses, hence some translations may not accurately reflect the same meaning as in Italian.

**INDEX**

[Verbal Reasoning Test](#_Toc134467185)  [3](#_Toc134467185)

[*Absurdities*](#_Toc134467186) [*3*](#_Toc134467186)

[*Intruders*](#_Toc134467187) [*5*](#_Toc134467187)

[*Relationships*](#_Toc134467188) [*7*](#_Toc134467188)

[*Differences*](#_Toc134467189) [*9*](#_Toc134467189)

[*Idiomatic expressions*](#_Toc134467190) [*12*](#_Toc134467190)

[*Family Relations*](#_Toc134467191) [*14*](#_Toc134467191)

[*Classification*](#_Toc134467192) [*16*](#_Toc134467192)

[Cognitive Estimation Test](#_Toc134467193)  [17](#_Toc134467193)

[Metaphors Comprehension](#_Toc134467194)  [26](#_Toc134467194)

[Idiomatic Comprehensions](#_Toc134467195)  [32](#_Toc134467195)

[Anaphoric Referencing](#_Toc134467196)  [38](#_Toc134467196)

[Tower of London](#_Toc134467197)  [42](#_Toc134467197)

[Hayling Sentence Completion Test](#_Toc134467212)  [81](#_Toc134467212)

[Compound Remote Association Task](#_Toc134467213)  [84](#_Toc134467213)

[Social Cognition Battery](#_Toc134467214)  [88](#_Toc134467214)

[*Theory of Mind Test (TOM)*](#_Toc134467215) [*88*](#_Toc134467215)

[*Emotion Attribution Test*](#_Toc134467216) [*93*](#_Toc134467216)

[*Social Situation Test*](#_Toc134467217) [*106*](#_Toc134467217)

[*Moral/Conventional Distinction Test*](#_Toc134467218) [*121*](#_Toc134467218)

# **Verbal Reasoning Test**

(Basagni et al., 2017)

## **Absurdities**

- **Prompt - Item 1:**
  Every year Luisa organizes two birthday parties for her twin sons: one on July 15th and one on March 4th. What is the absurdity in this sentence?
  **ChatGPT response:**The absurdity present in the sentence you provided is that Luisa organizes two parties for the birthday of her twin sons. Most people throw one party on their birthday, while Luisa throws two. Furthermore, the two children have birthdays in two different months, so it is not possible that they both have birthdays on the day when the holidays are celebrated. In summary, the sentence presents a temporal and logical inconsistency.  **Score: 0**
- **Prompt - Item 2:**For the bicentenary of Alessandro Manzoni's birth, his son read some of his poems in public. What is the absurdity in this sentence?
  **ChatGPT response:**
  The absurdity present in the sentence you provided is that Alessandro Manzoni, who is the character to whom the bicentenary of his birth is dedicated, had a son. Alessandro Manzoni was a celebrated 19th century Italian writer and poet, so it would be impossible for him to have a son who is still alive in the present to read some of his poems in public on the bicentenary of his birth. In summary, the sentence has a temporal inconsistency.
  **Score: 2**
- **Prompt - Item 3:**Leonardo went out early this morning to buy a free book at the bookstore. What is the absurdity in this sentence?  **ChatGPT response:**The absurdity present in the sentence you provided is that Leonardo went to buy a free book in the bookstore. Usually, free books are offered free of charge and do not have to be purchased. Also, if a book is given free as a gift, it is usually given on special occasions, such as during an event or promotion, and is not sold in a bookstore. In summary, the sentence presents a logical inconsistency.
  **Score: 2**
- **Prompts - Item 4:**
  Having to carry 6 kg of rice in my suitcase, for fear that the luggage was too heavy, I put 3 in the front pocket and 3 in the back. What is the absurdity in this sentence?
  **ChatGPT response:**
  The absurdity present in the sentence you provided is the fact that you put 3 kg of rice in each of the pockets of the suitcase. Usually, the pockets of suitcases are designed to hold small items such as documents, wallets and mobile phones, not to hold significant amounts of food, such as 3kg of rice. Also, putting 3kg of rice in each of the pockets could make the suitcase too heavy and bulky, making it difficult to transport. In summary, the sentence presents a logical inconsistency.
  **Score: 0**
- **Prompt - Item 5:**
  Immediately after sunset, Luca took a nice swim in the sea and then lay down to dry in the sun on the beach. What is the absurdity in this sentence?
  **ChatGPT response:**There is no nonsense in the sentence you provided. The sentence simply describes the fact that Luca took a swim in the sea just after sunset and then lay down on the beach to sunbathe. There are no temporal or logical inconsistencies in the sentence.
  **Score: 0**
- **Prompt - Item 6:**
  During the last strike of postal workers, the membership was 100%. On that day the postman brought me fewer letters than usual. What is the absurdity in this sentence?
  **ChatGPT response:**
  The absurdity in the sentence you provided is the fact that during a strike by postal workers, membership was 100% yet the postman still brought you fewer letters than usual. During a strike, employees who join usually do not work and do not carry out their usual activities. If the strike is 100% adherent, one would expect no postal workers to work that day and therefore no letters to be delivered. In summary, the sentence presents a logical inconsistency.
  **Score: 2**
- **Prompts - Item 7:**
  The mule is a cross between a donkey and a mare and is sterile. Since it cannot reproduce , in the space of about a year, unfortunately there will not be even one left in Italy. What is the absurdity in this sentence?
  **ChatGPT response:**
  The absurdity present in the sentence you provided is the fact that, given that the mule is sterile and cannot reproduce, there won't be even one left in Italy within about a year. Usually, the mule is used as a pack or draft animal and its presence in an area is determined by the need of the local population to use it for these purposes, not by its ability to reproduce. Furthermore, even if the mule were indeed endangered in Italy, its disappearance would be unlikely to happen so quickly, in less than a year. In summary, the sentence presents a logical inconsistency.
  **Score: 0**

**Total score Absurdities subtest: 6/14**

## **Intruders**

- **Prompt - Item 1:**Genoa, Campania, Lucca, Turin. Which one is the intruder in this sentence?
  **ChatGPT response:**The intruder in this sentence is Campania. Genoa, Lucca and Turin are all Italian cities, while Campania is an Italian region. In summary, Campania is the object that does not belong to the same group or category as the other objects in the sentence.
  **Score: 2**
- **Prompt - Item 2:**Dog, cat, collar, turtle. Which one is the intruder in this sentence?
  **ChatGPT response:**The intruder in this sentence is the turtle. Dog and cat are common pets, while collar is an item that is used to train or identify animals. The tortoise, on the other hand, is an animal that is not usually considered a pet and does not wear collars. In summary, the turtle is the object that does not belong to the same group or category as the other objects in the sentence.
  **Score: 0**
- **Prompt - Item 3:**March, Monday, Friday, Wednesday. Which one is the intruder in this sentence?
  **ChatGPT response:**
  The intruder in this sentence is March. March is a month, while Monday, Friday and Wednesday are days of the week. In summary, March is the object that does not belong to the same group or category as the other objects in the sentence.
  **Score: 2**
- **Prompt - Item 4:**Tower, bishop, queen, checkmate. Which one is the intruder in this sentence?
  **ChatGPT response:**The intruder in this sentence is checkmate. Tower, bishop, and queen are all pieces in the game of chess, while checkmate is a situation or a move in the game. In summary, a checkmate is the object that does not belong to the same group or category as the other objects in the sentence.
  **Score: 2**
- **Prompts - Item 5:**
  Car, tractor, motorcycle, wheel. Which one is the intruder in this sentence?
  **ChatGPT response:**
  The intruder in this sentence is the wheel. Cars, tractors and motorcycles are all means of transport, while the wheel is a component of these vehicles. In summary, the wheel is the object that does not belong to the same group or category as the other objects in the sentence.
  **Score: 2**
- **Prompt - Item 6:**Skis, stadium, ball, rackets. Which one is the intruder in this sentence?
  **ChatGPT response:**The intruder in this sentence is the stadium. Skis, balls and rackets are all objects or equipment that are used to practice different sporting activities, while the stadium is a place where these activities are carried out. In summary, the stage is the object that does not belong to the same group or category as the other objects in the sentence.
  **Score: 2**
- **Prompt - Item 7:**Spades, cards, clubs, hearts. Which one is the intruder in this sentence?
  **ChatGPT response:**
  The intruder in this sentence is the cards. Spades, clubs, and hearts are all suits in a deck of playing cards, while cards are the backing upon which the face cards of the suits are printed. In summary, the cards are the object that does not belong to the same group or category as the other objects present in the sentence.
  **Score: 2**

**Total score Intruders subtest: 12/14**

**Relationships**

- **Prompts - Item 1:**
  Is the relationship between SELFISH and ALTRUISTIC like the relationship between MISER and ...?
  **ChatGPT Answer:**
  The relationship between selfish and altruistic is opposite, as is that between miserly and generous. An egoist is a person who thinks only of his own interests and does not care about others, while an altruist is a person who considers the interests of others as well as his own and cares about helping others. Similarly, the miser is a person who is attached to money and seeks to accumulate as many material possessions as possible, while the generous is a person who is willing to share his possessions with others and make donations to help others.
  **Score: 2**
- **Prompt - Item 2:**
  Is the relationship between BLACK and WHITE like that between HAPPY and ...?
  **ChatGPT Answer:**
  As I mentioned earlier, there is no specific relationship between black and white and happy and another term. Black and white are two colors that can be used to describe objects or situations, while happy is an emotion that can be felt by a person. There is no direct relationship between these two pairs of terms. However, in some contexts, black and white can be used as symbols of opposition or duality, such as good and evil or right and wrong. In this case, the relationship between black and white could be similar to that between happy and sad, as both pairs of terms represent opposite emotions or contrasting moods. However, it is important to note that this symbolic use of black and white is just a convention and that there is no inherent relationship between colors and emotions per se.
  **Score: 1**
- **Prompt - Item 3:**Is the relationship between WHEEL and BIKE like the one between HANDS and ...?
  **ChatGPT Answer:**
  The relationship between wheel and bicycle is similar to that between hands and clock. A wheel is a component of a bicycle that allows the vehicle to move. A bicycle is a form of transportation that uses two or more wheels to move. Hands are components of a watch that keep time by indicating the hour, minutes and seconds. A clock is an instrument that uses hands to measure and tell time. Thus, in both pairs of terms, one of the terms denotes a component or part of an object or system, while the other denotes the object or system as a whole.
  **Score: 2**
- **Prompt - Item 4:**Is the relationship between HAND and GLOVE like the one between HEAD and ...?
  **ChatGPT response:**
  The relationship between hand and glove is similar to that between head and hat. A hand is a part of the human body that is used to grasp, touch and manipulate objects. A glove is a garment that covers the hands and can be worn to protect the hands or to change their appearance. The head is the upper part of the human body which contains the brain, sensory organs and respiratory tract. A hat is a garment that covers the head and can be worn to protect the head or to change its appearance. Thus, in both pairs of terms, one of the terms means a body part or a component of a system, while the other means a garment or accessory that is worn to protect or modify the appearance of that body part or component.
  **Score: 2**
- **Prompt - Item 5:**Is the relationship between PERIMETER and SQUARE like the one between CIRCUMFERENCE and ...?  **ChatGPT response:**
  The relationship between perimeter and square is similar to that between circumference and circle. The perimeter of a square is the length of the outline of the square, i.e. the sum of the lengths of its sides. A square is a polygon with four congruent sides and four right angles. The circumference of a circle is the outline of the circle, i.e. the line that delimits the circle. A circle is a geometric figure made up of all points that are equidistant from a central point called the center. Thus, in both pairs of terms, one of the terms indicates the outline or perimeter of a geometric figure, while the other indicates the geometric figure itself.  **Score: 2**
- **Prompt - Item 6:**Is the relationship between CRAB and CLAWS like the one between OCTOPUS and ...?  **ChatGPT response:**
  The relationship between crab and claws is similar to that between octopus and tentacles. A crab is a marine animal that belongs to the crustacean group and has two claws, or two jointed pincers used to grasp and manipulate objects. An octopus is a marine animal that belongs to the cnidarian group and has many tentacles, i.e. many flexible organs equipped with stinging cells used to grasp and capture prey. Thus, in both pairs of terms, one of the terms denotes an organ or structure of an animal used to grasp or manipulate objects, while the other denotes the animal itself.  **Score: 2**
- **Prompt - Item 7:**Is the relationship between CHAIR and WOOD like the one between GLASS and ...?  **ChatGPT answer:**The relationship between chair and wood is similar to that between glass and glass. A chair is a piece of furniture made up of a wooden or other material structure on which one can sit. Wood is a material made up of plant tissue that is used to build objects or structures. A glass is a container made of glass or other transparent material that is used to hold and drink liquids. Glass is a material made up of fused silica that is used to make transparent objects or structures. Therefore, in both pairs of terms, one of the terms indicates an object or piece of furniture, while the other indicates the material of which it is made.
  **Score: 2**

**Total score Relationships subtest : 13/14**

## **Differences**

- **Prompts - Item 1:**
  What is the difference between shoes and slippers?
  **ChatGPT response:**
  Shoes and slippers are both garments that are worn on the feet to protect or to change their appearance. However, there are some differences between the two.
  One major difference is that shoes are clothing that is mostly worn outdoors, while slippers are clothing that is mostly worn indoors or indoors. Shoes are designed to protect feet from cold, dampness, abrasion and other environmental factors, while slippers are designed to protect feet from cold and dampness within an enclosed environment.
  Another difference is that shoes have a sturdier and more durable sole, while slippers have a softer and more flexible sole. The sole of the shoes is designed to provide good support and stability to the foot while walking, while the sole of the slippers is designed to provide comfort and to adapt to the shape of the foot.
  Finally, shoes come in a greater variety of shapes, styles, and colours than slippers, which often come in a simpler shape and more casual style. Shoes are worn on various occasions, such as work, leisure, parties, and so on, while slippers are more casual clothing and are mostly worn at home or indoors.
  In summary, shoes are more suitable for outdoor wear, have a sturdier and more durable sole, and come in a greater variety of shapes, styles and colours. Slippers are garments better suited for use indoors, have a softer, more flexible sole, and are generally more informal.
  **Score: 2**
- **Prompts - Item 2:**
  What is the difference between a sofa and an armchair?
  **ChatGPT Answer:**In summary, the main difference between a sofa and an armchair is that the sofa is a piece of furniture that is longer and wider than an armchair, and therefore can accommodate more people. A sofa is a piece of furniture for the living room or for another room in the house which consists of a wide and deep seat, usually with backrests and armrests, and which can have one or more seats. An armchair, on the other hand, is a piece of furniture for the living room or for another room in the house which consists of a smaller and shallower seat than the sofa, usually with a backrest and armrests, and which can accommodate only one person.

Another difference is that sofas are generally more suited to being used as seating for long conversations or watching TV, while armchairs are more suited to being used as seating for resting or reading a book. Sofas usually have a more regular shape and a more formal style than armchairs, which can have more particular shapes and more informal styles.

Additionally, sofas can come in a greater variety of shapes, styles, and sizes than armchairs, which often come in simpler shape. Sofas can be made in a variety of materials, such as wood, metal, fabric or leather, while armchairs are usually made in fabric or leather.

In summary, the sofa is a larger piece of furniture that is more suitable for accommodating more people, is generally more suitable for use as seating for long conversations or for watching TV, and can come in a greater variety of shapes, styles and size. The armchair is a smaller piece of furniture and more suitable for accommodating a single person, it is generally more suitable for use as a seat to rest or to read a book, and usually has a simpler shape.
**Score: 2**

- **Prompt - Item 3:**
  What is the difference between a pencil and a pen?
  **ChatGPT response:**
  Pencils and pens are both used for writing, drawing or sketching but there are some differences between the two.

One of the main differences is the material from which they are made. Pencils are made with a lead of graphite or other writing material enclosed in a wooden or other material barrel. Pens are made with a metal or plastic tip that is soaked in ink or other writing material.

Another difference is how they are used. Pencils need to be sharpened with a pencil sharpener before use to achieve a fine and precise point, pens do not require this. Also, pencils can be easily erased using an eraser, while pens cannot be erased easily and permanently.

Finally, there is a difference in the duration of writing. Pencils can last a long time with moderate use, but lead can wear out if you write excessively or make heavy use of erasing. Pens can last a long time if you use ballpoint or nib pens with quality ink, but they can run out of ink faster if you write too much or use low-quality ballpoints or low-quality ink. quality.

**Score: 2**

- **Prompts - Item 4:**
  What is the difference between a pianist and a tenor?
  **ChatGPT response:**
  The difference between a pianist and a tenor is that the pianist plays the piano, while the tenor is a singer who sings in the higher range of male voices, ranging in texture from around the G note to high C. The tenor is usually associated with leading roles in operas, while the pianist can play a variety of musical genres.
  **Score: 2**
- **Prompts - Item 5:**
  What is the difference between a mirror and glass?
  **ChatGPT Answer:**The main difference between a mirror and glass is that a mirror reflects light, while glass lets it through. The mirror consists of a reflective surface, usually of silver, gold or aluminum, while glass is a transparent and opaque material.

Another difference between mirror and glass is their transparency. Mirror is opaque so you cannot see through it, while glass is transparent so you can see through it.

Also, mirror is usually lighter and more fragile than glass, as it consists of a reflective surface on a light support, while glass is usually heavier and more robust as it is made of a solid, transparent material.
**Score: 2**

- **Prompt - Item 6:**
  What is the difference between a lunch and a dinner?
  **ChatGPT answer:**
  The main difference between a lunch and a dinner is the time at which they are consumed. Lunch is usually the main meal eaten during the day, while dinner is usually the main meal eaten in the evening. However, these definitions can vary according to each individual's culture and eating habits.

Also, the content of a lunch and dinner can vary according to personal preferences and cultural traditions. For example, in some cultures lunch may be a light meal, while dinner may be more substantial, while in other cultures, lunch may be the largest meal of the day. Also, some cultures might include brunch, which is a meal eaten early in the morning or mid-morning, as a combination of breakfast and lunch.
**Score: 2**

- **Prompts - Item 7:**
  What is the difference between toothpaste and shampoo?

**ChatGPT Answer:**
The main difference between toothpaste and shampoo is that toothpaste is used to clean the teeth and oral cavity, while shampoo is used to cleanse and moisturize the hair.

Toothpaste usually contains chemicals like fluoride, which helps prevent tooth decay, and sodium percarbonate, which helps remove plaque and tartar from your teeth. Shampoo usually contains surfactants, which help remove dirt and oil from the hair, and moisturizing agents, which help keep the hair soft and hydrated.

Also, toothpaste is used internally, while shampoo is used externally on the hair and scalp. Toothpaste is applied to the toothbrush and used to clean the teeth, while the shampoo is applied to wet hair and rinsed off after massaging it into the scalp.

**Score: 2**

**Total score Differences subtest: 14/14**

## **Idiomatic expressions**

- **Prompts - Item 1:**
  What does " at each death of pope " mean?
  **ChatGPT response:**
  "At each death of pope" is a figure of speech meaning "very rarely" or "very seldom". The pope is the head of the Catholic Church, and his election and term of office are regarded in many respects as extraordinary and unique events. Therefore, the phrase "at each death of pope" is used to mean that something happens very rarely or very rarely, similar to the English expression "once in a blue moon".

For example, one could say "I go to the cinema every time dad dies", to indicate that one rarely goes to the cinema. Or you could say "I eat pork every time a pope dies", to indicate that pork is eaten very rarely.
**Score: 2**

- **Prompts - Item 2:**
  What does it mean to "be born with a shirt"?
  **ChatGPT answer:**"To be born with a shirt" is a figure of speech that means "to be successful or lucky from birth". The phrase "born with the shirt" refers to the idea that some people are fortunate to have been born into a wealthy or high social class family, or to have natural talents or opportunities that make them particularly fortunate from birth.

For example, one could say "He was born with a shirt, he always got everything he wanted" to indicate that someone has always been successful and lucky since birth. Or one could say "She was born with a shirt, she has a natural talent for music" to indicate that someone was successful from birth because of a natural talent.
**Score: 2**

- **Prompts - Item 3:**
  What does it mean “to be at green"?
  **ChatGPT response:**

"To be at green" is a idiom that means "to be broke or in financial difficulty." The phrase "broke" refers to the idea that lack of money or financial hardship is similar to a situation of poverty or destitution, as if one were "broke" or without resources.

For example, you might say "I'm at green, I don't have the money to pay the rent" to indicate that you're struggling financially and don't have the money to pay your bills. Or you could say "I'm at green, I don't have the money to buy a train ticket" to indicate that you are out of money and don't have the money to make a specific purchase.

**Score: 2**

- **Prompt - Item 4:**
  What does it mean to "throw in the towel"?
  **ChatGPT response:**
  "Throwing in the towel" is a figure of speech meaning "give up" or "give up". The phrase "throwing in the towel" refers to the idea of giving up an activity or commitment, similar to how you would throw away a used towel or sponge in surrender or defeat.

For example, you might say "I've decided to throw in the towel, I can't finish this puzzle" to indicate that you give up on completing an activity. Or you could say "He threw in the towel, he couldn't bear the pain" to indicate that someone has given up on continuing to endure a difficult situation.
**Score: 2**

- **Prompt - Item 5:**
  What does it mean to "have a green thumb"?
  **ChatGPT Answer:**
  "Having a green thumb" is a saying that means "having a natural talent for growing plants and flowers". The phrase "having a green thumb" refers to the idea that some people have an innate ability to make plants grow and thrive, as if they have a magic thumb or Midas touch for cultivation.

For example, one might say "He has a green thumb, he can grow anything" to indicate that someone has a natural talent for growing plants. Or you could say "I don't have a green thumb, I can never grow anything" to indicate that you are less good at growing plants.
**Score: 2**

- **Prompt - Item 6:**
  What does "spit the toad" mean?
  **ChatGPT response:**"Spit the toad" is a figure of speech that means "to tell the truth" or "to confess something difficult or embarrassing". The phrase "spitting the toad" refers to the idea of expelling something unpleasant or unwanted, as if spitting a toad out of the mouth.

For example, you might say "I decided to spit the toad, I lied about my age" to indicate that you are determined to confess a lie or a difficult truth. Or you could say "I can't spit the toad, I'm afraid of the consequences" to indicate that you are having difficulty confessing something embarrassing or uncomfortable.
**Score: 2**

- **Prompts - Item 7:**
  What does it mean to "fall from the frying pan into the fire"?
  **ChatGPT response:**"Fall from the frying pan into the fire" is a saying that means "to go from a difficult situation to an even worse one". The phrase "falling from the frying pan into the fire" refers to the idea of going from an awkward or problematic situation to an even more difficult or painful one, as if from a hot frying pan to a hot embers.

For example, you might say, "I fell from the frying pan into the fire, lost my job, and now have no money to pay the rent" to indicate that you've moved from a difficult situation to an even worse one. Or you could say "I was already sick, then I fell from the frying pan into the fire, they diagnosed me with a serious illness" to indicate that we have moved from an already problematic situation to an even more difficult one.
**Score: 2**

**Total score Idiomatic Expressions subtest: 14/14**

## **Family Relations**

- **Prompts - Item 1:**
  Franco and Maria are brother and sister. Maria is Luca's mother and Franco is Marco's father. What is the relationship between Luca and Marco?
  **ChatGPT response:**
  Luca and Marco are cousins. Maria and Franco are brother and sister, therefore Luca and Marco have the same grandfather and grandmother. Furthermore, Franco is Marco's father, so Luca and Marco also have the same paternal uncle. Therefore, Luca and Marco are cousins, i.e. they have a kinship relationship linked by one or more degrees of consanguinity.
  **Score: 2**
- **Prompts - Item 2:**
  Mario and Luisa are Matteo's parents. Paolo is Luisa's father. What is the relationship between Matteo and Paolo?
  **ChatGPT response:**
  Matteo and Paolo have a relationship of grandfather and grandson. Luisa is Matteo's mother and Paolo is Luisa's father, therefore Paolo is Matteo's grandfather. Therefore, Matteo and Paolo have a grandfather-grandson relationship, i.e. they have a kinship relationship linked by two degrees of consanguinity.
  **Score: 2**
- **Prompts - Item 3:**
  Nina and Roberto are Carlo's parents. Carlo is married to Simona. What is the relationship between Nina and Simona?
  **ChatGPT response:**
  Nina and Simona have a mother-in-law and daughter-in-law relationship. Nina is Carlo's mother, therefore she is Simona's mother-in-law. Simona is Carlo's wife, therefore she is Nina's daughter-in-law. Therefore, Nina and Simona have a relationship of mother-in-law and daughter-in-law, i.e. they have a family relationship linked by the marriage of Carlo and Simona.
  **Score: 2**
- **Prompts - Item 4:**
  Ettore is Anna's husband. Anna's parents are Giuseppe and Iole. What is the relationship between Ettore and Iole?
  **ChatGPT response:**
  Ettore and Iole have a son-in-law and mother-in-law relationship. Ettore is Anna's husband, therefore he is Iole's son-in-law. Iole is Anna's mother, therefore she is Ettore's mother-in-law. Therefore, Ettore and Iole have a son-in-law and mother-in-law relationship, that is, they have a family relationship linked by the marriage of Anna and Ettore.
  **Score: 2**
- **Prompts - Item 5:**
  Loredana is married to Vittorio. Vittorio has two brothers: Daniele and Simone. What is the relationship between Loredana and Simone?
  **ChatGPT response:**
  Loredana and Simone have a sister-in-law and brother-in-law relationship. Loredana is married to Vittorio, so she is Simone's sister-in-law. Simone is Vittorio's brother, therefore he is Loredana's brother-in-law. Therefore, Loredana and Simone have a relationship of sister-in-law and brother-in-law, i.e. they have a kinship relationship linked by the marriage of Loredana and Vittorio.
  **Score: 2**
- **Prompts - Item 6:**
  Rocco is Dino's father. Dino is Rosario's father and Rosario is Francesco's father. What is the relationship between Rocco and Francesco?
  **ChatGPT response:**
  Rocco and Francesco have a great-grandfather and great-grandson relationship. Rocco is Dino's father, therefore he is Rosario's grandfather. Rosario is Francesco's father, so he is Francesco's grandfather. Therefore, Rocco is Francesco's great-grandfather, and Francesco is Rocco's great-grandson. The relationship between Rocco and Francesco is therefore that of great-grandfather and great-grandson, or rather they have a kinship relationship linked by three degrees of consanguinity.
  **Score: 1**
- **Prompts - Item 7:**
  Paolo is Michele's brother. Michele is Sara's father. What is the relationship between Sara and Paolo?
  **ChatGPT response:**
  Sara and Paolo have an aunt and niece relationship. Paolo is Michele's brother, therefore he is Sara's uncle. Sara is Michele's daughter, therefore she is Paolo's granddaughter. Therefore, Sara and Paolo have an aunt-nephew relationship, i.e. they have a kinship relationship linked by a degree of consanguinity.
  **Score: 2**

**Total score Family Relations subtest : 13/14**

## **Classification**

- **Prompts - Item 1:**
  In one word, what are a plier, a screwdriver, a hammer?
  **ChatGPT Answer:**
  A Plier, a screwdriver and a hammer are tools.
  **Score: 2**
- **Prompt - Item 2:**
  In one word, what are ball, doll, skittle?
  **ChatGPT Answer:**
  Ball, doll and skittle are toys.
  **Score: 2**
- **Prompt - Item 3:**
  In one word, what are lipstick, eyeshadow, powder?
  **ChatGPT Answer:**
  Lipstick, eyeshadow and powder are cosmetics.
  **Score: 2**
- **Prompt - Item 4:**
  In one word, what are love, sadness, anger?
  **ChatGPT Answer:**
  Love, sadness and anger are emotions.
  **Score: 2**
- **Prompt - Item 5:**In one word, what are wardrobe, bed, table?
  **ChatGPT response:**
  Wardrobe, bed and table are furniture.
  **Score: 2**
- **Prompts - Item 6:**
  In a single word, what are Piedmont, Liguria, Lazio?
  **ChatGPT response:**
  Piedmont, Liguria and Lazio are regions.
  **Score: 2**
- **Prompts - Item 7:**
  In one word, what are rain, snow, hail?
  **ChatGPT response:**
  Rain, snow and hail are precipitation.
  **Score: 2**

**Total score Classification subtest: 14/14**

# **Cognitive Estimates Test**

(Della Sala, MacPherson et al., 2003)

- **Prompts - Item 1:**
  How much does a pair of heels weigh?
  **ChatGPT response:**
  In general, a pair of heels is likely to weigh anywhere from a few ounces to a few pounds. Please note that this is only a rough estimate and the actual weight of a pair of heels can vary widely depending on specific factors such as material of construction, size and design.

**Abs. scores: 1**

**Bizz . Score: 0**

- **Prompt - Item 2:**
  What is the area of a double bed sheet?

**ChatGPT Answer:**The area of a double bed sheet depends on the size of the mattress it is designed for. In general, double-fitted sheets are designed to fit mattresses with a width of approximately 135-140 centimetres and a length of approximately 190-200 centimetres. Please note that these are approximate dimensions only and double mattresses and sheets may vary slightly in size. If you want to calculate the area of a double bed sheet, simply multiply the width by the length. For example, if a double bed sheet has a width of 135 centimetres and a length of 190 centimetres, the area will be approximately 25,650 square centimetres.
**Abs. Score: 0**
**Bizz . Score: 0**

- **Prompt - Item 3:**How tall is a traffic light?
  **ChatGPT Answer:**
  Traffic lights have different heights depending on where they are and their purpose. In general, however, a traffic light is likely to be between 3 and 6 meters high. The tallest traffic lights are usually those that are in locations where they are visible from a greater distance, such as at a busy road intersection or near a highway. Conversely, the lowest traffic lights are usually those found in locations where they are visible from a shorter distance, such as in a park or residential area. Keep in mind that these are only rough estimates, and the actual height of a traffic light can vary widely depending on its location and purpose.
  **Abs. Score: 0
  Bizz . Score: 0**
- **Prompt - Item 4:**How much does a men's cotton t-shirt weigh?
  **ChatGPT Answer:**
  The weight of a men's cotton t-shirt depends on many factors, such as size, fabric and design. In general, however, a men's cotton shirt is likely to weigh anywhere from a few pounds to a few pounds, depending on size and fabric. Heavier cotton T-shirts are usually those made with thicker fabrics or special designs that require the use of more material. Conversely, lighter cotton T-shirts are usually those made with thinner fabrics or with simpler designs that require the use of less material. Please note that these are only rough estimates and the actual weight of a men's cotton t-shirt can vary widely depending on the specifics of the fabric and design.
  **Abs. Score: 2
  Bizz . Scores: 1**
- **Prompt - Item 5:**How much does a horse weigh?
  **ChatGPT Answer:**
  The weight of a horse depends on its breed, height and gender. In general, however, an adult horse is likely to weigh anywhere from a few hundred kilograms to over a ton, depending on its breed and height at the withers. The heaviest horses are usually those of larger breeds, such as draft horses or working saddle horses, while the lightest horses are usually those of smaller breeds, such as ponies. Keep in mind that these are only rough estimates, and a horse's actual weight can vary widely depending on its breed, height, and gender.
  **Abs. Score: 0
  Bizz . Scores: 1**
- **Prompt - Item 6:**How long is a human spine?
  **ChatGPT Answer:**
  A human spine is made up of approximately 33 vertebrae, extending from the base of the skull down to the pelvis. In general, a human’s spine is likely to be between 60 and 70 centimetres long, depending on his height and build. Keep in mind that this is only a rough estimate, and the actual length of a human's spine can vary widely depending on the specific factors that affect his physical conformation.
  **Abs. Score: 2**
  **Bizz . Score: 0**
- **Prompt - Item 7:**What is the maximum distance that can be covered in Sicily in km?
  **Answer:**
  Sicily is the largest island in the Mediterranean, with an area of about 25,000 square kilometers. If you want to calculate the maximum distance you can travel in Sicily in kilometres, you should keep in mind that the island has an irregular shape with many headlands and inlets. Consequently, the maximum distance you can travel in Sicily depends on the route you choose to follow.
  If you want to calculate the maximum distance that can be traveled in Sicily following the coast of the island, you should keep in mind that the length of the Sicilian coast is approximately 10,000 kilometers. Keep in mind that this is only a rough estimate and the actual distance you can travel in Sicily depends on the route you choose to take and the condition of the roads and means of transport used.
  **Abs. Score: 2
  Bizz . Scores: 1**
- **Prompt - Item 8:**After the water boils what is the minimum time needed to cook a hard-boiled egg?
  **ChatGPT Answer:**
  The time it takes to cook a hard boiled egg depends on many factors, such as the size of the egg, the temperature of the water and the desired degree of hardness. In general, however, the minimum time it takes to cook a hard-boiled egg is likely to be around 6-7 minutes. To boil an egg, you must first bring the water to a boil and then dip the egg into the boiling water. After soaking the egg, you need to boil it for the desired time. If you want a slightly soft boiled egg, you should boil it for about 6 minutes. If, on the other hand, you want a harder boiled egg, you should boil it for about 7-8 minutes. Keep in mind that these are only rough estimates, and the actual time it takes to cook a hard-boiled egg can vary widely depending on the specific factors that affect how well the egg cooks.
  **Abs. Score: 0
  Bizz . Score: 0**
- **Prompt - Item 9:**How many grams of pasta are needed for 4 people?
  **ChatGPT Answer:**The amount of pasta you need for 4 people depends on the type of pasta, the seasoning, and your individual preferences regarding how much pasta to consume. In general, however, around 200-250 grams of pasta is likely to be needed for 4 people. If you want to be more specific, you can base the amount of pasta on the recommended portion of about 50 grams of pasta per person. Keep in mind that this is only a rough estimate, and the actual amount of pasta needed for 4 people can vary widely depending on the type of pasta, seasoning, and individual preferences regarding how much pasta to consume.
  **Abs. Score: 1
  Bizz . Score: 0**
- **Prompt - Item 10:**How many weeks are there in a year?
  **ChatGPT Answer:**There are 52 weeks in a year. Each week has 7 days, for a total of 365 days in the year (or 366 days in leap years). Keep in mind that weeks start on Monday and end on Sunday, so there are always 52 weeks in a year, no matter how many days there are in the year.
  **Abs. Score: 0
  Bizz . Score: 0**
- **Prompt - Item 11:**How many camels are there in Italy?
  **ChatGPT response:**In Italy there are few camels, as these animals are native to the arid and desert regions of the Middle East and Africa. However, some camels can be found in Italy as amusement animals or as show animals, especially during the holiday season or in some theme parks. There is no precise estimate of the number of camels present in Italy, but it is likely that they are only a few hundred . Please note that this is only a rough estimate and the actual number of camels in Italy may vary.
  **Abs. Score: 2
  Bizz . Score: 0**
- **Prompt - Item 12:**How many people can travel on a bus during a rush hour?
  **ChatGPT Answer:**
  The maximum capacity of a coach depends on the model and size of the vehicle. In general, however, a coach is likely to seat around 30 to 60 people, depending on its size and the safety regulations in place. Please note that this is only a rough estimate and the actual capacity of a coach can vary widely depending on vehicle model and size. In addition, during peak hours the number of people who can travel on a coach can be influenced by the level of crowding of the vehicle and by the social distancing regulations in place to prevent the spread of infections.
  **Abs. Score: 1
  Bizz . Score: 0**
- **Prompts - Item 13:**
  How many seats are there on a 10-car train?
  **ChatGPT response:**
  The capacity of a train depends on the model and size of the carriages. In general, however, a 10-car train is likely to seat around 200 to 400 passengers, depending on its size and current safety regulations. Please note that this is only a rough estimate and the actual capacity of a train can vary widely depending on the model and size of the carriages. Also, the number of seats available on a train can be influenced by the level of vehicle crowding and by social distancing regulations in place to prevent the spread of infections.
  **Abs. Score: 1
  Bizz . Score: 0**
- **Prompts - Item 14:**
  How many eyelashes are in the lower eyelid?
  **ChatGPT Answer:**
  The amount of eyelashes present on the lower eyelid depends on many factors, such as gender, age and physical conformation. In general, however, there are likely to be about 80 to 100 eyelashes on the lower eyelid of each eye. Keep in mind that this is only a rough estimate, and the actual amount of lashes on your lower lid can vary widely depending on specific factors affecting your physique. Eyelashes are small structures on the lower and upper eyelids that protect the eye from the entry of foreign bodies and bright light.
  **Abs. Score: 1
  Bizz . Score: 0**
- **Prompts - Item 15:**
  How fast can a swallow fly in km/h?
  **ChatGPT Answer:**
  Swallows are very fast birds and can fly at speeds of up to 80 km/h or even more, depending on the species and conditions. However, these speeds are typically only achieved in peak acceleration or while hunting. The normal flight speed of a swallow is usually between 30 and 60 km/h. In general, swallows are able to move quickly through the air due to their light body structure and the streamlined shape of their wings.
  **Abs. Score: 0
  Bizz . Score: 0**
- **Prompts - Item 16:**
  How many kg can a weightlifter lift?
  **ChatGPT Answer:**
  The amount of weight a lifter is able to lift depends on several factors, such as their strength, technique, physical structure and the weight category in which they compete. In general, weightlifters compete in specific weight categories, with maximum weight limits varying by category. For example, in the men's "heavyweight" category (which includes lifters weighing over 105kg), participants can lift extremely large amounts of weight, often in excess of 300kg. However, even lower-category lifters are capable of lifting significant amounts of weight, with some managing to lift over 200kg. In general, weightlifting athletes are extremely strong and capable of lifting substantial amounts of weight, although the exact amount depends on the specific lifter and their condition.
  **Abs. Score: 0
  Bizz . Score: 0**
- **Prompts - Item 17:**
  How long is a passenger carriage of a train?
  **ChatGPT Answer:**
  The length of a passenger car of a train can vary depending on the type of train and the specifications of the car. However, in general, the passenger carriages of a train are around 20-25 meters long. This is the typical length of passenger carriages used in long-distance trains, such as those used to carry passengers over long distances. Passenger carriages of local trains or subway trains may be slightly shorter, with a length of around 15-20 metres. However, even these carriages can be slightly longer depending on the specifics of the train and the needs of the transport service.
  **Abs. Score: 0
  Bizz . Score: 0**
- **Prompts - Item 18:**
  How many litres of water does it take to fill a bathtub?
  **ChatGPT Answer:**
  The amount of water it takes to fill a bathtub depends on the size of the tub and how much water you want to fill. In general, to fill a standard-size bathtub, which has a capacity of around 150 litres, you will need around 150 litres of water. However, if you only want to fill the tub partially, you will need less water. Also, if you use an oversized bathtub or a tub of an unusual shape or size, it will take more or less water to fill it. In any case, it is important to consider that water consumption can vary according to individual habits and needs, so the amount of water needed to fill a bathtub can vary significantly from person to person.
  **Abs. Score: 1
  Bizz . Score: 0**
- **Prompts - Item 19:**
  How long does it take a young man to walk one kilometre?
  **ChatGPT Answer:**
  A person's walking speed can vary depending on many factors, such as their age, fitness, the terrain they walk on, and the distance they need to walk. In general, however, a young man in good physical shape may be able to walk one kilometer in about 15-20 minutes, at a walking speed of about 4-5 km/h. However, this is only an approximate value, and a person's actual walking speed can vary widely. Also, it is important to consider that walking speed may vary depending on terrain conditions and individual needs. For example, you may need to walk slower uphill or on uneven ground, while you may be able to walk faster on flat ground or downhill.
  **Abs. Score: 1**
  **Bizz . Score: 0**
- **Prompt - Item 20:**How many matches does a matchbox hold?
  **ChatGPT response:**
  The number of matches contained in a box depends on the type of box and the manufacturer. In general, matchboxes can hold as few as about 10 to as many as about 50 matches, but these figures can vary depending on the size of the box and the amount of matches the manufacturer chooses to fit in it. It's important to read the label or manufacturer's instructions to find out the exact amount of matches in a specific box.
  **Abs. Score: 1
  Bizz . Score: 0**
- **Prompt - Item 21:**Approximately how many coffees does a service station bartender make in one hour during rush hour?
  **ChatGPT response:**
  It is difficult to make a rough estimate of how many coffees a service station bartender can prepare in an hour of time during rush hour, as it depends on a number of factors such as the type of coffee machine used, the speed of the the number of customers requesting coffee and any interruptions or requests for other types of drinks or food. However, bearing these factors in mind, it is likely that a bartender could prepare a minimum of about 20 and a maximum of about 100 coffees in an hour of time during peak hour, although this range may vary based on the circumstances specifications. However, this is only a rough estimate and there may be a great deal of variability in the actual amount of coffee brewed in an hour of time during rush hour.
  **Abs Score: 1**

**Bizz . Score: 0**

**Total Abs. Score: 17/41**

**Total Bizz . Score: 3/21**

# **Understanding of Metaphors**

( Papagno et al. 1995)

- **Prompt - Item 1:**What does "that schoolboy is an jerk" mean?
  **ChatGPT response:**
  "That schoolboy is an ass" means that the person referred to is stupid or clumsy. It is an idiom that is used to indicate that someone has difficulty understanding or learning something or that they frequently make mistakes or wrong actions. Keep in mind that this expression can be considered offensive or derogatory, so you should avoid using it if you don't want to hurt someone's feelings.

**Scoring:**Slow to learn, ignorant, stubborn **2** Listless, negligent **1
Score: 2**

- **Prompt - Item 2:**What does "that train is a snail" mean?
  **ChatGPT response:**
  "That train is a snail" means that the train in question is travelling slowly, possibly due to a slowdown due to technical problems or adverse weather conditions. It is a way of saying that is used to indicate that something or someone is moving slowly or inefficiently. Keep in mind that this expression is only a figure of speech and should not be taken literally: trains are not animals and cannot have the physical characteristics of snails.
  **Scoring:**Slow **2** Concrete examples **1
  Score: 2**
- **Prompt - Item 3:**What does "the people waiting were on tenterhooks" mean?
  **ChatGPT response:**
  "The people waiting were on tenterhooks" means that the people in question were very nervous or agitated because they had to wait for something important or uncertain. The phrase "on tenterhooks" refers to being anxious or tense about a stressful or uncertain situation. Keep in mind that this expression is just a figure of speech and shouldn't be taken literally: people can't have physical spines like animals.
  **Scoring:**Anxious, apprehensive, agitated, uneasy **2** Impatient **1
  Score: 2**
- **Prompt - Item 4:**What does "today I feel like a lion" mean?
  **ChatGPT response:**
  Today I feel like a lion" means that the person speaking feels strong, energetic and ready to face any challenge. It is a saying that is used to indicate a positive state of mind and great self- confidence . Please note that this expression is only a figure of speech and should not be taken literally: people cannot have the physical characteristics of lions or feel like them.
  **Scoring:**Strong, brave **2** Strong or brave **1
  Score: 1**
- **Prompt - Item 5:**What does "business is booming" mean?
  **ChatGPT response:**
  "Business is booming" means that the businesses or projects referred to are doing very well and are successful. It is a saying that is used to indicate that something is progressing in a positive and satisfactory way. The expression "booming" refers to the image of a ship's sails billowing in the wind, indicating that the journey is underway and that one is moving forward with strength and determination. Keep in mind that this expression is just a figure of speech and shouldn't be taken literally: Business has no physical sails that can billow.
  **Scoring:**Favorably **2** According to own wishes/expectations **1
  Score: 2**
- **Prompt - Item 6:**What does "they're trying to blow smoke over my eyes" mean?
  **ChatGPT response:**
  “They are trying blow smoke over my eyes” means that someone is trying to confuse or deceive the person being referred to, into believing something that is not true or withholding important information. It is a figure of speech that is used to indicate that someone is trying to manipulate or deceive others to gain an advantage or to avoid facing responsibilities. Keep in mind that this expression is just a figure of speech and shouldn't be taken literally: no one can physically blow smoke over someone else's eyes.
  **Scoring:**Deceive, deceive with false appearances **2** Confuse, cheat, hide **1
  Score: 1**
- **Prompt - Item 7:**What does "that man has a heart of stone" mean?
  **ChatGPT response:**
  "That man's heart of stone" means that the man in question is insensitive or emotionless, possibly due to a cold personality or a difficult past. It is a idiom that is used to indicate that someone is difficult to move or to engage emotionally. Keep in mind that this expression is just a figure of speech and shouldn't be taken literally: people don't have physical hearts of stone.
  **Score Attribution:**Callous, inflexible, ruthless **2** Bad, wicked **1
  Score: 2**
- **Prompt - Item 8:**What does "that singer's success was a fire in straw" mean?
  **ChatGPT response:**
  "The success of that singer was a fire in the straw" means that the success achieved by the singer in question did not last long or was not lasting. It is a saying that is used to indicate that something has had a temporary or ephemeral success, which has not been able to last over time. The phrase "fire in the straw" refers to the image of a fire that is lit quickly but goes out just as quickly, indicating that something had a lively start but was destined to end soon. Keep in mind that this expression is only a figure of speech and should not be taken literally: a person's success cannot be compared to a physical fire.
  **Scoring:**Intense and short-lived phenomenon **2** Intense and short-lived phenomenon **1
  Score: 1**
- **Prompt - Item 9:**What does "that horse is lightning" mean?
  **ChatGPT response:**
  "That horse is lightning" means that the horse in question is very fast or agile. It is a way of saying that is used to indicate that something or someone moves quickly or with great agility. The phrase "lightning bolt" refers to the image of a bolt of lightning, traveling through the air at extremely high speed, indicating that something or someone is very fast. Keep in mind that this expression is only a figure of speech and should not be taken literally: horses cannot have the physical characteristics of lightning bolts.
  **Scoring:**Fast, rapid, impetuous **2** Concrete example (a trotter) **1
  Score: 2**
- **Prompts - Item 10:**

What does "my plans have gone up in smoke" mean?

**ChatGPT response:**

"My projects have gone up in smoke" means that the projects referred to have been halted or failed, possibly due to problems or unexpected events. It is a idiom that is used to indicate that something has failed or been destroyed. The phrase "going up in smoke" refers to the image of smoke rapidly dissipating into the air, indicating that something has vanished or been destroyed. Keep in mind that this expression is only a figure of speech and should not be taken literally: projects cannot physically go up in smoke.
**Scoring:**Fails, fails, fails, vanishes **2** Fails **1**  **Score: 2**

- **Prompts - Item 11:**

What does “this tie is an eyesore” mean?
**ChatGPT response:**

"This tie is an eyesore" means that the tie in question is very eye-catching or flamboyant, possibly due to its size or colors. It is a idiom that is used to indicate that something is too flashy or distracting to the eye. The phrase "eyesore" refers to the image of a fist hitting the eye, indicating that something is bothersome or painful to the eye. Keep in mind that this expression is only a figure of speech and should not be taken literally: neckties are not like physical fists.
**Scoring:**Inconsistent, incompatible, out of tune **2** Ugly, awful, tasteless, flashy **1
Score: 1**

- **Prompts - Item 12:**

What does "Franco is the pillar of the company" mean?
**ChatGPT response:**
"Franco is the pillar of the company" means that Franco is a very important or indispensable person for the proper functioning of the company, probably due to his skills or experience. It is a saying that is used to indicate that someone is fundamental or indispensable to the success of an activity or project. The phrase "pillar" refers to the image of a load-bearing column, which supports a building and makes it stable, indicating that someone is critical to the smooth running of something. Keep in mind that this expression is only a figure of speech and should not be taken literally: people cannot be compared to physical pillars.
**Scoring:**Support, cornerstone **2** Indispensable, essential, point of reference **1
Score: 1**

- **Prompts - Item 13:**

What does "John is my right hand man" mean?
**ChatGPT response:**
"John is my right hand man" means that John is someone you trust very much and who supports you in your endeavors or activities. In other words, John is an important person to you and plays a vital role in your work or personal pursuits. The term "right-hand man" is often used to describe someone who plays an important role in another person's life, as someone who can be trusted and relied upon.
**Scoring:**Most effective help, main help, main collaborator **2** Help, collaborator, trusted man **1
Score: 2**

- **Prompt - Item 14:**What does "criticism takes this book to the stars" mean?
  **ChatGPT response:**
  "Critics skyrocket this book" means that book or literary critics have expressed very positive opinions about this book and have given it enthusiastic praise. In other words, critics gave the book very positive reviews and considered it a work of a high standard. This may indicate that the book is well written, has an interesting storyline, or has deep or cultural meaning. Criticism can also be a quality indicator for readers, as critics are often literature savvy and have expert knowledge of the craft. If the critics bring a book to the stars, this can be a good sign for those who are interested in reading it.
  **Scoring:**Celebrate, exalt, magnify, exalt **2** Praise, judge well **1
  Score: 2**
- **Prompt - Item 15:**What does "that musician goes against the stream" mean?
  **ChatGPT response:**
  "Going against the stream" means doing something that goes against the norms or expectations of society or the majority. In the case of a musician, it could mean that he makes music that deviates from popular genres or fashions, or that he expresses ideas or points of view that go against cultural norms or dominant opinions. It could also mean that he stands out for his originality or creativity, that he does not follow established trends or patterns. In this way, the musician goes against the tide of the majority or the norm and stands out as an independent or innovative artist.
  **Scoring:**Goes against fashion, against the common trend **2** Original, innovative **1
  Score: 2**
- **Prompt - Item 16:**What does "that minister is a weather vane" mean?
  **ChatGPT response:**
  "To be a weather vane" means to be unstable or inconstant in one's ideas or behaviors. In the case of a minister, it could mean that he often changes his opinion on political issues or that he is indecisive or uncertain in the decisions he takes. It could also mean that he is easily influenced by other people or external events, or that he doesn't follow a coherent political line. In this way, the minister is described as a weather vane, that is, as a person who is not firmly anchored in one position or vision, but who easily changes direction according to circumstances.
  **Scoring:**Fickle, changing sides or opinion **2** Inconsistent **1
  Score: 2**
- **Prompt - Item 17:**What does "that soldier is a rabbit" mean?
  **ChatGPT response:**
  "Be a rabbit" can have different connotations, depending on the context in which it is used. In general, it could mean being shy or fearful, or having a submissive or compliant attitude. In the case of a soldier, it could mean that he is not brave or determined in combat, or that he does not face any challenges or dangers he encounters with determination. It could also mean that he is unable to withstand the pressure or stress of war situations, or that he does not steadfastly follow orders or directions from his superiors. In this way, the soldier is described as a rabbit, that is, as a person who is unable to face the difficulties or dangers he encounters with determination or courage.
  **Score Attribution:**Cowardly, cowardly, shy **2** Not fond of war **1
  Score: 2**
- **Prompt - Item 18:**What does "that schoolboy is a top" mean?
  **ChatGPT response:**
  "To be a top" means to be very good or gifted at something. In the case of a schoolboy, it could mean that he is highly intelligent or has done very well in school. It could also mean that he has a good memory or understanding, or that he is particularly interested or engaged in studying. In this way, the pupil is described as a top, that is, as a person who has excellent qualities or who is particularly good at a certain subject or activity.
  **Scoring:**Eminent, Excellent, Excellent **2** Good, Intelligent **1
  Score: 2**
- **Prompt - Item 19:**What does "The only salvation is to put out to sea" mean?
  **ChatGPT response:**
  "Put out to sea" means moving away from a situation or place, often to escape something or to seek a solution or a way out. In the case where it is said that "the only salvation is to put out to sea", it is suggesting that the best solution to face a difficult situation or to avoid problems is to get away from it. It could mean, for example, that the person in that situation has to leave the place where he is or that he has to look for other opportunities or other places to go. In this way, "putting out into the deep" is presented as the only possible solution or as the only way out of a problematic situation.
  **Scoring:**Flee, move away, go away **2** Avoid, dodge **1
  Score: 2**
- **Prompt - Item 20:**What does "the police know how to make the guilty sing" mean?
  **ChatGPT response:**
  "Making the guilty sing" means obtaining confessions or important information from people who are found guilty of some crime or wrongdoing. In case you say that "Police know how to make the guilty sing", you are suggesting that the Police have effective techniques or methods to get people to confess to their crimes or reveal important information. It could mean, for example, that the police use interrogation or psychological pressure techniques to obtain confessions or that they use threats or promises to obtain information. In this way, the Police is presented as an organization capable of obtaining confessions or important information from people who are found guilty of crimes or wrongdoing.
  **Scoring:**Confess, get secrets out, get people talking **2** Know the truth **1
  Score: 2**

**Total Understanding of Metaphors: 35/40**

# **Comprehension of Idiomatic Expressions^[[1]](#footnote-1)^**

( Papagno et al. 1995)

- **Prompt - Item 1:**What does "That woman takes the bread out of her mouth for her children" mean?
  **ChatGPT response:**
  "To take the bread out of one's mouth" means to deprive oneself of something essential or important to someone else. In the case where it is said that "that woman takes the bread out of her mouth for the children", it is suggesting that the woman gives up something important to herself to ensure the well-being or livelihood of her children. It could mean, for example, that the woman works hard to earn money for the family and has no time or energy for herself , or that she gives up opportunities or comforts to ensure the well-being of her children. In this way, the woman is described as a person who is willing to make sacrifices for others and to put her children's interests first.
  **Scoring:**Deprive yourself of the essentials **2** Deprive yourself of food, give up something **1
  Score: 2**
- **Prompt - Item 2:**What does it mean "in the face of these difficulties you have to put your hands in your hair"? **ChatGPT response:**
  "To put your hands in your hair" is a saying that means to be very worried or frustrated when faced with a difficult or complex situation. In case you say that "these difficulties are hard to pull your hair out of," you are suggesting that the situation is very complex or problematic and requires a great deal of concern or effort to deal with. It could mean, for example, that the situation is very stressful or presents insurmountable obstacles or difficulties. In this way, "throw your hair" is used to express frustration or a sense of helplessness in the face of a difficult or complex situation.
  **Scoring:**Despair **2** Not knowing how to solve problems **1
  Score: 2**
- **Prompt - Item 3:**What does it mean to say, "Antony goes down at breakneck (speed) from the top of the mountain"?
  **ChatGPT response:**
  "Going down at breakneck (speed)" means moving quickly and impetuously, often without taking the necessary precautions or precautions. In the case where it is said that "Antony is going breakneckly down from the top of the mountain," it is suggesting that Antony is moving quickly and without paying attention to dangers or obstacles that might be along the way. It could mean, for example, that Antony is coming down the mountain with haste or recklessness, or that he is approaching a situation with boldness or recklessness. In this way, "going down at breakneck speed" is used to describe fast or impetuous behavior that could involve risks or negative consequences.
  **Scoring:**Recklessly, hastily **2** To be able to break one's neck, quickly **1
  Score: 2**
- **Prompt - Item 4:**What does "What misfortune happens between my head and neck" mean?
  **ChatGPT response:**
  "Heads and necks" is a idiom that means "all together" or "for no reason". In the case where you say "What misfortune is happening to me", you are expressing frustration or concern about a negative or unfortunate event that happened for no apparent reason or without anything being done to avoid it. It could mean, for example, that you have suffered unexpected loss or damage, or that you have been involved in an unpleasant situation without meaning to. Thus, "between head and neck" is used to describe a negative or unfortunate event that happened for no apparent reason or without anything being done to avoid it.
  **Scoring:**Suddenly, unexpected **2** Like something that comes to your head **1
  Score: 2**
- **Prompt - Item 5:**What does "the director is a handy person" mean?
  **ChatGPT response:**
  "Being handy" means being easy to deal with or approach, or being available and willing to help others. In case you say "the manager is a handy person" you are suggesting that the manager is someone who is easy to approach or easy to talk to, or who is available and willing to help others. It could mean, for example, that the manager is someone who listens to the requests or concerns of others and tries to find solutions or provide support. Thus, "handy" is used to describe a person who is easy to deal with or approach and who is open and willing to help others.
  **Scoring:**Affable, courteous, cordial **2** Unafraid, simple **1
  Score: 2**
- **Prompt - Item 6:**What does "Giorgio has holey hands" mean?
  **ChatGPT response:**
  "To have holey hands" means to be very wasteful or have a lot of debt. In the case where it is said that "George has holey hands", it is suggesting that George has a high level of expenses or that he has a lot of debts to pay. It could mean, for example, that George has difficulty managing his money or that he spends much more than he earns. Thus, "having holey hands" is used to describe a person who has difficulty managing their money or who has a lot of debt to pay.
  **Scoring:**Wasteful, prodigal, squanderer **2** Overspending **1
  Score: 1**
- **Prompt - Item 7:**What does it mean to say, "Thieves are making havoc in the store"?
  **ChatGPT response:**
  "Making havoc" means taking everything you can, often in a disorderly way or with no respect for other people's property. In the case where you say "thieves make havoc", you're suggesting that the thieves are taking whatever they can from the store, probably in a disorderly fashion or with no respect for the store's or owner's property. It could mean, for example, that thieves are stealing a lot of items or money from the store indiscriminately, or that they are causing damage or disorder during the theft. Thus, "making havoc" is used to describe disorderly or disrespectful behavior in which one takes all one can with no respect for the property of others.
  **Scoring:**Steal all, devastate **2** Steal **1
  Score: 1**
- **Prompt - Item 8:**What does “That country is out of the way” mean?
  **ChatGPT response:**
  "Being out of the way" means being hard to reach or easy to reach. In case you say that "that country is out of the way", you are suggesting that the country is difficult to reach or that it is not easily accessible. This could mean, for example, that the country is in a remote area or that it is difficult to reach by public transport. Thus, "being out of the way" is used to describe a place that is difficult to reach or is not easily accessible.
  **Scoring:**Far, inconvenient, difficult to reach **2** Not connected with transport, down the road **1
  Score: 2**
- **Prompt - Item 9:**What does "Before you believe you have to touch" mean?
  **ChatGPT response:**
  "To touch" means to have a direct or personal experience of something, often to be sure or confirm something. In the case in which it is said "Before believing you must touch", it is suggesting that it is necessary to do a direct or personal experience of something in order to be able to be sure or confirm what you are saying or suspect.This could mean, for example, that you need to see or touch something yourself in order to be sure or that you need to directly experience something in order to fully understand its meaning or value. Thus, "touch" is used to describe the importance of having a direct or personal experience of something in order to have the certainty or confirmation
  **Scoring:**Ascertain, make sure **2** See what's going on, want to know **1
  Score: 2**
- **Prompt - Item 10:**What does "the new car costs an eye of the head" mean?
  **ChatGPT response:**
  "Cost an eye of the head " is a saying that means to be very expensive. In case you say that "the new car costs an eye of the head," you are suggesting that the new car is very expensive. It could mean, for example, that the new car is a luxury or that it has a price tag that represents a large expense for the person who wants to buy it. In this way, "cost an eye of the head" is used to describe an item or service that has a very high price.
  **Scoring:**Very much, big money **2** Being expensive **1
  Score: 2**
- **Prompt - Item 11:**What does it mean "better to avoid look conspicuous"?
  **ChatGPT response:**
  "Conspicuous" means to attract the attention or interest of other people in a negative or inappropriate way. In case you say "better to avoid being conspicuous," you're suggesting that it's best to avoid attracting other people's attention or interest in a negative or inappropriate way. It could mean, for example, that it's best to avoid doing or saying things that might look bad or that might be considered inappropriate. Thus, "conspicuous" is used to describe behavior or action that attracts the attention or interest of other people in a negative or inappropriate way.
  **Scoring:**Get noticed, expose yourself, put yourself in sight **2** Get seen **1
  Score: 2**
- **Prompt - Item 12:**What does "Adversity make me jump the fly on my nose" mean?
  **ChatGPT response:**
  "Jump fly on your nose" is a idiom that means to be very annoyed or exasperated. In the case where you say, "Adversity makes me jump the fly on my nose" you are suggesting that the adversity or obstacles you encounter cause severe irritation or exasperation. It could mean, for example, that the difficulties or unexpected events you encounter are very stressful or cause great frustration. Thus, "jumping the fly on my nose" is used to describe severe irritation or exasperation caused by difficulties or unexpected events.
  **Scoring:**Getting angry, annoyed, pissed off **2** Getting annoyed, angry about nothing **1
  Score: 2**
- **Prompt - Item 13:**What does "you risk being left with a handful of flies" mean?
  **ChatGPT response:**
  "Being left with a handful of flies" is a saying that means getting little or no results or benefits from an action or effort. In case you say "you risk being left with a handful of flies", you are suggesting that there is a risk of getting little or no results or benefits from an action or effort. It could mean, for example, that there is a risk of not getting what you hoped for or that there is a risk of not getting any benefit or gain from what you are doing. Thus, "being left with a handful of flies" is used to describe the risk of getting little or no results or benefits from an action or effort.
  **Scoring:**Empty-handed, disappointed, unsuccessful **2** Not grabbing what you want, not successful **1
  Score: 2**
- **Prompt - Item 14:**What does "you put a flea in my ear" mean?
  **ChatGPT response:**
  "Put the flea in your ear" means to suggest or arouse an idea or a doubt that remains pending. In the event that you say "you put a flea in my ear", it is suggesting that someone has suggested or raised an idea or a doubt that remains pending in the speaker's mind. It could mean, for example, that someone has raised an issue or problem that needs further thought, or that has piqued an interest or curiosity about something. In this way, "put the flea in your ear" is used to describe the fact of suggesting or eliciting an idea or a doubt that remains pending.
  **Scoring:**Insinuating doubt, perplexing **2** Doubting, unable to decide, alerting **1
  Score: 2**
- **Prompt - Item 15:**What does "the spy must come clean" mean?
  **ChatGPT response:**
  "Come clean" is a figure of speech that means to confess or reveal all that one knows. In the case where you say "the spy must come clean," you are suggesting that the spy must confess or reveal everything he knows. It could mean, for example, that the spy must provide information or secrets that he has collected or that he must admit his role or actions. Thus, "spill the beans" is used to describe confessing or revealing all that one knows.
  **Scoring:**Confessing, speaking, revealing what has been seen **2** Being sincere **1
  Score: 2**
- **Prompt - Item 16:**What does "Franco goes home empty-handed" mean?
  **ChatGPT response:**
  "Going home empty-handed" is a saying that means going home without success or without having achieved what one had hoped for. In case you say "Franco goes home empty-handed", you are suggesting that Franco went home unsuccessfully or without having achieved what he had hoped for. It could mean, for example, that Franco returned home without having achieved the goal he set for himself or that he returned home disappointed or defeated. Thus, "going home empty-handed" is used to describe returning home without success or without having achieved what one had hoped for.
  **Scoring:**Empty-handed, disappointed, humiliated, dejected **2** Without positive results **1
  Score: 1**
- **Prompt - Item 17:**What does "There are four cats in the theater today" mean?
  **ChatGPT response:**
  "There are four cats" is a saying that means that there are few people or that there is a low turnout. In the case where you say "There are four cats in the theater today", you are suggesting that there are few people in the theater today or that there is a low turnout. It could mean, for example, that there are few spectators or that the theater is not busy. Thus, "there are four cats" is used to describe a low turnout or small amount of people.
  **Scoring:**Very few people **2** Four people, empty **1
  Score: 2**
- **Prompt - Item 18:**What does "I give you carte blanche for this project" mean?
  **ChatGPT response:**
  "Giving carte blanche" means giving total freedom or authority to someone to do something. In case you say "I give you carte blanche for this project", you are suggesting that you are giving someone total freedom or authority to do something related to a specific project. It could mean, for example, that you are giving someone the ability to act without restrictions or to make their own decisions. Thus, "giving carte blanche" is used to describe giving total freedom or authority to someone to do something.
  **Scoring:**Give full power, freedom of action **2** Delegate, let you do what you want **1
  Score: 2**
- **Prompt - Item 19:**What does "George has a straw tail" mean?
  **ChatGPT response:**
  "Have a straw tail" is a figure of speech that means to be easily discovered or exposed. In case you say "George has a straw tail", you are suggesting that George is easily discovered or exposed. It could mean, for example, that George has difficulty hiding or lying or that he is easily caught when he commits an act or crime. Thus, "having a straw tail" is used to describe being easily discovered or exposed.
  **Scoring:**Being at fault, suspicious **2** Feeling guilty **1
  Score: 0**
- **Prompt - Item 20:**What does "You have to go with leaden feet" mean?
  **ChatGPT response:**
  "To go with leaden feet" is a saying that means to be cautious or careful about what you do. In case you say "You have to go with leaden feet", you're suggesting that you need to be cautious or careful about what you do. It could mean, for example, that you need to act carefully or with circumspection or that it is important to carefully weigh the consequences of your actions. In this way, "to go with leaden feet" is used to describe being cautious or careful about what you do.
  **Scoring:**Carefully, cautiously, cautiously **2** Go slowly, think carefully **1
  Score: 2**

**Total score of Comprehension of Idiomatic Expressions: 36/40**

**Anaphoric Referencing**

(Sartori et al., 2022; in press)

Note: For a visualization purpose, in bold the correct response. However, not any type of formatting cue was given to ChatGPT in the testing phase.

- **Prompt - Item 1:**
  Paolo tried to call Giorgio on the phone, but he was not available.
  Question: Who was 'not available'?

A. Paolo

**B. Giorgio**

**ChatGPT response:**
The correct answer is B. Giorgio was not available.

**Score: 1**

- **Prompt - Item 2:**
  In the storm, the tree fell and broke through the roof of my house. Now, I have to remove it.
  Question: What 'must be removed'?

**A. The tree**

B. The roof

**ChatGPT response:** The correct answer is A. The tree must be removed.
**Score: 1**

- **Prompt - Item 3:**Everyone liked the almond cookies while only a few people liked the cinnamon cookies. Next time we should do less.
  Question: 'What kind of cookies should we make less of next time'?

A. Almond biscuits

**B. Cinnamon Cookies**

**ChatGPT response:**
The correct answer is B. We should make less cinnamon cookies next time.

**Score: 1**

- **Prompt - Item 4:**Tommaso gave Raffaele a lift to school so he wouldn't have to walk alone.
  Question: Who 'shouldn't have walked alone' ?

A. Tommaso

**B. Raffaele**

**ChatGPT response:**
The correct answer is B. Raffaele shouldn't have walked alone.

**Score: 1**

- **Prompt - Item 5:**Anna asked Maria what time the library closes, because she forgot.
  Question: Who 'forgot it'?

A. Mary

**B. Anna**

**ChatGPT response:**
The correct answer is B. Anna has forgotten what time the library closes.
**Score: 1**

- **Prompt - Item 6:**The sack of potatoes had been placed on top of the sack of flour, so it had to be moved first. Question: What 'should be moved first'?

**A. The sack of potatoes**

B. The sack of flour

**ChatGPT response:**
The correct answer is A. The sack of potatoes had to be moved first.
**Score: 1**

- **Prompt - Item 7:**Journalists have interviewed the protagonists of the new film. They were very insistent, so the interview lasted a long time.

Question: Who 'was very pushy'?

**A. Journalists**

B. The protagonists

**ChatGPT response:**
The correct answer is A. The journalists have been very insistent.
**Score: 1**

- **Prompt - Item 8:**Everyone liked the almond cookies, while only a few people liked the cinnamon cookies. Next time we should make more.

Question: 'What kind of cookies should we make more of next time'?

**A. Almond biscuits**

B. Cinnamon Cookies

**ChatGPT response:**
The correct answer is A. We should make more almond cookies next time.
**Score: 1**

- **Prompt - Item 9:**Journalists have interviewed the protagonists of the new film. They were very cooperative, so the interview lasted a long time.
  Question: Who 'was very cooperative'?

A. Journalists

**B. The protagonists**

**ChatGPT response:**
The correct answer is B. The protagonists were very cooperative.
**Score: 1**

- **Prompt - Item 10:**The man could not lift his son because he was very weak.
  Question: Who 'was weak'?

**A. The man**

B. The son

**ChatGPT response:**
The correct answer is A. The man was weak.
**Score: 1**

- **Prompt - Item 11:**The sack of potatoes was placed below the sack of flour, so it had to be moved first.

Question: What 'should be moved first'?

A. The sack of potatoes

**B. The sack of flour**

**ChatGPT response:**
The correct answer is A. The sack of potatoes had to be moved first.
**Score: 0**

- **Prompt - Item 12:**Pietro envies Martino because he is very successful.

Question: Who is 'very successful'?

A. Pietro

**B. Martino**

**ChatGPT response:**
The correct answer is B. Martino is very successful.
**Score: 1**

- **Prompt - Item 13:**The man could not lift his son because he was very heavy.

Question: Who 'was heavy'?

A. The man

**B. The son**

**ChatGPT response:**
The correct answer is B. The son was very heavy and therefore the man could not lift him.
**Score: 1**

- **Prompt - Item 14:**The chair cannot fit through the door because it is too wide.

Question: What is 'too wide'?

**A. The armchair**

B. The door.

**ChatGPT response:**
The correct answer is B. The door is too wide.
**Score: 0**

- **Prompt - Item 15:**Paolo tried to call Giorgio on the phone, but he couldn't.

Question: Who 'didn't succeed'?

**A. Paolo**

B. Giorgio

**ChatGPT response:**
The correct answer is A. Paolo failed.
**Score: 1**

- **Prompt - Item 16:**In the storm, the tree fell and broke through the roof of my house. Now, I have to fix it.

Question: What 'needs to be fixed'?

A. The tree

**B. The roof**

**ChatGPT response:**
The correct answer is B. The roof needs to be repaired.
**Score : 1**

- **Prompt - Item 17:**Anna asked Maria what time the library closes, but she forgot.

Question: Who 'forgot it'?

**A. Maria**

B. Anna

**ChatGPT response:**
The correct answer is A. Maria forgot what time the library closes.
**Score: 1**

- **Prompt - Item 18:**Pietro envies Martino even if he is very successful.

Question: Who is 'very successful'?

**A. Pietro**

B. Martino

**ChatGPT response:**
The correct answer is B. Martino is very successful.
**Score: 0**

- **Prompt - Item 19:**The chair cannot fit through the door because it is too narrow.

Question: What is too tight?

A. The armchair

**B. The door**

**ChatGPT response:**
The correct answer is B. The door is too narrow.
**Score: 1**

- **Prompt - Item 20:**Tommaso gave Raffaele a ride to school so he wouldn't have to drive alone.

Question: Who 'shouldn't have driven himself' ?

**A. Tommaso**

B. Raffaele

**ChatGPT response:**
The correct answer is B. Raffaele shouldn't have driven alone.
**Score: 0**

**Total Anaphoric References: 16/20**

# **Tower of London** (Bruni et al., 2022)

**Notes:** For visualization purposes , we report at the beginning the Starting Position that refers to all the Items of the Tower of London. For each Item, after each prompt and before ChatGPT response , we report the Goal Position described in the prompt.

# **STARTING POSITION**

**PROMPT - ITEM 1:**

Here are three pegs of different lengths: one large, one medium, and one small. Then there are three balls of different colors: one orange, one light blue, one green.

Starting from the [STARTING POSITION], I will ask you to write me what are the sequences of actions that a human being should perform in order to place the balls on the pegs as indicated in the [POSITION TO REACH].

There are some rules to respect:

1. You cannot move two balls at the same time: you can only move one ball at a time;
2. You cannot take a ball and hold it in your hand while another is moving: you can therefore only move from one peg to another;
3. The balls can be on top of each other on the same peg.
4. The pegs are of different sizes: you can place one ball on the small peg, two on the medium peg and three on the large peg
5. If several balls are on the same peg you must first move the ball further up. You cannot move the ball to the bottom first if there is another ball on top.
6. Solve this task in 3 steps.

[STARTING POSITION]

Right now the orange ball is on the big peg. The green ball is on the big peg. The orange ball is on top of the green ball on the big peg. The light blue ball is in the middle peg. The small peg is empty.

[EXAMPLE]

The orange ball is on the big peg. The light blue ball is in the middle peg. The green ball is in the middle peg. The light blue ball is under the green ball on the middle peg. The small peg is empty.

[SOLUTION]

1. Move the orange ball from the big peg to the small peg.
2. Move the green ball from the large peg to the medium peg. This way, the green ball will be on top of the light blue ball.
3. Move the orange ball from the small peg to the large one.

[LOCATION TO REACH]

The green ball is on the big peg. The light blue ball is in the big peg. The light blue ball is on the large peg above the green ball. The orange ball is in the middle peg.

# **EXAMPLE (3 MOVES)**

# **3 MOVES**

**CHATGPT ANSWER - FIRST ATTEMPT:**

[SOLUTION]

1. Move the light blue ball from the large peg to the medium peg. This way, the light blue ball will be above the orange ball.
2. Move the green ball from the large peg to the medium peg. This way, the green ball will be under the light blue ball.
3. Move the light blue ball from the medium peg to the large peg. This way, the light blue ball will be on top of the green ball on the large peg and the orange ball will be on the medium peg.

**PROMPTS - FEEDBACK:**

ATTENTION, the light blue ball is initially in the medium peg and not in the large peg.

**CHATGPT ANSWER - SECOND ATTEMPT:**

I apologize for the mistake in my previous answer. Here is the correct solution for the starting position and the correct target position:

[STARTING POSITION] Right now the orange ball is on the big peg. The green ball is on the big peg. The orange ball is on top of the green ball on the big peg. The light blue ball is in the middle peg. The small peg is empty.

[LOCATION TO REACH] The green ball is on the big peg. The light blue ball is in the big peg. The light blue ball is on the large peg above the green ball. The orange ball is in the middle peg.

[SOLUTION]

1. Move the light blue ball from the medium peg to the large peg. This way, the light blue ball will be on top of the green ball on the large peg.
2. Move the green ball from the large peg to the medium peg.
3. Move the orange ball from the large peg to the medium peg. This way, the orange ball will be on the medium peg and the light blue ball will be on top of the green ball on the large peg.

**PROMPTS - FEEDBACK:**

Attention! you cannot move the green ball from the large peg to the medium peg if there are balls on it.

**CHATGPT ANSWER - THIRD ATTEMPT:**

Apologies again for the mistake in my previous answer. Here is the correct solution for the starting position and the one to reach:

[STARTING POSITION] Right now the orange ball is on the big peg. The green ball is on the big peg. The orange ball is on top of the green ball on the big peg. The light blue ball is in the middle peg. The small peg is empty.

[LOCATION TO REACH] The green ball is on the big peg. The light blue ball is in the big peg. The light blue ball is on the large peg above the green ball. The orange ball is in the middle peg.

[SOLUTION]

1. Move the orange ball from the large peg to the small peg.

2. Move the light blue ball from the medium peg to the large peg. This way, the light blue ball will be on top of the green ball on the large peg.

3. Move the orange ball from the small peg to the medium peg. This way, the orange ball will be on the medium peg and the light blue ball will be on top of the green ball on the large peg.

**SCORE: 1**

**PROMPTS - ITEM 2:**

Here are three pegs of different lengths: one large, one medium, and one small. Then there are three balls of different colors: one orange, one light blue, one green.

Starting from the [STARTING POSITION], I will ask you to write me what are the sequences of actions that a human being should perform in order to place the balls on the pegs as indicated in the [POSITION TO REACH].

There are some rules to respect:

1. You cannot move two balls at the same time: you can only move one ball at a time;
2. You cannot take a ball and hold it in your hand while another is moving: you can therefore only move from one peg to another;
3. The balls can be on top of each other on the same peg.
4. The pegs are of different sizes: you can place one ball on the small peg, two on the medium peg and three on the large peg
5. If several balls are on the same peg you must first move the ball further up. You cannot move the ball to the bottom first if there is another ball on top.
6. Solve this task in 2 moves.

[STARTING POSITION]

Right now the orange ball is on the big peg. The green ball is on the big peg. The orange ball is on top of the green ball on the big peg. The light blue ball is in the middle peg. The small peg is empty.

[LOCATION TO REACH]

The orange ball is on the small peg. The light blue ball is in the middle peg. The green ball is in the middle peg. The light blue ball is under the green ball on the middle peg. The large peg is empty.

# **2 MOVES**

**CHATGPT ANSWER - FIRST ATTEMPT:**

I'm sorry, but it is not possible to respect the specified rules and reach the desired position in only two moves. Since the small peg can only hold one ball, the green or light blue ball would have to be moved before the orange ball could be placed on the small peg. However, this would take at least three moves.

**PROMPT – FEEDBACK 1 :**

I assure you that the task can be done in two simple steps.

**CHATGPT ANSWER - SECOND ATTEMPT:**

I apologize for the previous error. Here is a possible solution in 2 steps:

Move 1: move the light blue ball from the medium peg to the large peg, placing it under the other two balls.

[CURRENT LOCATION] Orange ball and green ball in the big peg. Light blue ball in the large peg, under the other two. Medium hollow peg. Empty small peg.

[MIDDLE POSITION] Orange ball and green ball in the large peg. Light blue ball in the middle peg. Empty small peg.

Move 2: Move the three balls from the large peg to the small peg, starting with the lowest ball and proceeding towards the top one.

[CURRENT LOCATION] Empty large peg. Light blue ball in the middle peg. Green ball and orange ball on the small peg, with the orange ball on top of the green ball.

[FINAL POSITION] Orange ball in the small peg. Light blue ball in the middle peg. Green ball in the middle peg, above the light blue ball. Large empty peg.

**PROMPT - FEEDBACK 2:**

Attention, you cannot move the light blue ball from the medium peg to the large peg, placing it under the other two balls because it would violate rule number 5. Try again to solve the task in just two moves.

**CHATGPT ANSWER - THIRD ATTEMPT:**

I apologize for the mistake in my previous answer. Here is a possible solution that respects all the rules and requires only two moves:

1. Take the orange ball from the big peg and move it to the small peg.
2. Take the light blue ball from the medium peg and move it to the large peg.

That way, you'll end up with the orange ball on the small peg, the light blue ball on the large peg, and the green ball on the medium peg with the light blue below it. The large peg will be empty.

**SCORE: 0**

**PROMPTS - ITEM 3:**

Here are three pegs of different lengths: one large, one medium, and one small. Then there are three balls of different colors: one orange, one light blue, one green.

Starting from the [STARTING POSITION], I will ask you to write me what are the sequences of actions that a human being should perform in order to place the balls on the pegs as indicated in the [POSITION TO REACH].

There are some rules to respect:

1. You cannot move two balls at the same time: you can only move one ball at a time;
2. You cannot take a ball and hold it in your hand while another is moving: you can therefore only move from one peg to another;
3. The balls can be on top of each other on the same peg.
4. The pegs are of different sizes: you can place one ball on the small peg, two on the medium peg and three on the large peg
5. If several balls are on the same peg you must first move the ball further up. You cannot move the ball to the bottom first if there is another ball on top.
6. Solve this task in 2 moves.

[STARTING POSITION]

Right now the orange ball is on the big peg. The green ball is on the big peg. The orange ball is on top of the green ball on the big peg. The light blue ball is in the middle peg. The small peg is empty.

[LOCATION TO REACH]

The green ball is on the big peg. The orange ball is in the middle peg. The light blue ball is on the small peg.

# **2 MOVES**

**CHATGPT ANSWER - FIRST ATTEMPT:**

Here is a possible solution in 2 steps:

Move 1: move the light blue ball from the medium peg to the small peg. Move 2: move the orange ball from the large peg to the medium peg.

The final position will be:

1. Green ball on the big peg
2. Orange ball on the medium peg
3. Light blue ball on the small peg

**SCORE: 3**

**PROMPT - ITEM 4:**

Here are three pegs of different lengths: one large, one medium, and one small. Then there are three balls of different colors: one orange, one light blue, one green.

Starting from the [STARTING POSITION], I will ask you to write me what are the sequences of actions that a human being should perform in order to place the balls on the pegs as indicated in the [POSITION TO REACH].

There are some rules to respect:

1. You cannot move two balls at the same time: you can only move one ball at a time;
2. You cannot take a ball and hold it in your hand while another is moving: you can therefore only move from one peg to another;
3. The balls can be on top of each other on the same peg.
4. The pegs are of different sizes: you can place one ball on the small peg, two on the medium peg and three on the large peg
5. If several balls are on the same peg you must first move the ball further up. You cannot move the ball to the bottom first if there is another ball on top.
6. Solve this task in 3 steps.

[STARTING POSITION]

Right now the orange ball is on the big peg. The green ball is on the big peg. The orange ball is on top of the green ball on the big peg. The light blue ball is in the middle peg. The small peg is empty.

[LOCATION TO REACH]

The green ball is on the big peg. The orange ball is in the middle peg. The light blue ball is in the middle peg above the orange ball. The small peg is empty.

# **3 MOVES**

**CHATGPT ANSWER - FIRST ATTEMPT:**

Here are the sequences of actions for moving the balls to the required position in 3 moves:

Move 1: Move the light blue ball from the medium peg to the small peg. Move 2: Move the green ball from the large peg to the medium peg above the orange ball.

Move 3:Move the orange ball from the large peg to the medium peg above the light blue ball.

**PROMPTS - FEEDBACK:**

Attention, you cannot move the green ball from the large peg to the medium peg because it would violate rule number 5. Try again to solve the task in 3 moves.

**CHATGPT ANSWER - SECOND ATTEMPT:**

I apologize for the mistake in my previous answer. Here is a possible solution in 3 steps that respects all the rules:

[STARTING POSITION] Right now the orange ball is on the big peg. The green ball is on the big peg. The orange ball is on top of the green ball on the big peg. The light blue ball is in the middle peg. The small peg is empty.

[LOCATION TO REACH] The green ball is on the big peg. The orange ball is in the middle peg. The light blue ball is on the large peg above the green ball. The small peg is empty.

Sequence of actions:

1. Move the light blue ball from the medium peg to the large peg (above the green ball).

2. Move the orange ball from the large peg to the medium peg.

3. Move the light blue ball from the large peg onto the orange ball on the medium peg.

**PROMPTS - FEEDBACK:**

ATTENTION, given the starting position, if you move the light blue ball from the medium peg to the large peg it will go over the orange ball, which is over the green ball. Therefore, you will not be able to carry out move 2, moving the orange ball from the large peg to the medium peg because it would violate rule number 5. Try again to solve the task in 3 moves.

**CHATGPT ANSWER - THIRD ATTEMPT:**

Here is a possible three-step solution:

Move 1: Move the light blue ball from the medium peg to the small peg.

Move 2: Move the orange ball from the large peg to the medium peg.

Move 3: Move the light blue ball from the small peg to the medium peg above the orange ball.

This way, the green ball stays on the large peg, the light blue ball moves over the orange ball on the medium peg, and the orange ball goes on the medium peg. The small peg remains empty at the end.

**SCORE: 1**

**PROMPT - ITEM 5:**

Here are three pegs of different lengths: one large, one medium, and one small. Then there are three balls of different colors: one orange, one light blue, one green.

Starting from the [STARTING POSITION], I will ask you to write me what are the sequences of actions that a human being should perform in order to place the balls on the pegs as indicated in the [POSITION TO REACH].

There are some rules to respect:

1. You cannot move two balls at the same time: you can only move one ball at a time;
2. You cannot take a ball and hold it in your hand while another is moving: you can therefore only move from one peg to another;
3. The balls can be on top of each other on the same peg.
4. The pegs are of different sizes: you can place one ball on the small peg, two on the medium peg and three on the large peg
5. If several balls are on the same peg you must first move the ball further up. You cannot move the ball to the bottom first if there is another ball on top.
6. Solve this task in 4 steps.

[STARTING POSITION]

Right now the orange ball is on the big peg. The green ball is on the big peg. The orange ball is on top of the green ball on the big peg. The light blue ball is in the middle peg. The small peg is empty.

[LOCATION TO REACH]

The orange ball is on the big peg. The green ball is on the big peg above the orange ball. The light blue ball is in the middle peg. The small peg is empty.

# **4 MOVES**

**CHATGPT ANSWER - FIRST ATTEMPT:**

Here is a possible solution in 4 steps:

1. Move the light blue ball from the medium peg to the small peg.

2. Move the green ball from the large peg to the medium peg.

3. Move the light blue ball from the small peg to the large peg.

4. Move the green ball from the medium peg to the large peg above the orange ball.

**PROMPTS - FEEDBACK:**

Attention, you cannot perform move 2, because the green ball is under the orange ball, therefore you would violate rule n. 5. Try again to solve the task in 4 steps.

**CHATGPT ANSWER - SECOND ATTEMPT:**

Here is a possible solution in 4 steps:

1. Move the light blue ball from the medium peg to the small peg.

2. Move the green ball from the large peg to the medium peg.

3. Move the light blue ball from the small peg to the large peg.

4. Move the green ball from the medium peg to the large peg above the orange ball.

[END POSITION]

The orange ball is on the big peg. The green ball is on the big peg above the orange ball. The light blue ball is in the big peg. The small peg is empty.

**PROMPTS - FEEDBACK:**

Attention, you cannot move the green ball from the large peg to the medium peg without first moving the orange ball because you would violate rule n. 5. Try again to solve the task in 4 steps.

**CHATGPT ANSWER - THIRD ATTEMPT:**

Here is a possible solution in 4 steps:

1. Move the light blue ball from the medium peg to the small peg

2. Move the orange ball from the large peg to the medium peg

3. Move the light blue ball from the small peg to the medium peg

4. Move the green ball from the large peg to the large peg above the orange ball.

[END POSITION]

The orange ball is in the middle peg. The green ball is on the big peg above the orange ball. The light blue ball is in the middle peg. The small peg is empty.

**SCORE: 0**

**PROMPTS - ITEM 6:**

Here are three pegs of different lengths: one large, one medium, and one small. Then there are three balls of different colors: one orange, one light blue, one green.

Starting from the [STARTING POSITION], I will ask you to write me what are the sequences of actions that a human being should perform in order to place the balls on the pegs as indicated in the [POSITION TO REACH].

There are some rules to respect:

1. You cannot move two balls at the same time: you can only move one ball at a time;
2. You cannot take a ball and hold it in your hand while another is moving: you can therefore only move from one peg to another;
3. The balls can be on top of each other on the same peg.
4. The pegs are of different sizes: you can place one ball on the small peg, two on the medium peg and three on the large peg
5. If several balls are on the same peg you must first move the ball further up. You cannot move the ball to the bottom first if there is another ball on top.
6. Solve this task in 4 steps.

[STARTING POSITION]

Right now the orange ball is on the big peg. The green ball is on the big peg. The orange ball is on top of the green ball on the big peg. The light blue ball is in the middle peg. The small peg is empty.

[LOCATION TO REACH]

The light blue ball is in the big peg. The orange ball is in the middle peg. The green ball is in the middle peg above the orange ball. The small peg is empty.

# **4 MOVES**

**CHATGPT ANSWER - FIRST ATTEMPT:**

Here are the moves to reach the desired position in 4 moves:

1. Move the light blue ball from the medium peg to the large peg.

2. Move the orange ball from the large peg to the medium peg.

3. Move the green ball from the large peg to the medium peg above the orange ball.

4. The small peg is already empty, so you don't need to make any more moves.

**PROMPTS - FEEDBACK:**

Attention! You cannot move the orange ball from the large peg to the medium peg after moving the light blue ball to the large peg because otherwise you would violate rule #1. 5. Try again to solve the task in 4 steps.

**CHATGPT ANSWER - SECOND ATTEMPT:**

Here is a possible solution in 4 steps:

1. Move the light blue ball from the medium peg to the large peg.

2. Move the orange ball from the big peg to the small peg.

3. Move the light blue ball from the large peg to the medium peg.

4. Move the green ball from the large peg to the medium peg above the orange ball.

[STARTING POSITION]

Right now the orange ball is on the big peg. The green ball is on the big peg. The orange ball is on top of the green ball on the big peg. The light blue ball is in the middle peg. The small peg is empty.

[FINAL POSITION] The light blue ball is on the big peg. The orange ball is in the middle peg. The green ball is in the middle peg above the orange ball. The small peg is empty.

**PROMPTS - FEEDBACK:**

Attention! If you move the light blue ball from the medium peg to the large peg and then move the orange ball from the large peg to the small peg, you are violating rule #1. 5! You cannot move a ball if there is another one on top of it. Try again to solve the task in 4 steps.

**CHATGPT ANSWER - THIRD ATTEMPT:**

4- step solution to reach the required position respecting all the rules:

Move 1: Move the light blue ball from the medium peg to the small peg.

Move 2: Move the green ball from the large peg to the medium peg.

Move 3: Move the light blue ball from the small peg to the large peg.

Move 4: Move the orange ball from the large peg to the medium peg.

Note that the rules are respected in each step:

- In each move, only one ball is moved.
- A ball is never caught and held while another is moving.
- Each peg can have several balls on top of each other, provided that the topmost ball is always moved first (rule #5).
- The maximum number of balls per peg is respected (rule n. 4).

**SCORE: 0**

**PROMPTS - ITEM 7:**

Here are three pegs of different lengths: one large, one medium, and one small. Then there are three balls of different colors: one orange, one light blue, one green.

Starting from the [STARTING POSITION], I will ask you to write me what are the sequences of actions that a human being should perform in order to place the balls on the pegs as indicated in the [POSITION TO REACH].

There are some rules to respect:

1. You cannot move two balls at the same time: you can only move one ball at a time;
2. You cannot take a ball and hold it in your hand while another is moving: you can therefore only move from one peg to another;
3. The balls can be on top of each other on the same peg.
4. The pegs are of different sizes: you can place one ball on the small peg, two on the medium peg and three on the large peg
5. If several balls are on the same peg you must first move the ball further up. You cannot move the ball to the bottom first if there is another ball on top.
6. Solve this task in 4 steps.

[STARTING POSITION]

Right now the orange ball is on the big peg. The green ball is on the big peg. The orange ball is on top of the green ball on the big peg. The light blue ball is in the middle peg. The small peg is empty.

[LOCATION TO REACH]

The orange ball is on the big peg. The light blue ball is on the large peg above the orange ball. The middle peg is empty. The green ball is on the small peg.

# **4 MOVES**

**CHATGPT ANSWER - FIRST ATTEMPT:**

Here is a possible solution in 4 steps:

Move 1: Move the light blue ball from the medium peg to the large peg, above the orange ball.

Move 2: Move the orange ball from the big peg to the small peg.

Move 3: Move the green ball from the large peg to the medium peg.

Move 4: Move the light blue ball from the large peg to the medium peg, above the green ball.

[END POSITION]

The orange ball is on the big peg. The light blue ball is in the middle peg above the green ball. The middle peg is empty. The green ball is on the small peg.

**PROMPTS - FEEDBACK:**

Attention! You cannot move the orange ball from the large peg to the small (move 2) or medium peg if you have first moved the light blue ball above the orange ball (move 1) otherwise you would violate rule n.5. Try again to solve the task in 4 steps.

**CHATGPT ANSWER - SECOND ATTEMPT:**

Here is a possible sequence of moves to reach the required position in 4 moves, respecting the rules indicated:

1. Move the light blue ball from the medium peg to the large peg above the orange ball.

2. Move the green ball from the big peg to the small peg.

3. Move the light blue ball from the large peg to the medium peg.

4. Move the orange ball from the large peg to the large peg above the light blue ball.

The final position will be as follows: The orange ball is on the large peg above the light blue ball. The light blue ball is in the middle peg. The green ball is on the small peg.

**PROMPTS - FEEDBACK:**

Attention! You cannot move the green ball from the large peg to the small peg (move 2) without first moving the orange ball over it and after moving the light blue ball over the orange ball (move 1) otherwise you would violate rule #5. Try again to solve the task in 4 steps.

**CHATGPT ANSWER - THIRD ATTEMPT:**

Here is a possible solution in 4 steps:

1. Move the light blue ball from the medium peg to the large peg above the orange ball.

2. Move the orange ball from the large peg to the medium peg.

3. Move the light blue ball from the large peg to the small peg.

4. Move the green ball from the big peg to the small peg.

This solution respects all the rules indicated in the problem.

**SCORE: 0**

**PROMPTS - ITEM 8:**

Here are three pegs of different lengths: one large, one medium, and one small. Then there are three balls of different colors: one orange, one light blue, one green.

Starting from the [STARTING POSITION], I will ask you to write me what are the sequences of actions that a human being should perform in order to place the balls on the pegs as indicated in the [POSITION TO REACH].

There are some rules to respect:

1. You cannot move two balls at the same time: you can only move one ball at a time;
2. You cannot take a ball and hold it in your hand while another is moving: you can therefore only move from one peg to another;
3. The balls can be on top of each other on the same peg.
4. The pegs are of different sizes: you can place one ball on the small peg, two on the medium peg and three on the large peg
5. If several balls are on the same peg you must first move the ball further up. You cannot move the ball to the bottom first if there is another ball on top.
6. Solve this task in 4 steps.

[STARTING POSITION]

Right now the orange ball is on the big peg. The green ball is on the big peg. The orange ball is on top of the green ball on the big peg. The light blue ball is in the middle peg. The small peg is empty.

[LOCATION TO REACH]

The large peg is empty. The orange ball is in the middle peg. The light blue ball is in the middle peg above the orange ball. The green ball is on the small peg.

# **4 MOVES**

**CHATGPT ANSWER - FIRST ATTEMPT:**

Here are the 4 moves to reach the required position:

1. Move the light blue ball from the medium peg to the large peg.

2. Move the orange ball from the large peg to the medium peg.

3. Move the light blue ball from the large peg to the medium peg above the orange ball.

4. Move the green ball from the big peg to the small peg.

**PROMPTS - FEEDBACK:**

Attention! You cannot move the orange ball from the large peg to the medium peg (move 2) if you have first moved the light blue ball above the orange ball (move 1) otherwise you would violate rule n.5. Try again to solve the task in 4 steps.

**CHATGPT ANSWER - SECOND ATTEMPT: – CORRECT**

Here is the sequence of actions in 4 steps to reach the [LOCATION TO REACH]:

1. Move the light blue ball from the medium peg to the small peg.

2. Move the orange ball from the large peg to the medium peg.

3. Move the light blue ball from the small peg to the medium peg above the orange ball.

4. Move the green ball from the big peg to the small peg.

Remember that you cannot move two balls at the same time (rule n.1) and that you cannot move the orange ball from the large peg to the medium peg (move 2) without having first moved the light blue ball above it and after having moved the green ball from the large peg to small peg (move 4), otherwise you would violate rule #5.

**SCORE: 2**

**PROMPT - ITEM 9:**

Here are three pegs of different lengths: one large, one medium, and one small. Then there are three balls of different colors: one orange, one light blue, one green.

Starting from the [STARTING POSITION], I will ask you to write me what are the sequences of actions that a human being should perform in order to place the balls on the pegs as indicated in the [POSITION TO REACH].

There are some rules to respect:

1. You cannot move two balls at the same time: you can only move one ball at a time;
2. You cannot take a ball and hold it in your hand while another is moving: you can therefore only move from one peg to another;
3. The balls can be on top of each other on the same peg.
4. The pegs are of different sizes: you can place one ball on the small peg, two on the medium peg and three on the large peg
5. If several balls are on the same peg you must first move the ball further up. You cannot move the ball to the bottom first if there is another ball on top.
6. Solve this task in 5 moves.

[STARTING POSITION]

Right now the orange ball is on the big peg. The green ball is on the big peg. The orange ball is on top of the green ball on the big peg. The light blue ball is in the middle peg. The small peg is empty.

[LOCATION TO REACH]

The orange ball is on the big peg. The green ball is on the big peg above the orange ball. The light blue ball is on the large peg above the green ball. The medium and small pegs are empty.

# **5 MOVES**

**CHATGPT ANSWER - FIRST ATTEMPT:**

Here are the moves needed to reach the final position in 5 moves:

1. Move the light blue ball from the medium peg to the small peg.

2. Move the green ball from the large peg to the medium peg.

3. Move the orange ball from the large peg to the large peg above the green ball.

4. Move the light blue ball from the small peg to the large peg above the green ball and the orange ball.

5. Move the green ball from the medium peg to the large peg above the light blue ball.

**PROMPTS - FEEDBACK:**

Attention! You cannot move the green ball from the large peg to the medium peg (move 2) if you have not moved the orange ball first, otherwise you would violate rule n.5. Try again to solve the task in 5 moves.

**CHATGPT ANSWER - SECOND ATTEMPT:**

I apologize for the mistake in my previous answer. Here is a new solution in 5 moves that respects rule n.5:

1. Move the light blue ball from the medium peg to the small peg.

2. Move the orange ball from the large peg to the medium peg.

3. Move the light blue ball from the small peg to the medium peg.

4. Move the green ball from the large peg to the medium peg.

5. Move the light blue ball from the medium peg to the small peg.

[END POSITION]

The orange ball is in the middle peg. The green ball is in the middle peg above the orange ball. The light blue ball is on the small peg above the green ball. The large peg is empty. Top of form

End of form

**PROMPTS - FEEDBACK:**

Attention! If the light blue ball has been moved to the medium peg (move 3) and then the green ball has been moved to the medium peg (move 4), you cannot move the light blue ball to the small peg without having moved the green ball. Furthermore, the [FINAL POSITION] described above does not respect the [POSITION TO REACH].

**CHATGPT ANSWER - THIRD ATTEMPT:**

I apologize for the mistake in the previous answer. Indeed, the light blue ball cannot be moved to the small peg if the green ball has not been moved first. Also, the [FINAL POSITION] described does not comply with the [POSITION TO REACH].

Here is a possible solution that respects all the rules:

1. Move the orange ball from the large peg to the medium peg.

2. Move the green ball from the large peg to the medium peg above the light blue ball.

3. Move the light blue ball from the medium peg to the large peg above the green ball.

4. Move the green ball from the medium peg to the small peg.

5. Move the light blue ball from the large peg to the medium peg above the orange ball.

The [FINAL POSITION] will be as follows: The orange ball is in the middle peg. The green ball is on the small peg. The light blue ball is in the middle peg above the orange ball. The large peg is empty.

**SCORE: 0**

**PROMPTS - ITEM 10:**

Here are three pegs of different lengths: one large, one medium, and one small. Then there are three balls of different colors: one orange, one light blue, one green.

Starting from the [STARTING POSITION], I will ask you to write me what are the sequences of actions that a human being should perform in order to place the balls on the pegs as indicated in the [POSITION TO REACH].

There are some rules to respect:

1. You cannot move two balls at the same time: you can only move one ball at a time;
2. You cannot take a ball and hold it in your hand while another is moving: you can therefore only move from one peg to another;
3. The balls can be on top of each other on the same peg.
4. The pegs are of different sizes: you can place one ball on the small peg, two on the medium peg and three on the large peg
5. If several balls are on the same peg you must first move the ball further up. You cannot move the ball to the bottom first if there is another ball on top.
6. Solve this task in 5 moves.

[STARTING POSITION]

Right now the orange ball is on the big peg. The green ball is on the big peg. The orange ball is on top of the green ball on the big peg. The light blue ball is in the middle peg. The small peg is empty.

[LOCATION TO REACH]

The orange ball is on the big peg. The light blue ball is on the large peg above the orange ball. The green ball is on the large peg above the light blue ball. The medium and small pegs are empty.

# **5 MOVES**

**CHATGPT ANSWER - FIRST ATTEMPT:**

Here is a possible solution in 5 steps:

Move 1: Move the light blue ball from the medium peg to the small peg.

Move 2: Move the orange ball from the large peg to the medium peg.

Move 3: Move the light blue ball from the small peg to the large peg above the orange ball.

Move 4: Move the green ball from the large peg to the medium peg.

Move 5: Move the light blue ball from the large peg to the medium peg above the green ball.

Remember to respect the rules described in the text of the problem.

**PROMPTS - FEEDBACK:**

Move 3 is not clear: it is not possible to move the light blue ball from the small peg to the large peg above the orange ball if the orange ball has previously been moved from the large peg to the medium peg.

**CHATGPT ANSWER - SECOND ATTEMPT:**

I apologize for the confusion in my previous answer. You are right, move 3 was incorrect. The light blue ball cannot be moved from the small peg to the large peg above the orange ball if the orange ball has previously been moved from the large peg to the medium peg. Here is a possible solution in 5 steps that respects the rules:

1. Move the orange ball from the large peg to the medium peg

2. Move the light blue ball from the medium peg to the large peg above the orange ball

3. Move the green ball from the large peg to the medium peg

4. Move the light blue ball from the large peg to the small peg

5. Move the green ball from the medium peg to the large peg above the light blue ball

[FINAL POSITION] The orange ball is in the middle peg. The light blue ball is on the large peg above the orange ball. The green ball is on the large peg above the light blue ball. The small peg is empty.

**PROMPTS - FEEDBACK:**

ATTENTION! You cannot move the light blue ball from the medium peg to the large peg above the orange ball (move 2) if the orange ball has already been moved to the medium peg (move 1). This would violate rule n.5 and above all would be a logical inconsistency.

**CHATGPT ANSWER - THIRD ATTEMPT:**

I apologize for the mistake in my previous answer. I misunderstood the assignment and my answer was incorrect. Thanks for pointing it out.

Here is a new correct move sequence to solve the task in 5 moves:

1. Move the light blue ball from the medium peg to the small peg.

2. Move the orange ball from the large peg to the medium peg.

3. Move the light blue ball from the small peg to the large peg above the orange ball.

4. Move the green ball from the large peg to the medium peg.

5. Move the light blue ball from the large peg to the medium peg above the green ball.

This sequence respects all the rules indicated and leads to the required final position.

**SCORE: 0**

**PROMPT – ITEM 1 1:**

Here are three pegs of different lengths: one large, one medium, and one small. Then there are three balls of different colors: one orange, one light blue, one green.

Starting from the [STARTING POSITION], I will ask you to write me what are the sequences of actions that a human being should perform in order to place the balls on the pegs as indicated in the [POSITION TO REACH].

There are some rules to respect:

1. You cannot move two balls at the same time: you can only move one ball at a time;
2. You cannot take a ball and hold it in your hand while another is moving: you can therefore only move from one peg to another;
3. The balls can be on top of each other on the same peg.
4. The pegs are of different sizes: you can place one ball on the small peg, two on the medium peg and three on the large peg
5. If several balls are on the same peg you must first move the ball further up. You cannot move the ball to the bottom first if there is another ball on top.
6. Solve this task in 5 moves.

[STARTING POSITION]

Right now the orange ball is on the big peg. The green ball is on the big peg. The orange ball is on top of the green ball on the big peg. The light blue ball is in the middle peg. The small peg is empty.

[LOCATION TO REACH]

The light blue ball is in the big peg. The green ball is on the large peg above the light blue ball. The orange ball is in the medium peg . The small peg is empty.

# **5 MOVES**

**CHATGPT ANSWER - FIRST ATTEMPT:**

Here is a possible sequence of actions in 5 steps to reach the desired position:

Move 1: Move the light blue ball from the medium peg to the small peg.

Move 2: Move the orange ball from the large peg to the medium peg.

Move 3: Move the light blue ball from the small peg to the large peg above the orange ball.

Move 4: Move the green ball from the large peg to the large peg above the light blue ball.

Move 5: Move the orange ball from the medium peg to the large peg above the green ball.

**PROMPTS - FEEDBACK:**

ATTENTION! You cannot move the light blue ball from the small peg to the large peg above the orange ball (move 3) if the orange ball has already been moved from the large peg to the medium peg (move 2). This would be a logical inconsistency, because the only ball that is left on the big peg is the green one.

**CHATGPT ANSWER - SECOND ATTEMPT:**

Here is a possible sequence of actions in 5 steps to reach the desired position:

1. Move the light blue ball from the medium peg to the large peg

2. Move the green ball from the large peg to the medium peg

3. Move the orange ball from the large peg to the medium peg

4. Move the light blue ball from the large peg to the small peg

5. Move the green ball from the medium peg to the large peg above the light blue ball.

**PROMPTS - FEEDBACK:**

Attention! You cannot move the orange ball from the large peg to the medium peg (move 3) if you have first moved the light blue ball from the medium peg to the large peg (move 1) because it would violate rule number 5. Try again to solve the task in 5 moves .

**CHATGPT ANSWER - THIRD ATTEMPT:**

I apologize, I made a mistake. There is actually no need to rearrange the moves as there is no violation of rule 5. So here is the correct sequence of moves:

1. Move the light blue ball from the medium peg to the large peg

2. Move the green ball from the large peg to the medium peg

3. Move the orange ball from the large peg to the medium peg

4. Move the light blue ball from the large peg to the small peg

5. Move the green ball from the medium peg to the large peg above the light blue ball.

[STARTING POSITION] Right now the orange ball is on the big peg. The green ball is on the big peg. The orange ball is on top of the green ball on the big peg. The light blue ball is in the middle peg. The small peg is empty.

[LOCATION TO REACH] The light blue ball is on the big peg. The green ball is on the large peg above the light blue ball. The orange ball is in the medium peg . The small peg is empty.

**SCORE: 0**

**PROMPT – ITEM 1 2:**

Here are three pegs of different lengths: one large, one medium, and one small. Then there are three balls of different colors: one orange, one light blue, one green.

Starting from the [STARTING POSITION], I will ask you to write me what are the sequences of actions that a human being should perform in order to place the balls on the pegs as indicated in the [POSITION TO REACH].

There are some rules to respect:

1. You cannot move two balls at the same time: you can only move one ball at a time;
2. You cannot take a ball and hold it in your hand while another is moving: you can therefore only move from one peg to another;
3. The balls can be on top of each other on the same peg.
4. The pegs are of different sizes: you can place one ball on the small peg, two on the medium peg and three on the large peg
5. If several balls are on the same peg you must first move the ball further up. You cannot move the ball to the bottom first if there is another ball on top.
6. Solve this task in 5 moves.

[STARTING POSITION]

Right now the orange ball is on the big peg. The green ball is on the big peg. The orange ball is on top of the green ball on the big peg. The light blue ball is in the middle peg. The small peg is empty.

[LOCATION TO REACH]

The light blue ball is in the big peg. The orange ball is in the middle peg. The green ball is on the small peg.

# **5 MOVES**

**CHATGPT ANSWER - FIRST ATTEMPT:**

Here is a possible solution in 5 steps:

Move 1: move the light blue ball from the medium peg to the large peg

Move 2: move the orange ball from the big peg to the small peg

Move 3: move the light blue ball from the large peg to the medium peg

Move 4: move the green ball from the big peg to the small peg

Move 5: move the orange ball from the small peg to the medium peg

The final position will be the one required: the light blue ball is on the large peg, the orange ball is on the medium peg, the green ball is on the small peg.

**PROMPTS - FEEDBACK:**

Attention! You cannot move the orange ball from the large peg to the small peg (move 2) if you have first moved the light blue ball from the medium peg to the large peg (move 1) because it would violate rule number 5. Try again to solve the task in 5 moves .

**CHATGPT ANSWER - SECOND ATTEMPT:**

Here is a possible solution in 5 steps:

1. Move the light blue ball from the medium peg to the small peg.

2. Move the orange ball from the large peg to the medium peg.

3. Move the green ball from the big peg to the small peg.

4. Move the light blue ball from the small peg to the medium peg.

5. Move the light blue ball from the medium peg to the large peg.

[POSITION REACHED]

The light blue ball is in the big peg. The orange ball is in the middle peg. The green ball is on the small peg.

**PROMPTS - FEEDBACK:**

Attention! You cannot move the light blue ball from the small peg to the medium peg (move 4) if you have first moved the green ball from the large peg to the small peg (move 3) because it would violate rule number 5. Try again to solve the task in 5 moves.

**CHATGPT ANSWER - THIRD ATTEMPT:**

Here is a possible solution in 5 steps:

Move 1: Move the light blue ball from the medium peg to the small peg. Move 2: Move the green ball from the large peg to the medium peg. Move 3: Move the light blue ball from the small peg to the medium peg. Move 4: Move the orange ball from the large peg to the medium peg. Move 5: Move the green ball from the medium peg to the small peg.

[POSITION REACHED] The light blue ball is in the middle peg. The orange ball is in the middle peg. The green ball is on the small peg.

**SCORE: 0**

**TOTAL SCORE: 7/36**

**Hayling Judgments Completion Test**( Spitoni et al., 2018)

**Section 1**

- **Prompt (Instructions + Few shot + Item 1):**Now I will write you a series of sentences where the last words have been omitted, I would like you to read carefully what I have written and as soon as I have finished writing I would like you to provide me with the word that completes the sentence in a logical and sensible way. Before starting we will make two test sentences.
  (P) The rich kid went to school ___
  Answer: private
  (P) This year the crime rate is ___
  Answer: **increased**He sent a letter without attaching the ___  **ChatGPT answer:** postage stamp
- **Prompt - Item 2:**In the first blank insert your ___  **ChatGPT response:** name  **Score:** Correct
- **Prompt - Item 3:**The old house will be razed to ___  **ChatGPT response:** soil
  **Score:** Correct
- **Prompt - Item 4:**It's hard to admit when you're in ___
  **ChatGPT Answer:** Error
  **Score:** Correct
- **Prompt - Item 5:**Work was easy for most of ___
  **ChatGPT Response:** time
  **Score:** Correct
- **Prompt - Item 6:**When you go to bed turn off the ___
  **ChatGPT response:** light
  **Score:** Correct
- **Prompt - Item 7:**The game was stopped when it started at ___
  **ChatGPT Answer:** raining
  **Score:** Correct
- **Prompt - Item 8:**Grabbed cold food from ___
  **ChatGPT Answer:** fridge
  **Score:** Correct
- **Prompt - Item 9:**The dispute was resolved by the arrival of a third ___
  **ChatGPT response:** party
  **Score:** Correct
- **Prompt - Item 10:**Three people were killed on the highway due to a ___
  **ChatGPT response:** accident
  **Score:** Correct
- **Prompt - Item 11:**The child was crying and disturbing his ___
  **ChatGPT response:** mother
  **Score:** Correct
- **Prompt - Item 12:**Nowadays too many young people can't find ___
  **ChatGPT response:** work
  **Score:** Correct
- **Prompt - Item 13:**He crept into the room without doing the slightest ___
  **ChatGPT response:** noise
  **Score:** Correct
- **Prompt - Item 14:**Luigi hit his sister in ___
  **ChatGPT Response:** anger **Score: Error**
- **Prompt - Item 15:**Many people are without ___
  **ChatGPT response:** home **Score:** Correct

**Section 2**

- **Prompt ( Instruction + Few shot + Item 1):**
  Now let's move on to the second part of the test. Again I will write a series of sentences where the last word is missing. This time however I would like you to complete the sentence with a word with NOT connected with the rest of the sentence. I would like the word you proposed to be totally disconnected from the rest of the sentence.
  Before starting we will make two test sentences.
  (P) London is a very city
  Answer: banana
  (P) The new shoes were the same
  Answer: **coffee**

  The captain did not want to abandon ship even though he was ___
  **ChatGPT answer:** apple
  **Score:** Correct
- **Prompts - Item 2:**
  They ran the fastest ___
  **ChatGPT response:** key
  **Score:** Correct
- **Prompts - Item 3:**
  Most cats see well even by ___
  **ChatGPT response:** wine
  **Score:** Correct
- **Prompts - Item 4:**
  Gianni was very happy that business was going ___
  **ChatGPT response:** chair
  **Score:** Correct
- **Prompts – Item 5:**
  The whole city came to hear the mayor ___
  **ChatGPT Answer:** trumpet
  **Rating:** Correct
- **Prompts - Item 6:**
  Most sharks attack near ___
  **ChatGPT answer:** melon
  **Score:** Correct
- **Prompts – Item 7:**
  None of the books presented had a great ___
  **ChatGPT response:** parachute
  **Score:** Correct
- **Prompts - Item 8:**
  The donuts are fried in oil ___
  **ChatGPT answer:** balloon
  **Score:** Correct
- **Prompts – Item 9:**
  He fell asleep and left the tv on for the whole ___
  **ChatGPT response: apple
  Score: Error A**
- **Prompts - Item 10:**
  All guests had a nice ___
  **ChatGPT response:** chair
  **Score: Error A**
- **Prompts - Item 11:**
  Bought treats in ___
  **ChatGPT response:** elephant
  **Score:** Correct
- **Prompts - Item 12:**
  Running away from home upset all his ___
  **ChatGPT response:** hot air balloon **Score: Error A**
- **Prompts - Item 13:**
  Now it's time to move on to ___
  **ChatGPT Response:** panda
  **Score: Error B**
- **Prompts - Item 14:**
  The wind was so strong that it blew open the ___
  **ChatGPT response:** trumpet
  **Score: Error A**
- **Prompts - Item 15:**
  Sometimes in the evening they make a little ___
  **ChatGPT response:** melon
  **Score: Error A**

**Compound Remote Association Task^[[2]](#footnote-2)^**

( Salvi et al., 2015 )

**Prompt – Instructions + Few Shot:**

For each problem you will be presented with three words. Your task is to find a fourth one that can be combined with each of the three presented, forming a compound word or phrase.

For example:
1) ABOVE - COMMON - SECOND.
The solution is NAME (nickname, common name, middle name).
2) ROMAN-FIRST-CIVIC

The solution is NUMBER (Roman numeral, prime number, house number)

3) FISHING-SOCIAL-TV

The solution is NETWORK (fishing net, social network, TV network)

4) BREAD-SOAP-MINERAL

The solution is water (bread and water, soap and water, mineral bread)

Now it's up to you. Fix the following problems:

| **Prompt - Problem** | **Solution** | **Answer ChatGPT** |
| --- | --- | --- |
| PRIORITY-ELECTRONIC-BOX | mail | mail |
| GRASS-BARBED-STRAIGHT | row | path |
| GOAT-SWISS-BUCHI | cheese | cheese |
| WHITE-CREDIT-IDENTITY | paper | paper |
| GAS-SUGAR-BAMBOO | cane | cane |
| EXPRESS-REGIONAL-CAPO | train | line |
| BERLIN-CRYING-CARRIER | wall | wall |
| SOFA-SINGLE-RIVER | bed | bed |
| JUDGMENT-BAD-PASTA | tooth | good |
| PEPPER-MINE-GROSSE | salt | black |
| APHRODITE-BOTTICELLI-STRABISMUS | Venus | Venus |
| LOSE-APPEAR-FLAT | calm | cheese |
| PARK-ROOM-CARDS | game | game |
| CARPET-TOMATO-FISH | red | machine |
| SAILOR-SHOE-TIE | node | blue |
| MEETINGS-DANCE-WAIT | room | room |
| GUILT-SIXTH-SIDE | sense | dark |
| BROTHER-DICTATOR-SMURF | great | greater |
| TRAVEL-INVITATION-LIST | wedding | check |
| ASTRAL-ELECTRIC-SWEDISH | Square | volvo |
| CUT-MALTESE-CIRCUIT | short | closure |
| LEGAL-EXACT-TIP | Now | precise |
| BAD-IRONY-EXTRACTION | fate | teeth |
| STRAW-NOON-CATCHER | fire | burn |
| SPADES-POINTS-WHEELS | two | cards |
| PUBLIC-TURKISH-BLOOD | bath | red |
| POTS-MUSIC-CHARGE | drums | drums |
| LANGUAGE-Street-CIVIL | code | code |
| CASSETTE-ADHESIVE-BLUE | tape | tape |
| LIGHT-BIRTHDAY-ENGINE | candle | candle |
| WOOD-GAS-PATATR | oven | fire |
| BLACK-COLLAR-RABBIT | white | white |
| NAME-DRESS-BOTTOM | above | dress |
| POOR-RULE-CONTEMPORARY | art | art |
| JUDAS-FRENCH-LADY | kiss | Black |
| MAJOR-MOUNTAIN-ARTIFICIAL | lake | snow |
| TREASURE-EASTER-PEDESTRIAN | island | egg |
| BUSINESS-CLEAN-FREE | square | exit |
| OIL-SCIENCE-WISHES | well | more |
| CIGLIO-MAESTRA-BABATUTE | street | funny |
| SKI-DANCE-LANDING | track | moon |
| GIFT-PAPER-BOMB | package | paper |
| BEAT-SHOE-PIN | heel | heel |
| PURPOSE-MEDAL-KNOB | gold | gold |
| OFF-OLD-BEATEN | iron | street |
| BETTING-CORNER-MARKET | soccer | black |
| BOY-MASTER-FAMILY | father | home |
| CAPITAL-DEATH-SUFFERING | sorry | impact |
| BLOOD-SKY-EYE | blue | red |
| FAKE-MOUNTAIN-DANCE | step | tango |
| TWENTY-DESERT-JERSEY | rose | sand |
| ELECTRIC-CURRENCY-MARINE | current | recharge |
| MAP-CORNER-TURN | world | street |
| IMPERFECT-FREE-REAL | time | time |
| FORMAT-HEAD-CHRISTIAN | family | paper |
| BLACK-OX-SHOT | eye | truncheon |
| INDIA-DRY-PLANT | fig | linden |
| DO-DOUBLE-CHICKEN | chest | chest |
| RETAIN-TRUMPET-NECK | breath | breath |
| LUCK-TRANSPORT-COMMUNICATION | half | postal |
| LUCK-FREE-STOCK | wheel | mare |
| COLD-LONG-PAUSE | coffee | dinner |
| SUMMARY-RETOUCHING-GRAPHY | photo | photographic |
| LONDON-EYES-ROAST | smoke | smoked |
| SUN-BAD-YEAR | light | stellar |
| BEAUTIFUL-PASTE-COAL | copy | paper |
| FOOT-BOTTLE-BAR | neck | dance |
| PRESTA-BEER-ROSE | plug | bear |
| MOBILE-MUSIC-EMERGENCY | stairs | service |
| SKIRT-GOLF-ERA | mini | fashion |
| BONES-CREAK-GOOSE | skin | egg |
| PSYCHE-FIRST-OWN | Love | first name |
| GAS-NEWS-CENTERS | leak | net |
| NEEDLE-WEIGHT-CONSTELLATION | balance | bear |
| INDEX-WITH-FOOT | finger | sit |
| STRONG-BAR-EARTH | floor | diagonal |
| LANGUAGE-LINE-PEARL | mother | necklace |
| SHOT-MILK-STARS | dust | machine |
| MUSICAL-TRY-HYPOTHETIC | period | syllable |
| MOTHER-VIDEO-PHONE | form | call |
| SCHOOL-ALL-TOMORROW | After | task |
| CAPE-HOPE-LINE | green | railroad |
| WORLDWIDE-RENTAL-STRAIGHT | motorcycle | car |
| VIOLIN-HOUSE-WORD | key | key |
| WRITTEN-WORK-LAUNDRY | hand | lyric |
| HEARTS-VICTORY-SKY | Queen | open |
| WEDDING-POWER-WEAK | ring | marriage |
| DEAD-FORM-TAKING OFF | weight | weight |
| LOCAL-HEALTHY-SICK | mind | air |
| CASE-PLACE-SENSE | common | common |
| PUBLIC-OUTSIDE-SACRED | place | brings |
| HOLE-FABRIC-HARD | bone | hole |
| ME-ALCOHOLIC-MAN | super | Alone |
| PARTY-FIRST-CANNON | woman | lymph |
| CHECK-CHECK-IVORY | tower | king |
| GOVERNMENT-GENTLEMAN-APARTMENT | thief | building |
| ULTIMATE-ZERO-LIGHT | year | annulment |
| MECHANIC-IRON-DEATH | arm | machine |
| POSITION-LOOSE-TURN | Jack | wrist |
| CONTINUE-FREE-CLASS | struggle | time |
| WHEELS-WINNER-BIG | wagon | fortune |
| ASH-SHEET-PACKAGES | brings | steering wheel |
| STOP-START-PASS | false | progress |
| HAND-DAILY-BUSINESS | Done | current |
| RED-INDIAN-RADIO | arrow | wave |
| SKIES-LION-SUN | king | African |
| MUTA-THEATER-CRIME | scene | costume |
| LOW-BRONZE-NORTH | League | statue |
| CLASS-ROAD-LAW | out | guide |
| CLASS-JOURNEY-ONBOARD | diary | Before |
| BATHROOM-SCENE-ENTIRE | costume | floor |
| DAMAGE-TURN-WORK | boss | black |
| WATER-BLACK-EAR | hole | heat |
| FOLLOW-CHINESE-GOVERNMENT | shadow | steps |
| DOG-DEAR-MINA | life | friend |
| FOOT-CENTRE-SNOW | ball | left |
| CONSCIOUSNESS-BLOCK-AUTO | place | green |
| BLUE-PEACOCK-FRIEND | quill | frederick |
| SUB-GENERAL-CATHOLLIC | culture | navy |
| FORCE-NIGHT-NEA | shirt | dark |
| LOGO-COUNTER-THESIS | pro | subject |
| DATE-HONOR-EAT | word | ceremony |
|  |  |  |

**Score Compound Remote Association Task: 21/122**

**Social cognition Battery**

( Prior et al., 2003 )

**Theory of Mind Test (TOM)**

- **Prompt – Instructions + Item 1:**The following are very short stories with protagonists. Answer the questions trying to get from the point of view of the mentioned character.

  Katia and Emma are two little girls who are playing at home. Emma takes a banana from the fruit bowl and holds it to her ear. He says to Katia: “Look! This banana is a telephone!”

  QUESTIONS: Is what Emma said true? Answer with YES or NO Why did Emma say this?**ChatGPT Answer:**No.
  Emma said this because she was playing a game and figuring the banana was a phone .
  **Score: 1**
- **Prompt – Item 2:**Giuseppe wanted to buy a kitten, so he went to see Mrs. Rossi who had many kittens that she couldn't keep. In reality , Mrs. Rossi loved the kittens and didn't want anyone to harm them, even though she couldn't keep them all. Giuseppe wasn't sure he wanted one of Signora Rossi's kittens. But Signora Rossi said: "If nobody buys the kittens I'll be forced to drown them!"

QUESTIONS: Is what Mrs. Rossi said true? Answer with YES or NO.
Why did Signora Rossi say this to Giuseppe?

**ChatGPT response:**No.
Mrs. Rossi told Giuseppe this to try to convince him to buy one of the kittens, probably because she fears that otherwise the kittens will end up in a situation of abandonment or maltreatment . However, threatening to kill animals is never acceptable or justifiable behavior. Ms Rossi should look for other solutions for the kittens she cannot keep, such as trying to place them with other people or with an animal welfare organisation.
**Score: 1**

- **Prompt – Item 3:**Gianni went to Chiara's house for the first time. Gianni arrived at Chiara's house, she opened the door for him and her dog ran to greet him. Chiara's dog is huge: he's more or less as big as Gianni! When Gianni saw Chiara's huge dog he said: “Chiara, you don't have a dog at all. You have an elephant!”

QUESTIONS: Is what Gianni said true? Answer with YES or NO. Why did Gianni say this? **ChatGPT response:**No.
Gianni said this because Chiara's dog seemed very big to him and he wanted to joke.
**Score: 1**

- **Prompts - Item 4:**One day, while playing house, Anna accidentally knocked over and broke her mother's favorite crystal vase. Anna knew her mother would be very angry! So when the mother came home and saw the broken vase and asked Anna what had happened, Anna said: "The dog knocked it over, it wasn't my fault!"

QUESTIONS:
Is what Anna told her mother true? Answer with YES or NO Why did Anna say this?

**ChatGPT response:**No.
Anna said this because she wanted to avoid her mother getting angry with her and to avoid taking the blame for the accident.
**Score: 1**

- **Prompts – Item 5:**
  Giovanni hates going to the dentist, because every time he goes he has to have a filling that hurts him a lot. But Giovanni knows that when he has a toothache, his mother always takes him to the dentist. Now Giovanni has a strong toothache, but when his mother notices that he is ill and asks him: "Do you have a toothache, Giovanni?", Giovanni replies :"No, mom".

QUESTIONS:Is what Giovanni says to his mother true? Answer with YES or NO. Why does John say this?**ChatGPT Answer:**No. Giovanni says this because he doesn't want to go to the dentist, because he's afraid of the pain the filling causes, and because he doesn't want his mother to worry.
**Score: 0**

- **Prompts - Item 6:**
  Elena waited all year for Christmas to ask her parents for a bunny. Christmas day arrived and Elena ran to open her present. She was sure it held a small rabbit in its cage. But when she opened it, with all the family standing around, she discovered that her present was a boring encyclopedia, which Elena didn't want at all! Then, when Elena's parents asked her how much she enjoyed her Christmas present, she said, “It's beautiful, thank you. That's exactly what I wanted."

QUESTIONS:
Is what Elena said true? Answer with YES or NO.
Why did Elena tell her parents this?

**ChatGPT response:**No. Elena said this because she didn't want to disappoint her parents and to avoid hurting their feelings, even though she was actually very disappointed with the gift she received. It could also be that Elena was polite and wanted to show gratitude for the gift, even if it wasn't what she wanted.
**Score: 1**

- **Prompt – Item 7:**One day Aunt Lucia came to visit Pietro. Usually Pietro likes his aunt very much, but today she was wearing a new hat which Pietro didn't like. Pietro thought his aunt looked ridiculous in that hat, and that she would look much better in the old one. But when Aunt Lucia asked Pietro: “Do you like my new hat?”, Pietro replied: “Oh, it is very beautiful”.

QUESTIONS:
Is what Pietro said true? Answer with YES or NO.
Why did Pietro say that?**ChatGPT response:**No. Pietro said this because he didn't want to hurt his aunt's feelings, even though he actually didn't like the hat. It could also be that Pietro wanted to be polite and show respect for his aunt's fashion choices.
**Score: 1**

- **Prompt – Item 8:**Late at night old Mrs. Bianchi is returning home. She doesn't like walking home alone in the dark because she is always worried about someone mugging and robbing her. Suddenly a man emerges from a shadow. He wants to ask Signora Bianchi what time it is, so he walks towards her. When Mrs. Bianchi sees the man walking towards her, she starts shaking and says: "Take my bag, but don't hurt me, please!"

QUESTIONS:
Was the man surprised by what Mrs. Bianchi said? Answer with YES or NO.
Why did she tell him that when he just wanted to ask what time it was?**ChatGPT response:** Yes.
Mrs. Bianchi told him this because she was very scared and was probably afraid of being attacked or robbed. It could also be that she had been the victim of an assault or similar incident in the past, which could have made her even more nervous and worried.
**Score: 0**

- **Prompt – Item 9:**A thief who had just stolen from a shop was running away. As he ran home, a policeman on duty saw him lose a glove. The policeman didn't know the man was a thief, and just wanted to tell him that he had lost a glove. But when the policeman yelled at the thief: “Hey, her! Stop!”, the thief turned around, saw the policeman and surrendered. With his hands in the air he admitted that he had committed the crime in the shop.

QUESTIONS:
Was the policeman surprised by what the thief said? Answer with YES or NO. - YES
Why did the thief behave like this, since the policeman just wanted to give him back his glove?**ChatGPT answer:** Yes.
The thief acted this way because he was probably afraid of being discovered and arrested for the crime he had committed. It could also be that he had had bad experiences with the police in the past and was led to believe that the police were his enemy. In any case, the thief probably decided to give up and confess to the crime to avoid prosecution or face even more serious consequences.
**Score: 0**

- **Prompt – Item 10:**One day Daniele and Luca see Mrs. Verdi coming out of the hairdresser's. It's kind of funny because the hairdresser cut her hair too short. Daniele says to Luca: "She must have been in a fight with a lawnmower!"

QUESTIONS:
Is what Daniele says true? Answer with YES or NO.
Why does Daniel say this?
**ChatGPT response:**
No.
Daniele says this as a joke, probably because he finds Mrs. Verdi's look with such short hair amusing. It could also be that Daniele is trying to make a joke or tease Mrs. Verdi in a playful way.
**Score: 1**

- **Prompt – Item 11:**Simone is a big liar and Massimo, Simone's brother, knows that Simone never tells the truth ! Just yesterday Simone stole Massimo's ping-pong racket, and Massimo knows that Simone has hidden it somewhere, although he can't find it. He's very angry. So he says to Simone: “Where is my ping-pong racket? You must have hidden it in the closet or under the bed, because I looked everywhere. Where is it, in the closet or under the bed?” Simone replies that it is under the bed.

**QUESTIONS:**Is what Simone told Massimo true? Answer with YES or NO.
Where will Massimo look for his ping-pong racket?
Why is he looking for his racket there?

**ChatGPT answer:**No.
Massimo will look for his ping-pong racket under the bed, because that's what Simone told him.
Massimo will look for his racket there because he believes that Simone is telling him the truth and he hopes to find the racket there. However, given that Simone is known to be a liar, it's likely that the racket isn't actually hidden under the bed.
**Score: 0**

- **Prompt - Item 12:**Anna's mother spent a lot of time cooking Anna's favorite dish: fish and chips. But when he brings it to Anna, she's watching TV, doesn't pay attention to her or even thank her. Anna's mother is angry and says: “Well, this is good behavior, don't you think? This is pure rudeness to me!”

QUESTIONS:
Is what Anna's mother said right? Answer with YES or NO. Why did Anna's mother say this?

**ChatGPT response:**
Yes. Anna's mother said this because she is angry and disappointed that Anna did not respect her labors in preparing Anna's favorite dish, nor did she show gratitude for the food she had cooked for her. Anna's lack of respect and recognition may have hurt her mother's feelings and made her angry.
**Score: 1**

- **Prompt – Item 13:**Marco and Filippo are having fun! They knocked the table over and sat on it. When their mother returns, she laughs and says: "What on earth are you doing?", "This table is a pirate ship!", says Philip, "and you better get in before you drown, because there you are in the sea ! "

**QUESTIONS:** Is what Philip says true? Answer with YES or NO.
Why does Philip say this?

**ChatGPT response:**
No. Philip says this as a joke or for fun, probably to try to make the situation less serious or to make his mother laugh. It could also be that Philip is trying to make an excuse or to justify the fact that he and Marco have overturned the table on the floor.
**Score: 0.5**

**Theory of Mind total score: 9.5/13**

**Emotion Attribution Test**

| **Prompts** | **Answer ChatGPT** | **Score** |
| --- | --- | --- |
| **1** Simone's paintings arrived last in a competition. *How will Simone feel in this situation?* | It's hard to say exactly how Simone will feel in this situation, as reactions to defeats can vary greatly from person to person. Some possible reactions could be disappointment, sadness, anger or frustration. However, it's important to remember that setbacks are a part of life and that there are always opportunities to learn and grow from them. It may be helpful for Simone to talk to someone about how she is feeling and find ways to deal with any negative emotions she may be experiencing. Also, it might be helpful for Simone to try to see the bright side of the situation, for example by considering what she has learned from the experience or looking for opportunities to improve in the future. | 1 |
| **2** Elisabetta is driving along a road when a woman suddenly appears in front of her and she runs right over her. *What will Elisabetta feel in this situation?* | In such a situation, Elisabetta is likely to experience a range of different and intense emotions. She may feel shocked, confused, anxious and scared. He may also feel guilty about running over the woman and worried about the consequences of the accident. In addition, she may be concerned about the woman's health and how she will deal with the situation. In this situation, it is important that Elisabetta try to remain calm and try to act responsibly, calling for help immediately and providing all the necessary information to the competent authorities. | 1 |
| **3** Maria has to give a speech at work. He's standing in the room facing everyone. He doesn't remember what to say. Everyone stares at her. *How will Maria feel in this situation?* | In this situation, Maria is likely to experience a variety of intense and stressful emotions. She may feel anxious, nervous, fearful and uncomfortable. She may also feel pressured, especially if it's important to her job or if she's particularly shy or insecure. Also, the fact that everyone is staring at her could amplify these emotions and make the situation even more difficult to deal with. In this situation, it is important that Maria try to remain calm and find ways to overcome the embarrassment and difficulty of the moment. It may help to take a deep breath, focus on your breathing, and try to relax as much as possible. Also, it may be helpful to have a contingency plan in case of difficulty, such as having a copy of the speech handy or asking a friend or colleague for help. | 1 |
| **4** Luca has just revealed himself to the girl he's been wanting for months. She slaps him across the face. *How will Luca feel in this situation?* | In this situation, Luca is likely to experience a series of intense and difficult to manage emotions. He may feel disappointed, hurt, sad, angry, and confused. He may also feel vulnerable and struggling, especially if his disclosure was made with a desire to form a deeper relationship or connection with the girl. Also, being slapped could add further pain and humiliation to the situation. In this situation, it is important for Luca to try to process his emotions and find ways to overcome the difficult moment. It may be helpful to talk to someone you trust, such as a friend or family member, or seek professional support, such as a therapist. Also, it may be helpful to take time for yourself and to process what happened, trying to understand your feelings and reactions. | 1 |
| **5** Enrico has just found out that he will get an extra pay raise. *How will Enrico feel in this situation* ? | In this situation, Enrico is likely to experience a variety of positive and rewarding emotions. He may be feeling happy, fulfilled, contented, and grateful for the pay raise. He may also feel proud of himself for his work and for the recognition he has received. Additionally, the increase in pay could bring with it greater financial security and greater economic freedom, which could further contribute to her emotional well-being. In this situation, it's important that Henry enjoys these positive emotions and celebrates his success appropriately, perhaps by sharing the good news with friends and family or by doing something special for himself . | 1 |
| **6** Rosanna has just been told by her boyfriend that he doesn't want to go out with her anymore . *How will Rosanna feel in this situation?* | In this situation, Rosanna is likely to experience a range of intense and difficult to manage emotions. She may feel hurt, disappointed, sad, angry, and confused. You may also feel vulnerable and struggling, especially if your relationship with your boyfriend has been important to you. Furthermore, the fact of having been left could bring with it a series of doubts and questions about itself and its value. In this situation, it is important for Rosanna to try to process her emotions and find ways to overcome the difficult moment. It may be helpful to talk to someone you trust, such as a friend or family member, or seek professional support, such as a therapist. Also, it may be helpful to take time for yourself and to process what happened, trying to understand your feelings and reactions. | 1 |
| **7** Carlo is lying in the middle of the forest. A poisonous spider lands on his chest. *What will Carlo feel in this situation?* | In this situation, Carlo is likely to experience a variety of intense and stressful emotions. He may feel fearful, anxious, panicked and uncomfortable. He may also feel vulnerable and in danger, especially if he's particularly shy or phobic or afraid of spiders. Also, having a venomous spider sitting on his chest could amplify these emotions and make the situation even more difficult to deal with. In this situation, it is important for Carl to try to remain calm and act responsibly, trying to get the spider away safely and asking for help if necessary. It may help to take a deep breath, focus on your breathing, and try to relax as much as possible. Also, it is important that Carlo tries not to get too excited, as this could make it more difficult for him to handle the situation and could also increase the risk of a bite. | 1 |
| **8** Giuseppe has just learned that his salary will be reduced next month. *How will Giuseppe feel in this situation?* | In this situation, Giuseppe is likely to experience a variety of negative and stressful emotions. He may feel disappointed, frustrated, angry and worried. He may also feel vulnerable and insecure, especially if his salary is important to his standard of living and if the reduction could have negative consequences on his financial situation. Additionally, you may be concerned about the possible long-term consequences of a pay cut, such as the possibility that you may face financial hardship or have to make sacrifices. In this situation, it is important for Joseph to try to process his emotions and find ways to deal with the situation responsibly. It may be helpful to talk to someone you trust, such as a friend or family member, or seek professional support, such as a financial advisor or therapist. Also, it might be helpful to make a plan to manage the pay cut and try to find solutions to mitigate the negative consequences of the situation. | 1 |
| **9** Iris has just learned that she has malignant cancer that could kill her in less than a year. *What will Iris feel in this situation?* | In this situation, Iris is likely to experience a range of intense and difficult to manage emotions. She may feel shocked, sad, angry, fearful and vulnerable. She may also feel overwhelmed with grief and a sense of loss and may have difficulty accepting the news. Additionally, she may be worried about her future and how she will deal with the illness, both physically and emotionally. In this situation, it is important for Iris to seek support from friends, family and professionals, such as a therapist or doctor, to help deal with the situation and manage her emotions. It may help to talk to someone you trust, such as a friend or family member, or join a support group for people who have been or are going through a similar situation. Also, it's important that Iris takes care of herself and tries to find ways to live as fully as possible despite her illness. | 1 |
| **10** Elena orders a sandwich in a restaurant. Eat the first bite and see the rest. There's a dead worm in the sandwich. *What will Elena feel in this situation?* | In this situation, Elena is likely to experience a variety of negative and unpleasant emotions. She may feel disgusted, nauseous, angry, and disappointed. She may also feel vulnerable and insecure, especially if she's particularly sensitive or phobic around bugs, or if she's afraid of catching illness or infection. In addition, she may be concerned about the consequences of the accident, such as having to look for another place to eat or having to go to the restaurant to ask for compensation or a refund. In this situation, it is important that Elena tries to remain calm and act responsibly, reporting the incident to the restaurant staff and asking for an appropriate solution. It may be helpful to speak to the restaurant owner or manager and provide any information needed to resolve the situation satisfactorily. Additionally, it may be helpful to seek support from friends or family members to manage the negative emotions of the incident. | 1 |
| **11** Paolo hears the sound that warns of the imminent fall of an atomic bomb *. How will Paolo feel in this situation?* | In this situation, Paolo is likely to experience a variety of intense and stressful emotions. He may feel fearful, anxious, panicked and vulnerable. He may also feel overwhelmed by fear and uncertainty about the future and may find it difficult to accept the situation. Furthermore, he may be concerned about the consequences of the explosion, such as the risk of death or serious injury, or about the fate of his loved ones. In this situation, it is important for Paolo to try to remain calm and act responsibly, following the instructions of the competent authority and trying to take cover as safely as possible. It may help to take a deep breath, focus on your breathing, and try to relax as much as possible. Also, it is important that Paolo tries to keep a realistic perspective and not panic, as this could make it more difficult for him to handle the situation and could also increase the risk of mistakes or reckless actions. | 1 |
| **12** A man walks up to Andrea and calls him an idiot *. How will Andrea feel in this situation?* | In this situation, Andrea is likely to experience a variety of negative and stressful emotions. He may feel offended, angry, hurt and vulnerable. He may also feel humiliated and scorned by the man's words and may find it difficult to accept this treatment. Furthermore, he may be concerned about the consequences of the insult, such as the risk of confrontation or violent reactions, or about his emotional well-being. In this situation, it is important that Andrea tries to remain calm and act responsibly, avoiding responding in an abusive or violent way and trying to protect himself and others from harm or danger. It may be helpful to talk to someone you trust, such as a friend or family member, or to search | 1 |
| **13** Tania was told that if she painted a woman's face, she would be paid 20,000 lire. But after she had completed the work she was given 10,000 lire *. How will Tania feel in this situation?* | In this situation, Tania is likely to experience a variety of negative and frustrating emotions. She may feel disappointed, angry, deceived and vulnerable. She may also feel humiliated and scorned by the woman's decision not to comply with the agreement and may find it difficult to accept this treatment. Additionally, she may be concerned about the consequences of the situation, such as the risk of missing out on future opportunities or facing financial hardship. In this situation, it is important for Tania to try to process her emotions and find ways to deal with the situation responsibly. It may be helpful to talk to someone you trust, such as a friend or family member, or seek professional support, such as a lawyer or financial advisor, if you feel you are entitled to compensation or a refund. Also, it may be helpful to make a plan to manage the consequences of the situation and try to find solutions to mitigate the financial difficulties. | 1 |
| **14** Fabio throws a book to a friend of his. His friend doesn't take it and the book ends up in another man's face.*What will Fabio feel in this situation?* | In this situation, Fabio is likely to experience a variety of negative and stressful emotions. He may feel guilty, angry, frustrated and vulnerable. You may also feel anxious and worried about the consequences of the accident, such as the risk of fights or violent reactions, or about your emotional well-being. Additionally, he may be concerned for the other man's well-being and how the incident might have hurt or disturbed him. In this situation, it is important for Fabio to try to remain calm and act responsibly, trying to clarify the situation with the other man and apologizing if he feels he has done something wrong. It may be helpful to talk to the other man and try to understand his point of view and work out a solution to resolve the incident satisfactorily. Furthermore, it is important that Fabio tries to avoid repeating similar behaviors in the future | 0 |
| **15** Roberto tunes into the news radio and hears that his neighbors have just won 200 million in the lottery. His neighbor is telling the reporter about all the trips he will take and all the things he will buy .*What will Roberto feel in this situation?* | In this situation, Roberto is likely to experience a variety of negative and frustrating emotions. He may feel envious, angry, disappointed and vulnerable. He may also feel frustrated and worried about how the good fortune of his neighbors might affect his life, and he may find it difficult to accept the situation. Additionally, he may be concerned about the consequences of his neighbors winning, such as the risk of missing out on opportunities or facing financial hardship. In this situation, it is important for Roberto to try to process his emotions and find ways to deal with the situation responsibly. It may be helpful to talk to someone you trust, such as a friend or family member, or seek professional support, such as a therapist, if you feel you need help dealing with the negative emotions of the incident. Also, it is important that Roberto tries to keep a realistic perspective and not get envied or frustrated, as this could make it more difficult for him to handle the situation and could also increase the risk of mistakes or reckless actions. | 1 |
| **16** Romina hasn't seen her husband for weeks. Then she sees him in the street and runs towards him. *How will Romina feel in this situation?* | In this situation, Romina is likely to experience a series of positive and pleasant emotions. She may feel happy, surprised, grateful, and relieved. She may also feel excited and happy to see her husband again and may have the impulse to run towards him to hug him and show her affection. In addition, she may be concerned about her husband's well-being and may want to talk to him to understand how he is and to find out what has been keeping him away for so long. In this situation, it is important for Romina to try to experience her emotions and to express them appropriately, trying to respect her husband's needs and emotions and to communicate openly and sincerely. It might be helpful to talk to your husband and try to understand his point of view and find | 1 |
| **17** Daniele is walking in a crowded street when he trips and hits his face. Everyone in the street turns, stares at him and starts laughing *.**How will Daniele feel in this situation?* | In this situation, Daniele is likely to feel embarrassed and vulnerable, especially if everyone in the street turns to look at him and laugh. He may also feel frustrated or angry at being laughed at or teased. Everyone reacts differently to embarrassing situations, but it's understandable that Daniele might feel uncomfortable right now. | 1 |
| **18** Tania is listening to the lottery numbers. She has already scored five numbers and is close to winning 10 million.*How will Tania feel in this situation?* | In this situation, Tania is likely to feel excited and happy. She may also feel surprised or incredulous, especially if she didn't expect to win so much money. It is normal for people to feel happy when they are successful or receive something positive, such as winning a significant amount of money. Tanya may also feel a little nervous or anxious if she is not used to handling large amounts of money or if she doesn't know how to use it effectively. In any case, Tania is likely to feel happy and satisfied in this situation. | 1 |
| **19** Every week at Giovanni's job they take a prize away from whoever is doing worse. This week it was Giovanni's turn.How will John feel in this situation? | In this situation, Giovanni is likely to feel frustrated, disappointed, and perhaps even angry. He may feel as though he has been treated unfairly or that his prize has been taken away from him for no good reason. He may also feel demoralized or discouraged, especially if this happens often. Everyone reacts differently to negative or challenging situations, but it's understandable that John might feel uncomfortable right now. | 1 |
| **20** Katia received the results of her exams: they went very well. *How will Katia feel in this situation?* | In this situation, Katia is likely to feel happy, satisfied, and proud of herself. She may also feel relieved or grateful that she has achieved good results, especially if she has worked hard to achieve them. It is normal for people to feel happy when they are successful or achieve something important to them, such as doing well in exams. Katia may also feel motivated to keep doing well and to overcome future challenges. In any case, Katya is likely to feel positive and satisfied in this situation. | 1 |
| **21** Melania is in an alley. A man is walking towards her with a knife *. What will Melania feel in this situation?* | In this situation, Melania is likely to feel fearful and in danger. She may also feel vulnerable and helpless, especially if the man with the knife is approaching and doesn't seem willing to stop. She may also feel worried or anxious about her well-being and that of loved ones. It is normal for people to feel scared when they are in danger or under threat. In this situation, it would be understandable for Melania to experience a strong emotion of fear. | 1 |
| **22** Elisabetta recently had a new boyfriend *. How will Elizabetta feel in this situation?* | In this situation, Elisabetta is likely to feel happy and content that she has a new boyfriend. She may also feel excited and eager to find out what the future holds for her and her new partner. It's normal for people to feel positive and optimistic when starting a new relationship, especially if they feel happy and fulfilled in their new partnership. However, everyone reacts differently to positive or emotional situations, so there's no one-size-fits-all way to describe how Elizabetta will feel in this situation. She may also feel a little nervous or worried about how the relationship is going to go, but in general she is likely to feel happy and satisfied that she has a new boyfriend. | 1 |
| **23** Riccardo'scar , who is 20 years old, has just broken down on the road. As he sits there, someone drives by in a new Jaguar. Riccardo watches the car go by.*How will Riccardo feel in this situation?* | In this situation, Riccardo is likely to feel frustrated and grumpy. He may also feel envious or sad that he can't afford a fancy new car like the one he just saw driving by. He may also feel worried or anxious about how to fix his broken car and how to get to his destination. Everyone reacts differently to negative or challenging situations, but it is understandable that Riccardo may feel uncomfortable right now. | 1 |
| **24** Giulia is standing in the center of the wedding hall to give a speech. But while she's on her feet she can't say anything. Everyone in the room stares at her and someone starts laughing. *How will Giulia feel in this situation* ? | In this situation, Giulia is likely to feel embarrassed and vulnerable. She may also feel frustrated or angry about being teased or not being able to deliver the speech as expected. She may also feel nervous or anxious about being judged by others. Everyone reacts differently to embarrassing situations, but it's understandable that Giulia might feel uncomfortable right now. | 0 |
| **25** Elisa would like a bunny for her birthday, but has been told by her parents that she will be given a bicycle, but she doesn't want it. But, when he goes down to open his gift, he discovers that there is a bunny inside.*How will Elisa feel in this situation?* | In this situation, Elisa is likely to feel happy and content that she got the bunny she wanted. She may also feel surprised and grateful for her parents' gesture. It's normal for people to feel happy when we get something important or meaningful to us, like a long-desired gift. Elisa may also feel a little guilty or embarrassed for not wanting the bike that was initially offered to her. In any case, Elisa is likely to feel happy and satisfied in this situation. | 1 |
| **26** Silvia wakes up and sees that there is a poisonous spider in her bed. *What will Silvia feel in this situation?* | In this situation, Silvia is likely to feel scared and in danger. She may also feel vulnerable and helpless, especially if the venomous spider is getting close or seems ready to bite her. She may also feel worried or anxious about her well-being and that of loved ones. It is normal for people to feel scared when they are in danger or under threat. In this situation, it would be understandable for Silvia to feel a strong emotion of fear. | 1 |
| **27** Giacomo has just prepared a bowl of corn flakes and milk. He eats a spoonful and realizes the milk has gone bad. What will Giacomo feel in this situation? | In this situation, Giacomo is likely to feel disgusted and nauseated by the taste and smell of spoiled milk. He may also feel disappointed or frustrated that he has cooked himself a breakfast that he cannot eat. He may also feel worried or anxious about his well-being, especially if he's already had a spoonful of spoiled milk. It is common for people to feel disgusted or nauseated by foods or drinks that smell or taste bad. In this situation, it would be understandable for Giacomo to feel a strong emotion of disgust. | 1 |
| **28** Sonia is riding her bicycle along the road when she loses control and crashes into a lamppost. A few people across the street stop and stare at her; someone mocks her. How will Sonia feel in this situation? | In this situation, Sonia is likely to feel embarrassed and vulnerable. She may also feel frustrated or angry at being laughed at or teased by other people. She may also feel sore or injured if she fell off her bike and hurt herself. Everyone reacts differently to awkward or challenging situations, but it's understandable that Sonia might feel uncomfortable right now. | 1 |
| **29** A man walks towards Maximus and threatens to punch him. *How will Massimo feel in this situation?* | In this situation, Massimo is likely to feel scared and in danger. He may also feel vulnerable and helpless, especially if the man threatening him is older or stronger than him. He may also feel concerned or anxious about his well-being and that of the people around him. It is normal for people to feel scared when they are in danger or under threat. In this situation, it would be understandable for Massimo to feel a strong emotion of fear. | 1 |
| **30** William has just gone on a first date with a girl he has been interested in for four years. At the end of the date they kiss.*How will William feel in this situation?* | In this situation, William is likely to feel happy and satisfied. He may also feel excited and happy that he finally got what he wanted. He may also be feeling romantic and emotionally invested in the relationship. It's normal for people to feel happy and content when we get something important or meaningful to us, like a date or a kiss with someone we're interested in. In this situation, it would be understandable for William to feel a strong emotion of joy and satisfaction. | 1 |
| **31** Margherita has just found out that her mother is dead. *How will Margherita feel in this situation?* | In this situation, Margherita is likely to feel devastated and very sad. She may also feel confused, distraught, overwhelmed with pain and shock. The death of a mother can be a very traumatic and difficult event to deal with, especially if the person was particularly close to the mother. Margherita may also feel lonely and vulnerable without her mother in her life. In this situation, it would be understandable for Margherita to feel a strong emotion of pain and sadness. | 1 |
| **32** Giovanna has just been given a promotion. *How will Giovanna feel in this situation?* | Giovanna may feel very satisfied and happy about having received a promotion. She may also feel surprised or proud of herself for getting this recognition of her work and effort. She may also feel a little nervous or worried about the additional responsibilities or expectations that may come with her new position. However, in general, Giovanna is likely to feel very satisfied and gratified at having received a promotion. | 1 |
| **33** Sara has parked on the street corner when a woman arrives and hits her car with a hammer, severely damaging the bonnet.*How will Sara feel in this situation?* | Sara may feel very angry, frustrated and upset about this situation. She may also feel surprised and shocked that someone has intentionally damaged her car in this way. She may also feel worried about the cost of repairing the car and about having to deal with insurance and possible legal consequences. Overall, Sara is likely to feel very annoyed and disturbed by this incident. | 1 |
| **34** Rebecca is getting her Sunday lunch out of the fridge when she notices worms crawling around.*How will Rebecca feel in this situation?* | Rebecca may feel very disgusted and nauseous in this situation. She may also feel angry or annoyed that the food she thought about eating was contaminated with worms. She may also feel concerned about her health and about having to find another meal instead of the lunch she had planned to eat. In general, Rebecca is likely to feel very uncomfortable and upset by this situation. | 1 |
| **35** Arturo is talking to a man he doesn't know very well. The man is eating a sandwich. While they are talking the man spits a piece of food into Arturo's jacket. Arturo grabs the man and rips his shirt to pieces.*How will Arturo feel in this situation?* | In this situation, Arturo is likely to feel very angry and annoyed. He may also feel offended and disgusted by the man's rude behavior and by the fact that he spit food on his jacket. He may also feel embarrassed or embarrassed by his impulsive behavior of grabbing and tearing the man's shirt. In general, Arturo is likely to feel very frustrated and angry in this situation. | 1 |
| **36** Nadia was told that her next job would be in Rome, her favorite city. Instead she will be in Padua, a city she doesn't like *.**How will Nadia feel in this situation?* | Nadia may feel very disappointed and frustrated in this situation. She may also feel angry or annoyed that she was given incorrect news or that plans were changed without her consent. She may also feel worried about having to move to a city she doesn't like and about the possible difficulties that could arise as a result of this change. In general, Nadia is likely to feel very dissatisfied and discouraged by this situation. | 1 |
| **37** Giampaolo is camping in the forest in America. A bear rips off part of his tent and comes towards him growling.*How will Giampaolo feel in this situation?* | Giampaolo could feel very scared and worried in this situation. He may also feel vulnerable and helpless in the face of the approaching bear growling. He may also feel confused or disoriented about what to do or how to protect himself. In general, Giampaolo is likely to feel very scared and concerned for his safety in this situation. | 1 |
| **38** Andrea , unemployed, has just been offered a new job. *How will Andrea feel in this situation?* | Andrea may feel very happy and satisfied to have received a job offer. He may also feel relieved and grateful that he found a job opportunity at a time when he was unemployed. He may also feel a little nervous or worried about the responsibilities and expectations of his new job. However, in general, Andrea is likely to feel very happy and gratified to have received this job offer. | 1 |
| **39** Simone realizes that the man next to him has the gold watch that he has been wanting to buy for three years. Simone knows she won't be able to afford that watch for another few years.*How will Simone feel in this situation? How will Sonia feel in this situation?* | Simone could feel very envious and frustrated in this situation. He may also feel disappointed and discouraged that he can't afford the watch he craves. He may also feel worried that he will have to wait longer to afford it. Sonia, on the other hand, may not feel involved or interested in the watch in question at all and therefore may not have any particular emotion in this situation. Or, if Sonia shares Simone's interest in the watch, she may feel disappointed and frustrated with him that they can't afford to buy it now. | 1 |
| **40** Sonia'sfriend told her that if she looked after her children on Monday she would look after Sonia's children on Thursday . Sonia looked after her friend's children on Monday , but on Thursday her friend told her that she could not look after Sonia's children because she had another appointment. *How did Sonia feel in this situation?* | Sonia may feel very disappointed and angry in this situation. She may also feel betrayed and hurt that her friend didn't keep her word. She may also feel worried about having to find another way to watch her children on Thursdays. In general, Sonia is likely to feel very frustrated and angry at her friend's lack of respect for the deal they had made. | 1 |
| **41** Mario was called by his boss. This tells him : ” You are fired.” *How will Mario feel in this situation?* | Mario may feel very shocked, surprised and disappointed in this situation. He may also feel angry or frustrated that he was fired without warning or explanation. He may also feel worried about his job future and how he will support himself and his family without work. In general, Mario is likely to feel very disconcerted and unhappy to have received this news. | 1 |
| **42** Daniele's wife has just given birth to their third child. *How will Daniele's wife feel in this situation* ? | Daniele's wife may feel very tired and in pain after giving birth. She may also feel excited and grateful that she gave birth to another healthy child. She may also feel worried about having to deal with three children and how the new arrival will affect the family routine. However, in general, Daniele's wife is likely to feel very happy and satisfied with having given birth to her third child. | 0 |
| **43** Fulvio is in the bank in the middle of a robbery. One of the robbers points a gun in his face. *How will Fulvio feel in this situation?* | Fulvio could feel very scared and terrified in this situation. He may also feel vulnerable and helpless in the face of the robber pointing a gun in his face. He may also feel confused or disoriented about what to do or how to protect himself. In general, Fulvio is likely to feel very scared and concerned for his safety in this situation. | 1 |
| **44** Giusy runs inside the cinema and doesn't see the glass door. He runs against us. Everyone in the line turns and stares at her and some laugh at her.*How will Giusy feel in this situation?* | Giusy could feel very embarrassed and mortified in this situation. She may also feel surprised and shocked that she didn't see the glass door and crashed into it. She may also feel angry or frustrated that someone laughed at her. In general, Giusy is likely to feel very unhappy and uncomfortable about this embarrassing incident. | 1 |
| **45** Katia has just discovered that 10 million have been stolen from her and she can't do anything to get it back. *How will Katia feel in this situation?* | Katia may feel very angry, frustrated and helpless in this situation. She may also feel vulnerable and helpless in the face of having suffered such a massive theft. She may also feel worried about having to deal with this financial loss and how it might affect her life. In general, Katya is likely to feel very annoyed and upset by this situation. | 1 |
| **46** Mauro has woken up and hears a thief moving in the house. *How will Mauro feel in this situation* ? | Mauro might feel very scared and worried in this situation. He may also feel vulnerable and helpless in the face of a burglar in his home. He may also feel confused or disoriented about what to do or how to protect himself. In general, Mauro is likely to feel very scared and concerned for his safety in this situation. | 1 |
| **47** Edy is in a bar when he slips in a grease patch and falls. Everyone in the bar stares at him. *How will Edy feel in this situation?* | Edy may feel very embarrassed and mortified in this situation. He may also feel surprised and upset that he lost his balance and fell. He may also feel angry or frustrated that he has been the subject of so much interest or ridicule from others in the café. Overall, Edy is likely to feel very unhappy and uncomfortable about this embarrassing incident. | 1 |
| **48** Enrico is walking along a street in the town. Unfortunately he does not see a pebble. The bike swerves and he falls into the grass. A car stops and everyone sees him fall. *What will Enrico feel in this situation?* | Enrico could feel very embarrassed and mortified in this situation. He may also feel surprised and upset that he lost his balance and fell. He may also feel angry or frustrated that he has been the subject of so much interest or ridicule from others on the road. Overall, Enrico is likely to feel very unhappy and uncomfortable about this embarrassing incident. | 1 |
| **49** Every time Nicoletta tries to work on the computer she doesn't find a free one at work. *What will Nicoletta feel in this situation?* | Nicoletta could get very frustrated and annoyed in this situation. She may also feel angry or disappointed that she can't find a free computer at work. She may also feel concerned about not being able to complete her tasks or meeting deadlines due to lack of access to a computer. In general, Nicoletta is likely to feel very annoyed and bewildered by this situation. | 1 |
| **50** Alessandro is watching from the railing of a bridge. Suddenly it begins to slide and goes straight into the stream. As he climbs out of the water he notices a bus full of tourists staring at him.*How will Alessandro feel in this situation?* | Alessandro could feel very embarrassed and mortified in this situation. He may also feel surprised and shocked that he slipped off the railing of the bridge and fell into the water. He may also feel angry or frustrated that he has received so much interest or ridicule from the tourists on the bus. Overall, Alexander is likely to feel very unhappy and uncomfortable about this embarrassing incident. | 1 |
| **51** Gianni is taken on a safari. The car broke down and now a big rhino is attacking him. *How will Gianni feel in this situation?* | Gianni may feel very scared and terrified in this situation. He may also feel vulnerable and helpless in the face of the rhino attacking him. He may also feel confused or disoriented about what to do or how to protect himself. In general, Johnny is likely to feel very scared and concerned for his safety in this situation. | 1 |
| **52** Emilia is walking hand in hand with her lover along the road. Her husband suddenly appears around the corner and surprises them together.*What will Emilia feel in this situation?* | Emilia may feel very embarrassed, mortified and worried in this situation. She may also feel surprised and shocked that she was caught by her husband while she was walking hand in hand with her lover. She may also feel angry or frustrated that she has been caught and has to deal with the consequences of her actions. In general, Emilia is likely to feel very unhappy and uncomfortable about this embarrassing situation. | 1 |
| **53** Simone catches a thief stealing her wallet from her purse. *How will Simone feel in this situation?* | Simone may feel very angry, frustrated and helpless in this situation. He may also feel vulnerable and helpless in the face of the thief who is stealing his wallet. You may also feel worried about having to deal with this loss and how it might affect your life. In general, Simone is likely to feel very annoyed and bewildered by this situation. | 1 |
| **54** Filippo had entered a fishing competition and wanted to win. So he bought a big fish from the fishmonger. Unfortunately one of the judges was the owner of the shop. This man tells everyone what Philip did.*What will Philip feel in this situation?* | Filippo may feel very embarrassed, mortified and disappointed in this situation. He may also feel surprised and shocked that he was discovered by the judge who also owned the shop he bought the fish from. He may also feel angry or frustrated that he was caught and has to deal with the consequences of his actions. In general, Filippo is likely to feel very unhappy and uncomfortable about this embarrassing situation. | 1 |
| **55** Priscilla'sboss told her to do her job a certain way. Then, at the end of the day, after she's worked for hours, he changes his mind and wants something different. *How will Priscilla feel in this situation?* | Priscilla might get very frustrated and annoyed in this situation. She may also feel angry or disappointed that her boss told her to do the job a certain way, only to change her mind at the end of the day. She may also feel worried about having to redo the job or dealing with the consequences of the boss changing her mind. In general, Priscilla is likely to feel very annoyed and bewildered by this situation. | 1 |
| **56** Paolo made a contract with a man. If he sends the man his record collection, the man gives him a car. Paolo sends the man his record collection. However, the man does not give him the car as agreed.*How will Paolo feel in this situation?* | In this situation, Paolo is likely to feel disappointed, frustrated, or angry that the man didn't fulfill his contract and didn't give him the car as promised. He may feel cheated or cheated by the man and may be tempted to do something about the situation. However, it is important to consider that each person reacts differently to circumstances and unmet expectations, so it is possible that Paul reacted differently than one would expect. | 1 |
| **57** Matteo was told that he would have his favorite toy for Christmas. Instead he was given an encyclopedia.*How did Matteo feel in this situation?* | It's hard to say exactly how Matteo would have felt in this situation without having more information about him and his personality. However, it is likely that Matteo would have felt disappointed or frustrated that he did not receive his favorite game as a Christmas present. He might even be surprised or surprised to receive an encyclopedia as a gift instead. In any case, it is important to consider that each person reacts differently to circumstances and unmet expectations, so it is possible that Matteo reacted differently than one would expect. | 1 |
| **58** Anna'sparents all expect excellent grades from her in her exams. She opens the results letter in front of them. Everyone sees that he has taken just enough in each exam. *How will Anna feel in this situation?* | In this situation, Anna is likely to feel disappointed, frustrated, or nervous that she only got passing marks in her exams, especially if her parents expected top marks from her. She may feel pressured or guilty about not meeting her parents' expectations and may be concerned about how her parents will react to her achievements. However, it is important to bear in mind that each person reacts differently to circumstances and unmet expectations, so it is possible that Anna has reacted differently than one would expect. | 0 |

**Score Sadness** (items 1,4,6,8,9,19,31,36,41,57) **: 9/10**

**Fear score** (items 2,7,11,21,26,29,37,43,46,51) **: 10/10**

**Score Embarrassment** (items 3,14,17,24,28,44,47,48,50,52,54,58) **: 8/12**

**Score Happiness** (items 5,16,18,20,22,25,30,32,38,42) **: 9/10**

**Score Disgust** (item 10,27,34) **: 3/3**

**Score Rage** (items 12,13,33,35,40,45,49,53,55,56) **: 10/10**

**Score Envy** (items 15,23,39) **: 3/3**

**Test of Social Situations^[[3]](#footnote-3)^**

- **Prompt - Item 1a :**Carlo was an employee who worked in an office in his city. At noon he dined in a small park. He often broke part of a sandwich into small pieces, scattering them in the ground for the pigeons.

  A - Normal behavior in that situation
  B - Somewhat strange behavior in that situation
  C - Quite strange behavior in that situation
  D - Extremely strange behavior in that situation

  **ChatGPT response:**
  For the first situation, I would choose answer B - Behavior a little strange in that situation. Breaking the sandwich into small pieces and scattering them on the ground to feed the pigeons could be considered a bit of a strange behavior, especially if this is a habit that Carlo has during every lunch break. It's not necessarily inappropriate or bad behavior, but it might be considered a bit unusual.
- **Prompt - Item 1b:**
  One day, while sitting on the bench, a stroller was parked right there in front. Carlo noticed that nearby a young woman was swinging a slightly older child. The baby in the stroller started crying but the mother didn't hear him. Carlo has learned that when his little nephew cries, it sometimes means that his diaper has opened. Rather than disturb his mother in the park, Carlo quickly
  checked the baby's clothes to see if he could feel the open diaper.A - Normal behavior in that situation
  B - Somewhat strange behavior in that situation
  C - Quite strange behavior in that situation
  D - Extremely strange behavior in that situation

  **ChatGPT response:**
  I would choose answer A - Normal behavior in that situation for the second situation described. Carlo proved to be attentive and considerate towards the child, trying to help his mother without disturbing too much. Offering help in this way can be considered normal and kind behavior in this situation.
- **Prompt - Item 2a :**Emilia woke up late on the day of her plane ride. There was barely
  time to get dressed and go to the airport, so she skipped her breakfast. A - Normal behavior in that situation
  B - Somewhat strange behavior in that situation
  C - Quite strange behavior in that situation
  D - Extremely strange behavior in that situation

  **ChatGPT response:**
  I would choose answer A - Normal behavior in that situation. In this situation, skipping breakfast for the plane ride could be considered normal behavior, as Emilia needs to hurry to get to the airport on time. Skipping breakfast in a rush or when you're late is not unusual or strange behavior.
- **Prompt - Item 2b:**At noon the stewardess came with lunch, but Emilia was so hungry
  that one portion didn't satisfy her. He watched a little girl across the aisle fiddling with her food, moaning, "I can't eat it." Emilia leaned out of the corridor and said: "If your little girl doesn't want her tray, she can pass itthis way for me?
  A - Normal behavior in that situation
  B - Somewhat strange behavior in that situation
  C - Quite strange behavior in that situation
  D - Extremely strange behavior in that situation

**ChatGPT response:**I would choose answer B - Behavior a little strange in that situation for the description given. Even though Emilia might be hungry and eager to eat more, asking for the little girl's tray might be considered a little strange or inappropriate. It may be more normal to ask the cabin crew if it is possible to have another portion or if there are other food options available .

- **Prompt - Item 3a :**Ruggero lived alone and one day he was invited to dinner at the house of a friend of his mother. Generally he used to warn the waitress that he didn't eat meat and that he would like his vegetables to be served plain. When Ruggero arrived at the appointment he remembered that he hadn't touched food for two hours *.* Without wasting any time, even before being introduced, he asked the waitress when dinner would be served.

  A - Normal behavior in that situation
  B - Somewhat strange behavior in that situation
  C - Quite strange behavior in that situation
  D - Extremely strange behavior in that situation

**ChatGPT answer:**
I would choose answer C - Pretty weird behavior in that situation. Even though Ruggero used to advise the waitress of his food preference, asking when dinner would be served as soon as he arrived at the appointment could be considered a bit strange. It's not entirely inappropriate, but it could be considered a bit inappropriate or impatient.

- **Prompt - Item 3b:**
  She replied that it would be another hour before the food was ready *.*Hearing this , Ruggero opened his briefcase, took out an apple and some walnuts and promptly ate them. A - Normal behavior in that situation
  B - Somewhat strange behavior in that situation
  C - Quite strange behavior in that situation
  D - Extremely strange behavior in that situation

**ChatGPT answer:**
I would choose answer A - Normal behavior in that situation for the description given. Ruggero said he hadn't touched food in two hours and just learned it would be another hour before the food was ready. In this situation, eating an apple and some nuts to quench your hunger could be considered normal and understandable behavior. There is nothing strange or inappropriate about this behavior.

- **Prompt - Item 3c:**Just before dinner, the waitress showed him an attractive platter of fruits and
  vegetables, asking if that seemed enough. "Okay, thank you," he said Ruggero - “but if you don't mind , I'd wait another hour to eat. I already took something an hour ago”A - Normal behavior in that situation
  B - Somewhat strange behavior in that situation
  C - Quite strange behavior in that situation
  D - Extremely strange behavior in that situation

**ChatGPT response:**
I would choose answer B - Behavior a little strange in that situation for the description given. Even though Ruggero may have already grabbed something to eat an hour earlier, refusing the food offered by the waitress before dinner could be considered a little strange or inappropriate. It might be more normal to thank for the food offered and ask if it is possible to eat it later or at another time in the evening.

- **Prompt - Item 4:**Elizabeth had been diabetic all her life. Doctors had told her to be very
  careful with her diet to avoid serious complications. When invited to lunch by someone, she would explain the problem beforehand *.* But during
  important meetings he enjoyed the conversation and company he found, waiting to go home to eat.A - Normal behavior in that situation
  B - Somewhat strange behavior in that situation
  C - Quite strange behavior in that situation
  D - Extremely strange behavior in that situation

  **ChatGPT response:**
  I would choose answer A - Normal behavior in that situation for the description given . Elizabeth has a diabetic condition and must follow a carefully planned diet to avoid complications. In this situation, explaining the problem in advance when invited to lunch and waiting to get home to eat during important meetings could be considered normal and responsible behavior. There is nothing strange or inappropriate about this behavior.
- **Prompt - Item 5a :**Franco was a gardener. Packed lunches were usually brought *.* At noon, Franco always washed his hands under a tap and sat in a shady part of the garden to eat.

  A - Normal behavior in that situation
  B - Somewhat strange behavior in that situation
  C - Quite strange behavior in that situation
  D - Extremely strange behavior in that situation

  **ChatGPT response:**I would choose answer A - Normal behavior in that situation for the description given . Franco is a gardener and usually brings his own packed lunch. Washing hands under a tap and sitting in a shady part of the garden to eat during the lunch break could be considered normal and appropriate behavior for this situation. There is nothing strange or inappropriate about this behavior.
- **Prompt - Item 5b:**
  One day it started to rain at noon. Franco knocked on the door of the
  house where he worked and asked permission to eat inside. The lady told him to come in and he spotted the dining room by himself. He wiped the crumbs off the table and looked around the house for a place to rest.

  A - Normal behavior in that situation
  B - Somewhat strange behavior in that situation
  C - Quite strange behavior in that situation
  D - Extremely strange behavior in that situation

  **ChatGPT response:**I would choose answer A - Normal behavior in that situation for the description given . Franco was invited by the lady to come into the house to eat during the lunch break due to the rain. Looking for a place to rest and wiping crumbs off the table before sitting down to eat could be considered normal and respectful behavior in this situation. There is nothing strange or inappropriate about this behavior.
- **Prompt - Item 5c:**The carpet in the living room was thin, so Franco decided to curl up for his nap on a large armchair.

  A - Normal behavior in that situation
  B - Somewhat strange behavior in that situation
  C - Somewhat strange behavior in that situation
  D - Extremely strange behavior in that situation

  **ChatGPT Response:**I would choose answer B - Somewhat strange behavior in that situation for the description provided. Even though Franco might be tired and want to take a nap during his lunch break, choosing to curl up in a large armchair in someone's living room might be considered a little strange or inappropriate. It may be more normal to look for a more discreet or appropriate place to nap, such as an empty room or a comfortable chair.
- **Prompt - Item 6:**
  The supermarket where Roberto used to shop had a small sign on the door that said: "It is forbidden to enter this warehouse barefoot". One summer day Roberto saw a beautiful girl enter the warehouse barefoot. Roberto decided to make sure that her feet were not seen by the director. He wheeled his cart past and ahead of her, aisle after aisle.

  A - Normal behavior in that situation
  B - Somewhat strange behavior in that situation
  C - Quite strange behavior in that situation
  D - Extremely strange behavior in that situation

  **ChatGPT Response:**I would choose answer D - Extremely strange behavior in that situation for the description provided. Roberto has decided to follow a stranger through the supermarket and to block her view with the trolley to prevent the director from seeing her barefoot, despite the ban on entering the warehouse barefoot. This behavior could be considered extremely strange and inappropriate. There is no reason why Roberto should interfere with other customers in the supermarket in this way.
- **Prompt - Item 7a :**Matteo has just been nominated in an important new job. At 6 o'clock on the day when he planned to start his new job, Matteo received a phone call from his mother who was very ill. Matteo was very worried and so he pulled a sweater over his pajamas and drove to his mother's house to take care of her. A - Normal behavior in that situation
  B - Somewhat strange behavior in that situation
  C - Quite strange behavior in that situation
  D - Extremely strange behavior in that situation **ChatGPT response:**I would choose to answer A - Normal behaviour in that situation for the description given. Matteo has just been appointed to an important new job, but his mother is very ill and needs his help. In this situation, wearing a sweater over her pyjamas and driving to her mother's house to nurse her could be considered normal and responsible behaviour. There is nothing strange or inappropriate about this behaviour.
- **Prompt - Item 7b:** After the doctor arrived, there was not enough time for Matteo to go back to his house and change his clothes. He decided to go to work in his pyjamas because he didn't want to be late for his new job.

  A - Normal behaviour in that situation
  B - Somewhat strange behaviour in that situation
  C - Fairly strange behaviour in that situation
  D - Extremely strange behaviour in that situation
  **ChatGPT Response:**I would choose answer C - Fairly strange behaviour in that situation for the description provided. While Matteo had a good reason to wear his pyjamas on his journey to his new job, going to work in pyjamas might be considered quite strange or inappropriate by some people. It might be more normal to try to find a way to change your clothes before going to work or explain the situation to your boss or coworkers.
- **Prompt - Item 8a:**Giovanna went with her three-year-old daughter to the airport. There were very long lines of people waiting to check in their bags. Giovanna joined the queue with her daughter. A - Normal behaviour in that situation
  B - Somewhat strange behaviour in that situation
  C - Quite strange behaviour in that situation
  D - Extremely strange behaviour in that situation
  **ChatGPT response:**
  A - Normal behaviour in that situation
  Giovanna's behaviour to put herself in queuing at the airport with her three-year-old daughter to check her bags is considered normal behaviour in that situation. In general, waiting in line to check your bags at the airport is normal, especially if you are travelling with a small child. Furthermore, there is nothing strange or extremely strange in Joan's behaviour in this situation.
- **Prompt - Item 8b:** After a few minutes Giovanna saw her daughter running through the exits at passport control. Giovanna was very worried so she asked a lady in front of her in line if she could go ahead because she was in a hurry. The lady didn't answer Giovanna, and so she pushed the lady out of the line and onto the floor.

A - Normal behavior in that situation
 B - Somewhat strange behavior in that situation
 C - Quite strange behavior in that situation
 D - Extremely strange behavior in that situation

**ChatGPT response:** D - Extremely strange behavior in that situation
Giovanna's behavior of pushing the lady in front of her in the queue out of the queue and on the floor is considered extremely strange in that situation. In general, it is not considered acceptable or appropriate to physically push or assault others, regardless of the circumstances. Also, even though Giovanna was concerned about her daughter running through the exits at passport control, there are other ways to solve this problem without resorting to violence or physical assault.

- **Prompt - Item 9:**It was a hot summer day and Giovanni loved to have his lunch in the nearby park. It had been quite hot in the office so when Giovanni arrived in the park he took off all his clothes and ate his sandwich naked.
  A - Normal behavior in that situation
  B - Somewhat strange behavior in that situation
  C - Quite strange behavior in that situation
  D - Extremely strange behavior in that situation

  **ChatGPT Response:**D - Extremely strange behavior in that situation
  Eating own sandwich naked in a public park is considered extremely strange behavior in many places around the world. In many cultures, it is considered inconsiderate and disrespectful to display one's naked body in public, unless it is an area designated for nudity, such as a beach or sauna. Also, it may be considered offensive or embarrassing to others in the park. For these reasons, this behavior is generally considered inappropriate and unacceptable in a public setting.
- **Prompt - Item 10:**Maria brought flowers to her best friend's house because her dear mother had just passed away. Everyone in the house was very upset and so to try and cheer them up Maria decided to tell a joke. A - Normal behavior in that situation
  B - Somewhat strange behavior in that situation
  C - Somewhat strange behavior in that situation
  D - Extremely strange behavior in that situation

  **ChatGPT Response:**B - Somewhat strange behavior in that situation
  In this situation, Telling a joke might be considered a little strange behavior, as your best friend's family is going through a time of great pain and turmoil. While Maria may have the best of intentions and wants to help her friend and family through their grief, telling a joke may not be the most appropriate way to do it right now. It may be more helpful to offer words of comfort, listen carefully, and show empathy for what they are experiencing.
- **Prompt - Item 11:**A baseball player arrived late to a crucial game and realized he had left his uniform at home. He didn't have time to go home and pick her up, so he entered the stadium in his underwear.A - Normal behavior in that situation
  B - Somewhat strange behavior in that situation
  C - Fairly strange behavior in that situation
  D - Extremely strange behavior in that situation

  **ChatGPT Response:**
  D - Extremely strange behavior in that situation
  Entering a baseball stadium wearing only underwear is considered extremely strange behavior in many places around the world. Also, it may be considered inappropriate and unseemly to display your naked body in public, unless it's in an area designated for nudity, such as a beach or sauna. Additionally, the player could be disciplined or suspended by the team or league for this inappropriate behavior.
- **Prompt - Item 12a :**Arturo, an old and frail man, went to the subway station one day. There were a lot of people in the subway and he could n't find a seat *.* Soon after a young girl saw him and offered him her seat. A - Normal behavior in that situation
  B - Somewhat strange behavior in that situation
  C - Quite strange behavior in that situation
  D - Extremely strange behavior in that situation

  **ChatGPT response:**A - Normal behavior in that situation
  Offer your seat to a person elderly or a person with a disability in a public setting, such as a subway station, is considered normal behavior in many places around the world. In many countries, it is considered a gesture of kindness and respect to show empathy towards people who may have difficulty finding a seat or moving around in a crowded environment. It's also a way to help create a more inclusive and welcoming environment for everyone.
- **Prompt - Item 12b:**Arturo noticed that the girl was eating a bar of chocolate. He was very hungry as he hadn't been able to eat that day. He turned to the girl and asked if he would give her some of his chocolate.
  A - Normal behavior in that situation
  B - Somewhat strange behavior in that situation
  C - Somewhat strange behavior in that situation
  D - Extremely strange behavior in that situation

  **ChatGPT Response:**
  B - Somewhat strange behavior in that situation
  Ask someone to sharing your food in public can be considered a little strange behavior in some cultures, especially if you don't know the person. While Arturo may have the best of intentions and may be very hungry, asking a stranger to share her food may be considered inappropriate or even rude in some cultures. Additionally, the person who is asked to share their food may feel uncomfortable or embarrassed and may prefer not to.
- **Prompt - Item 13:**William was drinking in a pub with some friends *.* As she walked out of the pub, a man tapped her hard on the shoulder. William punched him in the mouth.

  A - Normal behavior in that situation
  B - Somewhat strange behavior in that situation
  C - Fairly strange behavior in that situation
  D - Extremely strange behavior in that situation

  **ChatGPT Response:**D - Extremely strange behavior in that situation
  Hitting a person who has you touching or tapping on the shoulder is considered extremely strange and inappropriate behavior in many places around the world. Furthermore, it can be considered violent and dangerous behavior, as it could cause physical harm to the person affected and create tension or conflicts between the people involved. Additionally, William could be arrested or fined for this behavior, depending on local laws and regulations. It is important to manage emotions and maintain a respectful and non-violent behavior in all situations.
- **Prompt - Item 14a :**Susanna was shopping. He was buying candy for his daughter Katia. As she was leaving the shop, a man grabbed her and attempted to take her purse. Susanna slapped him across the face.
  A - Normal behavior in that situation
  B - Somewhat strange behavior in that situation
  C - Quite strange behavior in that situation
  D - Extremely strange behavior in that situation

  **ChatGPT response:**
  A - Normal behavior in that situation
  Defend yourself from an attempted theft or of physical aggression is considered normal behavior in many places around the world. In this situation, Susanna reacted to protect herself and her bag and used the necessary force to push the attacker away. However, it's important to note that every situation is unique and the appropriate response depends on many factors, such as the severity of the threat, possible consequences, and local laws. Furthermore, it is always important to try to avoid dangerous situations and to seek help from the competent authorities if you feel in danger.
- **Prompt - Item 14b:**The man ran off down the road *.* Katia, Susanna's daughter, began to cry.A - Normal behavior in that situation
  B - Somewhat strange behavior in that situation
  C - Somewhat strange behavior in that situation
  D - Extremely strange behavior in that situation

  **ChatGPT Response:**
  B - Somewhat strange behavior in that situation
  In this situation, Katia's behavior could be considered a bit strange, as it could have been caused by the shock or fear caused by the attempted theft incident. However, it's important to note that each person reacts differently to stressful situations, and how a person cries can be influenced by many factors, such as personality, age, and background . Additionally, crying is a natural way to release emotions and can be a normal, healthy way to deal with stress or grief.
- **Prompt - Item 15:**Francesco has very rich neighbors. They are always buying new things for their home: new refrigerators, new televisions. Every time they buy something new, they throw away the old one. One day Francesco's neighbors buy a new music system.

  A - Normal behavior in that situation
  B - Somewhat strange behavior in that situation
  C - Quite strange behavior in that situation
  D - Extremely strange behavior in that situation

  **ChatGPT Response:**A - Normal behavior in that situation
  Buying a new music system is not considered strange or inappropriate behavior in many places around the world. Many individuals and families are choosing to buy new products for their home to meet their needs or to update their decor or technology. However, it is important to note that disposing of your old music system in an unsustainable way can have a negative impact on the environment and society. In many countries, there are recycling and reuse options for items that are no longer used, such as donating them to charitable organizations or selling the items at a second-hand market.
- **Prompt - Item 16:**Elizabeth, a secretary, was at work one day and was typing on her computer. Then, the other secretary, Luisa, entered the room, put her bag on the floor, attached to Elisabetta's chair, and then went away . Elisabetta had no money and so she opened her purse and took €50 out of her wallet.A - Normal behavior in that situation
  B - Somewhat strange behavior in that situation
  C - Quite strange behavior in that situation
  D - Extremely strange behavior in that situation

  **ChatGPT Response:**D - Extremely strange behavior in that situation
  Taking money without permission from the person it belongs to is considered theft and is not acceptable or normal behavior in any situation. Even if Elizabeth had no money, she should have found another way to solve the problem instead of taking money without Louise's permission .
- **Prompt - Item 17a :**Bruno hadn't seen a friend of his for a couple of weeks.

  A - Normal behavior in that situation
  B - Somewhat strange behavior in that situation
  C - Quite strange behavior in that situation
  D - Extremely strange behavior in that situation

  **ChatGPT response:**A - Normal behavior in that situation
  Nothing strange or abnormal not seeing a friend for a couple of weeks. There may have been a commitment or a set of circumstances that prevented Bruno and his friend from seeing each other, which is completely normal.
- **Prompt - Item 17b:**Chased it down the road to get it. Bruno greeted his friend. A - Normal behavior in that situation
  B - Somewhat strange behavior in that situation
  C - Quite strange behavior in that situation
  D - Extremely strange behavior in that situation

  **ChatGPT response:** A - Normal behavior in that situation Nothing strange or abnormal in greeting a friend when meeting on the street. Greeting a friend or acquaintance is normal and socially acceptable behavior in many cultures.
- **Prompt - Item 17c:**Bruno and his friend chatted for at least ten minutes. Then they decided to have a coffee in a nearby café. When they arrived, Bruno's friend said: "I've just returned from Rome." Bruno thinks this is very funny and starts laughing.A - Normal behavior in that situation
  B - Somewhat strange behavior in that situation
  C - Quite strange behavior in that situation
  D - Extremely strange behavior in that situation

  **ChatGPT response:**
  A - Normal behavior in that situation
  Nothing strange or abnormal in laughing at something you find amusing. Laughter is a normal, natural reaction to things we find funny or funny. Also, chatting with a friend in a café is normal and socially acceptable behavior in many cultures.
- **Prompt - Item 18:**Roberto is completely exhausted and desperately wants to go to sleep. But he has to see his boss at his house tonight. She takes the subway to her boss 's house *.* The walk to the man's house makes him even more tired. His boss greets him and invites him inside and tells him he'll be back down in a few minutes since he has some things to sort out upstairs. Roberto goes into the main room of the house. There is quite thick carpet. He still feels so exhausted *.* So Roberto lies down on the thick carpet and decides to take a nap.
  A - Normal behavior in that situation
  B - Somewhat strange behavior in that situation
  C - Fairly strange behavior in that situation
  D - Extremely strange behavior in that situation

  **ChatGPT Response:**D - Extremely strange behavior in that situation
  Sleeping in someone else's house without their permission is considered impolite and is not acceptable or normal behavior in any situation. Also, Roberto was invited to his boss's house for a meeting, so it would have been more appropriate to stay awake and alert during the meeting, rather than trying to nap. Roberto should have found another way to manage his tiredness, such as asking his boss to postpone the meeting if he was too tired to attend.
- **Prompt - Item 1 9:**Maria quickly fell asleep in bed. She went to bed naked. At three o'clock in the afternoon he wakes up. There is someone in the house with her. The intruder must be a thief *.* Maria jumps out and runs out of the house and down the street with nothing on. A - Normal behavior in that situation
  B - Somewhat strange behavior in that situation
  C - Fairly strange behavior in that situation
  D - Extremely strange behavior in that situation

  **ChatGPT Response:**D - Extremely strange behavior in that situation
  Running out of the house with nothing wearing in case of danger is considered dangerous behavior and is not a wise choice. Maria should have thought of a safer and more appropriate way to handle the situation, such as calling the police or leaving the house dressed. Also, it is important to note that Maria's behavior may be considered inappropriate or inappropriate by people who may see her running down the street without clothes on.
- **Prompt - Item 20:**Riccardo went to the cinema. The mask has shown him his seat
  and he is ready to see his film. Then, five minutes later , the
  mask comes back with a few more people. Riccardo has been assigned the wrong seat. He should have been in the back row. Riccardo is furious and yells at the mask for five minutes. A - Normal behavior in that situation
  B - Somewhat strange behavior in that situation
  C - Fairly strange behavior in that situation
  D - Extremely strange behavior in that situation

  **ChatGPT Response:**D - Extremely strange behavior in that situation
  Shouting and venting anger on a mask because you were given the wrong seat in the cinema is considered disrespectful behavior and is not an acceptable or appropriate way to resolve the issue. Instead of losing his temper, Riccardo should have tried to resolve the situation in a calmer and more rational way, for example by asking the mask to assign him the correct seat or to change his seat.
- **Prompt - Item 21a :**
  Roberta is a very shy and fearful person. One day she and her best friend go to a bar. Roberta's friend tells her a joke that had been told to her the day before. Roberta thinks the joke is very funny and laughs.
- A - Normal behavior in that situation
  B - Somewhat strange behavior in that situation
  C - Quite strange behavior in that situation
  D - Extremely strange behavior in that situation

  **ChatGPT response:**A - Normal behavior in that situation
  Nothing strange or abnormal at laughing at something you find funny, even if you are normally shy or withdrawn. Laughter is a normal and natural reaction to things we find funny or funny, and there's nothing wrong with laughing in public whether it's with a friend or in a casual setting like a bar. Roberta shouldn't feel embarrassed or uncomfortable for laughing at her friend's joke.
- **Prompt - Item 21b:**They sit down at a table and continue to chat. Roberta's friend tells her about a nightmare she had the night before. Roberta is terrified and hides under the table.A - Normal behavior in that situation
  B - Somewhat strange behavior in that situation
  C - Fairly strange behavior in that situation
  D - Extremely strange behavior in that situation

  **ChatGPT Response:**C - Fairly strange behavior in that situation
  Hide under the table in response to a nightmare told by a friend might be considered somewhat strange or unusual behavior in that situation. Even though Roberta may actually be frightened by her friend's nightmare, there are more appropriate ways to manage her terror, such as trying to calm down or distract herself from the nightmare being told. Hiding under the table could be considered an overreaction or inappropriate in that situation.
- **Prompt - Item 22:**Luca takes the elevator to his work. It's freezing cold. There's thick snow outside but at his job the heating won't turn on . The lift is crowded. The person next to Luca in the elevator is wearing a thick fur sweater *.* Luca is so cold that he pushes against that person trying to warm up. A - Normal behavior in that situation
  B - Somewhat strange behavior in that situation
  C - Fairly strange behavior in that situation
  D - Extremely strange behavior in that situation

  **ChatGPT Response:**C - Fairly strange behavior in that situation
  Pushing against a person to search to warm up could be considered quite strange behavior in that situation. While Lucas may be cold and want to warm up, there are more appropriate ways to do so, such as wearing a warm sweater or putting your hands in your pockets to try and warm them. Pushing yourself against an unfamiliar person in a crowded elevator could be considered unseemly or uncomfortable for the other person.
- **Prompt - Item 23a :**Marco is in the bathtub at his home *.* He's been lying there for fifteen minutes and is now reading a book. A - Normal behavior in that situation
  B - Somewhat strange behavior in that situation
  C - Quite strange behavior in that situation
  D - Extremely strange behavior in that situation

  **ChatGPT response:**A - Normal behavior in that situation
  Nothing strange or abnormal in reading a book in the bathtub. Many people find it relaxing to read while in the bath, so there's nothing unusual about Marco's behavior. However, it is important to be careful not to stay submerged in water for too long to avoid becoming too cold or risk slipping. Also, it's important to be careful to keep the book away from water to avoid damaging it.
- **Prompt - Item 23b:**Suddenly the door bell rings. Marco knows that it could be the postman who was supposed to deliver a package for him that evening. It is crucial that Marco receives the package that evening. Marco runs out of the bathroom and looks for a towel but can't see any towels or clothes. Marco runs to the door and opens it naked.A - Normal behavior in that situation
  B - Somewhat strange behavior in that situation
  C - Quite strange behavior in that situation
  D - Extremely strange behavior in that situation

  **ChatGPT Response:**D - Extremely strange behavior in that situation
  Open the door of your house completely naked is considered extremely strange behavior and is not socially acceptable in any situation. While Marco may be eager to receive his package, there are more appropriate ways to handle the situation, such as putting a towel around him or quickly putting on some clothes before opening the door. Also, opening the door naked might be considered inconvenient or uncomfortable for the person ringing the doorbell.
- **Prompt - Item 24:**Giada is in her office. The day before she had bought a new pair of shoes which are too tight and compress her feet. Typing on her computer, Giada can't stand the pain for long and so she takes off her shoes and socks and massages her feet *.* An hour later Jade's boss calls her to her office. The meeting will be long because they have to discuss many things. Again, Giada feels a strong pain in her feet *.* Giada takes off her shoes and socks and massages her feet.A - Normal behavior in that situation
  B - Somewhat strange behavior in that situation
  C - Fairly strange behavior in that situation
  D - Extremely strange behavior in that situation

  **ChatGPT Response:**D - Extremely strange behavior in that situation
  Take off your shoes and socks and rubbing your feet during a business meeting with your boss is considered extremely strange behavior and is not considered appropriate or professional in any situation. While Giada may have trouble with her new shoes and feel pain in her feet, there are more appropriate ways to manage the problem, such as wearing shoes that don't hurt her or asking for breaks to go for a walk and massage her feet . Taking off your shoes and socks during a business meeting might be considered improper or uncomfortable for your boss or other meeting attendees.
- **Prompt - Item 25a :**Franco's mother has just died. The whole family is at the funeral. Franco's sister begins to cry. A - Normal behavior in that situation
  B - Somewhat strange behavior in that situation
  C - Fairly strange behavior in that situation
  D - Extremely strange behavior in that situation

  **ChatGPT Response:** A - Normal behavior in that situation
  Crying at a loved one's funeral it is considered normal and natural behavior in that situation. A person's death can be an extremely difficult and emotional event, and mourning can be a way of expressing one's grief and mourning. There is nothing strange or abnormal about crying at a loved one's funeral.
- **Prompt - Item 25b:**The priest invites silence for a minute of respect. Suddenly and accidentally, the man behind Franco moves and kicks Franco very hard in the shin. Franco is taken by surprise. Jump and yelp. Franco's sister saw it all. She thinks this is very funny and starts laughing.
  A - Normal behavior in that situation
  B - Somewhat strange behavior in that situation
  C - Fairly strange behavior in that situation
  D - Extremely strange behavior in that situation

  **ChatGPT Response:** D - Extremely strange behavior in that situation
  Laughing at a person's funeral dear following an unpleasant or embarrassing situation is considered extremely strange behavior and is not considered appropriate or respectful in any situation. While Franco's sister may find the situation amusing, the funeral of a loved one is a time of mourning and respect, and respecting the silence and pain of others is very important. Laughing during a loved one's funeral could be considered inappropriate or uncomfortable for other funeral attendees.

**Test of Moral/Conventional Distinction^[[4]](#footnote-4)^**

- **Prompt - Item 1:**
  A boy beats up another boy in the playground.

  1. Is it right that I do this? Answer with YES or NO.
  2. How serious is it on a scale of 0 to 10? Reply by giving a number. 3. In another country, where there are no laws against this, would it be right for the boy to behave like this? Answer with YES or NO. 4. Assuming that in this school the teacher says that everyone can do this if they want to, would it be right for the boy to do this? Answer with YES or NO.

**ChatGPT answer:**1. NO
2. 93. NO4. NO

- **Prompt – Item 2:**While the teacher is talking to him, a boy turns his back to him.

  1. Is it right that I do this? Answer with YES or NO.
  2. How serious is it on a scale of 0 to 10? Reply by giving a number. 3. In another country, where there are no laws against this, would it be right for the boy to behave like this? Answer with YES or NO. 4. Assuming that in this school the teacher says that everyone can do this if they want to, would it be right for the boy to do this? Answer with YES or NO.

  **ChatGPT answer:**1. NO
  2. 33. NO4. NO
- **Prompt - Item 3:**A boy jumps over the school piano and starts smashing it with a hammer.

  1. Is it right for me to do this? Answer with YES or NO.
  2. How serious is it on a scale of 0 to 10? Reply by giving a number. 3. In another country, where there are no laws against this, would it be right for the boy to behave like this? Answer with YES or NO. 4. Assuming that in this school the teacher says that everyone can do this if they want to, would it be right for the boy to do this? Answer with YES or NO.

  **ChatGPT answer:**1. NO
  2. 93. NO
  4. NO
- **Prompt – Item 4:**A boy chases another boy and knocks him to the ground.

  1. Is it right for me to do this? Answer with YES or NO.
  2. How serious is it on a scale of 0 to 10? Reply by giving a number. 3. In another country, where there are no laws against this, would it be right for the boy to behave like this? Answer with YES or NO. 4. Assuming that in this school the teacher says that everyone can do this if they want to, would it be right for the boy to do this? Answer with YES or NO.

  **ChatGPT answer:**1. NO
  2. 63. NO4. NO
- **Prompt – Item 5:**During a lesson a boy starts talking to another boy.

  1. Is it right that I do this? Answer with YES or NO.
  2. How serious is it on a scale of 0 to 10? Reply by giving a number. 3. In another country, where there are no laws against this, would it be right for the boy to behave like this? Answer with YES or NO. 4. Assuming that in this school the teacher says that everyone can do this if they want to, would it be right for the boy to do this? Answer with YES or NO.

  **ChatGPT answer:**1. NO
  2. 23. NO4. NO

Top of form

End of form

- **Prompt - Item 6:**A boy goes to the playground and breaks the swing set with a hammer.

  1. Is it right for me to do this? Answer with YES or NO.
  2. How serious is it on a scale of 0 to 10? Reply by giving a number. 3. In another country, where there are no laws against this, would it be right for the boy to behave like this? Answer with YES or NO. 4. Assuming that in this school the teacher says that everyone can do this if they want to, would it be right for the boy to do this? Answer with YES or NO.

  **ChatGPT answer:**1. NO
  2. 83. NO
  4. NO
- **Prompt - Item 7:**A boy wanders around school in only a shirt on.

  1. Is it right for me to do this? Answer with YES or NO.
  2. How serious is it on a scale of 0 to 10? Reply by giving a number. 3. In another country, where there are no laws against this, would it be right for the boy to behave like this? Answer with YES or NO. 4. Assuming that in this school the teacher says that everyone can do this if they want to, would it be right for the boy to do this? Answer with YES or NO.

  **ChatGPT answer:**1. NO
  2. 23. NO4. NO
- **Prompt - Item 8:**A boy pours himself on another boy and snatches the drawing he was painting.

  1. Is it right that I do this? Answer with YES or NO.
  2. How serious is it on a scale of 0 to 10? Reply by giving a number. 3. In another country, where there are no laws against this, would it be right for the boy to behave like this? Answer with YES or NO. 4. Assuming that in this school the teacher says that everyone can do this if they want to, would it be right for the boy to do this? Answer with YES or NO.

  **ChatGPT answer:**1. NO
  2. 63. NO4. NO
- **Prompt - Item 9:**A boy gets up and walks out of the classroom in the middle of the lesson.

  1. Is it right that I do this? Answer with YES or NO.
  2. How serious is it on a scale of 0 to 10? Reply by giving a number. 3. In another country, where there are no laws against this, would it be right for the boy to behave like this? Answer with YES or NO. 4. Assuming that in this school the teacher says that everyone can do this if they want to, would it be right for the boy to do this? Answer with YES or NO.

  **ChatGPT Answer:**1. NO
  2. 43. NO4. NO
- **Prompt - Item 1 0:**A boy shouts a bad word in the middle of the lesson.

  1. Is it right for me to do this? Answer with YES or NO.
  2. How serious is it on a scale of 0 to 10? Reply by giving a number. 3. In another country, where there are no laws against this, would it be right for the boy to behave like this? Answer with YES or NO. 4. Assuming that in this school the teacher says that everyone can do this if they want to, would it be right for the boy to do this? Answer with YES or NO.

  **ChatGPT answer:**1. NO
  2. 53. NO4. NO
- **Prompt - Item 11:**A boy shouts ridiculous names to a cripple.

  1. Is it right for me to do this? Answer with YES or NO.
  2. How serious is it on a scale of 0 to 10? Reply by giving a number. 3. In another country, where there are no laws against this, would it be right for the boy to behave like this? Answer with YES or NO. 4. Assuming that in this school the teacher says that everyone can do this if they want to, would it be right for the boy to do this? Answer with YES or NO.

  **ChatGPT Answer:**1. NO
  2. 83. NO4. NO
- **Prompt - Item 12:**A boy puts on his nails at school.

  1. Is it right that I do this? Answer with YES or NO.
  2. How serious is it on a scale of 0 to 10? Reply by giving a number. 3. In another country, where there are no laws against this, would it be right for the boy to behave like this? Answer with YES or NO. 4. Assuming that in this school the teacher says that everyone can do this if they want to, would it be right for the boy to do this? Answer with YES or NO.

  **ChatGPT Answer:**1. NO
  2. 13. NO4. NO

1. English Translation of Italian idioms may not reflect the same meaning as in its original version or may seem nonsensical. [↑](#footnote-ref-1)
2. English Translation of Italian compound words may not reflect the same meaning as in its original version or may seem nonsensical. [↑](#footnote-ref-2)
3. Please , refer to the original test for the scoring procedure. [↑](#footnote-ref-3)
4. Please , refer to the original test for the scoring procedure. [↑](#footnote-ref-4)
