# Supplementary material for: Challenging large language models’ “intelligence” with human tools: A neuropsychological investigation in Italian language on prefrontal functioning
Source: Heliyon. 2024 Oct 3;10(19):e38911. doi: 10.1016/j.heliyon.2024.e38911 (PMC11490853; doi:10.1016/j.heliyon.2024.e38911)
Supplement: Multimedia component 4 [file mmc4.docx]

**Performance of GPT-4, Claude2, and Llama2 models on Prefrontal tasks**

**Table 1S.** Raw score, percentile ranks, and qualitative evaluations of the performance obtained by GPT-4 in the cognitive functions investigated.

| **Cognitive Function** | **Test** | **GPT-4**  **Raw Scores** | **Percentile Ranks** | **Qualitative evaluation of the performance** |
| --- | --- | --- | --- | --- |
| **Verbal Reasoning:**   - *Absurdities* - *Intruders* - *Relationships* - *Differences* - *Idiomatic Expressions* - *Family Relations* - *Classifications* | **VRT** | **97/98**  *14/14*  *14/14*  *14/14*  *14/14*  *14/14*  *13/14*  *14/14* | *66.97^th^*  *58.55^th^*  62.51^st^  *52.35^th^*  *62.89^th^*  *76.61^st^*  *69.01^st^*  *51.44^th^* | **Norm**  *Norm*  *Norm*  *Norm*  *Norm*  *Norm*  *Norm*  *Norm* |
| **Cognitive Estimation:**   - *Absolute error score* - *Bizarreness score* | **CET** | 8/41  1/21 | *80^th^* ^a^  *45^th^ - 70^th^* ^a^ | *Norm*  *Norm* |
| **Metaphors Comprehension** | **MC** | 33/40 | > 50^th^ | Norm |
| **Idioms Comprehension** | **IC** | 34/40 | > 50^th^ | Norm |
| **Anaphoric Referencing** | **Winograd Schema** | 19/20 | - | Norm |
| **Planning** | **ToL** | 8/36 | < 1^st^ | Severely Impaired |
| **Inhibition:**   - *Error A:* - *Error B:* | **HSCT** | 0  7 | >50^th^  5^th^-25^th^ | *Norm*  *Low-Norm* |
| **Insight:** | **CRA** | 49/122 | 52.16^th^ | Low-normal |

*Note.* VRT: Verbal Reasoning Test; CET: Cognitive estimation task; MC: Metaphor comprehension; IC: Idioms comprehension; ToL: Tower of London; HSCT: Hayling Sentence Completion Task; CRA: Compound Remote Association problems.
^a^ Absolute error and bizarreness scores obtained according to the original validation fell within the 90^th^-95^th^ and 70^th^-75^th^ percentile range, respectively. To report these results in terms of performance accuracy, in a homologous manner compared with the results of the other tests shown in the same table, we reported the symmetric values of the percentile computed as 100-p, whereas p is the percentile of interest.

**Table 2S.** Raw score, percentile ranks, and qualitative evaluations of the performance obtained by Claude2 in the cognitive functions investigated.

| **Cognitive Function** | **Test** | **Claude2**  **Raw Scores** | **Percentile Ranks** | **Qualitative evaluation of the performance** |
| --- | --- | --- | --- | --- |
| **Verbal Reasoning:**   - *Absurdities* - *Intruders* - *Relationships* - *Differences* - *Idiomatic Expressions* - *Family Relations* - *Classifications* | **VRT** | **84/98**  *5/14*  *10/14*  *14/14*  *14/14*  *13/14*  *14/14*  *14/14* | *66.97^th^*  *0.14^th^*  9.36^th^  *52.35^th^*  *62.89^th^*  *59.44^th^*  *79.84^th^*  *51.44^th^* | **Norm**  *Impaired*  *Borderline*  *Norm*  *Norm*  *Norm*  *Norm*  *Norm* |
| **Cognitive Estimation:**   - *Absolute error score* - *Bizarreness score* | **CET** | 14/41  4/21 | *20^th^* ^a^  *5^th^ - 10^th^* ^a^ | *Low-Norm*  *Borderline* |
| **Metaphors Comprehension** | **MC** | 31/40 | > 50^th^ | Norm |
| **Idioms Comprehension** | **IC** | 37/40 | > 50^th^ | Norm |
| **Anaphoric Referencing** | **Winograd Schema** | 18/20 | - | Norm |
| **Planning** | **ToL** | 2/36 | < 1^st^ | Severely Impaired |
| **Inhibition:**   - *Error A:* - *Error B:* | **HSCT** | 0  5 | >50^th^  25^th^ | *Norm*  *Low-Norm* |
| **Insight:** | **CRA** | 44/122 | 45.06^th^ | Low-norm |

*Note.* VRT: Verbal Reasoning Test; CET: Cognitive estimation task; MC: Metaphor comprehension; IC: Idioms comprehension; ToL: Tower of London; HSCT: Hayling Sentence Completion Task; CRA: Compound Remote Association problems.
^a^ Absolute error and bizarreness scores obtained according to the original validation fell within the 90^th^-95^th^ and 70^th^-75^th^ percentile range, respectively. To report these results in terms of performance accuracy, in a homologous manner compared with the results of the other tests shown in the same table, we reported the symmetric values of the percentile computed as 100-p, whereas p is the percentile of interest.

**Table 3S.** Raw score, percentile ranks, and qualitative evaluations of the performance obtained by Llama2 in the cognitive functions investigated.

| **Cognitive Function** | **Test** | **Llama2**  **Raw Scores** | **Percentile Ranks** | **Qualitative evaluation of the performance** |
| --- | --- | --- | --- | --- |
| **Verbal Reasoning:**   - *Absurdities* - *Intruders* - *Relationships* - *Differences* - *Idiomatic Expressions* - *Family Relations* - *Classifications* | **VRT** | **67/98**  *7/14*  *6/14*  *12/14*  *13/14*  *4/14*  *12/14*  *13/14* | *1.64^th^*  *1.45^th^*  0.15^th^  *24.7^th^*  *62.89^th^*  *0^th^*  *56.2^nd^*  *28.33^rd^* | **Impaired**  *Impaired*  *Impaired*  *Low-Norm*  *Norm*  *Impaired*  *Norm*  *Low-Norm* |
| **Cognitive Estimation:**   - *Absolute error score* - *Bizarreness score* | **CET** | 8/41  1/21 | *5^th^-10^th^* ^a^  *0^th^* ^a^ | *Borderline*  *Impaired* |
| **Metaphors Comprehension** | **MC** | 21/40 | 16.5^th^ | Low-Norm |
| **Idioms Comprehension** | **IC** | 7/40 | < 5^th^ | Impaired |
| **Anaphoric Referencing** | **Winograd Schema** | 13/20 | - | Mildly impaired |
| **Planning** | **ToL** | 0/36 | < 1^st^ | Severely Impaired |
| **Inhibition:**   - *Error A:* - *Error B:* | **HSCT** | 0  6 | >50^th^  5^th^-25^th^ | *Norm*  *Low-Norm* |
| **Insight:** | **CRA** | 4/122 | 6.18^th^ | Borderline |

*Note.* VRT: Verbal Reasoning Test; CET: Cognitive estimation task; MC: Metaphor comprehension; IC: Idioms comprehension; ToL: Tower of London; HSCT: Hayling Sentence Completion Task; CRA: Compound Remote Association problems.
^a^ Absolute error and bizarreness scores obtained according to the original validation fell within the 90^th^-95^th^ and 70^th^-75^th^ percentile range, respectively. To report these results in terms of performance accuracy, in a homologous manner compared with the results of the other tests shown in the same table, we reported the symmetric values of the percentile computed as 100-p, whereas p is the percentile of interest.

**Table 4S**. GPT-4’s Performance in Social Cognition Tasks: Raw Scores and Qualitative Performance Evaluations.

| **Cognitive Function**  **(Tested with the SC battery)** | **GPT-4**  **Raw Scores** | **Cut-off** | **Qualitative evaluation of the performance** |
| --- | --- | --- | --- |
| **Theory of Mind:** | 12/13 | ≽12 | Norm |
| **Emotion Attribution:**   - *Sadness* - *Fear* - *Embarrassment* - *Disgust* - *Happiness* - *Anger* - *Envy* | 8/10  10/10  11/12  3/3  10/10  8/10  2/3 | ≽6  ≽8  ≽8  ≽2  ≽10  ≽6  ≽1 | Norm  Norm  Norm  Norm  Norm  Norm  Norm |
| **Social Situations:**   - *Normative Behaviour* - *Violation* - *Severity of the Violation* | 15/15  24/35  57/75 | ≽13  ≽22  ≽45 | Norm  Norm  Norm |
| **Moral Judgements:**   - *Moral Behaviours: not allowed* - *Moral Behaviours: severity* - *Moral Behaviours: not allowed with no rules* - *Conventional Behaviours: not allowed* - *Conventional Behaviours: severity* - *Conventional Behaviours: not allowed with no rules* | 6  58  12  5  34  10 | ≽6  ≽39  ≽11  ≽5  ≽20  ≽6 | Norm  Norm  Norm  Norm  Norm  Norm |

**Table 5S**. Claude2’s Performance in Social Cognition Tasks: Raw Scores and Qualitative Performance Evaluations.

| **Cognitive Function**  **(Tested with the SC battery)** | **Claude2**  **Raw Scores** | **Cut-off** | **Qualitative evaluation of the performance** |
| --- | --- | --- | --- |
| **Theory of Mind:** | 13/13 | ≽12 | Norm |
| **Emotion Attribution:**   - *Sadness* - *Fear* - *Embarrassment* - *Disgust* - *Happiness* - *Anger* - *Envy* | 5/10  10/10  10/12  3/3  8/10  5/10  3/3 | ≽6  ≽8  ≽8  ≽2  ≽10  ≽6  ≽1 | Mildly Impaired  Norm  Norm  Norm  Impaired  Mildly Impaired  Norm |
| **Social Situations:**   - *Normative Behaviour* - *Violation* - *Severity of the Violation* | 14/15  24/35  47/75 | ≽13  ≽22  ≽45 | Norm  Norm  Norm |
| **Moral Judgements:**   - *Moral Behaviours: not allowed* - *Moral Behaviours: severity* - *Moral Behaviours: not allowed with no rules* - *Conventional Behaviours: not allowed* - *Conventional Behaviours: severity* - *Conventional Behaviours: not allowed with no rules* | 6  56  12  5  27  19 | ≽6  ≽39  ≽11  ≽5  ≽20  ≽6 | Norm  Norm  Norm  Norm  Norm  Norm |

**Table 6S**. Llama2’s Performance in Social Cognition Tasks: Raw Scores and Qualitative Performance Evaluations.

| **Cognitive Function**  **(Tested with the SC battery)** | **Llama2**  **Raw Scores** | **Cut-off** | **Qualitative evaluation of the performance** |
| --- | --- | --- | --- |
| **Theory of Mind:** | 11/13 | ≽12 | Mildly impaired |
| **Emotion Attribution:**   - *Sadness* - *Fear* - *Embarrassment* - *Disgust* - *Happiness* - *Anger* - *Envy* | 7/10  8/10  19/12  2/3  10/10  5/10  2/3 | ≽6  ≽8  ≽8  ≽2  ≽10  ≽6  ≽1 | Norm  Norm  Norm  Norm  Norm  Mildly Impaired  Norm |
| **Social Situations:**   - *Normative Behaviour* - *Violation* - *Severity of the Violation* | 8/15  25/35  58/75 | ≽13  ≽22  ≽45 | Impaired  Norm  Norm |
| **Moral Judgements:**   - *Moral Behaviours: not allowed* - *Moral Behaviours: severity* - *Moral Behaviours: not allowed with no rules* - *Conventional Behaviours: not allowed* - *Conventional Behaviours: severity* - *Conventional Behaviours: not allowed with no rules* | 6  48  12  5  35  12 | ≽6  ≽39  ≽11  ≽5  ≽20  ≽6 | Norm  Norm  Norm  Norm  Norm  Norm |
